# Structure–activity relationships of hydrophobic small molecule irreversible inhibitors of tissue transglutaminase

**DOI:** 10.1039/d5md00815h

**Published:** 2025-11-11

**Authors:** Daniel A. Wallace, Sarah Tribe, Pauline Navals, Christina Bi, Tarasha Sharma, Jeffrey W. Keillor

**Affiliations:** a Department of Chemistry and Biomolecular Sciences, University of Ottawa Ottawa ON N6H 1N5 Canada jkeillor@uottawa.ca

## Abstract

Tissue transglutaminase (TG2) is both an enzyme and a G-protein that is implicated in many diseases, such that small molecule inhibitors of TG2 have broad potential as drugs or research tools. Previous work has demonstrated how the structure of EB-2-16, a highly potent irreversible inhibitor of TG2, has been optimised with respect to its warhead, tether and bridge moieties. In this work, we studied the structure–activity relationships of the pendant hydrophobic group of the scaffold. This confirmed the superior affinity conferred by the parent adamantyl moiety, over other cycloalkyl, aryl, biaryl and bridged biaryl groups. Additionally, some substituted adamantyl derivatives were shown to exhibit superior inhibitory efficiency over the parent inhibitor, with *k*_inact_/*K*_I_ values over 10^6^ M^−1^ min^−1^. The best inhibitors were shown to exhibit excellent lipid membrane permeability, but evaluation of their human hepatocyte stability revealed a sharp distinction between them. Despite the bromo- and iodoadamantyl derivatives being more efficient inhibitors, chloroadamantyl inhibitor 25b exhibits the best overall properties (*k*_inact_ = 1.69 min^−1^, *K*_I_ = 1.79 μM, *k*_inact_/*K*_I_ = 941 × 10^3^ M^−1^ min^−1^, *P*_e_ = 1.41 × 10^−6^ cm s^−1^, CL_int_ = 6.91 μL min^−1^/10^6^ cells) and suitability for potential applications *in vivo*.

## Abstract 2

Tissue transglutaminase (TG2) is both an enzyme and a G-protein that is implicated in many diseases, such that small molecule inhibitors of TG2 have broad potential as drugs or research tools.

## Graphical abstract

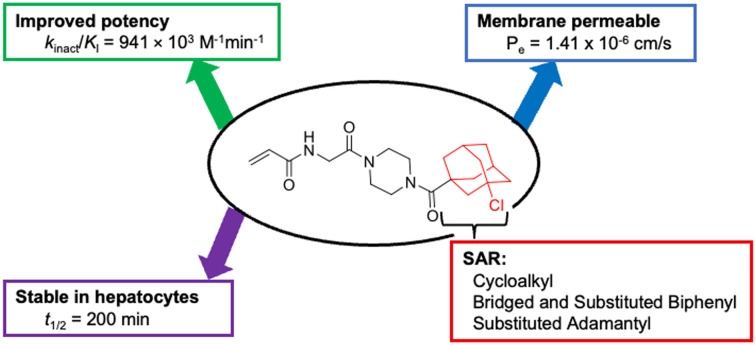

## Introduction

**Fig. 1:**
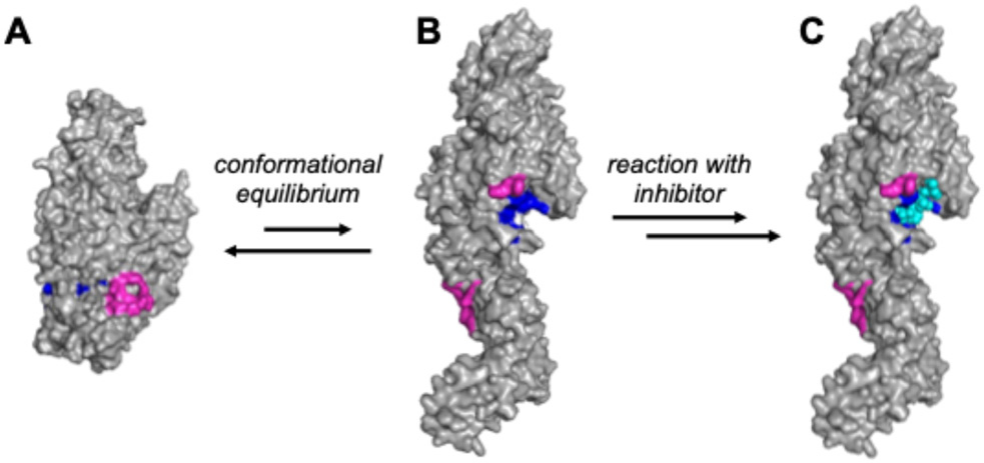
Conformational equilibrium and inhibition of TG2. A) Closed conformation (based on PDB code 4PYG)^4^ with assembled GTP-binding site shown in magenta. B) Open conformation (based on PDB code 2Q3Z) with substrate binding site shown in blue. C) Reaction with an irreversible inhibitor (cyan) locks TG2 in the open conformation.

Tissue transglutaminase 2 (TG2) is a dynamic enzyme present in all tissue types of the human body. It is one of eight enzymes belonging to the transglutaminase (TGase) family (TG1–TG7 and Factor XIII), all of which catalyze transamidation reactions between peptide-bound glutamine and lysine residues, leading to the formation of N^ε^(γ-glutaminyl)lysine isopeptide bonds. This reaction, leading to the cross-linking of protein substrates *via* the side chains of their Gln and Lys residues, is mediated by an active site comprising a cysteine catalytic triad. TG2 is also uniquely multifunctional among the TGases; in addition to its catalytic activity, it has the ability to act as a G-protein, participating in cell signalling pathways and hydrolyzing GTP to GDP in the process.^1,2^ These two functions are mutually exclusive, as each requires a unique (and substantially dissimilar) enzyme conformation.^3^ TG2 can act as a transamidase only in its “open” conformation (Fig. 1B), which is favoured in the extracellular space due to relatively high Ca^2+^ concentrations. Conversely, the lower Ca^2+^ environment within cells favours the “closed” conformation (Fig. 1A),^4^ allowing TG2 to function as a G-protein and disfavouring the catalysis of transamidation.^5^

Dysregulation of either the transamidase or G-protein functions of TG2 has been found to contribute to multiple diseases, including celiac disease,^6^ fibrosis,^7,8^ and several types of cancer.^9,10^ Individuals with these conditions generally have few—if any—available treatment options. Small molecule TG2 inhibitors have been shown to hold therapeutic potential for the treatment of these diseases, with ZED1227 advancing to clinical trials for the treatment of celiac disease.^11^ Thus, the development of novel therapeutic agents that target TG2 function is considered a crucial avenue of research.

Inhibition of TG2 has been studied extensively, prompting the discovery of inhibitors with a wide range of structures and modes of inhibition.^12–14^ Some of the most effective inhibitors found to date are small molecules that irreversibly inactivate TG2's catalytic activity. These small molecule inhibitors often have Michael-acceptor warheads (such as acrylamides and α,β-unsaturated esters) that form an enzyme-inhibitor adduct *via* 1,4-addition of the electrophilic warhead with the thiolate^15^ form of the enzyme's active site cysteine residue (Cys277).^16^ Evidence suggests this covalent bond formation not only prevents Cys277 from catalyzing transamidation reactions, but also locks the enzyme in its open conformation (Fig. 1C), thereby abolishing its G-protein activity, regardless of the overall size of the inhibitor.^17^ The ability to inhibit both of the main activities of TG2 makes this form of covalent inhibition very promising for future research on—and treatment of—celiac disease, fibrosis, and cancer.

The structure of TG2 in complex with a peptidic inhibitor (PDB code 2Q3Z),^5^ determined using X-ray crystallography, has been useful in the design of peptidomimetic inhibitors. A hydrophobic pocket near the enzyme's catalytic site, sometimes referred to as the “D-site,” was identified and recognized as having significant potential for binding hydrophobic moieties. Many peptidomimetic TG2 inhibitors have subsequently been designed to bear a hydrophobic moiety intended to promote affinity with this D-site.^18–25^ However, we have shown^26^ that this crystallographic structure cannot be used to account for the relative affinity of *small molecule* inhibitors through docking studies. The structure of TG2 in the absence of a ligand may differ from the crystallographic structure obtained after reaction with a peptidic inhibitor, such that the structure derived from the latter cannot be used to predict how the free enzyme may bind small molecule inhibitors that do not resemble the crystallographic ligand.

**Fig. 2:**
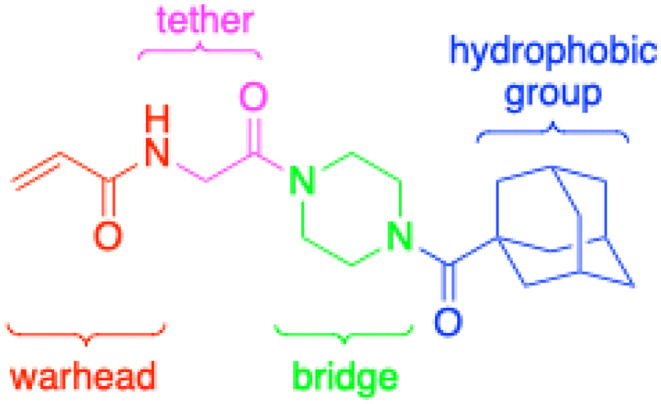
The four-part scaffold of inhibitor EB-2-16.

In the absence of structure-guided inhibitor design, we have undertaken more empirical studies in the exploration of small molecule inhibitor structures. One of the most effective TG2 inhibitors to date, known as EB-2-16 (Fig. 2), was discovered by the Griffin group in 2015.^16^ This small molecule possesses an acrylamide warhead attached to a glycine linker, piperazine bridge, and adamantane hydrophobic group. EB-2-16 is a highly efficient inhibitor that shows low toxicity and whose structure is amenable to facile modification. In previous studies, we have investigated the optimization of some of EB-2-16's four moieties. In a paper published by Mader *et al.*,^27^ a library of inhibitors with different warheads, including some reversible covalent, were evaluated to determine if any were superior to the original acrylamide. Only three warheads were found to be more efficient than acrylamide (chloroacrylamide, bromoacrylamide, and chloroacetamide), but each had higher off-target reactivity, suggesting the standard acrylamide remains the best warhead option. Similarly, in a study by Rangaswamy *et al.*,^26^ the glycine tether portion of the inhibitor was compared to tethers with longer carbon chains. Here, the original glycine tether was also the most effective. Recently, Ryan *et al.*^28^ explored variants of the bridge moiety and confirmed that the piperazine linker is superior to a large number of alkyl, cycloalkyl, and spirocyclic diamines. However, although some sections of this inhibitor scaffold have been investigated, a robust exploration into the hydrophobic moiety is still required. This missing scope inspired us to undertake the present SAR study, where we synthesized TG2 inhibitors bearing various hydrophobic groups on the scaffold of EB-2-16 and evaluated their inhibition efficiency. The most efficient inhibitors of this study were subjected to permeability and stability assays to further evaluate their feasibility as potential *in vivo* tools.

## Results and discussion

Our investigation into the structure–activity relationships of the hydrophobic moiety of this inhibitor scaffold was divided into three series wherein the hydrophobic group was varied, and the rest of the inhibitor remained unchanged. The first series replaced the parent adamantyl group with cycloalkyl and phenyl rings. The second series replaced adamantyl with modified biphenyl groups. The final series maintained the adamantyl group but bearing new substituents. Each novel inhibitor was evaluated under established continuous assay conditions,^28^ yielding the inhibition parameters *k*_inact_ and *K*_I_, which were combined to produce the inhibition efficiency parameter, *k*_inact_/*K*_I_. The parameters of each inhibitor were compared to each other and to the parent inhibitor EB-2-16, to obtain insight into which characteristics of the inhibitor's hydrophobic group are favourable for inhibition of TG2.

Though the adamantyl group of the parent compound was proposed^16^ to be one of the best hydrophobic moieties found for this scaffold, we noted that many other cyclic aliphatic moieties at this position had not been tested. To determine whether the bulky, three-dimensional adamantyl group is optimal, a series of inhibitors with different cycloalkyl hydrophobic groups was prepared, incorporating 3- to 7-membered rings (6a–e). Additionally, one inhibitor with a phenyl ring (6f) was included, to observe how aromaticity and planarity of the hydrophobic group may affect binding.

**Scheme 1:**
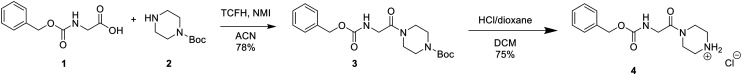
The synthesis of common intermediate 4.

**Scheme 2:**
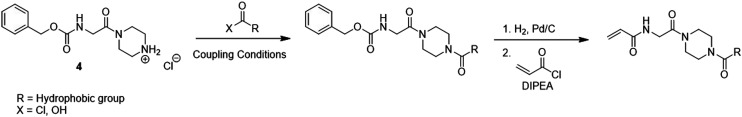
The general synthesis of inhibitors.

The synthesis of most inhibitors in this study passed through common intermediate 4, which was made *via* amide coupling between Cbz-glycine and Boc-piperazine, followed by removal of the Boc group to yield the hydrochloride salt of the free piperazine (Scheme 1). Next, this amine was coupled with each carboxylic acid bearing a hydrophobic group, under various conditions (Scheme 2). The final inhibitors were then obtained in two steps: removal of the Cbz group by hydrogenolysis, followed by coupling between the primary amine and acryloyl chloride to install the acrylamide warhead.

The inhibitors of this first series were synthesized *via* amide coupling between the free piperazine nitrogen of the common intermediate 4 and the commercially available cycloalkyl carboxylic acids to give 5a–f, followed by a deprotection reaction and a final coupling to the acrylamide warhead.

The cycloalkyl inhibitors of the first series did not show any improved inhibition over the parent compound, but a clear trend was observed showing a preference towards six-membered rings (cyclohexyl and phenyl), with inhibitory efficiencies decreasing as the rings become larger or smaller. Additionally, the increase in efficiency as the six-membered hydrophobic rings gained three-dimensional bulk (from phenyl to cycloalkyl to adamantyl) suggests the volume of the hydrophobic group also plays an important role in binding (Table 1).

**Table 1:** Kinetic data for inhibitors with cycloalkyl and phenyl hydrophobic groups

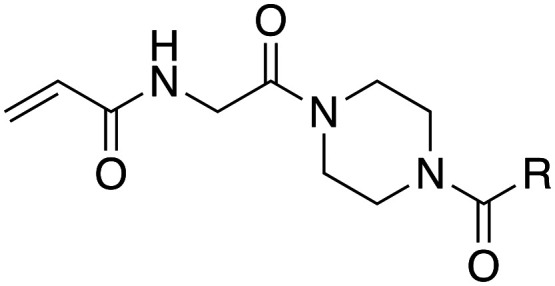				
Compound	R	*K*_I_ (μM)	*k*_inact_ (min^−1^)	*k*_inact_/*K*_I_ (× 10^3^ M^−1^ min^−1^)
6a	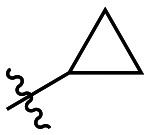	23.5 ± 6.5	0.36 ± 0.04	15.2 ± 4.6
6b	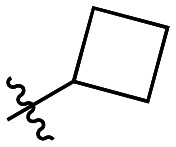	20.2 ± 3.5	0.51 ± 0.04	25.0 ± 4.7
6c	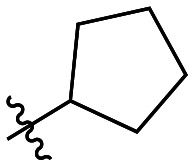	13.0 ± 1.6	0.52 ± 0.03	40.0 ± 5.3
6d	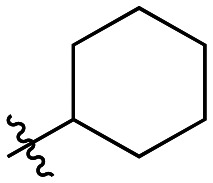	7.1 ± 1.3	1.06 ± 0.10	149 ± 31
6e	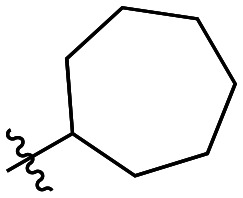	21.5 ± 11.0	0.59 ± 0.17	27.6 ± 16.1
6f	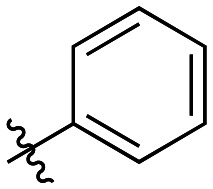	8.1 ± 2.5	0.50 ± 0.06	61.1 ± 20.0

The next series of inhibitors included hydrophobic groups containing a biphenyl motif, starting with each biphenyl regioisomer—*ortho*, *meta*, and *para*—without further modifications. These designs were partially inspired by a discovery by the Griffin group^16^ of a very potent inhibitor known as EB-1-155 (*k*_inact_/*K*_I_ = 1508 × 10^3^ M^−1^ min^−1^)^26^ bearing dansyl as the hydrophobic group. The high efficiency of the dansyl inhibitor gives precedence to the idea of having large aromatic structures in this position (Table 2).

**Table 2:** Kinetic data for inhibitors with biphenyl hydrophobic groups

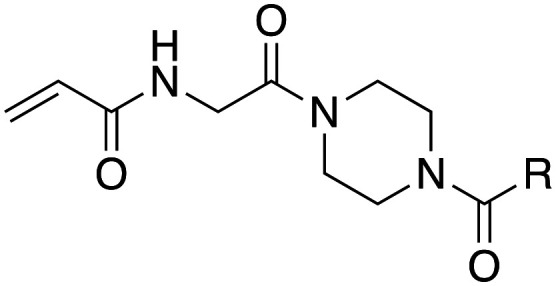				
Compound	R	*K*_I_ (μM)	*k*_inact_ (min^−1^)	*k*_inact_/*K*_I_ (× 10^3^ M^−1^ min^−1^)
7a	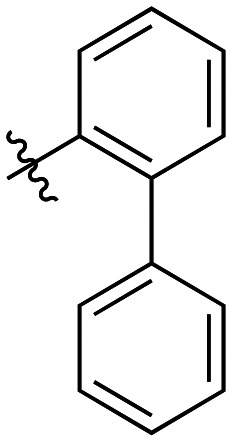	3.2 ± 1.0	0.58 ± 0.09	178.0 ± 68.6
7b	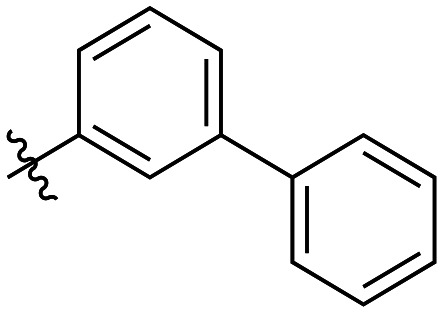	5.3 ± 1.4	0.71 ± 0.08	133.9 ± 38.4
7c	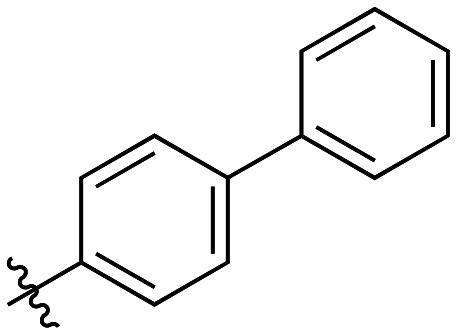	41.1 ± 32.2	0.71 ± 0.31	17.2 ± 15.5

For these three regioisomers, the inhibition efficiency increased as the angle of projection of the pendant phenyl ring decreased, with the *ortho* biphenyl inhibitor (7a) having similar efficiency to the cyclohexyl (6d), and much improved efficiency over the mono-phenyl inhibitor (6f). Interestingly, though the first hydrophobic ring appears to be limited to the size of a six-membered ring, extension from that ring can improve inhibition, as is seen by the relatively high *k*_inact_/*K*_I_ ratios of the *ortho* and *meta* inhibitors compared to the phenyl inhibitor (6f).

Additional biphenyl inhibitors were therefore prepared, introducing methyl substituents at different positions of the distal phenyl ring as well as bridging atoms between the two rings. Both the methyl additions and bridging atoms were chosen to probe for additional hydrophobic interactions. Incorporation of bridging atoms also sought to determine if additional freedom of rotation between the rings would be beneficial and if the presence of heteroatoms within the D-site could provide additional opportunities for binding.

**Scheme 3:**
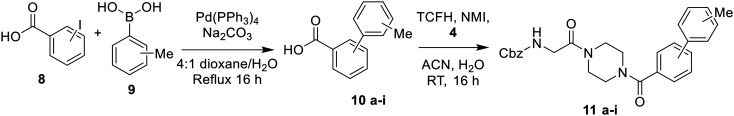
Synthesis of methylbiphenyl intermediates.

The synthesis of the methyl-substituted inhibitors followed Scheme 3. Methyl-substituted biphenyl carboxylic acids were obtained from Suzuki couplings between the respective iodobenzoic acids and tolylboronic acids (Scheme 3). The resulting methyl-substituted hydrophobic groups were coupled to intermediate 4 as was done before.

**Scheme 4:**
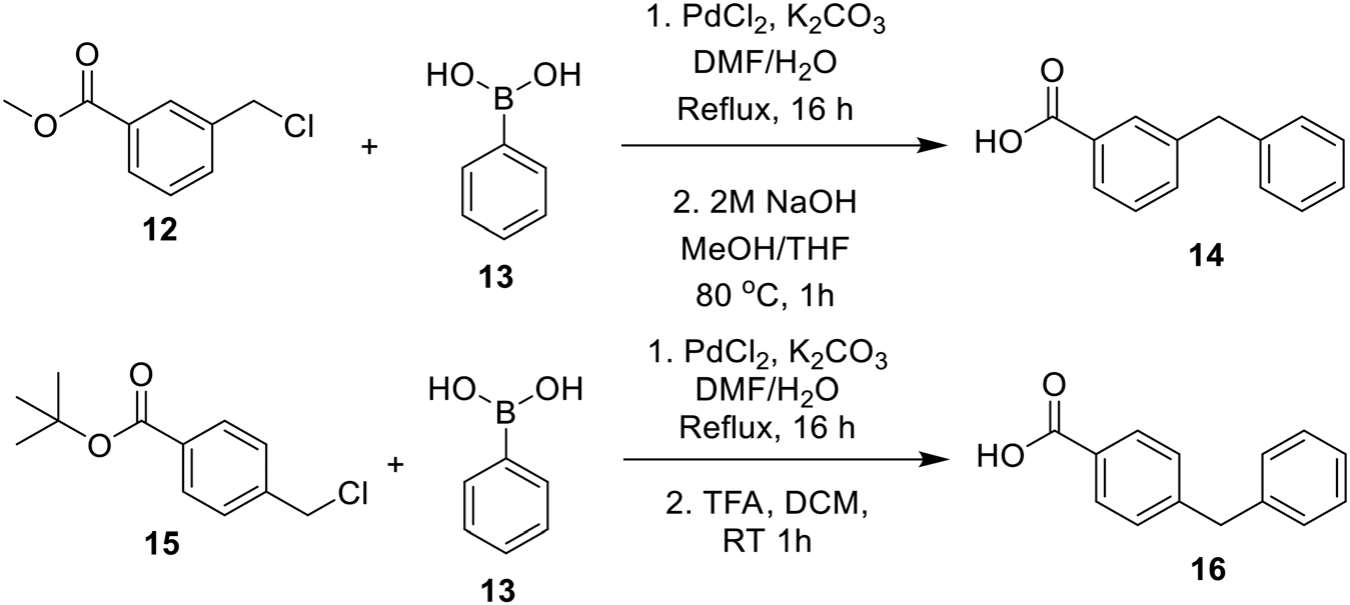
Synthesis of bridged biphenyl carboxylic acids.

**Scheme 5:**
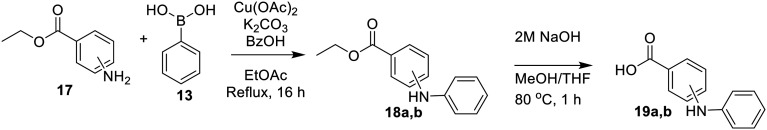
Synthesis of phenylaminobenzoic acids.

Bridged biphenyl carboxylic acids were obtained from a modified Suzuki coupling between the respective chloromethyl benzoate esters and boronic acid, followed by ester deprotection (Scheme 4). The remaining phenylaminobenzoic acids were synthesized from ethyl aminobenzoates and phenylboronic acid *via* Chan–Lam coupling. The resulting ethyl-protected phenylaminobenzoates were deprotected with aqueous NaOH to give the phenylaminobenzoic acids (Scheme 5). All the remaining bridged biphenyl precursors were purchased from chemical suppliers.

**Scheme 6:**
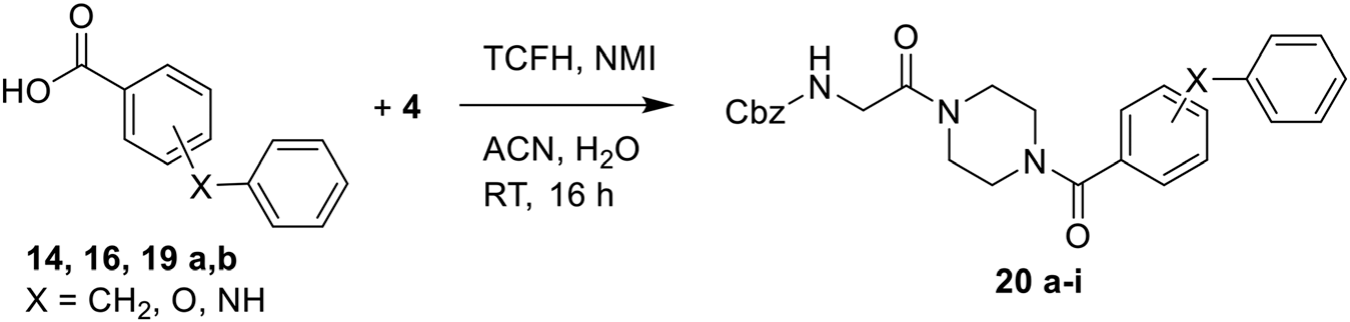
Synthesis of bridged biphenyl intermediates.

After synthesis of the bridged biphenyl carboxylic acids, they were coupled with intermediate 4 (Scheme 6) and then reacted following Scheme 2 to obtain the final inhibitors.

As shown in Table 3, the addition of methyl substituents to the distal phenyl rings was beneficial at some positions and detrimental at others. Compound 21d was the most efficient inhibitor of this series (*k*_inact_/*K*_I_ = 636 × 10^3^ M^−1^ min^−1^) and was the only inhibitor without an adamantyl group shown herein to approach the efficiency of EB-2-16. This result was unexpected, as 21d has the same *meta*-biphenyl backbone as 7b, which was less favoured than the unmodified *ortho* biphenyl inhibitor, 7a. Methyl additions to the *ortho*-biphenyl inhibitor only led to a slight improvement in efficiency when introduced at the 3′ position, and offered little overall improvement of the poor efficiency shown by the *para*-biphenyl scaffold.

**Table 3:** Kinetic data for inhibitors with methyl-biphenyl hydrophobic groups

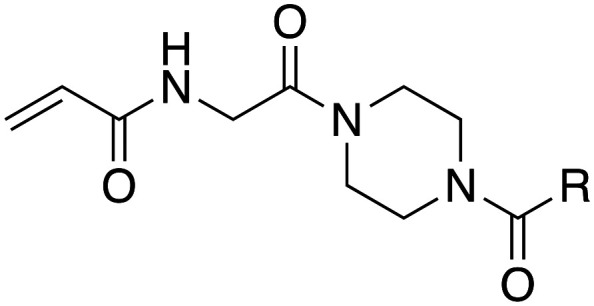				
Compound	R	*K*_I_ (μM)	*k*_inact_ (min^−1^)	*k*_inact_/*K*_I_ (× 10^3^ M^−1^ min^−1^)
21a	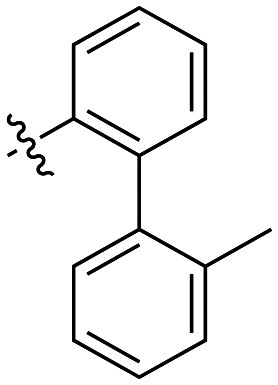	4.8 ± 1.0	0.57 ± 0.05	118 ± 27
21b	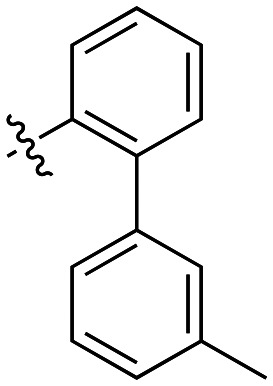	2.6 ± 0.7	0.52 ± 0.05	205 ± 60
21c	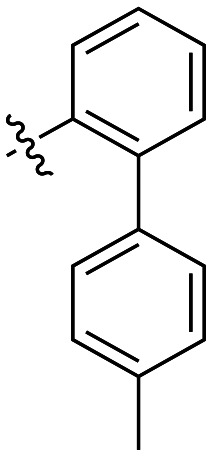	8.3 ± 2.1	0.48 ± 0.06	58.5 ± 16.2
21d	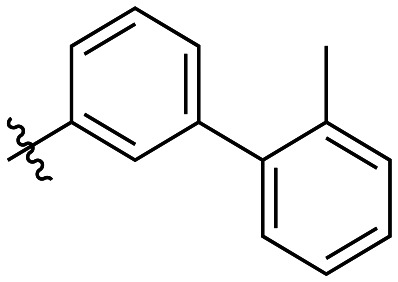	1.1 ± 0.1	0.72 ± 0.02	636 ± 58
21e	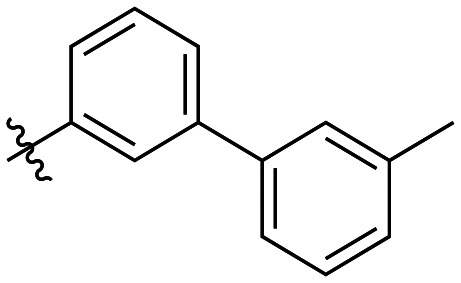	4.4 ± 2.8	0.62 ± 0.16	141 ± 98
21f	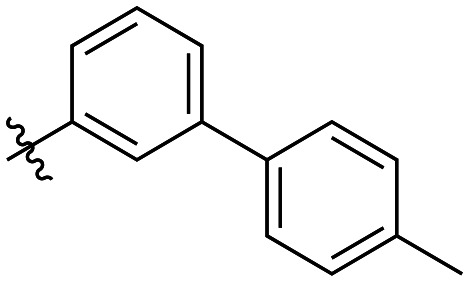	2.3 ± 0.6	0.55 ± 0.05	241 ± 74
21g	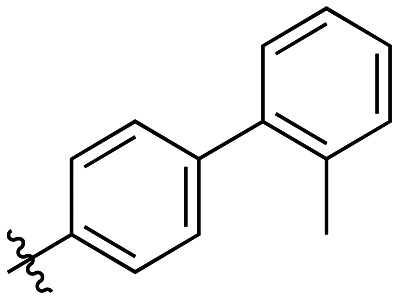	54.1 ± 14.6	1.30 ± 0.18	24.1 ± 7.3
21h	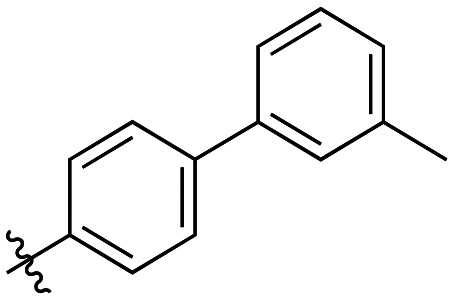	38.9 ± 17.7	0.89 ± 0.21	22.8 ± 11.7
21i	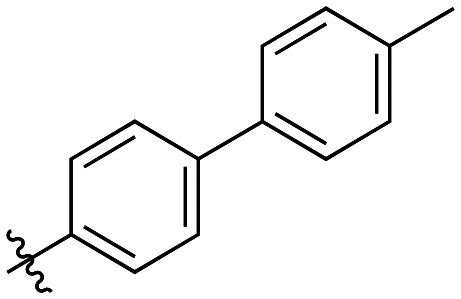	INS	INS	INS

Generally, incorporation of a bridging atom lowered inhibition efficiency, relative to the corresponding parent biphenyl compounds (22a–i, Table 4). This finding suggests either the extra freedom of rotation or increased length of the molecule (or a combination of the two) is not beneficial for binding. In most cases, the identity of the bridging group had a modest effect, with –O– and –NH– slightly increasing efficiency at the 2 position, –NH– decreasing efficiency at the 3 position, and both having little effect at the 4 position. These results provide some evidence that there are limited significant dipole interactions that can be exploited with heteroatoms at these positions, but does not necessarily rule out their potential with other hydrophobic motifs.

**Table 4:** Kinetic data for inhibitors with bridged-biphenyl hydrophobic groups

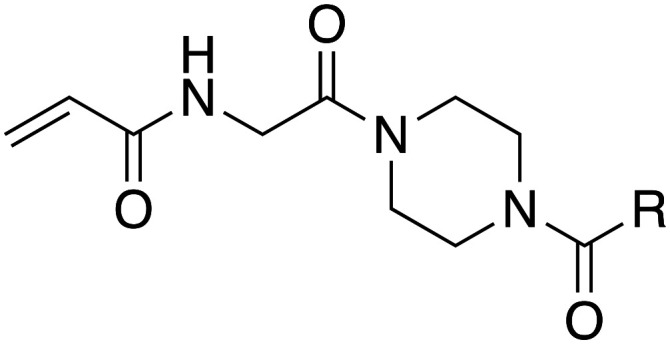				
Compound	R	*K*_I_ (μM)	*k*_inact_ (min^−1^)	*k*_inact_/*K*_I_ (× 10^3^ M^−1^ min^−1^)
22a	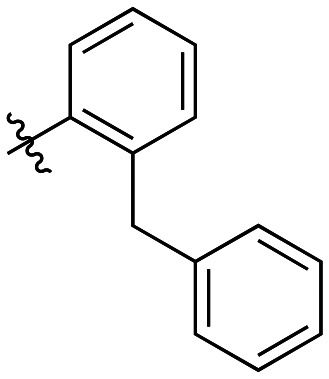	14.2 ± 3.3	0.87 ± 0.10	61.3 ± 15.9
22b	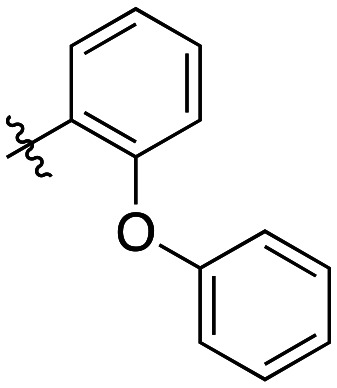	9.1 ± 0.8	0.86 ± 0.03	94.8 ± 9.0
22c	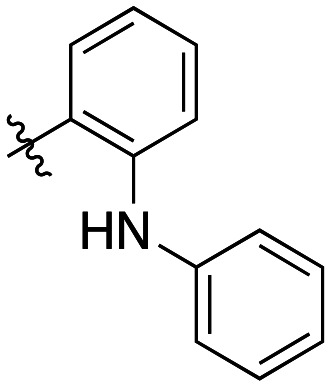	6.4 ± 0.7	0.72 ± 0.04	112 ± 14
22d	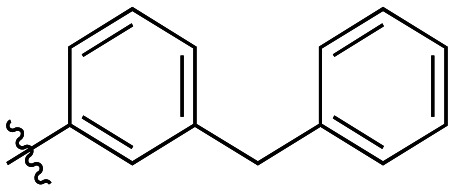	8.8 ± 2.7	1.01 ± 0.13	114 ± 38
22e	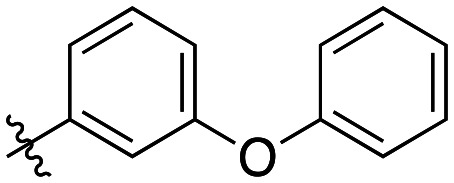	11.9 ± 4.0	1.34 ± 0.20	112 ± 41
22f	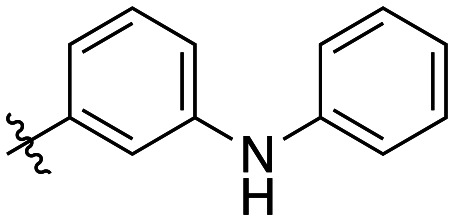	31.7 ± 8.6	0.99 ± 0.17	31.2 ± 10.0
22g	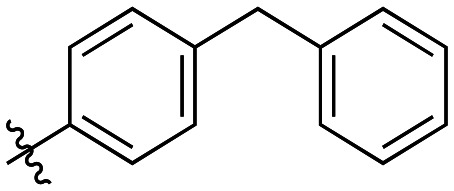	58.1 ± 9.4	1.52 ± 0.15	26.1 ± 4.9
22h	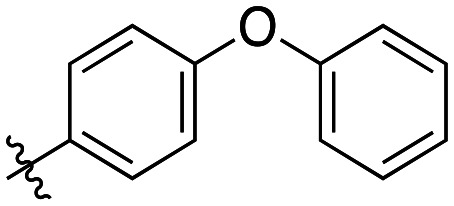	INS.	INS.	INS.
22i	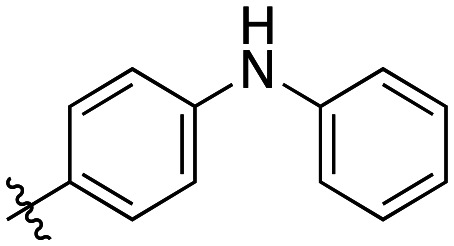	92.8 ± 43.3	1.53 ± 0.49	16.5 ± 9.4

The final series of inhibitors maintained the adamantyl group of EB-2-16, but introduced substitutions on the ring, including the halogens and a phenyl group, to investigate any steric and electronic effects of substituents on the adamantane ring. An adamantyl sulfonamide was also synthesized to determine how alteration of the bond angle, from carboxamide to sulfonamide, would affect binding and inhibitory efficiency.

**Scheme 7:**
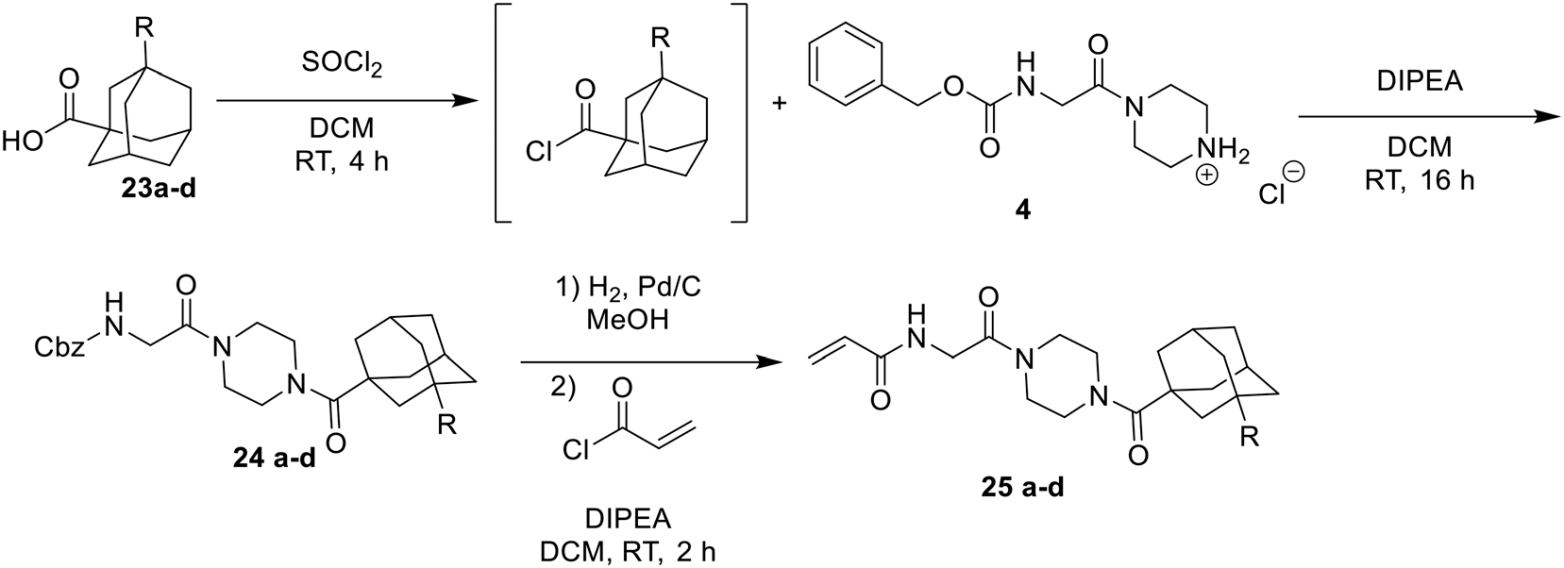
Synthesis of the substituted adamantane inhibitors.

After unsuccessful attempts to couple adamantane carboxylic acids according to Scheme 2, it was found that these inhibitors could only be synthesized from the adamantane acid chloride (Scheme 7). After the *in situ* formation of the acid chloride from the adamantane carboxylic acid, the same procedure was followed as shown in Scheme 2.

**Scheme 8:**
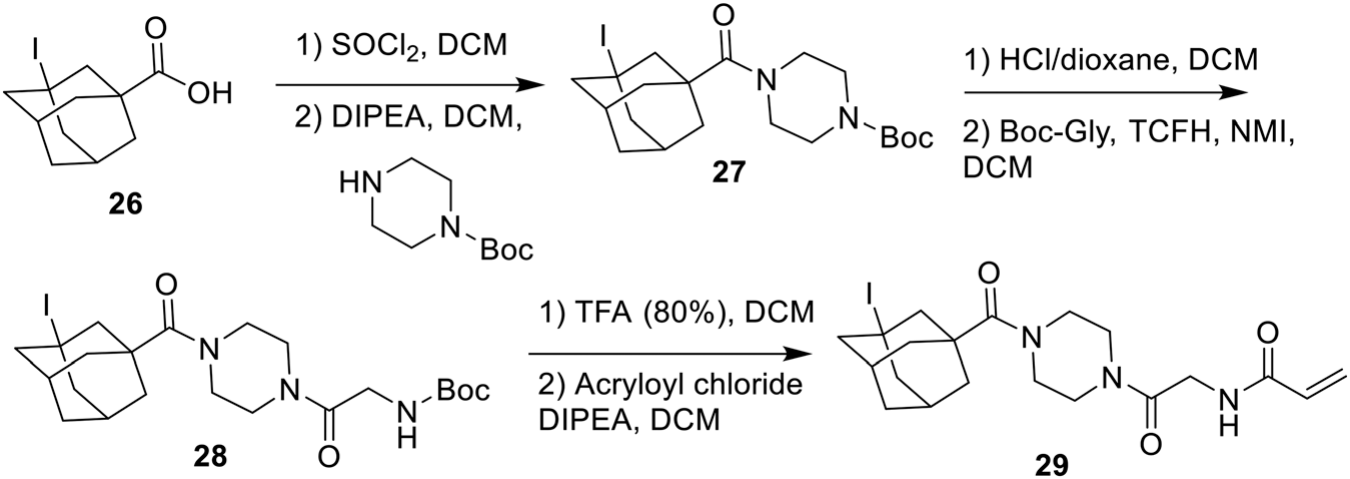
Synthesis of the iodo-adamantane inhibitor (29) following a stepwise approach.

The only inhibitor in this series that could not be accessed *via* this synthetic pathway was the iodo-adamantyl inhibitor (29). Iodine is known to act as a poison for palladium catalysts, suggesting that Cbz deprotection would not be facile synthetically.^29^ The iodo compound was therefore made following a stepwise procedure similar to that of Scheme 7, but using acid-labile protecting groups (Scheme 8).

**Scheme 9:**
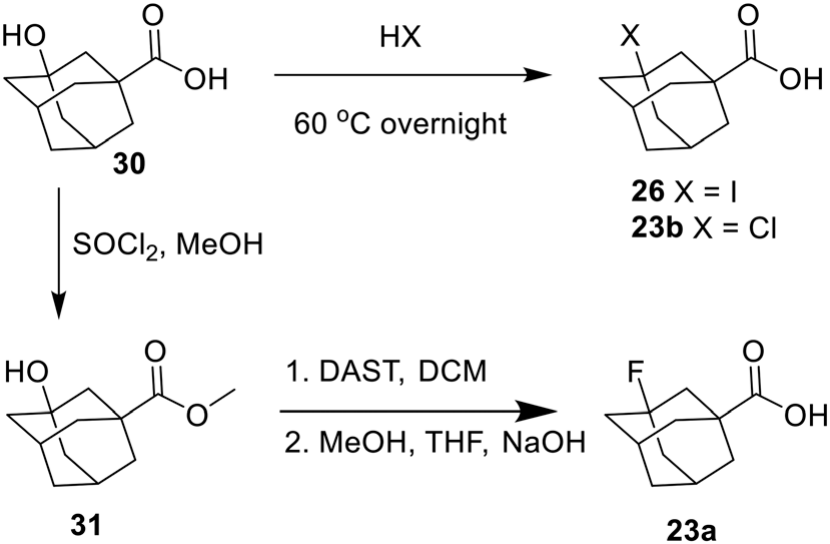
Synthesis of the I, Cl, and F adamantane carboxylic acids.

The halogenated adamantyl carboxylic acids had to be synthesized before incorporating them into the inhibitor. The I, Cl, and F inhibitors were all synthesized from a 3-hydroxyadamantane-1-carboxylic acid starting material, substituting the hydroxy for each halogen as shown in Scheme 9.^30,31^

**Scheme 10:**
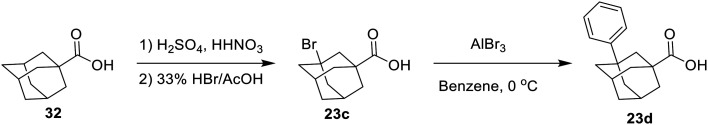
Synthesis of the bromo and phenyl adamantane carboxylic acids.

3-Bromoadamantanecarboxylic acid was synthesized *via* a two-step *in situ* reaction between 1-adamantanecarboxylic acid and a mixture of concentrated H_2_SO_4_/HNO_3_ which selectively oxidized the tertiary 3-position of the adamantyl group to an alcohol. Next, HBr/AcOH was added to the mixture, replacing the tertiary alcohol with bromide, presumably *via* S_N_1 substitution.^32^ Then, 3-phenyladamantane-1-carboxylic acid was made *via* Friedel-Crafts arylation with benzene, catalyzed by AlBr_3_ (Scheme 10).^33^

**Fig. 3:**
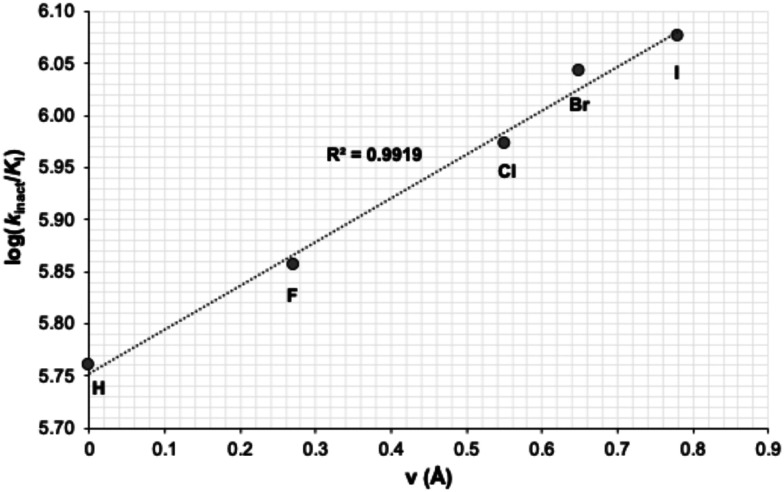
The linear relationship between log(*k*_inact_/*K*_I_) (in M^−1^ min^−1^) and the size index (*v*)^36^ of the halide substituent.

This series gave the best inhibitory activities of all compounds tested; as shown in Table 5, all these substituents resulted in better inhibition than the parent compound (R = H). We also observed a clear trend whereby the efficiency of inhibition (as reflected in the *k*_inact_/*K*_I_ ratio) increases with the size of the halide substituent. It should be noted that this trend is in contrast to the apparent efficiency reflected in the IC_50_ values reported in a recent patent, where the values of 54 nM, 58 nM and 119 nM were measured for the fluoro, chloro and bromo derivatives, respectively.^34^ This apparent discrepancy may be due to the IC_50_ values being measured by a different assay, with a different source of enzyme, and without taking time-dependence into account.^35^ It is also worth noting that the inhibitor bearing the largest substituent of the series, the phenyl group (25d), showed worse inhibitory efficiency than the bromo (25c) and iodo (29) derivatives. Taken together, we hypothesize that a substituent presenting a certain volume of diffuse electron density appears to be best suited for binding, over a larger volume such as phenyl or a smaller dense one such as fluoro. This size dependency was examined further by plotting the log(*k*_inact_/*K*_I_) values of the halide inhibitors against the relative size of the halide substituent. As shown in Fig. 3, this correlation illustrated that larger halogens result in more efficient inhibition, according to a linear free energy relationship with respect to their size parameter (*v*).^36^

**Table 5:** Kinetic data for inhibitors with substituted adamantane hydrophobic groups

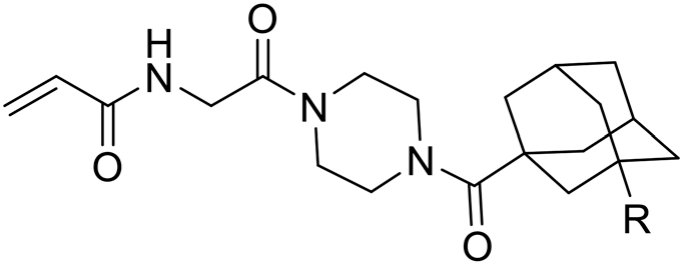				
Compound	R	*K*_I_ (μM)	*k*_inact_ (min^−1^)	*k*_inact_/*K*_I_ (× 10^3^ M^−1^ min^−1^)
EB-2-16	H	2.43 ± 0.19	1.40 ± 0.05	576 ± 50
25a	F	3.15 ± 0.33	2.26 ± 0.16	718 ± 91
25b	Cl	1.79 ± 0.17	1.69 ± 0.09	941 ± 101
25c	Br	0.65 ± 0.08	0.71 ± 0.05	1104 ± 162
29	I	1.40 ± 0.12	1.67 ± 0.07	1195 ± 112
25d	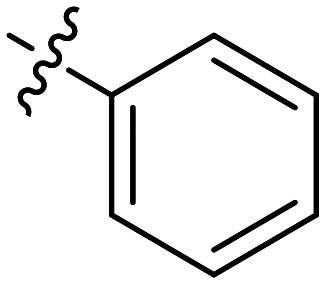	0.69 ± 0.07	0.67 ± 0.03	972 ± 103

**Scheme 11:**
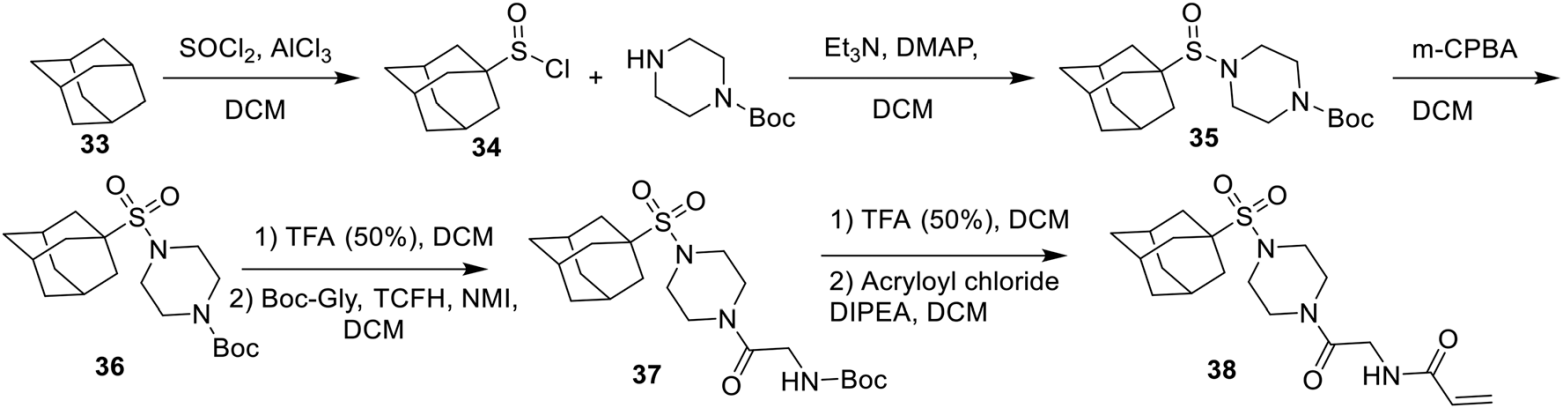
Synthesis of the adamantane sulfonamide inhibitor.

Finally, an adamantane sulfonamide derivative was synthesized to test the importance of the bond angle of the parent amide inhibitor. Originally, we envisioned proceeding *via* a coupling reaction between the sulfonic acid and the scaffold amine (4), but all attempts to perform the coupling between the hindered sulfonyl chloride and the secondary amine failed. Therefore, the sulfonamide derivative (38) was synthesised by way of the sulfinamide intermediate (35), which was prepared by coupling the less hindered sulfinyl chloride (34) with the secondary amine, followed by oxidation to the sulfonamide using *m*-CBPA (Scheme 11).^37^

Evaluation of the sulfonamide inhibitor (38) provided a *k*_inact_ of (0.97 ± 0.14) min^−1^ and *K*_I_ of (7.56 ± 1.87) μM for a *k*_inact_/*K*_I_ ratio of (129 ± 36) × 10^3^ M^−1^ min^−1^. Compared to other derivatives, 38 proved to be a much less efficient inhibitor, due to its substantially higher *K*_I_ value. This suggests the smaller C–S–N bond angle of the sulfonamide linkage cannot be accommodated in the binding site as easily as the C–C–N bond angle of amide linkage.

Once the efficiency of the compounds had been determined, the pharmacokinetic properties of the best inhibitors were tested. We have shown previously that EB-2-16 shows excellent bidirectional diffusion across the membrane of MDCK-MDR1 cells, and it is not susceptible to P-glycoprotein-mediated efflux.^28^ Considering the similarity of the adamantyl derivatives studied herein, and the structural requirements for P-glycoprotein affinity,^38^ all of them would be expected to show similarly excellent permeability properties. All the halide derivatives, the phenyl derivative (25d), and the parent EB-2-16 were evaluated (at Pharmaron, Inc.) in a parallel artificial membrane permeability assay (PAMPA). This assay is a measure of the compound's ability to pass through a lipid membrane, such as a cell membrane. In the case of the PAMPA testing, a lower −log *P*_e_ value indicates a more membrane permeable compound. All the tested compounds, except for the fluoro derivative, have −log *P*_e_ values less than 6 (*i.e. P*_e_ >10^−6^ cm s^−1^), which may be considered excellent permeability. The phenyl substituted adamantane (25d) was the most cell permeable with a −log *P*_e_ value of 4.88. The permeabilities of all the other derivatives were similar to that of the parent inhibitor EB-2-16, as expected (Table 6).

**Table 6:** PAMPA results for the substituted adamantane derivatives

Compound	−log[*P*_e_ (cm s^−1^)]
EB-2-16	5.81
25a	6.45
25b	5.85
25c	5.66
29	5.28
25d	4.88

Next, the stability of the compounds in human hepatocytes was studied (at Pharmaron, Inc.). Mader *et al.* reported that the parent compound EB-2-16 has a half-life of over 120 minutes in human hepatocytes.^27^ In order to be able to make a direct comparison with this reference, we also evaluated the stability of our inhibitors in human hepatocytes (Table 7). We reasoned that any compound that may be pharmacologically useful should be at least as stable as the parent compound EB-2-16. Two of the most potent compounds, the iodo (29) and the bromo (25c) were first incubated in phosphate buffer (0.1 M, pH = 8.0), and were found to degrade after 24 hours, even with no hepatocytes present. This instability was also reflected in a 20 minute half-life in the presence of hepatocytes for the iodo compound (29). The phenyl derivative (25d) was slightly more stable, with a half-life in hepatocytes of around 47 minutes, but was still not as stable as the parent compound. The chloro derivative (25b), on the other hand, has a half-life of 200 minutes in human hepatocytes, and is at least as stable as the parent compound. Therefore, despite the bromo (25c) and iodo (29) derivatives being more efficient and having slightly better membrane permeability, the chloro compound (25b) appears to be the most suitable for *in vivo* application. It is important to note that this chloroadamantyl inhibitor, as well as inhibitors 25a, c and d, were also divulged in a more recent patent by Griffin *et al.*^34^ This patent revealed that inhibitor 25b has a low IC_50_ (58 nM), as measured in an assay monitoring the incorporation of biotinylated cadaverine (0.25 mM) into immobilized DMC using rhTG2; however, that inhibitor was not subjected to further evaluation. In the current study, our kinetic evaluations confirm its potency, and our study of its membrane permeability and hepatocyte stability confirm its suitability for future applications.

**Table 7:** The stability of the substituted adamantane derivatives in human hepatocytes

Compound	*In vitro t*_1/2_ (min)	*In vitro* CL_int_ (μL min^−1^/10^6^ cells)
EB-2-16	>120 (ref. 27)	
25b	200	6.91
25d	47.2	29.3
29	20.0	69.2

## Conclusions

We present an extensive SAR study of the hydrophobic portion of the EB-2-16 scaffold. This study replaced the adamantane group of the parent compound with a range of cycloalkyl, biphenyl, bridged biphenyl, substituted biphenyl, and substituted adamantyl groups. Of the 33 compounds studied herein, 4 were previously divulged in a recent patent^34^ and 29 are novel compounds. Using a continuous assay, each of these compounds was rigorously evaluated to determine their kinetic parameters *k*_inact_ and *K*_I_, for the first time*.* While most of the cycloalkyl and biphenyl modifications resulted in worse inhibition, it was found that substituting the adamantyl group of the parent compound gave better inhibition. The iodo (29), bromo (25c), and phenyl (25d) adamantane derivatives gave significantly increased inhibition efficiencies and had good membrane permeability; however, these compounds had much worse stability than the parent compound. On the other hand, the chloro derivative 25b also showed improved inhibition efficiency, increased stability and similar permeability relative to the parent compound. Based on these results, the chloroadamantyl inhibitor 25b appears to be the most suitable for future studies, and potentially for *in vivo* applications.

## Materials and methods

### Synthesis

#### General remarks

All reagents and solvents used for the synthesis of chemical intermediates and final inhibitors were purchased from chemical supply companies including Sigma Aldrich, Fisher Scientific, Combi-Blocks, and Oakwood Chemical. Upon receipt, they were used without further purification. Anhydrous solvents were obtained using a Phoenix Solvent Dispensing System (JC Meyer Solvent Systems) with neutral alumina-packed columns. Thin layer chromatography was performed using EMD aluminum-backed silica 60 F254-coated plates and were visualized using either UV-light (254 nm), KMnO_4_ or ninhydrin stain. ^1^H NMR, ^13^C NMR, and ^19^F NMR were obtained using Bruker Avance II 400 and Bruker Avance III HD 600 spectrometers. Chemical shifts were reported in parts per million (PPM) with reference to the deuterated solvent peak (CDCl_3_: ^1^H = 7.26 ppm, ^13^C = 77.0 ppm; D_2_O: ^1^H = 4.79 ppm, ^13^C = spectrometer internal standard used only). HRMS accurate masses of novel molecules were obtained by the John L. Holmes Mass Spectrometry Facility using a Waters Synapt G1 electrospray ionization (ESI) quadrupole time-of-flight (QTOF) mass spectrometer. Compounds were purified by manual, normal-phase silica columns with 230–400 mesh silica gel and Biotage Selekt automated flash chromatography system with normal-phase Biotage Sfar 60 μM silica columns (or reverse-phase Biotage Sfar C18 Duo 100 Å 30 μm columns, when specified). Purity of the final inhibitors was evaluated *via* HPLC using a Gilson 322-H2 pump, a Gilson 159 UV-vis detector at 254 nm, and a Luna 5 μm C18(2) 100 Å 150 × 4.6 mm LC column.

##### General procedure A: synthesis of methyl-substituted biphenyl carboxylic acids

To a round bottom flask equipped with a magnetic stir bar was added the iodobenzoic acid (1.0 eq.), tolylboronic acid (1.5 eq.), and Na_2_CO_3_ (3.0 eq.). A 4 : 1 mixture of dioxane/H_2_O was then added to the flask followed by Pd(PPh_3_)_4_ (0.05 eq.). The reaction mixture was stirred at reflux overnight (approx. 16 h). Upon completion, the mixture was diluted with equal volumes of EtOAc and H_2_O until the organic and aqueous phases separate. This mixture was then filtered through celite and transferred to a separatory funnel where it was acidified to pH = 1–2 using conc. HCl. and extracted with EtOAc (×3). The combined organic phases were dried over MgSO_4_, filtered and evaporated under vacuum. The resulting crude mixture was purified *via* column chromatography (5% MeOH/DCM on silica).

##### General procedure B: synthesis of Cbz–Gly–PZ-hydrophobic intermediates

To a round bottom flask equipped with a magnetic stir bar was added the carboxylic acid (1.0 eq.) and TCFH (1.1 eq.), then solubilized in 10 mL ACN. NMI (3.5 eq.) was then added, and the mixture was left to stir for 15 minutes at R.T. Benzyl *N*-[2-oxo-2-(piperazin-1-yl)ethyl]carbamate HCl (4) (1.2 eq.) was then added to the flask with enough drops of H_2_O to dissolve the salt. The reaction was allowed to stir overnight (approx. 16 h) at R.T. under open atmosphere. Upon completion, the reaction mixture was diluted with 50 mL EtOAc and transferred to a separatory funnel where it was washed with 10% AcOH (×3), brine (×3), and 2 M NaOH (×3). The organic phase was dried over MgSO_4_, filtered, and the solvent was evaporated under vacuum. The resulting crude product was purified *via* column chromatography.

##### General procedure C: synthesis of Cbz–Gly–PZ-substituted adamantane intermediates

The product was obtained by adding the acid (1.0 eq.) to a round bottom flask charged with a stir bar and dissolving in DCM. Thionyl chloride (2.0 eq.) was added dropwise slowly and left to stir for 4 hours at room temperature in open atmosphere. The solvent was evaporated under vacuum, then co-evaporated with DCM (×3). This powder was dissolved directly in DCM. To this mixture was added DIPEA (2.0 eq.) and benzyl *N*-[2-oxo-2-(piperazin-1-yl)ethyl]carbamate HCl (4) (1.2 eq.), then left to stir overnight (approx. 16 h). The product was worked up using either work up A or B. The crude product was purified *via* flash silica column chromatography (if required).

Work up A: the solvent was removed and the residue was dissolved in EtOAc, and washed with 2 M NaOH (×3) and brine (×3). The organic phase was then dried over MgSO_4_, filtered, and evaporated under vacuum.

Work up B: the solvent was removed and the residue was dissolved in EtOAc, and washed with 10% AcOH (×3), brine (×2), saturated NaHCO_3_ (×3), and brine (×3). The organic phase was then dried over MgSO_4_, filtered, and evaporated under vacuum.

##### General procedure D: synthesis of final acrylamide inhibitors

A round bottom flask equipped with a magnetic stir bar was purged with N_2_ and sealed with a rubber stopper. To this flask was added the Cbz-protected Gly–PZ-Hyd intermediate (1.0 eq.), palladium on carbon (10% w/w), and dry MeOH. The atmosphere of the flask replaced with H_2_ with a balloon and the reaction was stirred until deemed complete by TLC (5% MeOH/DCM) and ninhydrin staining (between 2 and 16 h). Upon completion, the reaction mixture was filtered through celite, and the solvent was evaporated under vacuum. To this flask containing the free-amine intermediate was added a magnetic stir bar and dry DCM. DIPEA (3.0 eq.) was then added to the mixture, followed by acryloyl chloride (1.1 eq.) which was added dropwise slowly, either at room temperature or at 0 °C (the equivalencies were determined based on the amount of Cbz-protected starting material used). The reaction was allowed to stir for 4 h at R.T. or allowed to warm to room temperature, then washed using work up B or C. The product from the work up was washed with hexanes (×3) and purified *via* column chromatography.

Work up B: the solvent was removed and the product was dissolved in EtOAc, and washed with 10% AcOH (×3), brine (×2), saturated NaHCO_3_ (×3), and brine (×3). The organic phase was then dried over MgSO_4_, filtered, and evaporated under vacuum.

Work up C: the reaction was diluted with diluted with EtOAc and transferred to a separatory funnel where it was washed with 10% AcOH (×3) and brine (×3). The organic phase was dried over MgSO_4_, filtered, and the solvent was evaporated under vacuum.

#### *Tert*-butyl 4-(2-(((benzyloxy)carbonyl)amino)acetyl)piperazine-1-carboxylate (3)

To a round bottom flask equipped with a magnetic stir bar and 100 mL ACN was added *N*-benzyloxycarbonylglycine (5 g, 24.0 mmol, 1.0 eq.), TCFH (8.04 g, 28.7 mmol, 1.2 eq.), and NMI (3.81 mL, 48 mmol, 2.0 eq.), and was left to stir for 20 minutes. 1-Boc-piperazine (4.47 g, 24.0 mmol, 1.0 eq.) was added and the reaction mixture was stirred overnight (approx. 16 h) in open atmosphere at room temperature. Upon completion, the solvent was evaporated under vacuum to give a light-yellow oil, which was dissolved in 100 mL EtOAc and washed in a separatory funnel with 5% AcOH (×3), brine (×3), and sat. NaHCO_3_ (×3). The organic phase was then dried over MgSO_4_, filtered, and evaporated under vacuum to give a white an off-white foam. This crude product was purified *via* column chromatography (3% MeOH/DCM) to give the pure product as a white foam (7.1 g, 78%).

^1^H NMR (600 MHz, CDCl_3_) *δ* 7.38–7.28 (m, 5H), 5.77 (br s, 1H), 5.12 (s, 2H), 4.06–3.94 (m, 2H), 3.64–3.56 (m, 2H), 3.51–3.40 (m, 4H), 3.40–3.28 (m, 2H), 1.47 (s, 9H). ^13^C NMR (150 MHz, CDCl_3_) *δ* 166.6, 156.2, 154.4, 136.3, 128.5, 136.3, 128.5, 128.1, 128.0, 80.5, 67.0, 44.2, 42.7, 41.8, 28.3.

HRMS (ESI-QTOF) *m*/*z* (M + Na)^+^ calcd for C_19_H_27_N_3_O_5_Na: 400.1848, found: 400.1832.

#### Benzyl[2-oxo-2-(piperazin-1-yl)ethyl]carbamate HCl (4)

To a round bottom flask equipped with a stir bar was added compound 3 (5 g, 13.3 mmol, 1.0 eq.), which was then solubilized in DCM (70 mL) and 4 M HCl/dioxane solution (70 mL). The reaction mixture was stirred for 4 hours in open atmosphere at room temperature until deemed complete by TLC (5% MeOH/DCM and ninhydrin stain). During this time, white precipitate formed. Upon completion, the solvent was evaporated under vacuum. The resulting white powder to washed with cold ether (×3) and filtered to give the pure product as a white powder (3.13 g, 75%).

^1^H NMR (600 MHz, D_2_O) *δ* 7.50–7.39 (m, 5H), 5.19–5.12 (m, 2H), 4.10 (s, 2H), 3.88–3.69 (m, 4H), 3.39–3.10 (m, 4H). ^13^C NMR (150 MHz, D_2_O) *δ* 169.7, 158.6, 136.3, 128.8, 128.5, 127.7, 67.3, 42.9, 42.8, 41.9, 41.4, 39.8.

HRMS (ESI-QTOF) *m*/*z* (M + H)^+^ calcd for C_14_H_19_N_3_O_3_H: 278.1505, Found: 278.1555.

#### Benzyl[2-(4-cyclopropanecarbonylpiperazin-1-yl)-2-oxoethyl]carbamate (5a)

The product was obtained following *general procedure B* using cyclopropanecarboxylic acid (70 μL, 0.884 mmol), TCFH (272 mg, 0.972 mmol), NMI (247 μL, 3.094 mmol), and compound 4 (333 mg, 1.156 mmol) in a solution of ACN (10 mL) and H_2_O (approx. 0.5 mL). The product was isolated following silica column chromatography (5% MeOH/DCM) and solvent evaporation to give a colourless oil. A white powder was obtained by dissolving the oil in a minimal amount of DCM and precipitating the product *via* hexane addition and solvent evaporation (171.0 mg, 56%).

^1^H NMR (600 MHz, CDCl_3_) *δ* 7.40–7.28 (m, 5H), 5.75 (br s, 1H), 5.13 (s, 2H), 4.08–3.97 (m, 2H), 3.79–3.56 (m, 6H), 3.53–3.30 (m, 2H), 1.75–1.67 (m, 1H), 1.04–1.00 (m, 2H), 0.86–0.76 (m, 2H). ^13^C NMR (150 MHz, CDCl_3_) *δ* 172.3, 156.2, 136.3, 128.6, 128.2, 128.1, 67.0, 45.1, 42.7, 42.0, 11.0, 7.8.

HRMS (ESI-QTOF) *m*/*z* (M + Na)^+^ calcd for C_18_H_23_N_3_O_4_Na: 368.1586, found: 368.1592.

#### Benzyl[2-(4-cyclobutanecarbonylpiperazin-1-yl)-2-oxoethyl]carbamate (5b)

The product was obtained following *general procedure B* using cyclobutanecarboxylic acid (84 μL, 0.884 mmol), TCFH (272 mg, 0.972 mmol), NMI (247 μL, 3.094 mmol), and compound 4 (333 mg, 1.156 mmol) in a solution of ACN (10 mL) and H_2_O (approx. 0.5 mL). The product was isolated following silica column chromatography (5% MeOH/DCM) and solvent evaporation to give a colourless oil. A white powder was obtained by dissolving the oil in a minimal amount of DCM and precipitating the product *via* hexane addition and solvent evaporation (267 mg, 84%).

^1^H NMR (600 MHz, CDCl_3_) *δ* 7.39–7.28 (m, 5H), 5.80–5.69 (m, 1H), 5.12 (s, 2H), 4.08–3.95 (m, 2H), 3.68–3.54 (m, 4H), 3.42–3.30 (m, 4H), 3.30–3.19 (m, 1H), 2.40–2.29 (m, 2H), 2.21–2.12 (m, 2H), 2.03–1.93 (m, 1H), 1.92–1.84 (m, 1H). ^13^C NMR (150 MHz, CDCl_3_) *δ* 173.4, 173.3, 166.8, 166.6, 156.2, 136.3, 128.5, 128.2, 128.0, 67.0, 44.7, 44.5, 44.2, 42.6, 42.1, 41.8, 41.4, 41.2, 37.1, 37.1, 25.0, 17.9.

HRMS (ESI-QTOF) *m*/*z* (M + Na)^+^ calcd for C_19_H_25_N_3_O_4_Na: 382.1743, found: 382.1778.

#### Benzyl[2-(4-cyclopentanecarbonylpiperazin-1-yl)-2-oxoethyl]carbamate (5c)

The product was obtained following *general procedure B* using cyclopentanecarboxylic acid (50 μL, 0.884 mmol), TCFH (272 mg, 0.972 mmol), NMI (247 μL, 3.094 mmol), and compound 4 (333 mg, 1.156 mmol) in a solution of ACN (10 mL) and H_2_O (approx. 0.5 mL). The product was isolated following silica column chromatography (5% MeOH/DCM) and solvent evaporation to give a colourless oil. A white powder was obtained by dissolving the oil in a minimal amount of DCM and precipitating the product *via* hexane addition and solvent evaporation (158 mg, 48%).

^1^H NMR (600 MHz, CDCl_3_) *δ* 7.39–7.28 (m, 5H), 5.83–5.70 (m, 1H), 5.13 (s, 2H), 4.08–3.96 (m, 2H), 3.70–3.58 (m, 4H), 3.58–3.47 (m, 2H), 3.44–3.35 (m, 2H), 2.92–2.83 (m, 1H), 1.86–1.78 (m, 4H), 1.78–1.70 (m, 2H), 1.62–1.54 (m, 2H). ^13^C NMR (150 MHz, CDCl_3_) *δ* 174.9, 174.7, 166.8, 166.6, 156.2, 136.3, 128.5, 128.1, 128.0, 67.0, 45.1, 45.0, 44.6, 44.2, 42.6, 42.1, 41.9, 41.5, 41.0, 30.1, 26.0.

HRMS (ESI-QTOF) *m*/*z* (M + Na)^+^ calcd for C_20_H_27_N_3_O_4_Na: 396.1899, found: 396.1877.

#### Benzyl[2-(4-cyclohexanecarbonylpiperazin-1-yl)-2-oxoethyl]carbamate (5d)

The product was obtained following *general procedure B* using cyclohexanecarboxylic acid (113 μL, 0.884 mmol), TCFH (272 mg, 0.972 mmol), NMI (247 μL, 3.094 mmol), and compound 4 (333 mg, 1.156 mmol) in a solution of ACN (10 mL) and H_2_O (approx. 0.5 mL). The product was isolated following silica column chromatography (5% MeOH/DCM) and solvent evaporation to give a colourless oil. A white powder was obtained by dissolving the oil in a minimal amount of DCM and precipitating the product *via* hexane addition and solvent evaporation (252.6 mg, 74%).

^1^H NMR (600 MHz, CDCl_3_) *δ* 7.40–7.29 (m, 5H), 5.82–5.66 (m, 1H), 5.13 (s, 2H), 4.08–3.95 (m, 2H), 3.71–3.29 (m, 8H), 2.49–2.39 (s, 1H), 1.87–1.75 (m, 2H), 1.75–1.64 (m, 3H), 1.56–1.48 (m, 2H), 1.33–1.19 (m, 3H). ^13^C NMR (150 MHz, CDCl_3_) *δ* 174.9, 166.8, 156.2, 136.3, 128.5, 128.2, 128.0, 67.0, 44.9, 44.3, 42.6, 42.2, 41.9, 41.2, 40.5, 20.3, 25.7, 25.7.

HRMS (ESI-QTOF) *m*/*z* (M + Na)^+^ calcd for C_21_H_29_N_3_O_4_Na: 410.2056, found: 410.2072.

#### Benzyl[2-(4-cycloheptanecarbonylpiperazin-1-yl)-2-oxoethyl]carbamate (5e)

The product was obtained following *general procedure B* using cycloheptanecarboxylic acid (60 μL, 0.442 mmol), TCFH (136 mg, 0.486 mmol), NMI (123 μL, 1.547 mmol), and compound 4 (140 mg, 0.503 mmol) in a solution of ACN (10 mL) and H_2_O (approx. 0.5 mL). The product was isolated following silica column chromatography (5% MeOH/DCM) and solvent evaporation to give a colourless oil. A white powder was obtained by dissolving the oil in a minimal amount of DCM and precipitating the product *via* hexane addition and solvent evaporation (138 mg, 78%).

^1^H NMR (600 MHz, CDCl_3_) *δ* 7.39–7.30 (m, 5H), 5.81–5.67 (m, 1H), 5.13 (s, 2H), 4.08–3.96 (m, 2H), 3.70–3.56 (m, 4H), 3.56–3.34 (m, 4H), 2.66–2.57 (m, 1H), 1.84–1.74 (m, 4H), 1.74–1.65 (m, 2H), 1.62–1.53 (m, 4H), 1.49–1.39 (m, 2H). ^13^C NMR (150 MHz, CDCl_3_) *δ* 176.1, 166.8, 156.2, 136.3, 128.5, 128.2, 128.1, 67.0, 45.1, 44.3, 42.6, 42.1, 41.5, 41.2, 31.2, 28.2, 26.6.

HRMS (ESI-QTOF) *m*/*z* (M + Na)^+^ calcd for C_22_H_31_N_3_O_4_Na: 424.2212, found: 424.2216.

#### Benzyl [2-(4-benzoylpiperazin-1-yl)-2-oxoethyl]carbamate (5f)

The product was obtained following *general procedure B* using benzoic acid (108 mg, 0.963 mmol), TCFH (297 mg, 1.06 mmol), NMI (269 μL, 3.37 mmol), and compound 4 (363 mg, 1.16 mmol) in a solution of ACN (10 mL) and H_2_O (approx. 0.5 mL). The product was isolated following silica column chromatography (5% MeOH/DCM) and solvent evaporation to give a colourless oil. A white powder was obtained by dissolving the oil in a minimal amount of DCM and precipitating the product *via* hexane addition and solvent evaporation (275 mg, 75%).

^1^H NMR (400 MHz, CDCl_3_) *δ* 7.48–7.38 (m, 5H), 7.38–7.29 (m, 5H), 5.75 (br s, 1H), 5.13 (s, 2H), 4.05 (s, 2H), 3.91–3.22 (m, 8H). ^13^C NMR (100 MHz, CDCl_3_) δ 170.7, 166.8, 156.2, 136.3, 134.9, 130.2, 128.7, 128.5, 128.2, 128.0, 127.1, 67.0, 47.3, 44.3, 42.6, 42.1.

HRMS (ESI-QTOF) *m*/*z* (M + Na)^+^ calcd for C_21_H_23_N_3_O_4_Na: 404.1586, found: 404.1591.

#### *N*-2-[4-[(Cyclopropanecarbonyl)-1-piperazinyl]-2-oxoethyl]propenamide (6a)

A round bottom flask equipped with a magnetic stir bar was purged of its air by vacuum and its atmosphere was replaced with N_2_ and sealed with a rubber stopper. To this flask was compound 5a (150 mg, 0.434 mmol, 1.0 eq.) and palladium on carbon (15 mg, 10% w/w). The atmosphere of the flask replaced with H_2_ with a balloon and the reaction was stirred until deemed complete by TLC (5% MeOH/DCM) and ninhydrin staining (approx. 2 h). Upon completion, the reaction mixture was filtered through celite, and the solvent was evaporated under vacuum. This intermediate was then dissolved in dry DCM (10 mL) under N_2_ atmosphere. To this solution was added acryloyl chloride (43 μL, 0.477 mmol, 1.1 eq.), and was left to stir for 20 minutes, during which the mixture became white and cloudy. DIPEA (227 μL, 1.3 mmol, 3.0 eq.) was then added to the solution, which was stirred for 1 h. Upon completion, the solvent was evaporated to give a golden oil, which was purified by silica column chromatography (3–6% MeOH/DCM manual gradient) to give the pure product as a colourless oil. A white powder was obtained by dissolving the oil in a minimal amount of DCM and precipitating the product *via* hexane addition and solvent evaporation (36.2 mg, 31%).

^1^H NMR (600 MHz, CDCl_3_) *δ* 6.68 (br s, 1H), 6.32 (dd, *J* = 17.0, 1.4 Hz, 1H), 6.20 (dd, *J* = 17.0, 10.3 Hz, 1H), 5.69 (dd, *J* = 10.2, 1.6 Hz, 1H), 4.19–4.15 (m, 2H), 3.79–3.61 (m, 6H), 3.56–3.40 (m, 2H), 1.75–1.70 (m, 1H), 1.04–1.01 (m, 2H), 0.84–0.80 (m, 2H). ^13^C NMR (150 MHz, CDCl_3_) δ 172.4, 166.7, 165.4, 130.3, 127.0, 45.1, 44.3, 42.0, 41.7, 41.3, 11.0, 7.8.

HRMS (ESI-QTOF) *m*/*z* (M + Na)^+^ calcd for C_13_H_19_N_3_O_3_Na: 288.1324, found: 288.1339.

#### *N*-2-[4-[(Cyclobutanecarbonyl)-1-piperazinyl]-2-oxoethyl]propenamide (6b)

A round bottom flask equipped with a magnetic stir bar was purged of its air by vacuum and its atmosphere was replaced with N_2_ and sealed with a rubber stopper. To this flask was added compound 5b (150 mg, 0.434 mmol, 1.0 eq.) and palladium on carbon (15 mg, 10% w/w). The atmosphere of the flask replaced with H_2_ with a balloon and the reaction was stirred until deemed complete by TLC (5% MeOH/DCM) and ninhydrin staining (approx. 2 h). Upon completion, the reaction mixture was filtered through celite, and the solvent was evaporated under vacuum. This intermediate was then dissolved in dry DCM (10 mL) under N_2_ atmosphere. To this solution was added Na_2_CO_3_ (138 mg, 1.3 mmol, 3.0 eq.) and acryloyl chloride (43 μL, 0.477 mmol, 1.1 eq.) which was added dropwise. The reaction mixture was left to stir for 2 h. Upon completion, the solvent was evaporated to give a golden oil, which was purified by silica column chromatography (3–5% MeOH/DCM manual gradient) to give the pure product as a colourless oil. A white powder was obtained by dissolving the oil in a minimal amount of DCM and precipitating the product *via* hexane addition and solvent evaporation (27.5 mg, 23%).

^1^H NMR (600 MHz, CDCl_3_) *δ* 6.72–6.59 (m, 1H), 6.31 (d, *J* = 17.0 Hz, 1H), 6.19 (dd, *J* = 17.0, 10.3 Hz, 1H), 5.69 (d, *J* = 10.3 Hz, 1H), 4.18–4.09 (m, 2H), 3.70–3.59 (m, 4H), 3.45–3.34 (m, 4H), 3.26 (quint, *J* = 8.5 Hz, 1H), 2.35 (quint, *J* = 9.6 Hz, 2H), 2.21–2.13 (m, 2H), 2.04–1.94 (m, 1H), 1.93–1.85 (m, 1H). ^13^C NMR (150 MHz, CDCl_3_) *δ* 173.4, 173.3, 166.8, 166.5, 165.4, 130.3, 127.0, 44.7, 44.6, 44.5, 44.3, 42.2, 41.9, 41.4, 41.3, 41.2, 37.1, 25.3, 18.0.

HRMS (ESI-QTOF) *m*/*z* (M + Na)^+^ calcd for C_14_H_21_N_3_O_3_Na: 302.1481, found: 302.1489.

#### *N*-2-[4-[(Cyclopentanecarbonyl)-1-piperazinyl]-2-oxoethyl]propenamide (6c)

The product was obtained following *general procedure D* using compound 5c (100 mg, 0.268 mmol) and Pd/C (20 mg) in dry MeOH and stirred for 2 h. Then dry DCM, acryloyl chloride (24 μL, 0.295 mmol), and DIPEA (140 μL, 0.804 mmol), added at room temperature. Work up C was used. The product was isolated following silica column chromatography (5% MeOH/DCM) to give a colourless oil. A white powder was obtained by dissolving the oil in a minimal amount of DCM and precipitating the product *via* hexane addition and solvent evaporation (31.2 mg, 40%).

^1^H NMR (600 MHz, CDCl_3_) *δ* 6.75–6.64 (m, 1H), 6.34 (dd, *J* = 17.0, 1.2 Hz, 1H), 6.22 (dd, *J* = 17.0, 10.3 Hz, 1H), 5.71 (d, *J* = 10.3 Hz, 1H), 4.22–4.16 (m, 2H), 3.74–3.55 (m, 6H), 3.50–3.42 (m, 2H), 2.91 (quint, *J* = 7.9 Hz, 1H), 1.90–1.81 (m, 4H), 1.80–1.72 (m, 2H), 1.66–1.59 (m, 2H). ^13^C NMR (150 MHz, CDCl_3_) *δ* 174.9, 169.4, 165.4, 130.3, 127.0, 44.3, 42.0, 41.3, 41.3, 41.1, 39.8, 39.2, 30.1, 26.0.

HRMS (ESI-QTOF) *m*/*z* (M + Na)^+^ calcd for C_15_H_23_N_3_O_3_Na: 316.1637, found: 316.1618.

#### *N*-2-[4-[(Cyclohexanecarbonyl)-1-piperazinyl]-2-oxoethyl]propenamide (6d)

The product was obtained following *general procedure D* compound 5d (100 mg, 0.258 mmol) and Pd/C (20 mg) in dry MeOH and stirred for 2 h. Then dry DCM, acryloyl chloride (23 μL, 0.284 mmol), and DIPEA (135 μL, 0.774 mmol), added at room temperature. Work up C was used. The product was isolated following silica column chromatography (5% MeOH/DCM) to give a colourless oil. A white powder was obtained by dissolving the oil in a minimal amount of DCM and precipitating the product *via* hexane addition and solvent evaporation (50.9 mg, 64%).

^1^H NMR (600 MHz, CDCl_3_) *δ* 6.73–6.60 (m, 1H), 6.32 (d, *J* = 17.1 Hz, 1H), 6.19 (dd, *J* = 17.0, 10.3 Hz, 1H), 5.69 (d, *J* = 10.3 Hz, 1H), 4.16 (m, 1H), 4.18–4.14 (m, 2H), 3.72–3.38 (m, 8H), 2.50–2.41 (m, 1H), 1.86–1.77 (m, 2H), 1.74–1.66 (m, 3H), 1.60–1.49 (m, 2H), 1.34–1.21 (m, 3H). ^13^C NMR (150 MHz, CDCl_3_) *δ* 174.9, 166.8, 165.4, 130.3, 127.0, 45.0, 44.9, 44.7, 44.3, 42.3, 42.0, 41.3, 40.5, 29.4, 25.8, 25.7.

HRMS (ESI-QTOF) *m*/*z* (M + Na)^+^ calcd for C_16_H_25_N_3_O_3_Na: 330.1794, found: 330.1784.

#### *N*-2-[4-[(Cycloheptanecarbonyl)-1-piperazinyl]-2-oxoethyl]propenamide (6e)

The product was obtained following *general procedure D* using compound 5e (100 mg, 0.249 mmol) and Pd/C (20 mg) in dry MeOH and stirred for 2 h. Then dry DCM, acryloyl chloride (22 μL, 0.274 mmol), and DIPEA (130 μL, 0.747 mmol), added at room temperature. Work up C was used. The product was isolated following silica column chromatography (5% MeOH/DCM) to give a colourless oil. A white powder was obtained by dissolving the oil in a minimal amount of DCM and precipitating the product *via* hexane addition and solvent evaporation (39.2 mg, 49%).

^1^H NMR (600 MHz, CDCl_3_) *δ* 6.73–6.61 (m, 1H), 6.32 (dd, *J* = 17.1, 1.3 Hz, 1H), 6.19 (dd, *J* = 17.0, 10.3 Hz, 1H), 5.69 (d, *J* = 10.4 Hz, 1H), 4.19–4.14 (m, 2H), 3.72–3.60 (m, 4H), 3.57–3.50 (m, 2H), 3.49–3.39 (m, 2H), 2.66–2.59 (m, 1H), 1.84.1.75 (m, 4H), 1.74–1.66 (m, 2H), 1.64–1.56 (m, 5H), 1.50–1.41 (m, 2H). ^13^C NMR (150 MHz, CDCl_3_) *δ* 176.0, 166.6, 165.4, 130.3, 127.0, 45.1, 44.4, 44.3, 42.1, 41.6, 41.3, 31.2, 28.2, 26.6.

HRMS (ESI-QTOF) *m*/*z* (M + Na)^+^ calcd for C_17_H_27_N_3_O_3_Na: 344.1950, found: 344.1933.

#### *N*-2-[4-[(Benzoyl)-1-piperazinyl]-2-oxoethyl]propenamide (6f)

The product was obtained following *general procedure D* using compound 5f (150 mg, 0.393 mmol) and Pd/C (30 mg) in dry MeOH and stirred for 2 h. Then dry DCM, acryloyl chloride (32 μL, 0.433 mmol), and DIPEA (205 μL, 1.179 mmol), added at room temperature. Work up C was used. The product was isolated following silica column chromatography (5% MeOH/DCM) to give a colourless oil. A white powder was obtained by dissolving the oil in a minimal amount of DCM and precipitating the product *via* hexane addition and solvent evaporation (65.7 mg, 55%).

^1^H NMR (600 MHz, CDCl_3_) *δ* 7.49–7.39 (m, 5H), 6.66, (br s, 1H), 6.32 (dd, *J* = 17.0, 1.2 Hz, 1H), 6.19 (dd, *J* = 17.0, 10.3 Hz, 1H), 5.69 (dd, *J* = 10.3, 1.3 Hz, 1H), 4.17 (s, 2H), 3.93–3.31 (m, 8H). ^13^C NMR (150 MHz, CDCl_3_) *δ* 170.7, 166.7, 134.9, 130.3, 130.3, 128.7, 127.1, 127.0, 44.4, 42.0, 41.3.

HRMS (ESI-QTOF) m/z (M + Na)^+^ calcd for C_16_H_19_N_3_O_3_Na: 324.1324, found: 324.1327.

#### *N*-2-[4-[((2-Biphenyl)carbonyl)-1-piperazinyl]-2-oxoethyl]propenamide (7a)

The compound was obtained by first following *general procedure B* using biphenyl-2-carboxylic acid (200 mg, 1.009 mmol), TCFH (311 mg, 1.110 mmol), NMI (281 μL, 3.532 mmol), and compound 4 (380 mg, 1.211 mmol) in a solution of ACN (10 mL) and H_2_O (approx. 0.5 mL). *General procedure D* was then followed using this crude intermediate without purification, Pd/C (20 mg) in dry MeOH and stirred for 2 h. Then dry DCM, acryloyl chloride (82 μL, 1.110 mmol) and DIPEA (527 μL, 3.027 mmol) added at room temperature. Work up C was used. The product was isolated following silica column chromatography (5% MeOH/DCM) to give a colourless oil. A white powder was obtained by dissolving the oil in a minimal amount of DCM and precipitating the product *via* hexane addition and solvent evaporation (118 mg, 31%).

^1^H NMR (600 MHz, CDCl_3_) *δ* 7.55–7.40 (m, 8H), 7.39–7.35 (m, 1H), 6.77–6.64 (m, 1H), 6.29 (dd, *J* = 17.1, 7.1 Hz, 1H), 6.16 (dd, *J* = 17.0, 10.3 Hz, 1H), 5.69 (d, *J* = 10.4 Hz, 1H), 4.14–3.83 (m, 4H), 3.50–3.40 (m, 1H), 3.39–3.27 (m, 1H), 3.00–2.88 (m, 2H), 2.87–2.79 (m, 1H), 2.16–1.95 (m, 1H). ^13^C NMR (150 MHz, CDCl_3_) *δ* 170.5, 170.4, 166.2, 166.2, 165.7, 139.5, 138.5, 138.3, 134.0, 130.2, 130.1, 129.9, 129.9, 129.4, 129.3, 128.8, 128.8, 128.8, 128.8, 128.2, 128.2, 128.1, 128.1, 128.0, 127.8, 127.5, 127.5, 46.0, 45.9, 43.6, 41.3, 41.2, 41.1.

HRMS (ESI-QTOF) *m*/*z* (M + Na)^+^ calcd for C_22_H_23_N_3_O_3_Na: 400.1637, found: 400.1616.

#### *N*-2-[4-[((3-Biphenyl)carbonyl)-1-piperazinyl]-2-oxoethyl]propenamide (7b)

The compound was obtained by first following *general procedure B* using biphenyl-2-carboxylic acid (200 mg, 1.009 mmol), TCFH (311 mg, 1.110 mmol), NMI (281 μL, 3.532 mmol), and compound 4 (380 mg, 1.211 mmol) in a solution of ACN (10 mL) and H_2_O (approx. 0.5 mL). *General procedure D* was then followed using this crude intermediate without purification, Pd/C (20 mg), in dry MeOH and stirred for 2 h. Then dry DCM, acryloyl chloride (82 μL, 1.110 mmol) and DIPEA (527 μL, 3.027 mmol) added at room temperature. Work up C was used. The product was isolated following silica column chromatography (5% MeOH/DCM) to give a colourless oil. A white powder was obtained by dissolving the oil in a minimal amount of DCM and precipitating the product *via* hexane addition and solvent evaporation (91.4 mg, 24%).

^1^H NMR (600 MHz, CDCl_3_) *δ* 7.64–7.60 (m, 1H), 7.57–7.55 (m, 1H), 7.53–7.50 (m, 2H), 7.45 (t, *J* = 7.7 Hz, 1H), 7.39 (t, *J* = 7.9 Hz, 2H), 7.34–7.30 (m, 2H), 6.70 (br s, 1H), 6.26 (dd, *J* = 17.1 Hz, 1H), 6.13 (dd, *J* = 17.0, 10.3 Hz, 1H), 5.65 (d, *J* = 10.3 Hz, 1H), 4.12 (s, 2H), 3.86–3.26 (m, 8H). ^13^C NMR (150 MHz, CDCl_3_) *δ* 170.8, 166.7, 165.7, 141.9, 140.0, 135.2, 130.0, 129.2, 129.1, 129.0, 127.9, 127.5, 127.1, 125.8, 125.7, 47.2, 44.4, 42.1, 41.3.

HRMS (ESI-QTOF) *m*/*z* (M + Na)^+^ calcd for C_22_H_23_N_3_O_3_Na: 400.1637, found: 400.1611.

#### *N*-2-[4-[((4-Biphenyl)carbonyl)-1-piperazinyl]-2-oxoethyl]propenamide (7c)

The compound was obtained by first following *general procedure B* using biphenyl-2-carboxylic acid (200 mg, 1.009 mmol), TCFH (311 mg, 1.110 mmol), NMI (281 μL, 3.532 mmol), and compound 4 (380 mg, 1.211 mmol) in a solution of ACN (10 mL) and H_2_O (approx. 0.5 mL). *General procedure D* was then followed using this crude intermediate without purification, Pd/C (20 mg) in dry MeOH and stirred for 2 h. Then dry DCM, acryloyl chloride (82 μL, 1.110 mmol), and DIPEA (527 μL, 3.027 mmol) added at room temperature. Work up C was used. The product was isolated following silica column chromatography (5% MeOH/DCM) to give a colourless oil. A white powder was obtained by dissolving the oil in a minimal amount of DCM and precipitating the product *via* hexane addition and solvent evaporation (103 mg, 27%).

^1^H NMR (600 MHz, CDCl_3_) *δ* 7.66 (d, *J* = 8.1 Hz, 2H), 7.60 (d, *J* = 7.8 Hz, 2H), 7.50 (d, *J* = 8.2 Hz, 2H), 7.47 (t, *J* = 7.5 Hz, 2H), 7.39 (t, *J* = 7.3 Hz, 1H), 6.68 (br s, 1H), 6.32 (d, *J* = 16.9 Hz, 1H), 6.20 (dd, *J* = 17.0, 10.2 Hz, 1H), 5.69 (d, *J* = 10.2 Hz, 1H), 4.24–4.11 (m, 2H), 3.95–3.37 (m, 8H). ^13^C NMR (150 MHz, CDCl_3_) *δ* 170.5, 166.7, 165.4, 143.3, 140.0, 133.5, 130.2, 128.9, 128.0, 127.7, 127.4, 127.2, 127.0, 44.5, 42.1, 41.3.

HRMS (ESI-QTOF) *m*/*z* (M + Na)^+^ calcd for C_22_H_23_N_3_O_3_Na: 400.1637, found: 400.1634.

#### 2′-Methyl-2-biphenylcarboxylic acid (10a)

The product was obtained following *general procedure A* using 4-iodobenzoic acid (606 mg, 2.44 mmol), 3-tolylboronic acid (500 mg, 3.68 mmol), Na_2_CO_3_ (773 mg, 7.29 mmol) and Pd(PPh_3_)_4_ (140 mg, 0.122 mmol) in a solution of dioxane (20 mL) and H_2_O (5 mL). The product was isolated following silica column chromatography (5% MeOH/DCM) and C18 flash chromatography (10–90% ACN/H_2_O gradient) to yield a white powder (342.9 mg, 66%).

^1^H NMR (400 MHz, CDCl_3_) *δ* 8.04 (dd, *J* = 7.9, 1.4 Hz, 1H), 7.57 (td, *J* = 7.5, 1.4 Hz, 1H), 7.43 (td, *J* = 7.7, 1.2 Hz, 1H), 7.29–7.17 (m, 4H), 7.08 (d, *J* = 7.5 Hz, 1H), 2.07 (s, 3H). ^13^C NMR (100 MHz, CDCl_3_) *δ* 171.1, 143.4, 141.1, 135.4, 132.4, 131.2, 130.8, 129.6, 128.9, 128.5, 127.4, 127.2, 125.3, 20.0.

Characterization is consistent with literature data.^39^

#### 3′-Methyl-2-biphenylcarboxylic acid (10b)

The product was obtained following *general procedure A* using 4-iodobenzoic acid (400 mg, 1.61 mmol), 3-tolylboronic acid (330 mg, 2.42 mmol), Na_2_CO_3_ (515 mg, 4.86 mmol) and Pd(PPh_3_)_4_ (95 mg, 0.082 mmol) in a solution of dioxane (16 mL) and H_2_O (4 mL). The product was isolated following silica column chromatography (5% MeOH/DCM) and C18 flash chromatography (10–90% ACN/H_2_O gradient) to yield a white powder (164.5 mg, 48%).

^1^H NMR (600 MHz, CDCl_3_) *δ* 7.93 (dd, *J* = 7.8, 1.2 Hz, 1H), 7.55 (td, *J* = 7.5, 1.3 Hz, 1H), 7.42 (td, *J* = 7.4, 1.1 Hz, 1H), 7.37 (d, *J* = 7.8 Hz, 1H), 7.28 (t, *J* = 8.0 Hz, 1H), 7.17 (d, *J* = 10.8 Hz, 2H), 7.14 (d, *J* = 7.7 Hz, 1H), 2.39 (s, 3H). ^13^C NMR (150 MHz, CDCl_3_) *δ* 171.7, 143.3, 140.9, 137.7, 132.0, 131.4, 130.6, 129.2, 129.1, 128.2, 128.0, 127.1, 125.6, 21.5.

Characterization is consistent with literature data.^39^

#### 4′-Methyl-2-biphenylcarboxylic acid (10c)

The product was obtained following *general procedure A* using 4-iodobenzoic acid (400 mg, 1.61 mmol), 3-tolylboronic acid (330 mg, 2.42 mmol), Na_2_CO_3_ (515 mg, 4.86 mmol) and Pd(PPh_3_)_4_ (95 mg, 0.082 mmol) in a solution of dioxane (16 mL) and H_2_O (4 mL). The product was isolated following silica column chromatography (5% MeOH/DCM) and C18 flash chromatography (10–90% ACN/H_2_O gradient) to yield a white powder (119.0 mg, 35%).

^1^H NMR (600 MHz, CDCl_3_) *δ* 7.93 (d, *J* = 7.7 Hz, 1H), 7.55 (t, *J* = 7.6 Hz, 1H), 7.41 (t, *J* = 7.5 Hz, 1H), 7.37 (d, *J* = 7.8 Hz, 1H), 7.25 (d, *J* = 8.0 Hz, 2H), 7.21 (d, *J* = 7.9 Hz, 2H), 2.40 (s, 3H). ^13^C NMR (150 MHz, CDCl_3_) δ 171.2, 143.2, 138.0, 137.2, 132.0, 131.2, 130.6, 129.1, 128.9, 128.4, 127.0, 21.2.

Characterization is consistent with literature data.^40^

#### 2′-Methyl-3-biphenylcarboxylic acid (10d)

The product was obtained following *general procedure A* using 4-iodobenzoic acid (400 mg, 1.61 mmol), 3-tolylboronic acid (330 mg, 2.42 mmol), Na_2_CO_3_ (515 mg, 4.86 mmol) and Pd(PPh_3_)_4_ (95 mg, 0.082 mmol) in a solution of dioxane (16 mL) and H_2_O (4 mL). The product was isolated following silica column chromatography (5% MeOH/DCM) and C18 flash chromatography (10–90% ACN/H_2_O gradient) to yield a white powder (108.1 mg, 32%).

^1^H NMR (600 MHz, CDCl_3_) *δ* 8.11–8.08 (m, 2H), 7.60–7.57 (m, *J* = 7.6 Hz, 1H), 7.53 (t, *J* = 7.8 Hz, 1H), 7.31–7.23 (m, 4H), 2.28 (s, 3H). ^13^C NMR (150 MHz, CDCl_3_) δ 170.8, 142.4, 140.6, 135.3, 134.5, 130.9, 130.5, 129.7, 129.1, 128.6, 128.3, 127.8, 125.9, 20.4.

Characterization is consistent with literature data.^41^

#### 3′-Methyl-3-biphenylcarboxylic acid (10e)

The product was obtained following *general procedure A* using 4-iodobenzoic acid (400 mg, 1.61 mmol), 3-tolylboronic acid (330 mg, 2.42 mmol), Na_2_CO_3_ (515 mg, 4.86 mmol) and Pd(PPh_3_)_4_ (95 mg, 0.082 mmol) in a solution of dioxane (16 mL) and H_2_O (4 mL). The product was isolated following silica column chromatography (5% MeOH/DCM) and C18 flash chromatography (10–90% ACN/H_2_O gradient) to yield a white powder (94.0 mg, 28%).

^1^H NMR (600 MHz, CDCl_3_) *δ* 8.34 (t, *J* = 1.7 Hz, 1H), 8.08 (dt, *J* = 7.7, 1.3 Hz, 1H), 7.84 (d, *J* = 7.7, 1H), 7.55 (t, *J* = 7.7 Hz, 1H), 7.45 (m, 2H), 7.37 (t, *J* = 8.6 Hz, 1H), 7.21 (d, *J* = 7.5 Hz, 1H), 2.44 (s, 3H). ^13^C NMR (150 MHz, CDCl_3_) *δ* 170.3, 141.8, 139.9, 138.6, 132.4, 129.5, 128.9, 128.9, 128.8, 128.6, 128.0, 124.3, 21.5.

Characterization is consistent with literature data.^42^

#### 4′-Methyl-3-biphenylcarboxylic acid (10f)

The product was obtained following *general procedure A* using 3-iodobenzoic acid (400 mg, 1.61 mmol), 4-tolylboronic acid (330 mg, 2.42 mmol), Na_2_CO_3_ (515 mg, 4.86 mmol) and Pd(PPh_3_)_4_ (95 mg, 0.082 mmol) in a solution of dioxane (16 mL) and H_2_O (4 mL). The product was isolated following silica column chromatography (5% MeOH/DCM) and C18 flash chromatography (10–90% ACN/H_2_O gradient) to yield a white powder (145.2 mg, 42%).

^1^H NMR (600 MHz, CDCl_3_) *δ* 8.33 (s, 1H), 8.06 (d, *J* = 7.7 Hz, 1H), 7.83 (d, *J* = 7.7 Hz, 1H), 7.56–7.52 (m, 3H), 7.28 (d, *J* = 8.0 Hz, 2H), 2.41 (s, 3H). ^13^C NMR (150 MHz, CDCl_3_) *δ* 169.9, 141.6, 137.7, 137.0, 132.1, 129.7, 129.5, 129.0, 128.7, 128.6, 127.0, 21.1.

Characterization is consistent with literature data.^43^

#### 2′-Methyl-4-biphenylcarboxylic acid (10g)

The product was obtained following *general procedure A* using 4-iodobenzoic acid (366 mg, 1.48 mmol), 2-tolylboronic acid (300 mg, 2.21 mmol), Na_2_CO_3_ (470 mg, 4.43 mmol) and Pd(PPh_3_)_4_ (85 mg, 0.073 mmol) in a solution of dioxane (16 mL) and H_2_O (4 mL). The product was isolated following silica column chromatography (5% MeOH/DCM) and C18 flash chromatography (10–90% ACN/H_2_O gradient) to yield a white powder (98.6 mg, 31%).

^1^H NMR (600 MHz, CDCl_3_) *δ* 8.15 (d, *J* = 8.3 Hz, 2H), 7.44 (d, *J* = 8.4 Hz, 2H), 7.31–7.26 (m, 3H), 7.24 (d, *J* = 7.4 Hz, 1H), 2.28 (s, 3H). ^13^C NMR (150 MHz, CDCl_3_) *δ* 170.0, 147.6, 140.7, 135.2, 130.6, 130.5, 130.1, 129.5, 129.4, 127.9, 127.4, 125.9, 125.2, 20.4.

Characterization is consistent with literature data.^44^

#### 3′-Methyl-4-biphenylcarboxylic acid (10h)

The product was obtained following *general procedure A* using 4-iodobenzoic acid (400 mg, 1.61 mmol), 3-tolylboronic acid (330 mg, 2.42 mmol), Na_2_CO_3_ (515 mg, 4.86 mmol) and Pd(PPh_3_)_4_ (95 mg, 0.082 mmol) in a solution of dioxane (16 mL) and H_2_O (4 mL). The product was isolated following silica column chromatography (5% MeOH/DCM) and C18 flash chromatography (10–90% ACN/H_2_O gradient) to yield a white powder (221.4 mg, 65%).

^1^H NMR (600 MHz, CDCl_3_) *δ* 8.17 (d, *J* = 8.4 Hz, 2H), 7.69 (d, *J* = 8.4 Hz, 2H), 7.45 (d, *J* = 10.2 Hz, 1H), 7.37 (t, *J* = 7.5 Hz, 1H), 7.23 (d, *J* = 7.4 Hz), 2.33 (s, 3H). ^13^C NMR (150 MHz, CDCl_3_) *δ* 169.8, 146.6, 139.9, 138.6, 130.7, 129.1, 128.9, 128.1, 127.6, 127.2, 124.4, 21.5.

Characterization is consistent with literature data.^45^

#### 4′-Methyl-4-biphenylcarboxylic acid (10i)

The product was obtained following *general procedure A* using 4-iodobenzoic acid (400 mg, 1.61 mmol), 4-tolylboronic acid (330 mg, 2.42 mmol), Na_2_CO_3_ (515 mg, 4.86 mmol) and Pd(PPh_3_)_4_ (95 mg, 0.082 mmol) in a solution of dioxane (16 mL) and H_2_O (4 mL). The product was isolated following silica column chromatography (5% MeOH/DCM) and recrystallization from hot methanol to yield a white powder (186.8 mg, 58%).

^1^H NMR (600 MHz, CDCl_3_) *δ* 8.16 (d, *J* = 8.6 Hz, 2H), 7.69 (d, *J* = 8.5 Hz, 2H), 7.55 (d, *J* = 8.1 Hz, 2H), 7.29 (d, *J* = 7.9 Hz, 2H), 2.42 (s, 3H). ^13^C NMR (150 MHz, CDCl_3_) *δ* 169.8, 146.4, 138.3, 137.0, 130.7, 129.7, 127.4, 127.2, 126.9, 21.2.

Characterization is consistent with literature data.^46^

#### Benzyl [2-(4-(2′-methyl-2-biphenyl)carbonylpiperazin-1-yl)-2-oxoethyl]carbamate (11a)

The product was obtained following *general procedure B* using acid 10a (200 mg, 0.942 mmol), TCFH (291 mg, 1.037 mmol), NMI (263 μL, 3.297 mmol), and compound 4 (355 mg, 1.130 mmol) in a solution of ACN (10 mL) and H_2_O (approx. 0.5 mL). The product was isolated following silica column chromatography (5% MeOH/DCM) to give a colourless oil. A white powder was obtained by dissolving the oil in a minimal amount of DCM and precipitating the product *via* hexane addition and solvent evaporation (311.7 mg, 70%).

^1^H NMR (600 MHz, CDCl_3_) *δ* 7.50–7.26 (m, 11H), 7.25–7.16 (m, 2H), 5.76–5.61 (m, 1H), 5.10 (s, 2H), 4.10–2.66 (m, 10), 2.34–2.12 (m, 3H). ^13^C NMR (150 MHz, CDCl_3_) *δ* 170.0, 166.3, 156.2, 137.3, 136.3, 135.5, 130.6, 129.6, 128.9, 128.5, 128.3, 128.3, 128.2, 128.1, 128.0, 127.8, 127.8, 125.4, 67.0, 45.9, 43.9, 42.5, 41.5, 41.2, 41.1, 39.0, 20.3, 20.2.

HRMS (ESI-QTOF) *m*/*z* (M + Na)^+^ calcd for C_28_H_29_N_3_O_4_Na: 494.2056, found: 494.2072.

#### Benzyl [2-(4-(3′-methyl-2-biphenyl)carbonylpiperazin-1-yl)-2-oxoethyl]carbamate (11b)

The product was obtained following *general procedure B* using acid 10b (245 mg, 1.154 mmol), TCFH (356 mg, 1.270 mmol), NMI (322 μL, 4.039 mmol), and compound 4 (435 mg, 1.385 mmol) in a solution of ACN (10 mL) and H_2_O (approx. 0.5 mL). The product was isolated following silica column chromatography (5% MeOH/DCM) to give a colourless oil. A white powder was obtained by dissolving the oil in a minimal amount of DCM and precipitating the product *via* hexane addition and solvent evaporation (325.7 mg, 60%).

^1^H NMR (600 MHz, CDCl_3_) *δ* 7.52–7.46 (m, 1H), 7.46–7.38 (m, 3H), 7.37–7.27 (m, 8H), 7.20–7.15 (m, 1H), 5.66 (m, 1H), 5.10 (m, 2H), 4.02–3.75 (m, 4H), 3.49–3.24 (m, 2H), 3.00–2.77 (m, 3H), 2.39 (s, 3H), 2.16–1.90 (m, 1H). ^13^C NMR (150 MHz, CDCl_3_) δ 170.2, 170.1, 156.1, 139.5, 138.4, 136.3, 134.3, 129.9, 129.3, 128.9, 128.8, 128.5, 128.2, 128.1, 128.0, 127.9, 127.9, 125.9, 46.0, 45.9, 43.6, 42.5, 41.2, 41.1, 21.5.

HRMS (ESI-QTOF) *m*/*z* (M + Na)^+^ calcd for C_28_H_29_N_3_O_4_Na: 494.2056, found: 494.2079.

#### Benzyl [2-(4-(4′-methyl-2-biphenyl)carbonylpiperazin-1-yl)-2-oxoethyl]carbamate (11c)

The product was obtained following *general procedure B* using acid 10c (200 mg, 0.942 mmol), TCFH (291 mg, 1.037 mmol), NMI (263 μL, 3.297 mmol), and compound 4 (355 mg, 1.130 mmol) in a solution of ACN (10 mL) and H_2_O (approx. 0.5 mL). The product was isolated following silica column chromatography (5% MeOH/DCM) to give a colourless oil. A white powder was obtained by dissolving the oil in a minimal amount of DCM and precipitating the product *via* hexane addition and solvent evaporation (354.0 mg, 80%).

^1^H NMR (600 MHz, CDCl_3_) *δ* 7.53–7.45 (m, 1H), 7.44–7.39 (m, 3H), 7.39–7.28 (m, 7H), 7.22 (t, *J* = 7.8 Hz, 2H), 5.70–5.61 (m, 2H), 5.12–5.07 (m, 2H), 3.96–3.93 (m, 1H), 3.86–3.73 (m, 3H), 3.48–3.30 (m, 2H), 3.10–2.85 (m, 2H), 2.85–2.73 (m, 1H), 2.38 (s, 3H), 2.31–2.02 (m, 1H). ^13^C NMR (150 MHz, CDCl_3_) *δ* 170.3, 170.2, 166.3, 166.3, 156.2, 138.4, 138.3, 138.1, 138.0, 136.6, 136.3, 134.3, 134.2, 129.9, 129.9, 129.5, 129.4, 129.3, 129.3, 128.6, 128.5, 128.2, 128.1, 128.9, 127.9, 127.8, 67.0, 46.0, 45.9, 43.6, 42.5, 42.5, 41.4, 41.2, 41.1, 41.1, 21.2.

HRMS (ESI-QTOF) *m*/*z* (M + Na)^+^ calcd for C_28_H_29_N_3_O_4_Na: 494.2056, found: 494.2065.

#### Benzyl [2-(4-(2′-methyl-3-biphenyl)carbonylpiperazin-1-yl)-2-oxoethyl]carbamate (11d)

The product was obtained following *general procedure B* using acid 10d (108 mg, 0.509 mmol), TCFH (157 mg, 0.559 mmol), NMI (142 μL, 1.782 mmol), and compound 4 (355 mg, 1.130 mmol) in a solution of ACN (10 mL) and H_2_O (approx. 0.5 mL). The product was isolated following silica column chromatography (5% MeOH/DCM) to give a colourless oil. A white powder was obtained by dissolving the oil in a minimal amount of DCM and precipitating the product *via* hexane addition and solvent evaporation (116.0 mg, 48%).

^1^H NMR (600 MHz, CDCl_3_) *δ* 7.49 (t, *J* = 7.5 Hz, 1H), 7.44–7.38 (m, 2H), 7.38–7.30 (m, 6H), 7.29–7.23 (m, 3H), 7.21 (d, *J* = 7.3 Hz, 1H), 5.76 (br s, 1H), 5.12 (s, 2H), 4.05 (s, 2H), 3.89–3.34 (m, 8H), 2.27 (s, 3H). ^13^C NMR (150 MHz, CDCl_3_) *δ* 170.6, 166.8, 156.2, 142.4, 140.6, 136.3, 135.1, 134.8, 131.1, 130.5, 129.7, 128.6, 128.2, 128.0, 127.8, 127.7, 126.0, 125.6, 47.1 44.4, 42.6, 42.1, 20.4.

HRMS (ESI-QTOF) *m*/*z* (M + Na)^+^ calcd for C_28_H_29_N_3_O_4_Na: 494.2056, found: 494.2064.

#### Benzyl [2-(4-(3′-methyl-3-biphenyl)carbonylpiperazin-1-yl)-2-oxoethyl]carbamate (11e)

The product was obtained following *general procedure B* using acid 10e (94 mg, 0.443 mmol), TCFH (137 mg, 0.487 mmol), NMI (124 μL, 1.551 mmol), and compound 4 (167 mg, 0.532 mmol) in a solution of ACN (10 mL) and H_2_O (approx. 0.5 mL). The product was isolated following silica column chromatography (5% MeOH/DCM) to give a colourless oil. A white powder was obtained by dissolving the oil in a minimal amount of DCM and precipitating the product *via* hexane addition and solvent evaporation (91.6 mg, 44%).

^1^H NMR (600 MHz, CDCl_3_) *δ* 7.71–7.68 (m, 1H), 7.64 (s, 1H), 7.52 (t, *J* = 7.6 Hz, 1H), 7.43–7.31 (m, 9H), 7.22 (d, *J* = 7.4 Hz, 1H), 5.76 (br s, 1H), 5.15 (s, 2H), 4.08 (s, 2H), 3.94–3.29 (m, 8H), 2.45 (s, 3H). ^13^C NMR (150 MHz, CDCl_3_) *δ* 170.7, 166.8, 156.2, 142.0, 140.0, 138.6, 136.3, 135.4, 129.1, 129.0, 128.9, 128.6, 128.6, 128.2, 128.1, 127.9, 125.8, 125.7, 124.2, 47.4, 44.4, 42.7, 21.5.

HRMS (ESI-QTOF) *m*/*z* (M + Na)^+^ calcd for C_28_H_29_N_3_O_4_Na: 494.2056, found: 494.2072.

#### Benzyl [2-(4-(4′-methyl-3-biphenyl)carbonylpiperazin-1-yl)-2-oxoethyl]carbamate (11f)

The product was obtained following *general procedure B* using acid 10f (120 mg, 0.565 mmol), TCFH (174 mg, 0.621 mmol), NMI (158 μL, 1.978 mmol), and compound 4 (213 mg, 0.678 mmol) in a solution of ACN (10 mL) and H_2_O (approx. 0.5 mL). The product was isolated following silica column chromatography (5% MeOH/DCM) to give a colourless oil. A white powder was obtained by dissolving the oil in a minimal amount of DCM and precipitating the product *via* hexane addition and solvent evaporation (109.1 mg, 41%).

^1^H NMR (600 MHz, CDCl_3_) *δ* 7.66 (d, *J* = 7.8 Hz, 1H), 7.61 (s, 1H), 7.50–7.46 (m, 3H), 7.40–7.29 (m, 6H), 7.29–7.26 (m, 2H), 5.73 (br s, 1H), 5.13 (s, 2H), 4.06 (s, 2H), 3.92–3.24 (m, 8H), 2.40 (s, 3H). ^13^C NMR (150 MHz, CDCl_3_) *δ* 170.7, 166.8, 156.2, 141.8, 137.8, 137.1, 136.3, 135.4, 129.7, 129.1, 128.7, 128.5, 128.2, 128.1, 126.9, 125.6, 125.5, 67.0, 44.4, 42.7, 42.2, 21.1.

HRMS (ESI-QTOF) *m*/*z* (M + Na)^+^ calcd for C_28_H_29_N_3_O_4_Na: 494.2056, found: 494.2068.

#### Benzyl [2-(4-(2′-methyl-4-biphenyl)carbonylpiperazin-1-yl)-2-oxoethyl]carbamate (11g)

The product was obtained following *general procedure B* using acid 10g (200 mg, 0.942 mmol), TCFH (291 mg, 1.037 mmol), NMI (263 μL, 3.297 mmol), and compound 4 (355 mg, 1.130 mmol) in a solution of ACN (10 mL) and H_2_O (approx. 0.5 mL). The product was isolated following silica column chromatography (5% MeOH/DCM) to give a colourless oil. A white powder was obtained by dissolving the oil in a minimal amount of DCM and precipitating the product *via* hexane addition and solvent evaporation (311.2 mg, 70%).

^1^H NMR (400 MHz, CDCl_3_) *δ* 7.50–7.44 (m, 2H), 7.43–7.26 (m, 10H), 7.25–7.19 (m, 1H), 5.76 (br s, 1H), 5.13 (s, 2H), 4.07 (s, 2H), 3.89–3.41 (m, 8H), 2.28 (s, 3H). ^13^C NMR (100 MHz, CDCl_3_) δ 170.7, 167.3, 166.8, 156.2, 144.1, 140.8, 136.3, 135.2, 133.2, 130.5, 129.6, 129.5, 128.5, 128.2, 128.1, 127.8, 127.0, 125.9, 42.7, 20.4.

HRMS (ESI-QTOF) *m*/*z* (M + Na)^+^ calcd for C_28_H_29_N_3_O_4_Na: 494.2056, found: 494.2060.

#### Benzyl [2-(4-(3′-methyl-4-biphenyl)carbonylpiperazin-1-yl)-2-oxoethyl]carbamate (11h)

The product was obtained following *general procedure B* using acid 10 h (200 mg, 0.942 mmol), TCFH (291 mg, 1.037 mmol), NMI (263 μL, 3.297 mmol), and compound 4 (355 mg, 1.130 mmol) in a solution of ACN (10 mL) and H_2_O (approx. 0.5 mL). The product was isolated following silica column chromatography (5% MeOH/DCM) to give a colourless oil. A white powder was obtained by dissolving the oil in a minimal amount of DCM and precipitating the product *via* hexane addition and solvent evaporation (182.0 mg, 41%).

^1^H NMR (600 MHz, CDCl_3_) *δ* 7.64 (d, *J* = 8.2 Hz, 2H), 7.48 (d, *J* = 8.2 Hz, 2H), 7.43–7.27 (m, 8H), 7.19 (d, *J* = 7.3 Hz, 1H), 5.74 (br s, 1H), 5.13 (s, 3H), 4.07 (s, 2H), 3.88–3.33 (m, 8H), 2.43 (s,3 H). ^13^C NMR (100 MHz, CDCl_3_) *δ* 170.6, 167.3, 166.8, 156.2, 143.4, 140.0, 138.6, 136.3, 133.4, 128.8, 128.7, 128.5, 128.2, 128.1, 127.9, 127.7, 127.4, 124.3, 42.7, 67.0, 42.7, 21.5.

HRMS (ESI-QTOF) *m*/*z* (M + Na)^+^ calcd for C_28_H_29_N_3_O_4_Na: 494.2056, found: 494.2076.

#### Benzyl [2-(4-(4′-methyl-4-biphenyl)carbonylpiperazin-1-yl)-2-oxoethyl]carbamate (11i)

The product was obtained following *general procedure B* using acid 10i (140 mg, 0.660 mmol), TCFH (204 mg, 0.726 mmol), NMI (184 μL, 2.31 mmol), and compound 4 (248.5 mg, 0.792 mmol) in a solution of ACN (10 mL) and H_2_O (approx. 0.5 mL). The product was isolated following silica column chromatography (5% MeOH/DCM) to give a colourless oil. A white powder was obtained by dissolving the oil in a minimal amount of DCM and precipitating the product *via* hexane addition and solvent evaporation (342.9 mg, 66%).

^1^H NMR (600 MHz, CDCl_3_) *δ* 7.63 (d, *J* = 8.2 Hz, 2H), 7.51–7.46 (m, 4H), 7.39–7.30 (m, 5H), 7.27 (d, *J* = 8.0 Hz, 2H), 5.75 (br s, 1H), 5.13 (s, 2H), 4.09 (s, 2H), 3.88–3.36 (m, 8H), 2.41 (s, 3H). ^13^C NMR (150 MHz, CDCl_3_) *δ* 170.6, 166.8, 156.2, 143.2, 137.9, 137.1, 136.3, 133.2, 129.7, 128.7, 128.3, 128.2, 128.1, 127.7, 127.1, 127.0, 67.0, 42.7, 21.1.

HRMS (ESI-QTOF) *m*/*z* (M + Na)^+^ calcd for C_28_H_29_N_3_O_4_Na: 494.2056, found: 494.2073.

#### 3-Benzylbenzoic acid (14)

To a round bottom flask equipped with a stir bar was added phenylboronic acid (183 mg, 1.5 mmol, 1.0 eq.), methyl 3-(chloromethyl)benzoate (332 mg, 1.8 mmol, 1.2 eq.), palladium(ii) chloride (3 mg, 0.015 mmol, 0.01 eq.), and K_2_CO_3_ (138 mg, 3.0 mmol, 2.0 eq.), and solubilized in a solution of DMF (12 mL) and H_2_O (3 mL). The reaction vessel was fitted with a a reflux condenser and the mixture was stirred at 90 °C overnight (approx. 16 h). The reaction mixture was then transferred to a separatory funnel where it was extracted with 20 mL ether (×3). The combined organic phase was then washed with brine (×3), dried over Na_2_SO_4_, and evaporated under vacuum to give a yellow oil. The oil was dissolved directly in THF (5 mL), MeOH (5 mL), and 2 M NaOH (5 mL) and stirred for 1 h at 80 °C. Upon completion, the mixture was acidified to pH = 2 with conc. HCl, then extracted with 20 mL EtOAc (×3). The combined organic phase was dried over MgSO_4_ and evaporated under vacuum to give the crude product as a golden oil. The pure product was obtained following silica column chromatography (3% MeOH/DCM) to give a white powder (164 mg, 52%).

^1^H NMR (600 MHz, CDCl_3_) *δ* 7.98–7.94 (m, 2H), 7.44 (d, *J* = 7.8 Hz, 1H), 7.39 (t, *J* = 7.6 Hz, 1H), 7.30 (t, *J* = 7.5 Hz, 2H), 7,24–7.18 (m, 3H), 4.05 (s, 2H). ^13^C NMR (150 MHz, CDCl_3_) δ 171.4, 141.7, 140.3, 134.4, 130.6, 129.3, 128.9, 128.7, 128.6, 128.1, 126.4, 41.7.

Characterization is consistent with literature data.^47^

#### 4-Benzylbenzoic acid (16)

To a round bottom flask equipped with a stir bar was added phenylboronic acid (183 mg, 1.5 mmol, 1.0 eq.), *tert*-butyl 4-(chloromethyl)benzoate (408 mg, 1.8 mmol, 1.2 eq.), palladium(ii) chloride (3 mg, 0.015 mmol, 0.01 eq.), and K_2_CO_3_ (138 mg, 3.0 mmol, 2.0 eq.), and solubilized in a solution of DMF (12 mL) and H_2_O (3 mL). The reaction mixture was stirred at 90 °C with a reflux condenser overnight (approx. 16 h). The reaction mixture was transferred to a separatory funnel where it was extracted with 20 mL ether (×3). The combined organic phase was then washed with brine (×3), dried over Na_2_SO_4_, and evaporated under vacuum to give a yellow oil. This oil was dissolved directly in a solution of TFA (5 mL) and DCM (5 mL) and stirred for 1 h. Upon completion, the solvent was evaporated under vacuum to give a yellow solid. The pure product was obtained following recrystallization from a 10 : 1 solution of MeOH and H_2_O to give a white powder (155 mg, 49%).

^1^H NMR (600 MHz, CDCl_3_) *δ* 8.00 (d, *J* = 8.2 Hz, 2H), 7.32–7.27 (m, 4H), 7.23 (t, *J* = 7.4 Hz, 1H), 7.18 (d, *J* = 7.4 Hz, 2H), 4.05 (s, 2H). ^13^C NMR (150 MHz, CDCl_3_) *δ* 170.2, 147.5, 139.9, 130.5, 129.1, 129.0, 128.6, 127.0, 126.4, 42.0.

Characterization is consistent with literature data.^48^

#### Ethyl 3-(phenylamino)benzoate (18a)

To a round bottom flask equipped with a magnetic stir bar was added ethyl 3-aminobenzoate (360 mg, 2.18 mmol, 1.0 eq.), phenylboronic acid (900 mg, 4.36 mmol, 2.0 eq.), benzoic acid (280 mg, 2.18 mmol, 1.0 eq.), copper(ii) acetate (120 mg, 0.44 mmol, 0.2 eq.) and K_2_CO_3_ (300 mg, 2.18 mmol, 1.0 eq.) and dissolved in EtOAc (50 mL). The reaction mixture was stirred at reflux overnight (approx. 16 h). Upon completion, the reaction mixture was washed in a separatory funnel with brine (×3), dried over Na_2_SO_4_, and the solvent was evaporated under vacuum to give the crude product as a brown oil. The product was isolated following silica column chromatography (10% EtOAc/hexanes) to give a white powder (239 mg, 45%).

^1^H NMR (400 MHz, CDCl_3_) *δ* 7.74–7.70 (m, 1H), 7.60–7.59 (m, 1H), 7.34–7.23 (m, 4H), 7.09 (d, *J* = 7.6 Hz, 2H), 6.98 (t, *J* = 7.4 Hz, 1H), 5.86 (br s, 1H), 4.37 (q, *J* = 7.1 Hz, 2H), 1.38 (t, *J* = 7.1 Hz, 3H). ^13^C NMR (100 MHz, CDCl_3_) *δ* 166.6, 143.5, 142.5, 131.7, 129.5, 129.3, 121.8, 121.7, 121.4, 118.3, 118.3, 61.0, 14.3.

Characterization is consistent with literature data.^49^

#### Ethyl-4-(phenylamino)benzoate (18b)

To a round bottom flask equipped with a magnetic stir bar was added ethyl 4-aminobenzoate (400 mg, 1.64 mmol, 1.0 eq.), phenylboronic acid (271 mg, 3.28 mmol, 2.0 eq.), benzoic acid (200 mg, 1.64 mmol, 1.0 eq.), copper(ii) acetate (298 mg, 1.64 mmol, 1.0 eq.) and K_2_CO_3_ (227 mg, 1.64 mmol, 1.0 eq.) and dissolved in EtOAc (40 mL). The reaction mixture was stirred at reflux overnight (approx. 16 h). Upon completion, the reaction mixture was washed in a separatory funnel with brine (×3), dried over Na_2_SO_4_, and the solvent was evaporated under vacuum to give the crude product as a brown oil. The product was isolated following silica column chromatography (10% EtOAc/hexanes) to give a white powder (165 mg, 35%).

^1^H NMR (600 MHz, CDCl_3_) *δ* 7.93 (d, *J* = 8.7 Hz, 2H), 7.30–7.27 (m, 2H), 7.17 (d, *J* = 7.7 Hz, 2H), 7.06 (t, *J* = 7.5 Hz, 1H), 6.99 (d, *J* = 8.7 Hz, 2H), 6.01 (br s, 1H), 4.34 (q, *J* = 7.1 Hz, 2H), 1.38 (t, *J* = 7.1 Hz, 3H). ^13^C NMR (150 MHz, CDCl_3_) *δ* 166.5, 147.9, 140.9, 131.4, 129.5, 123.0, 121.5, 120.3, 114.6, 60.4, 14.4.

Characterization is consistent with literature data.^50^

#### 3-(Phenylamino)benzoic acid (19a)

To a round bottom flask equipped with a stir bar was added ethyl 3-(phenylamino)benzoate (18a) (220 mg, 0.91 mmol, 1.0 eq.), which was dissolved in THF (7 mL), MeOH (7 mL) and 2 M NaOH (7 mL). The reaction mixture was stirred at 80 °C for 1 h until deemed complete by TLC (10% EtOAc/hexanes). Upon completion, the mixture was acidified to pH = 2 with conc. HCl, then extracted with 20 mL EtOAc (×3). The combined organic phase was dried over MgSO_4_ and evaporated under vacuum to give the crude product as a golden oil. The pure product was isolated following silica column chromatography (3% MeOH/DCM) to give a white powder (192 mg, 99%).

^1^H NMR (400 MHz, CDCl_3_) *δ* 7.78 (s, 1H), 7.64 (d, *J* = 7.5 Hz, 1H), 7.38–7.28 (m, 4H), 7.11 (d, *J* = 7.5 Hz, 2H), 7.00 (t, *J* = 7.4 Hz, 1H). ^13^C NMR (100 MHz, CDCl_3_) *δ* 171.3, 143.7, 142.1, 130.3, 129.5, 129.5, 122.3, 122.1, 122.0, 118.6, 118.4.

Characterization is consistent with literature data.^51^

#### 4-(Phenylamino)benzoic acid (19b)

To a round bottom flask equipped with a stir bar was added ethyl 3-(phenylamino)benzoate (18b) (240 mg, 0.91 mmol, 1.0 eq.), which was dissolved in THF (10 mL), MeOH (10 mL) and 2 M NaOH (10 mL). The reaction mixture was stirred at 80 °C for 1 h until deemed complete by TLC (10% EtOAc/hexanes). Upon completion, the mixture was acidified to pH = 2 with conc. HCl, then extracted with 20 mL EtOAc (×3). The combined organic phase was dried over MgSO_4_ and evaporated under vacuum to give the crude product as a golden oil. The pure product was isolated following silica column chromatography (3% MeOH/DCM) to give a white powder (185 mg, 88%).

^1^H NMR (600 MHz, CDCl_3_) *δ* 7.98 (d, *J* = 8.9 Hz, 2H), 7.37–7.34 (m, 2H), 7.20 (d, *J* = 7.8 Hz, 2H), 7.10 (t, *J* = 7.4 Hz, 1H), 7.00 (d, *J* = 8.8 Hz, 2H). ^13^C NMR (150 MHz, CDCl_3_) *δ* 171.5, 148.9, 140.5, 132.3, 129.5, 123.5, 120.8, 119.9, 114.4.

Characterization is consistent with literature data.^50^

#### Benzyl [2-(4-(2-benzylphenyl)carbonylpiperazin-1-yl)-2-oxoethyl]carbamate (20a)

The product was obtained following *general procedure B* using 2-benzylbenzoic acid (150 mg, 0.707 mmol), TCFH (218 mg, 0.777 mmol), NMI (197 μL, 2.475 mmol), and compound 4 (266 mg, 0.848 mmol) in a solution of ACN (10 mL) and H_2_O (approx. 0.5 mL). The product was isolated following silica column chromatography (5% MeOH/DCM) and solvent evaporation to give a colourless oil. A white powder was obtained by dissolving the oil in a minimal amount of DCM and precipitating the product *via* hexane addition and solvent evaporation (115.0 mg, 34%).

^1^H NMR (600 MHz, CDCl_3_) *δ* 7.41–7.29 (m, 7H), 7.29–7.26 (m, 1H), 7.25–7.20 (m, 2H), 7.18–7.13 (m, 1H), 7.13–7.08 (m, 3H), 5.74–5.66 (m, 1H), 5.16–5.08 (m, 2H), 4.23 (t, *J* = 14.0 Hz, 1H), 4.01 (s, 1H), 3.93–3.78 (m, 2H), 3.72–3.65 (m, 0.5H), 3.65–3.56 (m, 2H), 3.50–3.42 (m, 0.5 H), 3.40–3.32 (m, 0.5H), 3.30–3.20 (m, 1H), 3.02–2.89 (m, 1.5H), 2.80–2.72 (m, 0.5H), 2.55–2.43 (m, 1H), 2.38–2.28 (m, 0.5H). ^13^C NMR (150 MHz, CDCl_3_) *δ* 169.9, 169.8, 166.6, 166.5, 156.2, 140.3, 140.2, 138.6, 136.3, 135.3, 135.2, 131.4, 131.3, 129.5, 129.2, 129.2, 128.5, 128.5, 128.4, 128.2, 128.0, 126.5, 126.3, 67.0, 46.2, 46.2, 43.8, 43.6, 42.6, 42.5, 41.5, 41.3, 40.9, 40.8, 39.1.

HRMS (ESI-QTOF) *m*/*z* (M + Na)^+^ calcd for C_28_H_29_N_3_O_4_Na: 494.2056, found: 494.2045.

#### Benzyl[2-(4-(2-phenoxyphenyl)carbonylpiperazin-1-yl)-2-oxoethyl]carbamate (20b)

The product was obtained following *general procedure B* using 2-phenoxybenzoic acid (200 mg, 0.934 mmol), TCFH (288 mg, 1.027 mmol), NMI (261 μL, 3.269 mmol), and compound 4 (352 mg, 1.123 mmol) in a solution of ACN (10 mL) and H_2_O (approx. 0.5 mL). The product was isolated following silica column chromatography (5% MeOH/DCM) and solvent evaporation to give a white powder (217.0 mg, 46%).

^1^H NMR (600 MHz, CDCl_3_) *δ* 7.42–7.28 (m, 9H), 7.20–7.11 (m, 2H), 6.98 (d, *J* = 7.9 Hz, 2H), 6.89 (t, *J* = 7.9 Hz, 1H), 5.80–5.69 (m, 1H), 5.12 (s, 2H), 4.13–3.21 (m, 10H). ^13^C NMR (150 MHz, CDCl_3_) *δ* 167.3, 167.2, 166.7, 166.5, 156.2, 156.1, 153.1, 152.9, 136.3, 131.0, 130.0, 130.0, 129.0, 128.9, 128.5, 128.2, 128.1, 128.0, 124.1, 124.0, 123.8, 119.0, 118.9, 118.3, 118.1, 46.7, 46.6, 44.6, 44.2, 42.6, 42.3, 41.8, 41.5, 41.1.

HRMS (ESI-QTOF) *m*/*z* (M + Na)^+^ calcd for C_27_H_27_N_3_O_5_Na: 496.1848, found: 496.1844.

#### Benzyl[2-(4-((2-phenylamino)phenyl)carbonylpiperazin-1-yl)-2-oxoethyl]carbamate (20c)

The product was obtained following *general procedure B* using 2-(phenylamino)benzoic acid (200 mg, 0.938 mmol), TCFH (289 mg, 1.032 mmol), NMI (261 μL, 3.283 mmol), and compound 4 (352 mg, 1.126 mmol) in a solution of ACN (15 mL) and H_2_O (approx. 1.0 mL). The product was isolated following silica column chromatography (5% MeOH/DCM) and solvent evaporation to give a colourless oil. A white powder was obtained by dissolving the oil in a minimal amount of DCM and precipitating the product *via* hexane addition and solvent evaporation (110.0 mg, 25%).

^1^H NMR (600 MHz, CDCl_3_) *δ* 7.41 (d, *J* = 8.3 Hz, 1H), 7.39–7.32 (m, 5H), 7.32–7.26 (m, 3H), 7.18 (d, *J* = 7.4 Hz, 1H), 7.10 (d, *J* = 8.0 Hz, 2H), 6.99 (t, *J* = 7.4 Hz, 1H), 6.92 (t, *J* = 7.5 Hz, 1H), 5.75 (br s, 1H), 5.14 (s, 2H), 4.08–3.97 (m, 2H), 3.80–3.56 (m, 6H), 3.48–3.34 (m, 2H). ^13^C NMR (150 MHz, CDCl_3_) *δ* 170.2, 166.7, 156.2, 142.6, 141.9, 136.3, 131.0, 129.4, 128.5, 128.2, 128.1, 128.0, 122.3, 121.9, 119.8, 119.0, 117.4, 67.0, 44.4, 42.6, 42.1.

HRMS (ESI-QTOF) *m*/*z* (M + Na)^+^ calcd for C_27_H_28_N_4_O_4_Na: 495.2032, found: 495.2038.

#### Benzyl[2-(4-(3-benzylphenyl)carbonylpiperazin-1-yl)-2-oxoethyl]carbamate (20d)

The product was obtained following *general procedure B* using 14 (150 mg, 0.707 mmol), TCFH (218 mg, 0.777 mmol), NMI (197 μL, 2.475 mmol), and compound 4 (266 mg, 0.848 mmol) in a solution of ACN (10 mL) and H_2_O (approx. 0.5 mL). The product was isolated following silica column chromatography (5% MeOH/DCM) and solvent evaporation to give a colourless oil. A white powder was obtained by dissolving the oil in a minimal amount of DCM and precipitating the product *via* hexane addition and solvent evaporation (193.5 mg, 58%).

^1^H NMR (600 MHz, CDCl_3_) *δ* 7.38–7.26 (m, 9H), 7.25–7.20 (m, 2H), 7.20–7.16 (m, 3H), 5.73 (br s, 1H), 5.13 (s, 2H), 4.04 (s, 2H), 4.01 (s, 2H), 3.88–3.19 (m, 8H). ^13^C NMR (150 MHz, CDCl_3_) *δ* 170.7, 166.8, 156.2, 142.0, 140.2, 136.3, 135.0, 130.8, 129.0, 128.8, 128.6, 128.5, 128.2, 128.1, 127.6, 126.4, 124.9, 67.0, 47.2, 44.3, 42.6, 41.7.

HRMS (ESI-QTOF) *m*/*z* (M + Na)^+^ calcd for C_28_H_29_N_3_O_4_Na: 494.2056, found: 494.2082.

#### Benzyl[2-(4-(3-phenoxyphenyl)carbonylpiperazin-1-yl)-2-oxoethyl]carbamate (20e)

The product was obtained following *general procedure B* using 2-phenoxybenzoic acid (200 mg, 0.934 mmol), TCFH (288 mg, 1.027 mmol), NMI (261 μL, 3.269 mmol), and compound 4 (352 mg, 1.123 mmol) in a solution of ACN (10 mL) and H_2_O (approx. 0.5 mL). The product was isolated following silica column chromatography (5% MeOH/DCM) and solvent evaporation to give a white powder (256.6 mg, 54%).

^1^H NMR (600 MHz, CDCl_3_) *δ* 7.42–7.28 (m, 8H), 7.16 (t, *J* = 11.2 Hz, 1H), 7.13–7.06 (m, 2H), 7.06–6.99 (m, 3H), 5.75 (br s, 1H), 5.12 (s, 2H), 4.04 (s, 2H), 3.89–3.24 (m, 8H). ^13^C NMR (150 MHz, CDCl_3_) *δ* 169.8, 166.8, 157.9, 156.2, 136.5, 136.3, 130.2, 130.0, 128.5, 128.2, 128.0, 124.1, 121.4, 120.0, 119.5, 116.8, 67.0, 42.6.

HRMS (ESI-QTOF) *m*/*z* (M + Na)^+^ calcd for C_27_H_27_N_3_O_5_Na: 496.1848, found: 496.1848.

#### Benzyl[2-(4-((3-phenylamino)phenyl)carbonylpiperazin-1-yl)-2-oxoethyl]carbamate (20f)

The product was obtained following *general procedure B* using acid 19a (200 mg, 0.938 mmol), TCFH (289 mg, 1.032 mmol), NMI (261 μL, 3.283 mmol), and compound 4 (352 mg, 1.126 mmol) in a solution of ACN (15 mL) and H_2_O (approx. 1.0 mL). The product was isolated following silica column chromatography (5% MeOH/DCM) and solvent evaporation to give a colourless oil (313.5 mg, 71%).

^1^H NMR (600 MHz, CDCl_3_) *δ* 7.38–7.27 (m, 9H), 7.25–7.21 (m, 2H), 7.20–7.16 (m, 3H), 5.74 (br s, 1H), 5.13 (s, 2H), 4.08–4.02 (m, 2H), 3.87–3.15 (m, 8H). ^13^C NMR (150 MHz, CDCl_3_) *δ* 170.7, 166.8, 156.2, 142.0, 140.2, 136.3, 135.0, 130.8, 129.0, 128.8, 128.6, 128.5, 128.2, 128.1, 127.6, 126.4, 124.9, 67.0, 44.3, 42.6, 41.7.

HRMS (ESI-QTOF) *m*/*z* (M + Na)^+^ calcd for C_27_H_28_N_4_O_4_Na: 495.2008, found: 495.2013.

#### Benzyl[2-(4-(4-benzylphenyl)carbonylpiperazin-1-yl)-2-oxoethyl]carbamate (20g)

The product was obtained following *general procedure B* using acid 16 (150 mg, 0.707 mmol), TCFH (218 mg, 0.777 mmol), NMI (197 μL, 2.475 mmol), and compound 4 (266 mg, 0.848 mmol) in a solution of DMF (10 mL) and H_2_O (approx. 0.5 mL). The product was isolated following silica column chromatography (5% MeOH/DCM) and solvent evaporation to give a colourless oil. A white powder was obtained by dissolving the oil in a minimal amount of DCM and precipitating the product *via* hexane addition and solvent evaporation (263.4 mg, 79%).

^1^H NMR (600 MHz, CDCl_3_) *δ* 7.38–7.28 (m, 9H), 7.25 (d, *J* = 8.1 Hz, 2H), 7.22 (t, *J* = 7.5 Hz, 1H), 7.18 (d, *J* = 7.6 Hz, 2H), 5.74 (br s, 1H), 5.12 (s, 2H), 4.04 (s, 2H), 4.01 (s, 2H), 3.88–3.20 (m, 8H). ^13^C NMR (150 MHz, CDCl_3_) *δ* 170.7, 166.8, 156.2, 143.7, 140.2, 136.3, 132.6, 129.2, 129.0, 128.6, 128.5, 128.0, 127.4, 126.4, 67.0, 44.4, 42.6, 42.0, 41.8.

HRMS (ESI-QTOF) *m*/*z* (M + Na)^+^ calcd for C_28_H_29_N_3_O_4_Na: 494.2056, found: 494.2036.

#### Benzyl[2-(4-(4-phenoxyphenyl)carbonylpiperazin-1-yl)-2-oxoethyl]carbamate (20h)

The product was obtained following *general procedure B* using 4-phenoxybenzoic acid (200 mg, 0.934 mmol), TCFH (288 mg, 1.027 mmol), NMI (261 μL, 3.269 mmol), and compound 4 (352 mg, 1.123 mmol) in a solution of ACN (10 mL) and H_2_O (approx. 0.5 mL). The product was isolated following silica column chromatography (5% MeOH/DCM) and solvent evaporation to give a white powder (321.0 mg, 68%).

^1^H NMR (600 MHz, CDCl_3_) *δ* 7.44–7.37 (m, 4H), 7.37–7.29 (m, 5H), 7.18 (t, *J* = 7.4 Hz, 1H), 7.05 (d, *J* = 7.7 Hz, 1H), 7.02 (d, *J* = 8.6 Hz, 1H), 5.74 (br s, 1H), 5.13 (s, 2H), 4.06 (s, 2H), 3.79–3.38 (m, 8H). ^13^C NMR (150 MHz, CDCl_3_) *δ* 170.4, 166.8, 159.5, 156.2, 155.9, 136.3, 130.0, 129.3, 128.8, 128.5, 128.2, 128.1, 124.3, 119.8, 118.0, 67.0, 44.4, 42.6, 42.1.

HRMS (ESI-QTOF) *m*/*z* (M + Na)^+^ calcd for C_27_H_27_N_3_O_5_Na: 496.1872, found: 496.1865.

#### Benzyl[2-(4-((4-phenylamino)phenyl)carbonylpiperazin-1-yl)-2-oxoethyl]carbamate (20i)

The product was obtained following *general procedure B* using acid 19b (180 mg, 0.844 mmol), TCFH (260 mg, 0.929 mmol), NMI (235 μL, 2.954 mmol), and compound 4 (318 mg, 1.013 mmol) in a solution of ACN (10 mL) and H_2_O (approx. 0.5 mL). After solvent evaporation, an off-white solid was obtained, which was purified by rinsing with acetone (×3) to afford the pure product as a white powder (229.0 mg, 57%).

^1^H NMR (600 MHz, CDCl_3_) *δ* 7.38–7.29 (m, 9H), 7.14 (d, *J* = 7.6 Hz, 2H), 7.06–7.00 (m, 3H), 5.93 (br s, 1H), 5.76 (br s, 1H), 5.13 (s, 2H), 4.08–3.99 (m, 2H), 3.75–3.58 (m, 6H), 3.49–3.38 (m, 2H). ^13^C NMR (150 MHz, CDCl_3_) *δ* 170.8, 166.8, 156.2, 145.8, 141.3, 136.3, 129.5, 129.4, 128.5, 128.2, 128.1, 125.7, 122.7, 119.7, 115.5, 67.0, 44.4, 42.7, 42.1.

HRMS (ESI-QTOF) *m*/*z* (M + Na)^+^ calcd for C_27_H_28_N_4_O_4_Na: 495.2008, found: 495.1980.

#### *N*-2-[4-[((2′-Methyl-2-biphenyl)carbonyl)-1-piperazinyl]-2-oxoethyl]propenamide (21a)

The product was obtained following *general procedure D* using compound 11a (165 mg, 0.350 mmol) and Pd/C (20 mg) in dry MeOH and stirred for 2 h. Then dry DCM, acryloyl chloride (31 μL, 0.384 mmol), and DIPEA (183 μL, 1.05 mmol) added at room temperature. Work up C was used. The product was isolated following silica column chromatography (5% MeOH/DCM) to give a colourless oil. A white powder was obtained by dissolving the oil in a minimal amount of DCM and precipitating the product *via* hexane addition and solvent evaporation (60.0 mg, 44%).

^1^H NMR (600 MHz, CDCl_3_) *δ* 7.51–6.99 (m, 8H), 6.68–6.50 (m, 1H), 6.29 (d, *J* = 17.0 Hz, 1H), 6.16 (dd, *J* = 17.0, 10.3 Hz, 1H), 5.67 (d, *J* = 10.2 Hz, 1H), 4.14–3.93 (m, 2H), 3.91–2.65 (m, 8H), 2.40–2.10 (m, 3H). ^13^C NMR (150 MHz, CDCl_3_) *δ* 170.6, 166.7, 165.4, 142.0, 140.0, 138.6, 135.4, 130.2, 129.1, 129.0, 128.9, 128.6, 127.9, 127.0, 125.8, 125.7, 124.2, 44.4, 42.0, 41.2, 21.5.

HRMS (ESI-QTOF) *m*/*z* (M + Na)^+^ calcd for C_23_H_25_N_3_O_3_Na: 414.1794, found: 414.1776.

#### *N*-2-[4-[((3′-Methyl-2-biphenyl)carbonyl)-1-piperazinyl]-2-oxoethyl]propenamide (21b)

The product was obtained following *general procedure D* using compound 11b (160 mg, 0.339 mmol) and Pd/C (20 mg) in dry MeOH and stirred for 2 h. Then dry DCM, acryloyl chloride (31 μL, 0.373 mmol), and DIPEA (177 μL, 1.017 mmol) added at room temperature. Work up C was used. The product was isolated following silica column chromatography (5% MeOH/DCM) to give a colourless oil. A white powder was obtained by dissolving the oil in a minimal amount of DCM and precipitating the product *via* hexane addition and solvent evaporation (92.2 mg, 69%).

^1^H NMR (600 MHz, CDCl_3_) *δ* 7.52–7.47 (m, 1H), 7.46–7.40 (m, 3H), 7.33–7.26 (m, 3H), 7.18 (d, *J* = 7.0 Hz, 1H), 6.59 (m, 1H), 6.28 (dd, *J* = 17.3, 7.8 Hz, 1H), 6.15 (dd, *J* = 17.0, 10.3 Hz, 1H), 5.66 (d, *J* = 10.4 Hz, 1H), 4.12–4.01 (m, 1H), 3.98–3.82 (m, 2.5H), 3.50–3.39 (m, 1H), 3.39–3.25 (m, 1H), 3.06–3.00 (m, 0.5H), 2.97–2.87 (m, 2H), 2.87–2.77 (m, 1H), 2.39 (s, 3H), 2.18–1.94 (m, 1H). ^13^C NMR (150 MHz, CDCl_3_) *δ* 170.2, 170.1, 166.2, 166.1, 165.3, 139.5, 138.5, 138.5, 138.4, 138.4, 134.3, 130.2, 130.2, 129.9, 129.9, 129.4, 129.4, 129.3, 129.3, 128.9, 128.8, 128.7, 128.6, 128.9, 127.9, 127.9, 127.0127.0, 125.9, 46.0, 45.9, 43.6, 43.6, 41.3, 41.2, 41.1, 41.1, 41.1, 21.5.

HRMS (ESI-QTOF) *m*/*z* (M + Na)^+^ calcd for C_23_H_25_N_3_O_3_Na: 414.1794, found: 414.1783.

#### *N*-2-[4-[((4′-Methyl-2-biphenyl)carbonyl)-1-piperazinyl]-2-oxoethyl]propenamide (21c)

The product was obtained following *general procedure D* using compound 11c (150 mg, 0.318 mmol) and Pd/C (30 mg) in dry MeOH and stirred for 2 h. Then dry DCM, acryloyl chloride (32 μL, 0.350 mmol), and DIPEA (166 μL, 0.954 mmol) added at room temperature. Work up C was used. The product was isolated following silica column chromatography (5% MeOH/DCM) to give a colourless oil. A white powder was obtained by dissolving the oil in a minimal amount of DCM and precipitating the product *via* hexane addition and solvent evaporation (57.0 mg, 46%).

^1^H NMR (600 MHz, CDCl_3_) *δ* 7.51–7.46 (m, 1H), 7.45–7.39 (m, 3H), 7.39–7.35 (m, 2H), 7.25–7.19 (m, 2H), 6.64–6.53 (m, 1H), 6.28 (dd, *J* = 17.1, 7.2 Hz, 1H), 6.15 (dd, *J* = 17.0, 10.1 Hz, 1H), 5.66 (dd, *J* = 10.3 Hz, 1H), 4.07 (d, *J* = 4.1 Hz, 1H), 3.94 (d, *J* = 3.9 Hz, 1H), 3.88–3.75 (m, 1H), 3.48–3.31 (m, 2H), 3.11–2.86 (m, 3H), 2.86–2.75 (m, 1H), 2.38 (s, 3H), 2.33–2.06 (m, 1H). ^13^C NMR (150 MHz, CDCl_3_) *δ* 170.3, 170.2, 166.3, 166.2, 165.3, 138.4, 138.3, 138.1, 138.0, 136.6, 134.2, 130.2, 130.2, 129.9, 129.9, 128.6, 128.6, 127.9, 127.8, 127.8, 127.8, 127.0, 127.0, 46.0, 45.9, 43.7, 41.4, 41.2, 41.2, 41.1, 41.1, 41.0.

HRMS (ESI-QTOF) *m*/*z* (M + Na)^+^ calcd for C_23_H_25_N_3_O_3_Na: 414.1794, found: 414.1794.

#### *N*-2-[4-[((2′-Methyl-3-biphenyl)carbonyl)-1-piperazinyl]-2-oxoethyl]propenamide (21d)

The product was obtained following *general procedure D* using compound 11d (163 mg, 0.346 mmol) and Pd/C (20 mg) in dry MeOH and stirred for 2 h. Then dry DCM, acryloyl chloride (31 μL, 0.380 mmol), and DIPEA (181 μL, 1.038 mmol) added at room temperature. Work up C was used. The product was isolated following silica column chromatography (5% MeOH/DCM) to give a colourless oil. A white powder was obtained by dissolving the oil in a minimal amount of DCM and precipitating the product *via* hexane addition and solvent evaporation (65.0 mg, 40%).

^1^H NMR (600 MHz, CDCl_3_) *δ* 7.49 (t, *J* = 7.6 Hz, 1H), 7.45–7.39 (m, 2H), 7.36 (s, 1H), 7.31–7.27 (m, 2H), 7.27–7.24 (m, 2H), 7.23–7.19 (m, 1H), 6.67 (br s, 1H), 6.32 (d, *J* = 17.1 Hz, 1H), 6.19 (dd, *J* = 17.0, 10.3 Hz, 1H), 5.69 (d, *J* = 10.3 Hz, 1H), 4.17 (s, 2H), 3.95–3.35 (m, 8H), 2.27 (s, 3H). ^13^C NMR (150 MHz, CDCl_3_) *δ* 170.6, 166.7, 165.4, 142.4, 140.6, 135.2, 134.7, 131.1, 130.5, 130.2, 129.7, 128.6, 127.8, 127.7, 127.0, 126.0, 125.6, 44.4, 42.1, 41.3, 20.5.

HRMS (ESI-QTOF) *m*/*z* (M + Na)^+^ calcd for C_23_H_25_N_3_O_3_Na: 414.1794, found: 414.1798.

#### *N*-2-[4-[((3′-Methyl-3-biphenyl)carbonyl)-1-piperazinyl]-2-oxoethyl]propenamide (21e)

The product was obtained following *general procedure D* using compound 11e (130 mg, 0.276 mmol) and Pd/C (20 mg) in dry MeOH and stirred for 2 h. Then dry DCM, acryloyl chloride (25 μL, 0.303 mmol), and DIPEA (144 μL, 0.954 mmol) added at room temperature. Work up C was used. The product was isolated following silica column chromatography (5% MeOH/DCM) to give a colourless oil. A white powder was obtained by dissolving the oil in a minimal amount of DCM and precipitating the product *via* hexane addition and solvent evaporation (65.0 mg, 60%).

^1^H NMR (600 MHz, CDCl_3_) *δ* 7.68 (m, 1H), 7.62 (m, 1H), 7.50 (t, *J* = 7.7 Hz, 1H), 7.41–7.32 (m, 4H), 7.20 (d, *J* = 7.2 Hz, 1H), 6.66 (br s, 1H), 6.32 (dd, *J* = 1.2, 17.0 Hz, 1H), 6.19 (dd, *J* = 10.3, 17.0 Hz, 1H), 5.69 (dd, *J* = 1.4, 10.2 Hz, 1H), 4.17 (s, 2H), 3.95–3.31 (m, 8H), 2.43 (s, 3H). ^13^C NMR (150 MHz, CDCl_3_) *δ* 170.7, 166.7, 165.4, 142.0, 140.0, 138.6, 135.4, 130.2, 129.1, 129.0, 128.9, 128.6, 127.9, 127.1, 125.8, 125.7, 124.2, 41.3, 21.5.

HRMS (ESI-QTOF) *m*/*z* (M + Na)^+^ calcd for C_23_H_25_N_3_O_3_Na: 414.1794, found: 414.1819.

#### *N*-2-[4-[((4′-Methyl-3-biphenyl)carbonyl)-1-piperazinyl]-2-oxoethyl]propenamide (21f)

The product was obtained following *general procedure D* using compound 11f (109 mg, 0.231 mmol) and Pd/C (20 mg) in dry MeOH and stirred for 2 h. Then dry DCM, acryloyl chloride (21 μL, 0.245 mmol), and DIPEA (121 μL, 0.693 mmol) added at room temperature. Work up C was used. The product was isolated following silica column chromatography (5% MeOH/DCM) to give a colourless oil. A white powder was obtained by dissolving the oil in a minimal amount of DCM and precipitating the product *via* hexane addition and solvent evaporation (41.1 mg, 45%).

^1^H NMR (600 MHz, CDCl_3_) *δ* 7.68–7.65 (m, 1H), 7.62–7.60 (m, 1H), 7.51–7.47 (m, 3H), 7.36–7.34 (m, 1H), 7.28–2.26 (m, 2H), 6.66 (br s, 1H), 6.32 (dd, *J* = 1.3, 17.1 Hz, 1H), 6.19 (dd, *J* = 10.3, 17.0 Hz, 1H), 5.69 (dd, *J* = 1.3, 10.3 Hz, 1H), 4.17 (s, 2H), 3.95–3.35 (m, 8H), 2.40 (s, 3H)^13^C NMR (150 MHz, CDCl_3_) *δ* 170.7, 166.7, 165.4, 141.8, 137.8, 137.1, 135.4, 130.2, 129.7, 129.1, 128.8, 127.0, 126.9, 125.6, 125.5, 47.3, 42.1, 41.3, 21.1.

HRMS (ESI-QTOF) *m*/*z* (M + Na)^+^ calcd for C_23_H_25_N_3_O_3_Na: 414.1794, found: 414.1796.

#### *N*-2-[4-[((2′-Methyl-4-biphenyl)carbonyl)-1-piperazinyl]-2-oxoethyl]propenamide (21g)

The product was obtained following *general procedure D* using compound 11g (150 mg, 0.318 mmol) and Pd/C (30 mg) in dry MeOH and stirred for 2 h. Then dry DCM, acryloyl chloride (32 μL, 0.350 mmol), and DIPEA (166 μL, 0.954 mmol) added at room temperature. Work up C was used. The product was isolated following silica column chromatography (5% MeOH/DCM) to give a colourless oil. A white powder was obtained by dissolving the oil in a minimal amount of DCM and precipitating the product *via* hexane addition and solvent evaporation (55.0 mg, 44%).

^1^H NMR (600 MHz, CDCl_3_) *δ* 7.47 (d, *J* = 8.3 Hz, 2H), 7.40 (d, *J* = 8.3 Hz, 2H), 7.30–7.25 (m, 3H), 7.23–7.20 (m, 1H), 6.66 (br s, 1H), 6.33 (dd, *J* = 17.0, 1.3 Hz, 1H), 6.20 (dd, *J* = 17.0, 10.3 Hz, 1H), 5.70 (dd, *J* = 10.3, 1.4 Hz, 1H), 4.19 (s, 1H), 3.91–3.42 (m, 8H), 2.28 (s, 3H). ^13^C NMR (150 MHz, CDCl_3_) 170.7, 166.7, 165.4, 144.2, 135.2, 133.2, 130.5, 130.2, 129.6, 129.5, 127.8, 127.1, 127.0, 125.9, 41.3, 20.4.

HRMS (ESI-QTOF) *m*/*z* (M + Na)^+^ calcd for C_23_H_25_N_3_O_3_Na: 414.1794, found: 414.1791.

#### *N*-2-[4-[((3′-Methyl-4-biphenyl)carbonyl)-1-piperazinyl]-2-oxoethyl]propenamide (21h)

The product was obtained following *general procedure D* using compound 11h (150 mg, 0.318 mmol) and Pd/C (30 mg) in dry MeOH and stirred for 2 h. Then dry DCM, acryloyl chloride (32 μL, 0.350 mmol), and DIPEA (166 μL, 0.954 mmol) added at room temperature. Work up C was used. The product was isolated following silica column chromatography (5% MeOH/DCM) to give a colourless oil. A white powder was obtained by dissolving the oil in a minimal amount of DCM and precipitating the product *via* hexane addition and solvent evaporation (64.0 mg, 51%).

^1^H NMR (600 MHz, CDCl_3_) *δ* 7.67 (d, *J* = 8.2 Hz, 2H), 7.51 (d, *J* = 8.3 Hz, 2H), 7.44–7.40 (m, 2H), 7.38 (t, *J* = 7.5 Hz, 1H), 7.23 (d, *J* = 7.3 Hz, 1H), 6.68 (br s, 1H), 6.35 (dd, *J* = 17.0, 1.2 Hz, 1H), 6.20 (dd, *J* = 17.0, 10.3 Hz, 1H), 5.72 (dd, *J* = 10.3, 1.3 Hz, 1H), 4.20 (s, 2H), 3.97–3.38 (m, 8H), 2.46 (s, 3H). ^13^C NMR (150 MHz, CDCl_3_) *δ* 170.6, 166.7, 165.4, 143.4, 140.0, 138.6, 133.4, 130.3, 128.9, 128.7, 127.9, 127.7, 127.4, 127.0, 124.3, 41.3, 21.5.

HRMS (ESI-QTOF) *m*/*z* (M + Na)^+^ calcd for C_23_H_25_N_3_O_3_Na: 414.1794, found: 414.1782.

#### *N*-2-[4-[((4′-Methyl-4-biphenyl)carbonyl)-1-piperazinyl]-2-oxoethyl]propenamide (21i)

The product was obtained following *general procedure D* using compound 11i (150 mg, 0.318 mmol) and Pd/C (30 mg) in dry MeOH and stirred for 2 h. Then dry DCM, acryloyl chloride (32 μL, 0.350 mmol), and DIPEA (166 μL, 0.954 mmol) added at room temperature. Work up C was used. The product was isolated following silica column chromatography (5% MeOH/DCM) to give a colourless oil. A white powder was obtained by dissolving the oil in a minimal amount of DCM and precipitating the product *via* hexane addition and solvent evaporation (28.4 mg, 23%).

^1^H NMR (400 MHz, CDCl_3_) *δ* 7.64 (d, *J* = 8.2 Hz, 2H), 7.52–7.46 (m, 4H), 7.28 (d, *J* = 8.1 Hz, 2H), 6.67 (br s, 1H), 6.33 (dd, *J* = 17.2, 1.5 Hz, 1H), 6.20 (dd, *J* = 16.9, 10.2 Hz, 1H), 5.70 (dd, *J* = 10.1, 1.5 Hz, 1H), 4.18 (s, 2H), 3.91–3.40 (m, 8H), 2.41 (s, 3H). ^13^C NMR (150 MHz, CDCl_3_) δ 170.6, 166.7, 165.4, 143.2, 137.9, 137.1, 133.2, 130.3, 129.7, 127.7, 127.2, 127.1, 127.0, 41.3, 21.1.

HRMS (ESI-QTOF) *m*/*z* (M + Na)^+^ calcd for C_23_H_25_N_3_O_3_Na: 414.1794, found: 414.1797.

#### *N*-2-[4-[((2-Benzylphenyl)carbonyl)-1-piperazinyl]-2-oxoethyl]propenamide (22a)

The product was obtained following *general procedure D* using compound 20a (150 mg, 0.318 mmol) and Pd/C (30 mg) in dry MeOH and stirred for 2 h. Then dry DCM, acryloyl chloride (32 μL, 0.350 mmol), and DIPEA (166 μL, 0.954 mmol) added at room temperature. Work up C was used. The product was isolated following silica column chromatography (5% MeOH/DCM) to give a colourless oil. A white powder was obtained by dissolving the oil in a minimal amount of DCM and precipitating the product *via* hexane addition and solvent evaporation (31.2 mg, 25%).

^1^H NMR (600 MHz, CDCl_3_) *δ* 7.42–7.34 (m, 2H), 7.30–7.26 (m, 1H), 7.25–7.22 (m, 2H), 7.18–7.09 (m, 4H), 6.64–6.58 (m, 1H), 6.31 (dd, *J* = 16.9, 8.2 Hz, 1H), 6.17 (dd, *J* = 17.0, 10.2 Hz, 1H), 5.68 (d, *J* = 10.4 Hz, 1H), 4.24 (t, *J* = 12.5 Hz, 1H), 4.17–4.08 (m, 1H), 4.02–3.87 (m, 2H), 3.74–3.57 (m, 2.5H), 3.52–3.36 (m, 1H), 3.52–3.36 (m, 1H), 3.34–3.24 (m, 1H), 2.82–2.72 (m, 0.5H), 2.57–2.45 (m, 1H), 2.40–2.32 (m, 0.5H). ^13^C NMR (150 MHz, CDCl_3_) *δ* 169.9, 169.9, 166.5, 166.4, 165.3, 140.3, 140.2, 138.7, 135.3, 135.2, 131.4, 131.3, 130.3, 130.2, 129.6, 129.5, 129.2, 129.2, 128.5, 128.4, 127.0, 127.0, 126.5, 126.5, 126.4, 126.4, 126.3, 46.2, 46.1, 43.9, 43.7, 41.5, 41.4, 41.2, 41.2, 40.9, 40.8, 39.1, 39.1.

HRMS (ESI-QTOF) *m*/*z* (M + Na)^+^ calcd for C_23_H_25_N_3_O_3_Na: 414.1794, found: 414.1813.

#### *N*-2-[4-[((2-Phenoxyphenyl)carbonyl)-1-piperazinyl]-2-oxoethyl]propenamide (22b)

The product was obtained following *general procedure D* using compound 20b (150 mg, 0.317 mmol) and Pd/C (30 mg) in dry MeOH (15 mL) for the hydrogenation, stirring for 2 h. Acryloyl chloride (28 μL, 0.349 mmol) and DIPEA (193 μL, 1.110 mmol) in DCM (20 mL) were used for the acrylamide formation, added at room temperature. Work up C was used. The product was isolated following silica column chromatography (3–6% MeOH/DCM manual gradient) and solvent evaporation to give a colourless oil. A white powder was obtained by dissolving the oil in a minimal amount of DCM and precipitating the product *via* hexane addition and solvent evaporation (41.4 mg, 33%).

^1^H NMR (600 MHz, CDCl_3_) *δ* 7.42–7.38 (m, 1H), 7.38–7.32 (m, 3H), 7.20–7.11 (m, 2H), 6.99 (d, *J* = 8.1 Hz, 2H), 6.91–6.87 (m, 1H), 6.66 (m, 1H), 6.31 (d, *J* = 17.2 Hz, 1H), 6.19 (dd, *J* = 17.1, 10.3 Hz, 1H), 5.68 (dd, *J* = 10.3, 1.3 Hz, 1H), 4.25–4.09 (m, 2H), 4.00–3.26 (m, 8H). ^13^C NMR (150 MHz, CDCl_3_) *δ* 167.3, 167.2, 166.6, 166.4, 165.3, 156.2, 153.1, 153.0, 131.1, 131.1, 130.3, 130.3, 130.1, 130.0, 129.0, 128.9, 127.1, 127.1, 127.0, 127.0, 124.1, 124.1, 123.9, 123.9, 119.0, 118.9, 118.2, 118.2, 46.7, 46.6, 44.7, 44.3, 42.3, 41.8, 41.5, 41.4, 41.3.

HRMS (ESI-QTOF) *m*/*z* (M + Na)^+^ calcd for C_22_H_23_N_3_O_4_Na: 416.1586, found: 416.1596.

#### *N*-2-[4-[(((2-Phenylamino)phenyl)carbonyl)-1-piperazinyl]-2-oxoethyl]propenamide (22c)

The product was obtained following *general procedure D* using compound 20c (100 mg, 0.212 mmol) and Pd/C (20 mg) in dry MeOH (10 mL) and DCM (10 mL) for the hydrogenation, stirring for 2 h. Acryloyl chloride (20 μL, 0.233 mmol) and DIPEA (110 μL, 0.636 mmol) in DCM (20 mL) were used for the acrylamide formation, added at room temperature. Work up C was used. The product was isolated following silica column chromatography (5% MeOH/DCM) and solvent evaporation to give a colourless oil. A white powder was obtained by dissolving the oil in a minimal amount of DCM and precipitating the product *via* hexane addition and solvent evaporation (25.0 mg, 30%).

^1^H NMR (600 MHz, CDCl_3_) *δ* 7.39 (d, *J* = 8.3 Hz, 1H), 7.31–7.26 (m, 3H), 7.17 (d, *J* = 7.5 Hz, 1H), 7.09 (d, *J* = 8.1 Hz, 1H), 6.98 (t, *J* = 7.4 Hz, 1H), 6.91 (t, *J* = 7.4 Hz, 1H), 6.66 (br s, 1H), 6.31 (d, *J* = 17.1 Hz, 1H), 6.18 (dd, *J* = 17.0, 10.1 Hz, 1H), 5.69 (d, *J* = 10.0 Hz, 1H), 4.15 (s, 2H), 3.79–3.59 (m, 6H), 3.49–3.40 (m, 2H). ^13^C NMR (150 MHz, CDCl_3_) *δ* 170.2, 166.7, 165.4, 142.6, 141.9, 131.0, 130.2, 129.4, 128.1, 127.1, 122.3, 121.9, 119.9, 118.9, 117.5, 44.6, 42.1, 41.3.

HRMS (ESI-QTOF) *m*/*z* (M + Na)^+^ calcd for C_22_H_24_N_4_O_3_Na: 415.1770, found: 415.1766.

#### *N*-2-[4-[((3-benzylphenyl)carbonyl)-1-piperazinyl]-2-oxoethyl]propenamide (22d)

The product was obtained following *general procedure D* using compound 20d (150 mg, 0.318 mmol) and Pd/C (30 mg) in dry MeOH and stirred for 2 h. Then dry DCM, acryloyl chloride (32 μL, 0.350 mmol), and DIPEA (166 μL, 0.954 mmol) added at room temperature. Work up C was used. The product was isolated following silica column chromatography (5% MeOH/DCM) to give a colourless oil. A white powder was obtained by dissolving the oil in a minimal amount of DCM and precipitating the product *via* hexane addition and solvent evaporation (56.8 mg, 46%).

^1^H NMR (600 MHz, CDCl_3_) *δ* 7.35 (t, *J* = 7.6 Hz, 1H), 7.32–7.28 (m, 3H), 7.25–7.22 (m, 2H), 7.22–7.16 (m, 3H), 6.65 (br s, 1H), 6.32 (dd, *J* = 17.0, 1.4 Hz, 1H), 6.19 (dd, *J* = 17.0, 10.3 Hz, 1H), 5.69 (dd, *J* = 10.3, 1.4 Hz, 1H), 4.15 (s, 2H), 4.01 (s, 2H), 3.90–3.17 (m, 8H). ^13^C NMR (150 MHz, CDCl_3_) *δ* 170.7, 166.7, 165.4, 142.0, 140.2, 134.9, 130.8, 130.2, 129.0, 128.8, 128.6, 127.6, 127.0, 126.4, 124.9, 47.1, 44.4, 41.7, 41.3.

HRMS (ESI-QTOF) *m*/*z* (M + Na)^+^ calcd for C_23_H_25_N_3_O_3_Na: 414.1794, found: 414.1803.

#### *N*-2-[4-[((3-Phenoxyphenyl)carbonyl)-1-piperazinyl]-2-oxoethyl]propenamide (22e)

The product was obtained following *general procedure D* using compound 20e (100 mg, 0.211 mmol) and Pd/C (20 mg) in dry MeOH (10 mL) and dry DCM (2 mL) for the hydrogenation, stirring for 2 h. Acryloyl chloride (20 μL, 0.232 mmol) and DIPEA (129 μL, 0.739 mmol) in DCM (10 mL) were used for the acrylamide formation, added at room temperature. Work up C was used. The product was isolated following silica column chromatography (5% MeOH/DCM) and solvent evaporation to give a colourless oil. A white powder was obtained by dissolving the oil in a minimal amount of DCM and precipitating the product *via* hexane addition and solvent evaporation (59.6 mg, 72%).

^1^H NMR (600 MHz, CDCl_3_) *δ* 7.41–7.34 (m, 3H), 7.16 (t, *J* = 7.4 Hz, 1H), 7.13–7.06 (m, 2H), 7.06–6.99 (m, 3H), 6.66 (br s, 1H), 6.32 (dd, *J* = 17.0, 1.6 Hz, 1H), 6.19 (dd, *J* = 17.1, 10.2 Hz, 1H), 5.69 (dd, *J* = 10.2, 1.3 Hz, 1H), 4.16 (s, 2H), 3.89–3.29 (m, 8H). ^13^C NMR (150 MHz, CDCl_3_) *δ* 169.9, 166.7, 165.4, 157.9, 156.2, 136.5, 130.3, 130.2, 130.0, 127.1, 124.1, 121.4, 120.1, 119.5, 116.8, 41.3.

HRMS (ESI-QTOF) *m*/*z* (M + Na)^+^ calcd for C_22_H_23_N_3_O_4_Na: 416.1586, found: 416.1571.

#### *N*-2-[4-[(((3-Phenylamino)phenyl)carbonyl)-1-piperazinyl]-2-oxoethyl]propenamide (22f)

The product was obtained following *general procedure D* using compound 20f (125 mg, 0.265 mmol) and Pd/C (20 mg) in dry MeOH (15 mL) for the hydrogenation, stirring for 2 h. Acryloyl chloride (20 μL, 0.291 mmol) and DIPEA (138 μL, 0.795 mmol) in DCM (20 mL) were used for the acrylamide formation, added at room temperature. Work up C was used. The product was isolated following silica column chromatography (5% MeOH/DCM) and solvent evaporation to give a colourless oil. A white powder was obtained by dissolving the oil in a minimal amount of DCM and precipitating the product *via* hexane addition and solvent evaporation (50.3 mg, 48%).

^1^H NMR (600 MHz, CDCl_3_) *δ* 7.33–7.27 (m, 2H), 7.12–7.08 (m, 3H), 7.07–7.05 (m, 1H), 7.00 (t, *J* = 7.4 Hz, 1H), 6.88 (d, *J* = 7.5 Hz, 1H), 6.66 (br s, 1H), 6.32 (dd, *J* = 17.0, 1.3 Hz, 1H), 6.19 (dd, *J* = 17.0, 10.3 Hz, 1H), 5.69 (dd, *J* = 10.3, 1.2 Hz, 1H), 4.17 (s, 2H), 3.90–3.33 (m, 8H). ^13^C NMR (150 MHz, CDCl_3_) *δ* 170.6, 166.7, 165.4, 144.1, 141.9, 136.1, 130.2, 129.8, 129.5, 127.0, 122.2, 119.1, 118.6, 118.5, 115.0, 41.3.

HRMS (ESI-QTOF) *m*/*z* (M + Na)^+^ calcd for C_22_H_24_N_4_O_3_Na: 415.1746, found: 415.1760.

#### *N*-2-[4-[((4-Benzylphenyl)carbonyl)-1-piperazinyl]-2-oxoethyl]propenamide (22g)

The product was obtained following *general procedure D* using compound 20g (150 mg, 0.318 mmol) and Pd/C (30 mg) in dry MeOH and stirred for 2 h. Then dry DCM, acryloyl chloride (32 μL, 0.350 mmol), and DIPEA (166 μL, 0.954 mmol) added at room temperature. Work up C was used. The product was isolated following silica column chromatography (5% MeOH/DCM) to give a colourless oil. A white powder was obtained by dissolving the oil in a minimal amount of DCM and precipitating the product *via* hexane addition and solvent evaporation (42.6 mg, 34%).

^1^H NMR (600 MHz, CDCl_3_) *δ* 7.34 (d, *J* = 8.0 Hz, 2H), 7.32–7.29 (m, 2H), 7.26–7.24 (m, 2H), 7.22 (t, *J* = 7.4 Hz, 1H), 7.19–7.17 (m, 2H), 6.68 (br s, 1H), 6.31 (dd, *J* = 17.0, 1.4 Hz, 1H), 6.19 (dd, *J* = 17.0, 10.2 Hz, 1H), 5.69 (dd, *J* = 10.2, 1.4 Hz, 1H), 4.16 (s, 2H), 4.01 (s, 2H), 3.85–3.36 (m, 8H). ^13^C NMR (150 MHz, CDCl_3_) *δ* 170.7, 166.7, 165.4, 143.7, 140.2, 132.6, 130.2, 129.2, 129.0, 128.6, 127.4, 127.0, 126.4, 44.4, 42.1, 41.8, 41.3.

HRMS (ESI-QTOF) *m*/*z* (M + Na)^+^ calcd for C_23_H_25_N_3_O_3_Na: 414.1794, found: 414.1780.

#### *N*-2-[4-[((4-Phenoxyphenyl)carbonyl)-1-piperazinyl]-2-oxoethyl]propenamide (22h)

The product was obtained following *general procedure D* using compound 20h (150 mg, 0.317 mmol) and Pd/C (30 mg) in dry MeOH (15 mL) for the hydrogenation, stirring for 2 h. Acryloyl chloride (28 μL, 0.349 mmol) and DIPEA (193 μL, 1.110 mmol) in DCM (20 mL) were used for the acrylamide formation, added at room temperature. Work up C was used. The product was isolated following silica column chromatography (3–6% MeOH/DCM manual gradient) and solvent evaporation to give a colourless oil. A white powder was obtained by dissolving the oil in a minimal amount of DCM and precipitating the product *via* hexane addition and solvent evaporation (92.2 mg, 69%).

^1^H NMR (400 MHz, CDCl_3_) *δ* 7.44–7.34 (m, 4H), 7.18 (t, *J* = 7.4 Hz, 1H), 7.09–6.99 (m, 4H), 6.66 (br s, 1H), 6.32 (dd, *J* = 17.0, 1.5 Hz, 1H), 6.19 (dd, *J* = 17.0, 10.1 Hz, 1H), 5.70 (dd, *J* = 10.2, 1.4 Hz, 1H), 4.22–4.14 (m, 2H), 3.83–3.40 (m, 8H). ^13^C NMR (150 MHz, CDCl_3_) *δ* 170.3, 166.7, 165.4, 159.5, 155.9, 130.3, 130.0, 129.3, 129.0, 127.0, 124.3, 119.8, 118.0, 44.5, 42.1, 41.3.

HRMS (ESI-QTOF) *m*/*z* (M + Na)^+^ calcd for C_22_H_23_N_3_O_4_Na: 416.1586, found: 416.1592.

#### *N*-2-[4-[(((4-Phenylamino)phenyl)carbonyl)-1-piperazinyl]-2-oxoethyl]propenamide (22i)

The product was obtained following *general procedure D* using compound 20i (150 mg, 0.317 mmol) and Pd/C (30 mg) in dry DCM (15 mL) for the hydrogenation, stirring for 2 h. Acryloyl chloride (31 μL, 0.349 mmol) and DIPEA (166 μL, 0.951 mmol) in DMF (20 mL) were used for the acrylamide formation, added at room temperature. Work up C was used. The product was isolated following silica column chromatography (3% MeOH/DCM) and solvent evaporation to give a colourless oil. A white powder was obtained by dissolving the oil in a minimal amount of EtOAc and precipitating the product *via* hexane addition and solvent evaporation (63.2 mg, 51%).

^1^H NMR (400 MHz, CDCl_3_) *δ* 7.37–7.29 (m, 4H), 7.15 (d, *J* = 7.9 Hz, 1H), 7.06–7.1 (m, 3H), 6.70 (br s, 1H), 6.32 (dd, *J* = 17.1, 1.5 Hz, 1H), 6.19 (dd, *J* = 17.0, 10.1, Hz, 1H), 5.69 (dd, *J* = 10.1, 1.5 Hz, 1H), 4.21–4.13 (m, 2H), 3.77–3.61 (m, 6H), 3.52–3.44 (m, 2H). ^13^C NMR (100 MHz, CDCl_3_) δ 170.9, 166.7, 165.4, 145.9, 141.3, 130.3, 129.5, 129.4, 127.0, 125.6, 122.7, 119.7, 155.5, 44.5, 42.2, 41.3.

HRMS (ESI-QTOF) *m*/*z* (M + Na)^+^ calcd for C_22_H_24_N_4_O_3_Na: 415.1746, found: 415.1754.

#### 3-Fluoroadamantane-1-carboxylic acid (23a)

31 (1.20 g, 6.65 mmol, 1.0 eq) was flushed with N_2_ and dissolved in MeOH (2.9 mL), THF (2.2 mL), and H_2_O (1.45 mL). NaOH (0.46 g, 11.5 mmol, 2.04 eq.) was added which turned the reaction yellow. The reaction stirred at room temperature overnight under N_2_. The organic solvents were removed under reduced pressure and 6 mL of water were added. The solution was acidified to pH 1 with 6 M HCl (white solid formed). The white solid was filtered and washed with cold water, then dried under vacuum (0.9577 g, 86%).

^1^H NMR (400 MHz, CDCl_3_) *δ* 2.36 (d, *J* = 4.3 Hz, 2H), 2.05 (d, *J* = 5.6 Hz, 2H), 1.93–1.81 (m, 8H), 1.61 (d, *J* = 3.0 Hz, 2H). ^13^C NMR (100 MHz, CDCl_3_) *δ* 181.8 (d, *J*_CF_ = 2.2 Hz), 91.2 (d, *J*_CF_ = 183.4 Hz), 44.8 (d, *J*_CF_ = 10.1 Hz), 43.5 (d, *J*_CF_ = 20.2 Hz), 41.9 (d, *J*_CF_ = 17.3 Hz), 37.5 (d, *J*_CF_ = 2.2 Hz), 34.9 (d, *J*_CF_ = 2.2 Hz), 30.9 (d, *J*_CF_ = 10.1 Hz). ^19^F NMR (282 MHz, CDCl_3_) *δ* −132.7.

HRMS (EI) *m*/*z* (M)^+^ calcd for C_11_H_15_FO_2_: 198.1056, found: 198.1056.

#### 3-Chloroadamantane-1-carboxylic acid (23b)

30 (0.500 g, 2.55 mmol, 1 eq.) was dissolved in 5 mL of HCl and was heated in a capped round bottom flask at 60 °C overnight. After cooling to 0 °C the mixture was filtered, and the precipitate was washed with cold water. The resulting white solid was dried under high vacuum overnight (0.4592, 84%).

^1^H NMR (400 MHz, CDCl_3_) *δ* 2.33–2.20 (m, 4H), 2.11 (s, 4H), 1.87 (d, *J* = 3.1 Hz, 4H), 1.72–1.61 (m, 2H). ^13^C NMR (100 MHz, CDCl_3_) *δ* 181.5, 66.9, 48.1, 46.7, 44.3, 37.1, 34.6, 31.0.

HRMS (EI) *m*/*z* (M)^+^ calcd for C_11_H_15_ClO_2_: 214.0761, found: 214.0768.

#### 3-Bromoadamantanecarboxylic acid (23c)

To a round bottom flask charged with a stir bar was added adamantanecarboxylic acid (1.25 g, 6.935 mmol, 1.0 eq.) to conc. HNO_3_ (1 mL) and cooled to 0 °C in an ice bath. Conc. H_2_SO_4_ (7.5 mL) was then added dropwise slowly, then the mixture was stirred for 2 h at 0 °C. 33% HBr/AcOH (15 mL) was then added dropwise slowly. The mixture was then heated to 90 °C with a condenser and stirred for 18 h. Upon completion, the mixture was cooled to room temperature, upon which a precipitate formed. Cold water was added to the flask and the solid product was filtered under vacuum to give a yellow solid, which was dissolved in EtOAc, dried over MgSO_4_ and evaporated to give the pure product as a white powder (1.618 g, 90%).

^1^H NMR (400 MHz, CDCl_3_) *δ* 2.49 (s, 2H), 2.36–2.26 (m, 4H), 2.25–2.19 (m, 2H), 1.95–1.88 (m, 4H), 1.76–1.65 (m, 2H). ^13^C NMR (100 MHz, CDCl_3_) *δ* 181.1, 63.1, 49.3, 48.0, 44.6, 36.9, 34.4, 31.6.

Characterization is consistent with literature data.^31^

#### 3-Phenyladamantanecarboxylic acid (23d)

The product was obtained by dissolving 3-bromoadamantylcarboxylic acid, 23c (100 mg, 0.356 mmol, 1.0 eq.), in anhydrous benzene (10 mL) in a round bottom flask equipped with a stir bar under N_2_ atmosphere. In a separate round bottom flask equipped with a stir bar, AlBr_3_ was added to anhydrous benzene (10 mL) under N_2_ but did not fully dissolve. After approx. 5 minutes, the solution of 23c was added to the AlBr_3_ solution dropwise slowly then left to stir overnight (approx. 16 h) at room temperature. Upon completion, the reaction mixture was dilute with EtOAc (50 mL) and washed in a separatory funnel with 1 M HCl (×3). The organic phase was then dried over MgSO_4_, filtered, and the solvent was evaporated to give the pure product as a white powder (82.0 mg, 90%).

^1^H NMR (600 MHz, CDCl_3_) *δ* 7.38–7.35 (m, 2H), 7.35–7.31 (m, 2H), 7.20 (t, *J* = 7.1 Hz, 1H), 2.28–2.23 (m, 2H), 2.07 (s, 2H), 2.00–1.87 (m, 8H), 1.75 (s, 2H). ^13^C NMR (150 MHz, CDCl_3_) *δ* 182.8, 149.8, 128.2, 125.9, 124.8, 43.8, 42.1, 41.6, 37.9, 36.4, 35.5, 28.6.

Characterization is consistent with literature data.^52^

#### Benzyl[2-(4-(3-fluoroadamantyl)carbonylpiperazin-1-yl)-2-oxoethyl]carbamate (24a)

The product was obtained following *general procedure C* using compound 23a (0.300 g, 1.51 mmol, 1.0 eq.), DCM (4 mL), and SOCl_2_ (0.220 mL, 3.03 mmol, 2.0 eq.). Then DCM (10 mL), DIPEA (0.526 mL, 3.02 mmol, 2.0 eq.), and benzyl *N*-[2-oxo-2-(piperazin-1-yl)ethyl]carbamate HCl (4) (0.568 g, 1.81 mmol, 1.2 eq.). Work-up B gave a white solid that was used without further purification (0.4499 g, 65%).

^1^H NMR (600 MHz, CDCl_3_) *δ* 7.38–7.29 (m, 5H), 5.74 (t, *J* = 4.5 Hz, 1H), 5.12 (s, 2H), 4.04 (d, *J* = 4.3 Hz, 2H), 3.73–3.66 (m, 4H), 3.65–3.60 (m, 2H), 3.43–3.37 (m, 2H), 2.38 (m, 2H), 2.10 (d, *J* = 6.1 Hz, 2H), 1.93–1.85 (m, 9H), 1.62 (d, *J* = 3.2 Hz, 2H). ^13^C NMR (151 MHz, CDCl_3_) *δ* 174.16 (d, *J*_CF_ = 1.9 Hz), 166.98, 156.37, 128.68, 128.31, 128.18, 92.4 (d, *J*_CF_ = 184.4 Hz), 67.15, 46.15 (d, *J*_CF_ = 9.7 Hz), 45.14 (d, *J*_CF_ = 87.4 Hz), 44.57, 44.41 (d, *J*_CF_ = 19.6 Hz), 42.52 (d, *J*_CF_ = 76.0 Hz), 41.95 (d, *J*_CF_ = 17.4 Hz), 37.78 (d, *J*_CF_ = 1.9 Hz), 34.96 (d, *J*_CF_ = 2.2 Hz), 31.14 (d, *J*_CF_ = 10.0 Hz). ^19^F NMR (376 MHz, CDCl_3_) *δ* −131.01.

HRMS (ESI-QTOF) *m*/*z* (M + H)^+^ calcd for C_25_H_33_FN_3_O_4_: 458.2455, found: 458.2477.

#### Benzyl[2-(4-(3-chloroadamantyl)carbonylpiperazin-1-yl)-2-oxoethyl]carbamate (24b)

The product was obtained following *general procedure C* using compound 23b (0.400 g, 1.86 mmol, 1.0 eq.), DCM (5 mL), and SOCl_2_ (0.270 mL, 3.73 mmol, 2.0 eq). Then DCM (12 mL), DIPEA (0.650 mL, 3.73 mmol, 2.0 eq.), and benzyl *N*-[2-oxo-2-(piperazin-1-yl)ethyl]carbamate HCl (4) (0.700 g, 2.23 mmol, 1.2 eq). Work-up B gave a white solid that was used without further purification (0.4645 g, 53%).

^1^H NMR (400 MHz, CDCl_3_) *δ* 7.39–7.29 (m, 5H), 5.73 (s, 1H), 5.13 (s, 2H), 4.04 (d, *J* = 4.4 Hz, 2H), 3.72–3.60 (m, 6H), 3.41 (t, *J* = 5.3 Hz, 2H), 2.34 (s, 2H), 2.28 (s, 2H), 2.17–2.07 (m, 4H), 1.94 (d, *J* = 3.4 Hz, 4H), 1.67 (d, *J* = 8.1 Hz, 2H). ^13^C NMR (100 MHz, CDCl_3_) *δ* 174.1, 167.0, 156.4, 136.5, 128.7, 128.3, 128.2, 67.4, 67.2, 48.9, 46.8, 45.7, 45.5, 42.8, 42.3, 37.50, 34.7, 31.4.

HRMS (ESI-QTOF) *m*/*z* (M + H)^+^ calcd for C_25_H_33_ClN_3_O_4_: 474.2160, found: 474.2156.

#### Benzyl[2-(4-(3-bromoadamantyl)carbonylpiperazin-1-yl)-2-oxoethyl]carbamate (24c)

The product was obtained following *general procedure C* using compound 23c (200 mg, 0.722 mmol, 1.0 eq.), DCM (2 mL), and SOCl_2_ (0.112 mL, 1.444 mmol, 2.0 eq.). Then DCM (5 mL), DIPEA (0.250 mL, 1.444 mmol, 2.0 eq.), and benzyl *N*-[2-oxo-2-(piperazin-1-yl)ethyl]carbamate HCl (4) (0.280 g, 0.866 mmol, 1.2 eq.). Work up A was used, and the product was isolated following flash silica column chromatography (3–5% MeOH/DCM manual gradient) to give the product as a white solid (134.3 mg, 33%).

^1^H NMR (600 MHz, CDCl_3_) *δ* 7.38–7.29 (m, 5H), 5.73 (br s, 1H), 5.13 (s, 2H), 4.06–3.97 (m, 6H), 3.44–3.32 (m, 2H), 2.55 (s, 2H), 2.33 (m, 4H), 2.25 (m, 2H), 2.02–1.95 (m, 4H), 1.76–1.67 (m, 2H). ^13^C NMR (150 MHz, CDCl_3_) *δ* 173.8, 166.8, 156.2, 136.3, 128.5, 136.3, 128.5, 128.2, 128.0, 67.0, 63.7, 50.1, 48.1, 46.0, 45.3, 44.7, 44.4, 42.6, 42.1, 37.3, 34.5, 31.9.

HRMS (ESI-QTOF) *m*/*z* (M + Na)^+^ calcd for C_25_H_32_BrN_3_O_4_Na: 542.1456, found: 542.1453.

#### Benzyl[2-(4-(3-phenyladamantyl)carbonylpiperazin-1-yl)-2-oxoethyl]carbamate (24d)

The product was obtained following *general procedure C* using compound 23d (0.140 g, 0.546 mmol, 1.0 eq.), DCM (2 mL), and SOCl_2_ (0.100 mL, 1.092 mmol, 2.0 eq.). Then DCM (5 mL), DIPEA (0.250 mL, 1.444 mmol, 2.0 eq.), and Benzyl *N*-[2-oxo-2-(piperazin-1-yl)ethyl]carbamate HCl (4) (0.280 g, 0.866 mmol, 1.2 eq.). Work up A was used, and the product was isolated following flash silica column chromatography (3–5% MeOH/DCM manual gradient) to give the product as a white solid (117.1 mg, 42%).

^1^H NMR (400 MHz, CDCl_3_) *δ* 7.39–7.29 (m, 9H), 7.24–7.18 (m, 1H), 5.75 (br s, 1H), 5.12 (s, 2H), 4.07–3.96 (m, 2H), 3.75–3.66 (m, 4H), 3.65–3.59 (m, 2H), 3.44–3.32 (m, 2H), 2.31–2.25 (m, 2H), 2.11 (s, 2H), 2.07–1.97 (m, 4H), 1.97–1.89 (m, 4H), 1.79–1.72 (m, 2H). ^13^C NMR (100 MHz, CDCl_3_) *δ* 175.7, 166.8, 156.2, 149.7, 136.3, 128.5, 128.3, 128.2, 128.0, 126.0, 124.7, 67.0, 45.3, 44.7, 44.5, 42.9, 42.6, 42.2, 42.1, 38.3, 36.8, 35.7, 29.0.

HRMS (ESI-QTOF) *m*/*z* (M + Na)^+^ calcd for C_31_H_37_N_3_O_4_Na: 538.2682, found: 538.2673.

#### *N*-2-[4-[(3-Fluoroadamantanecarbonyl)-1-piperazinyl]-2-oxoethyl]propenamide (25a)

The product was obtained following *general procedure D* using compound 24a (0.424 g, 0.93 mmol, 1.0 eq.) and Pd/C (0.0424 g) in dry MeOH (10 mL) and stirred for 16 h. Then dry DCM (10 mL), DIPEA (0.484 mL, 2.78 mmol, 3.0 eq), and acryloyl chloride (0.082 mL, 1.02 mmol, 1.1 eq) added at 0 °C and warmed to room temperature. Work up B was used, and the product was isolated following flash silica column chromatography (10% MeOH/DCM) to give a white solid (0.0953 g, 27%).

^1^H NMR (400 MHz, CDCl_3_) *δ* 6.79 (s, 1H), 6.29 (dd, *J* = 17.1, 1.8 Hz, 1H), 6.18 (dd, *J* = 17.0, 10.0 Hz, 1H), 5.66 (dd, *J* = 10.1, 1.8 Hz, 1H), 4.14 (d, *J* = 4.0 Hz, 2H), 3.71–3.60 (m, 6H), 3.48–3.39 (m, 2H), 2.36 (t, *J* = 5.4 Hz, 2H), 2.08 (d, *J* = 6.1 Hz, 2H), 1.87 (s, 8H), 1.60 (d, *J* = 3.1 Hz, 2H). ^13^C NMR (100 MHz, CDCl_3_) *δ* 174.1 (d, *J*_CF_ = 1.8 Hz), 166.9, 165.5, 130.4, 127.1, 92.4 (d, *J*_CF_ = 183.0 Hz), 46.1 (d, *J*_CF_ = 9.4 Hz), 45.1 (d, *J*_CF_ = 37.5 Hz), 44.6, 44.4 (d, *J*_CF_ = 19.5 Hz), 42.2, 41.9 (d, *J*_CF_ = 17.3 Hz), 41.3, 37.7 (d, *J*_CF_ = 2.2 Hz), 34.9 (d, *J*_CF_ = 2.2 Hz), 31.1 (d, *J*_CF_ = 10.1 Hz). ^19^F NMR (376 MHz, CDCl_3_) δ −130.75.

HRMS (ESI-QTOF) *m*/*z* (M + Na)^+^ calcd for C_20_H_28_FN_3_O_3_Na: 400.2012, found: 400.1997.

#### *N*-2-[4-[(3-Chloroadamantanecarbonyl)-1-piperazinyl]-2-oxoethyl]propenamide (25b)

The product was obtained following *general procedure D* using compound 24b (0.4406 g, 0.93 mmol, 1.0 eq.) and Pd/C (0.0441 g) in dry MeOH (10 mL) and stirred for 16 h. Then dry DCM (10 mL), DIPEA (0.486 mL, 2.79 mmol, 3.0 eq), and acryloyl chloride (0.0824 mL, 1.02 mmol, 1.1 eq) added at 0 °C and warmed to room temperature. Work up B was used, and the product was isolated following flash silica column chromatography (10% MeOH/DCM) to give a white solid (0.1821 g, 50%).

^1^H NMR (400 MHz, CDCl_3_) *δ* 6.82 (s, 1H), 6.28 (dd, *J* = 17.1, 1.8 Hz, 1H), 6.18 (dd, *J* = 17.0, 10.0 Hz, 1H), 5.65 (dd, *J* = 10.0, 1.9 Hz, 1H), 4.14 (d, *J* = 4.1 Hz, 2H), 3.71–3.58 (m, 6H), 3.44 (dd, *J* = 6.8, 3.6 Hz, 2H), 2.30 (s, 2H), 2.28–2.22 (m, 2H), 2.14–2.04 (m, 4H), 1.90 (s, 4H), 1.70–1.57 (m, 2H). ^13^C NMR (100 MHz, CDCl_3_) *δ* 174.0, 166.9, 165.5, 130.4, 127.0, 67.4, 48.8, 46.7, 45.6, 45.2, 44.9, 44.6, 42.2, 41.3, 37.4, 34.6, 31.3.

HRMS (ESI-QTOF) *m*/*z* (M + Na)^+^ calcd for C_20_H_28_ClN_3_O_3_Na: 416.1717, found: 416.1739.

#### *N*-2-[4-[(3-Bromoadamantanecarbonyl)-1-piperazinyl]-2-oxoethyl]propenamide (25c)

The product was obtained following *general procedure D* using compound 24c (100 mg, 0.193 mmol) and Pd/C (20 mg) in dry MeOH and stirred for 2 h. Then dry DCM, acryloyl chloride (18 μL, 0.212 mmol), and DIPEA (100 μL, 0.579 mmol) added at room temperature. Work up C was used. The product was isolated following silica column chromatography (5% MeOH/DCM) to give a colourless oil. A white powder was obtained by dissolving the oil in a minimal amount of DCM and precipitating the product *via* hexane addition and solvent evaporation (44.5 mg, 53%).

^1^H NMR (400 MHz, CDCl_3_) *δ* 6.67 (br s, 1H), 6.32 (dd, *J* = 17.0, 1.5 Hz, 1H), 6.19 (dd, *J* = 17.0, 10.2 Hz, 1H), 5.69 (dd, *J* = 10.1, 1.5 Hz, 1H), 4.18–4.13 (m, 2H), 3.75–3.41 (m, 6H), 3.49–3.41 (m, 2H), 2.55 (s, 2H), 2.39–2.27 (m, 4H), 2.28–2.21 (m, 2H), 2.04–1.93 (m, 4H), 1.79–1.66 (m, 2H). ^13^C NMR (100 MHz, CDCl_3_) δ 173.8, 166.7, 165.4, 130.2, 127.0, 63.7, 50.1, 48.1, 46.0, 45.2, 44.8, 44.5, 42.1, 41.3, 37.3, 34.5, 31.9.

HRMS (ESI-QTOF) *m*/*z* (M + Na)^+^ calcd for C_20_H_28_BrN_3_O_3_Na: 462.1191, found: 462.1194.

#### *N*-2-[4-[(3-Phenyladamantanecarbonyl)-1-piperazinyl]-2-oxoethyl]propenamide (25d)

The product was obtained following *general procedure D* using compound 24d (100 mg, 0.194 mmol) and Pd/C (20 mg) in dry MeOH and stirred for 2 h. Then dry DCM (10 mL), acryloyl chloride (18 μL, 0.212 mmol), and DIPEA (100 μL, 0.579 mmol), added at room temperature. Work up C was used. The product was isolated following silica column chromatography (5% MeOH/DCM) to give a colourless oil. A white powder was obtained by dissolving the oil in a minimal amount of DCM and precipitating the product *via* hexane addition and solvent evaporation (53.0 mg, 63%).

^1^H NMR (400 MHz, CDCl_3_) *δ* 7.38–7.31 (m, 4H), 7.24–7.18 (m, 1H), 6.66 (br s, 1H), 6.32 (dd, *J* = 17.0, 1.5 Hz, 1H), 6.18 (dd, *J* = 17.0, 10.2 Hz, 1H), 5.69 (dd, *J* = 10.2, 1.5 Hz, 1H), 4.18–4.12 (m, 2H), 3.78–3.69 (m, 4H), 3.68–3.61 (m, 2H), 3.47–3.40 (m, 2H), 2.32–2.25 (m, 2H), 2.12 (s, 2H), 2.08–1.98 (m, 4H), 1.97–1.89 (m, 4H), 1.79–1.72 (m, 2H). ^13^C NMR (100 MHz, CDCl_3_) *δ* 175.7, 166.7, 165.3, 149.7, 130.3, 128.3, 127.0, 126.0, 124.7, 45.2, 44.8, 44.5, 42.9, 42.2, 42.1, 41.3, 38.4, 36.9, 35.7, 29.0.

HRMS (ESI-QTOF) *m*/*z* (M + Na)^+^ calcd for C_26_H_33_N_3_O_3_Na: 458.2420, found: 458.2397.

#### 3-Iodoadamantane-1-carboxylic acid (26)

Compound 30 (0.500 g, 2.55 mmol, 1 eq.) was dissolved in 5 mL of HI and was heated in a capped round bottom flask at 60 °C overnight. After cooling to 0 °C the mixture was filtered, and the precipitate was washed with cold water to remove all the colour from the product. The resulting white solid was dried under high vacuum overnight (0.6856 g, 88%).

^1^H NMR (300 MHz, CDCl_3_) *δ* 2.75 (s, 2H), 2.57 (m, 4H), 2.06 (m, 2H), 1.99 (s, 4H), 1.79 (m, 2H). ^13^C NMR (100 MHz, CDCl_3_) *δ* 181.8, 52.4, 51.2, 45.7, 44.8, 37.0, 34.6, 32.1.

Characterization is consistent with literature data.^30^

#### *Tert*-butyl *N*-[4-(3-iodoadamantane-1-carbonyl)piperazine]-1-carboxylate (27)

Compound 26 (0.300 g, 0.98 mmol, 1.0 eq.) was dissolved in 3 mL of DCM and SOCl_2_ (0.142 mL, 1.96 mmol, 2.0 eq.) was added dropwise, the reaction turned pink. The reaction was stirred at room temperature for 4 hours. The solvent was evaporated under vacuum and then co-evaporated with DCM (×3) to obtain a pink solid. The solid was dissolved in DCM (5 mL) and DIPEA (0.341 mL, 1.96 mmol, 2.0 eq.) was added, the reaction turned from pink to yellow. Boc-piperazine (0.220 g, 1.18 mmol, 1.2 eq.) was added and the reaction was stirred overnight. The DCM was removed under reduced pressure and the resulting yellow oil was dissolved in EtOAc. The organic layer was washed with 10% AcOH (×3), brine (×2), 2 M NaOH (×3), and brine (×2), dried with MgSO_4_, and the solvent was removed. A white solid was obtained which was dried under vacuum (0.360 g, 74%).

^1^H NMR (400 MHz, CDCl_3_) *δ* 3.55 (s, 4H), 3.35 (s, 4H), 2.73 (s, 2H), 2.50 (s 4H), 1.99 (m, 6H), 1.71 (s, 2 H), 1.22 (s, 9H). ^13^C NMR (100 MHz, CDCl_3_) *δ* 173.3, 154.5, 80.1, 53.0, 51.1, 47.1, 46.0, 45.1, 37.6, 32.3, 28.3.

HRMS (ESI-QTOF) *m*/*z* (M + H)^+^ calcd for C_20_H_32_IN_2_O_3_: 475.1458, found: 475.1481.

#### *Tert*-butyl *N*-[2-[4-(3-iodoadamantane-1-carbonyl)piperazin-1-yl]-2-oxoethyl]carbamate (28)

Compound 27 (0.35 g, 0.73 mmol, 1.0 eq.) was dissolved in 4 mL of DCM. 4 mL of 4.0 M HCl/dioxane were added. A white solid formed. The reaction was stirred at room temperature for 4 hours (1 mL of both DCM and HCl/dioxane were added after 2 and 3 hours). The solvent was removed under reduced pressure and DCM was added and evaporated ×3. The resulting white solid was washed with cold ether and decanted ×3. The solid was dried under vacuum (0.28 g, 93%). SM (0.28 g, 0.68 mmol, 1.0 eq.) was dissolved in DCM (5 mL). TCFH (0.210 g, 0.75 mmol, 1.1 eq.) and NMI (1.90 mL, 2.39 mmol, 3.5 eq.) (solid formed) were added followed by Boc–Gly–OH (0.131 g, 0.75 mmol, 1.1 eq.) (solid dissolved). The reaction was stirred at room temperature for 20 hours. The solvent was removed and the resulting yellow oil was dissolved in EtOAc. The organic layer was washed with 10% AcOH (×3), brine (1×), saturated NaHCO_3_ (×3), and brine (×3), dried with MgSO_4_, and the solvent was removed to give a white solid. The solid was dried under vacuum (0.32 g, 89%).

^1^H NMR (400 MHz, CDCl_3_) *δ* 5.45 (bs, 1H), 3.97 (s, 2H), 3.66 (m, 6H), 3.41 (s, 2H), 2.82 (s, 2H), 2.58 (m, 4H), 2.05 (m, 6H), 1.79 (s, 2H), 1.45 (s, 9H). ^13^C NMR (100 MHz, CDCl_3_) *δ* 173.7, 167.5, 155.9, 80.0, 53.1, 51.3, 46.6, 46.2, 45.5, 44.9, 44.5, 42.4, 42.2, 37.4, 34.7, 32.5, 28.5.

HRMS (ESI-QTOF) *m*/*z* (M + H)^+^ calcd for C_22_H_35_IN_3_O_4_: 532.1672, found: 532.1665.

#### *N*-2-[4-[(3-Iodoadamantanecarbonyl)-1-piperazinyl]-2-oxoethyl]propenamide (29)

Compound 28 (0.300 g, 0.56 mmol, 1.0 eq.) was dissolved in 1 mL of DCM. 4 mL of TFA were added. The reaction stirred at room temperature for 1 h. The DCM was removed under reduced pressure and the TFA was co-evaporated with DCM (5×). The product was rinsed ×3 with cold ether to give a white solid. The solid dissolved in 10 mL of dry DCM and a N_2_ balloon was added. DIPEA (0.335 mL, 1.93 mmol, 3.4 eq) was added and the reaction was cooled to 0 °C. Acryloyl chloride (0.057 mL, 0.71 mmol, 1.3 eq.) was added dropwise. The reaction was stirred at 0 °C, until the ice melted and was then stirred at room temperature for a total of 2 hours. The DCM was removed, and the resulting yellow oil was dissolved in EtOAc. The organic layer was washed with 10% AcOH (×3), brine (1×), saturated NaHCO_3_ (×3), and brine (×3), dried with MgSO_4_ and the solvent was removed to give a yellow solid. The solid was washed with hexanes (×3) and dried under vacuum (0.19 g, 70%).

^1^H NMR (400 MHz, CDCl_3_) *δ* 6.83 (s, 1H), 6.28 (dd, *J* = 17.0, 1.9 Hz, 1H), 6.18 (dd, *J* = 17.0, 10.0 Hz, 1H), 5.65 (dd, *J* = 9.9, 1.8 Hz, 1H), 4.14 (d, *J* = 4.1 Hz, 2H), 3.71–3.58 (m, 6H), 3.47–3.37 (m, 2H), 2.77 (s, 2H), 2.55 (q, *J* = 9.4 Hz, 4H), 2.10–1.94 (m, 6H), 1.76 (t, *J* = 2.9 Hz, 2H). ^13^C NMR (100 MHz, CDCl_3_) *δ* 173.6, 166.9, 165.5, 130.3, 127.0, 53.0, 51.2, 46.65, 46.1, 45.2, 44.9, 44.6, 42.2, 41.3, 37.4, 34.6, 32.4.

HRMS (ESI-QTOF) *m*/*z* (M + Na)^+^ calcd for C_20_H_28_IN_3_O_3_Na: 508.1073, found: 508.1101.

#### Methyl 3-hydroxyadamantane-1-carboxylate (31)

Compound 30 (1.5 g, 7.64 mmol, 1.0 eq.) was dissolved in 83 mL of dry MeOH. SOCl_2_ (0.665 mL, 9.17 mmol, 1.2 eq.) was added dropwise. The reaction stirred for 5 hours at room temperature. The methanol was removed *in vacuo* and the resulting oil was dissolved in EtOAc. The organic layer was washed with saturated NaHCO_3_ (×3) and brine (×2), dried with MgSO_4_, and the solvent was removed. The resulting yellow oil was dried under high vacuum overnight (1.4614 g, 91%).

^1^H NMR (400 MHz, CDCl_3_) *δ* 3.64 (s, 3H), 2.23 (s, 2H), 1.82–1.57 (m, 11H), 1.57 (s, 2H). ^13^C NMR (100 MHz, CDCl_3_) *δ* 177.0, 68.4, 51.9, 46.4, 44.4, 44.2, 37.6, 35.1, 30.3.

HRMS (EI) *m*/*z* (M)^+^ calcd for C_12_H_18_O_3_: 210.1256, found: 210.1253.

#### Adamantane-1-sulfinyl chloride (34)

DCM (5 mL) was added to a flame-dried flask. Thionyl chloride (0.323 mL, 4.45 mmol, 2.0 eq.) was added and cooled to −20 °C. AlCl_3_ (0.294 g, 2.20 mmol, 1.0 eq.) was added in 3 batches. The reaction was stirred at −20 °C for 20 minutes. Adamantane (33) (0.300 g, 2.20 mmol, 3.0 eq.) was added all at once and the reaction turned yellow. The reaction was stirred at −20 °C for 3 h. Ice water was added dropwise to the reaction to quench it and then the reaction was warmed to room temperature. The aqueous layer was separated and the organic layer was washed with water (×2), dried with MgSO_4_, and the solvent was removed under reduced pressure to give a white solid (0.3135, 65%).

^1^H NMR (400 MHz, CDCl_3_) δ 2.26–2.19 (m, 2H), 1.94 (d, *J* = 1.6 Hz, 4H), 1.86–1.81 (m, 1H), 1.80–1.62 (m, 8H). ^13^C NMR (101 MHz, CDCl_3_) *δ* 66.2, 37.7, 36.0, 34.5, 28.8, 28.3.

HRMS (EI) *m*/*z* (M)^+^ calcd for C_10_H_15_OS: 183.0838, found: 183.0821 (Fragmentation of Cl).

#### *Tert*-butyl *N*-[4-((adamantan-1-yl)sulfinyl)piperazine]-1-carboxylate (35)

Compound 34 (1.59 g, 7.27 mmol, 1.0 eq.) was dissolved in 35 mL of DCM. Triethylamine (2.03 mL, 14.54 mmol, 2.0 eq.) was added followed by DMAP (0.0879 g, 0.72 mmol, 0.1 eq.). Boc-piperazine (1.62 g, 8.72 mmol, 1.2 eq.) was added and the reaction stirred at room temperature overnight. In the morning, the reaction was not done, so another 0.0442 g (0.05 eq.) of DMAP were added. The reaction stirred for another 5 hours then diluted with DCM and the organic layer was washed with 1 M HCl (×2) and brine (1×). The organic layer was dried with MgSO_4_ and the solvent was evaporated. The resulting whiteish yellow solid was purified using column chromatography in 100% EtOAc to collect a white solid (1.43 g, 53%).

^1^H NMR (400 MHz, CDCl_3_) *δ* 3.43 (t, *J* = 5.4 Hz, 4H), 3.04 (m, 4H), 2.08 (m, 3H), 1.80 (m, 3H), 1.66 (m, 9H), 1.41 (s, 9H). ^13^C NMR (100 MHz, CDCl_3_) *δ* 154.7, 80.1, 60.4, 47.3, 36.4, 35.3, 28.6, 28.4.

HRMS (ESI-QTOF) *m*/*z* (M + H)^+^ calcd for C_19_H_33_N_2_O_3_S: 369.2212, found: 369.2217.

#### *Tert*-butyl *N*-[4-((adamantan-1-yl)sulfonyl)piperazine]-1-carboxylate (36)

Compound 35 (0.1065 g, 0.29 mmol, 1.0 eq.) was dissolved in DCM (4 mL) and *m*-CPBA (0.0623 g, 0.361 mmol, 1.25 eq.) was added. The reaction stirred at room temperature for 3 hours. The reaction was diluted with DCM and washed with saturated NaHCO_3_ (×2), dried with MgSO_4_, and the solvent was removed to give a white solid. The white solid was dried under vacuum (0.0974 g, 87%).

^1^H NMR (400 MHz, CDCl_3_) *δ* 3.58–3.20 (m, 8H), 2.13 (s, 2H), 1.98 (s, 6H), 1.70 (q, *J* = 12.8 Hz, 6H), 1.46 (s, 9H). ^13^C NMR (100 MHz, CDCl_3_) *δ* 154.7, 80.4, 62.9, 47.4, 36.2, 36.0, 28.5, 28.3.

HRMS (ESI-QTOF) *m*/*z* (M + Na)^+^ calcd for C_19_H_32_N_2_O_4_SNa: 407.1980, found: 407.1983.

#### *Tert*-butyl *N*-[(2-(4-((adamantan-1-yl)sulfonyl)piperazin-1-yl)-2-oxoethyl)]carbamate (37)

Compound 36 (0.4205 g, 1.09 mmol, 1.0 eq.) was dissolved in DCM (5 mL). TFA (5 mL) was added. The reaction was stirred at room temperature for 30 minutes. The solvent was removed under reduced pressure and the TFA was co-evaporated 5× with DCM. The resulting white solid was washed 3× with cold ether and dried under vacuum (0.4262 g, 97.8%). The resulting product (0.4262 g, 1.07 mmol, 1.0 eq.) was dissolved in DCM (5 mL) and ACN (5 mL). TCFH (0.331 g, 1.18 mmol, 1.1 eq.) and NMI (0.298 mL, 3.74 mmol, 3.5 eq.) were added which caused a solid to form. Boc–Gly (0.206 g, 1.18 mmol, 1.1 eq.) was added which caused the solid to dissolve. The reaction was stirred overnight at room temperature. The solvent was removed, and the resulting product was dissolved in EtOAc. The organic layer was washed with 10% AcOH (×3), brine (×1), saturated NaHCO_3_ (×3), and brine (×3), dried with MgSO_4_, and the solvent was removed to give a white solid (0.4123, 87%).

^1^H NMR (600 MHz, CDCl_3_) *δ* 3.96 (s, 2H), 3.43 (m, 6H), 2.14 (s, 3H), 1.97 (d, *J* = 3.3 Hz, 6H), 1.73 (dt, *J* = 12.7, 3.0 Hz, 3H), 1.67 (d, *J* = 10.5 Hz, 3H), 1.45 (s, 9H). ^13^C NMR (151 MHz, CDCl_3_) δ 167.2, 155.9, 80.0, 63.0, 47.4, 47.3, 45.5, 43.0, 42.4, 36.2, 35.9, 28.5, 28.2.

HRMS (ESI-QTOF) *m*/*z* (M + Na)^+^ calcd for C_21_H_35_N_3_O_5_SNa: 464.2195, found: 464.2189.

#### *N*-[2-(4-((Adamantan-1-yl)sulfonyl)piperazin-1-yl)-2-oxoethyl]propenamide (38)

Compound 37 (0.3981 g, 0.90 mmol, 1.0 eq.) was dissolved in 5 mL of DCM. 5 mL of TFA were added. The reaction stirred at room temperature for 1 h. The DCM was removed under reduced pressure and the TFA was co-evaporated with DCM (×5). The product was rinsed ×3 with cold ether to give a white solid. The solid was dissolved in 10 mL of dry DCM and a N_2_ balloon was added. DIPEA (0.4860 mL, 2.79 mmol, 3.0 eq.) was added and the reaction was cooled to 0 °C. Acryloyl chloride (0.082 mL, 1.02 mmol, 1.1 eq.) was added dropwise. The reaction was stirred at 0 °C, until the ice melted and was then stirred at room temperature for a total of 2 hours. The DCM was removed, and the resulting yellow oil was dissolved in EtOAc. The organic layer was washed with 10% AcOH (×3), brine (×2), saturated NaHCO_3_ (×3), and brine (×3), dried with MgSO_4_ and the solvent was removed to give a white solid. The solid was washed with hexanes (×3) and dried under vacuum (0.087 g, 24%).

^1^H NMR (400 MHz, CDCl_3_) *δ* 6.75 (s, 1H), 6.30 (dd, *J* = 17.0, 1.8 Hz, 1H), 6.19 (dd, *J* = 17.0, 10.0 Hz, 1H), 5.67 (dd, *J* = 10.1, 1.7 Hz, 1H), 4.13 (d, *J* = 4.0 Hz, 2H), 3.92–3.11 (m, 8H), 2.13 (s, 3H), 1.96 (d, *J* = 3.4 Hz, 6H), 1.76–1.60 (m, 6H). ^13^C NMR (101 MHz, CDCl_3_) *δ* 166.7, 165.5, 130.4, 127.1, 63.0, 47.3, 45.6, 43.0, 41.4, 36.1, 35.9, 28.2.

HRMS (ESI-QTOF) *m*/*z* (M + Na)^+^ calcd for C_19_H_29_N_3_O_4_SNa: 418.1776, found: 418.1761.

### TG2 enzyme expression and purification protocol

The enzyme was expressed and purified according to a modified procedure developed by Han *et al.*^53^ A swab of Novagen Rosetta 2 (DE3) *E. coli* cells, which had been transformed with a plasmid containing the C-terminal His-tag TG2 gene (purchased from Addgene, plasmid #100719), was added to two test tubes with 5 mL of sterile Terrific Broth (TB) medium and 5 μL each of kanamycin (50 mg mL^−1^) and chloramphenicol (34 mg mL^−1^) stock solutions. The test tubes were incubated at 37 °C for 16 h while shaking at 250 RPM. The contents were added to 500 mL of TB medium and 500 μL each of kanamycin (50 mg mL^−1^) and chloramphenicol (34 mg mL^−1^) stock solutions in a single 1 L Erlenmeyer flask. The mixture was incubated at 37 °C while shaking at 250 RPM until the optical density at 600 nm measure between 0.5 and 0.7 (approximately 2.75 h). Protein expression was induced by addition of isopropyl β-d-thiogalactopyranoside, which was added for a final concentration of 1 mM in the flask, then incubated at 18 °C for 20 h while shaking at 250 RPM. The induced mixture was centrifuged at 4000 RPM for 20 min at 4 °C, and the supernatant liquid was removed to leave a pellet which was resuspended with 12 mL of lysis buffer (50 mM Na_3_PO_4_, 400 mM NaCl, 5 mM imidazole, 0.5% v/v Triton-X100, pH 7.5). The suspension was homogenized using an Avestin EmulsiFlex-B15 homogenizer at 60 PSI. The lysate was then centrifuged at 19000 RPM for 1 h at 4 °C and the resulting supernatant liquid was filtered through 0.45 μm PFTE sterile filters and incubated with 0.5 mL nickel-NTA agarose resin for 1 h at 4 °C. The expressed protein was purified by washing the resin in a gravity-flow column twice with 10 mL lysis buffer then twice with 10 mL wash buffer (50 mM Na_3_PO_4_, 500 mM NaCl, 60 mM imidazole, pH 7.5). Purified enzyme was eluted twice with 10 mL of elution buffer (50 mM HEPES, 100 mM NaCl, 300 mM imidazole, 10% v/v glycerol, pH 7.0), aliquoted, and flash frozen in an acetone/dry ice bath.

### Inhibition assay protocol

For each inhibitor molecule, the following assay was performed in triplicate. A 125 μL volume of buffer (111.11 mM MOPS, 15.56 mM CaCl_2_, pH 6.9) was added to seven 1.5 mL microcentrifuge tubes designated to contain five different inhibitor concentrations, a positive control (no inhibitor), and a negative control (no inhibitor or enzyme). Separately, inhibitor stock solutions were prepared by dissolving the inhibitor in DMSO and water such that the DMSO concentration in the final assay mixture (250 μL total) does not exceed 6%. All inhibitors tested were soluble in their concentrated stock solutions at concentrations up to 500 μM. Inhibitor stock solutions were added to five of the microcentrifuge tubes to reach the target inhibitor concentrations. Water was then added to each microcentrifuge tube to reach a volume of 245 μL. 5 μL of 5.56 mM AL5 in DMSO was added last to each microcentrifuge tube, which was vortexed to fully mix the solution. An aliquot of 180 μL of each solution was transferred from their respective microcentrifuge tubes into separate wells of a 96-well transparent polystyrene microplate. The assay was initiated by the addition of 20 μL of 50 mU mL^−1^ C-terminal His-tag TG2 using a multichannel pipette for a final enzyme concentration of 5 mU mL^−1^ in each well (except the negative control) simultaneously. The hydrolysis of AL5 by TG2 to produce *p*-nitrophenolate was monitored immediately after enzyme addition using a BioTek Synergy H4 Hybrid Multi-Mode microplate reader (20 min at 25 °C) to measure absorbance at 405 nm against time. GraphPad Prism's one-phase association analysis was used to obtain the *k*_obs_ inactivation rate constants for each inhibitor concentration and positive control. The *K*_I_ and *k*_inact_ values for the inhibitor were obtained by plotting *k*_obs_ against [*I*]/*α* (where *α* = (1 + ([AL5]/*K*_M_)), [AL5] = 100 μM, *K*_M_ = 10 μM), and fitting this plot, using GraphPad Prism, by non-linear regression to the following hyperbolic equation:
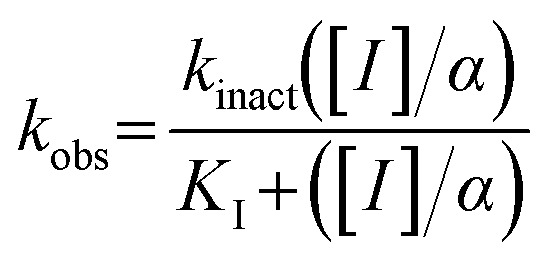


#### Pharmacokinetics testing

All hepatocyte stability assays and PAMPA studies were completed at Pharmaron, Inc. Human hepatocytes (20-donor, pooled) were provided by BiolVT (Cat. No. X008000, Lot. No. YIT).

## Author contributions

Conceptualization: J. W. K. and P. N.; funding acquisition: J. W. K.; supervision: J. W. K.; investigation and methodology: D. A. W., S. T., P. N., C. B. and T. S.; writing – original draft: D. A. W., S. T. and J. W. K.; writing – reviewing and editing: all authors.

## Conflicts of interest

There are no conflicts of interest to declare.

## Supplementary Material

MD-017-D5MD00815H-s001

## Data Availability

The data supporting this article have been included as part of the SI. Supplementary information (SI): including kinetic data, HPLC traces and NMR spectra. See DOI: https://doi.org/10.1039/d5md00815h.
