# Supplementary material for: Structure–activity relationships of hydrophobic small molecule irreversible inhibitors of tissue transglutaminase
Source: RSC Med Chem. 2025 Nov 11;17(1):422–51. doi: 10.1039/d5md00815h (PMC12631574; doi:10.1039/d5md00815h)

Supplementary Information (SI) for RSC Medicinal Chemistry.
This journal is © The Royal Society of Chemistry 2025

Supporting Information for:

Structure-Activity Relationships of Hydrophobic Small Molecule Irreversible Inhibitors of Tissue Transglutaminase

Daniel A. Wallace, Sarah Tribe, Pauline Navals, Christina Bi, Tarasha Sharma,

Jeffrey W. Keillor*

Department of Chemistry and Biomolecular Sciences,

University of Ottawa, Ottawa, ON Canada N6H 1N5

Contents

Kinetics Data and Fitting S2

HPLC Purity Chromatograms S18

^{1}H and ^{13}C NMR Spectra S35

Kinetics Data and Fitting

Compound 6a

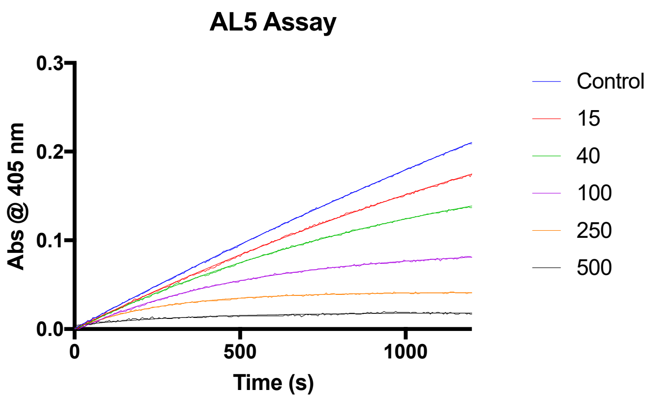

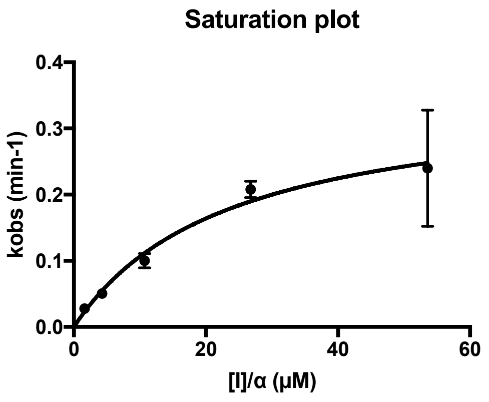

$k_{inact}/K_I = (15.2 \pm 4.6) \times 10^3\ M^{-1}min^{-1}$

$k_{inact} = 0.356 \pm 0.043\ min^{-1}$

$K_I = 23.5 \pm 6.5\ \mu M$

Compound 6b

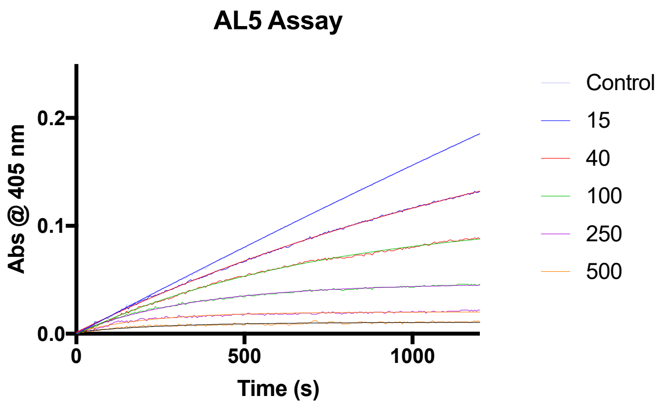

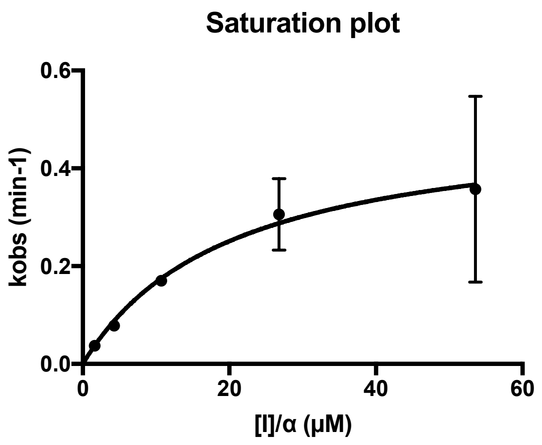

$k_{inact}/K_I = (25.0 \pm 4.7) \times 10^3\ M^{-1}min^{-1}$

$k_{inact} = 0.505 \pm 0.036\ min^{-1}$

$K_I = 20.2 \pm 3.5\ \mu M$

Compound 6c

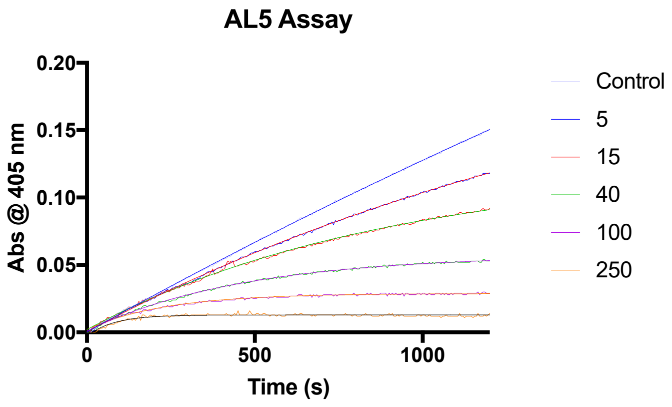

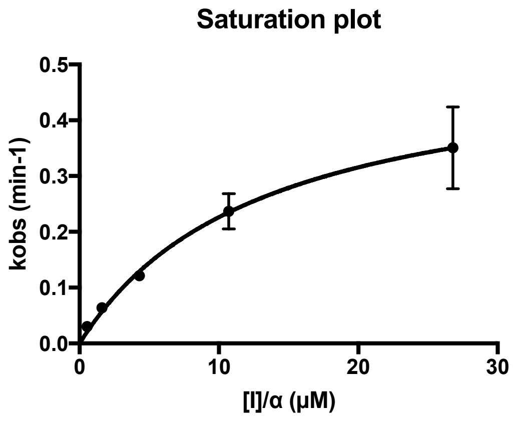

$k_{inact}/K_I = (40.0 \pm 5.3) \times 10^3\ M^{-1}min^{-1}$

$k_{inact} = 0.521 \pm 0.029\ min^{-1}$

$K_I = 13.0 \pm 1.6\ \mu M$

Compound 6d

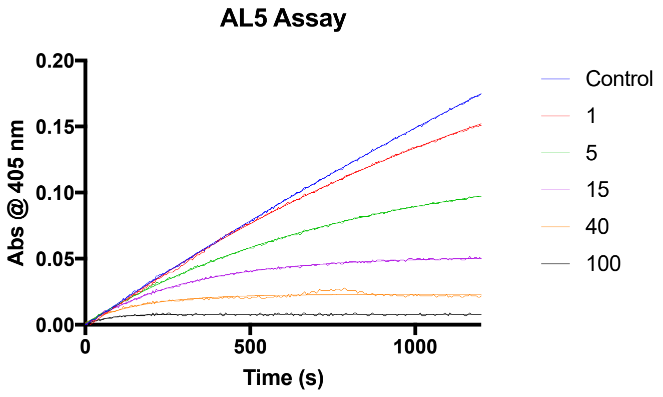

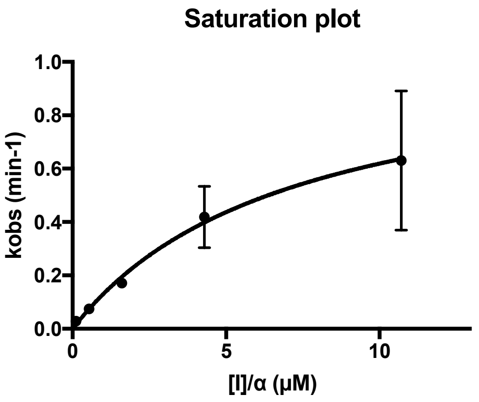

$k_{inact}/K_I = (149 \pm 31) \times 10^3\ M^{-1}min^{-1}$

$k_{inact} = 1.06 \pm 0.10\ min^{-1}$

$K_I = 7.14 \pm 1.34\ \mu M$

Compound 6e

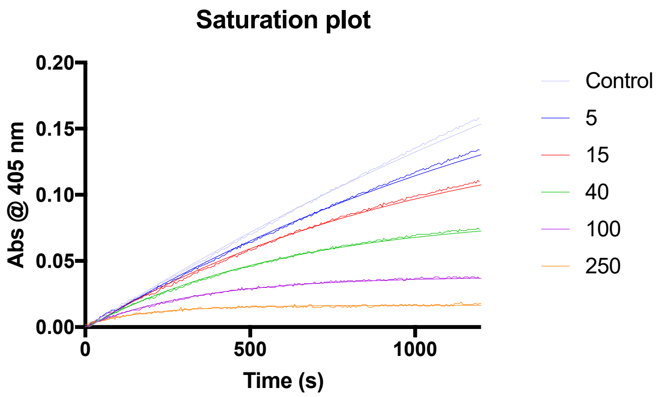

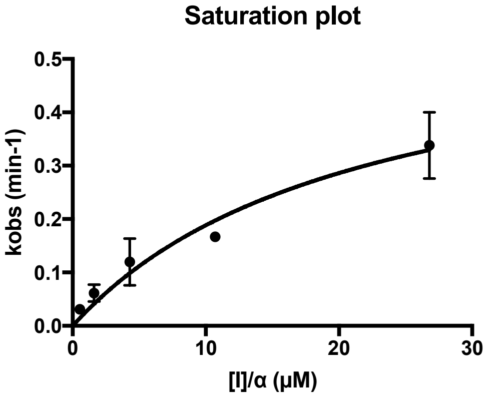

$k_{inact}/K_I = (27.6 \pm 16.1) \times 10^3\ M^{-1}min^{-1}$

$k_{inact} = 0.594 \pm 0.166\ min^{-1}$

$K_I = 21.5 \pm 11.0\ \mu M$

Compound 6f

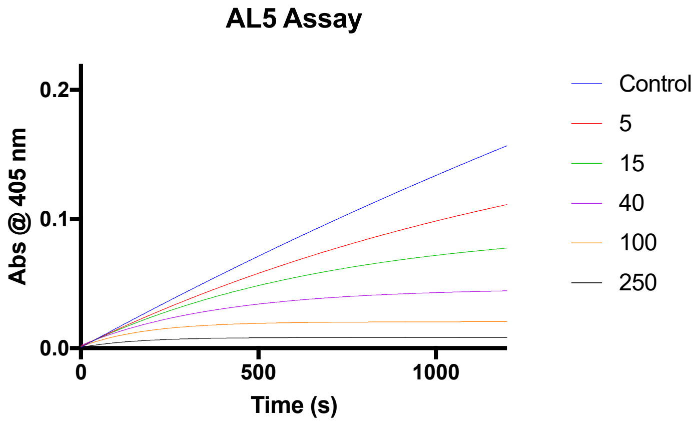

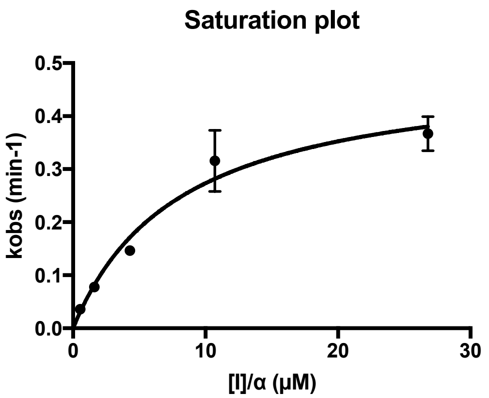

$k_{inact}/K_I = (61.1 \pm 20.0) \times 10^3\ M^{-1}min^{-1}$

$k_{inact} = 0.496 \pm 0.059\ min^{-1}$

$K_I = 8.12 \pm 2.47\ \mu M$

Compound 7a

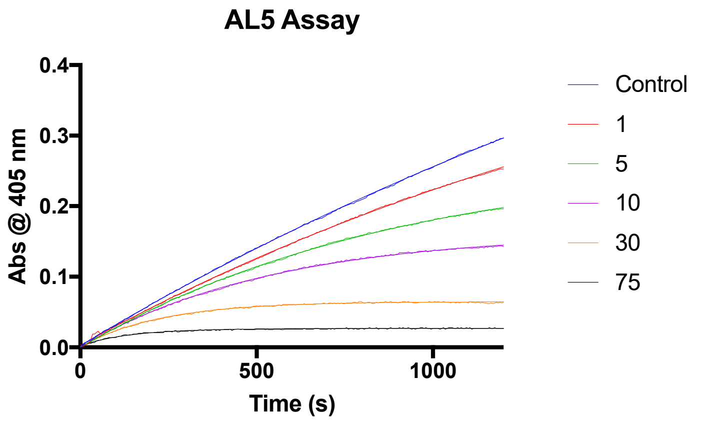

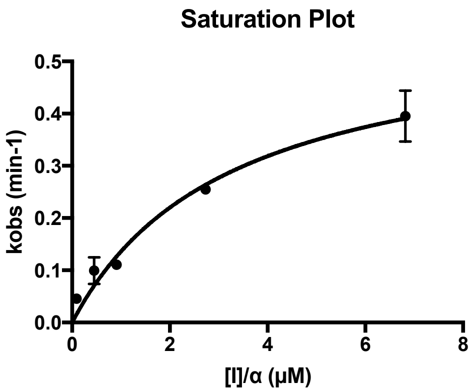

$k_{inact}/K_I = (178.0 \pm 68.6) \times 10^3\ M^{-1}min^{-1}$

$k_{inact} = 0.575 \pm 0.092\ min^{-1}$

$K_I = 3.23 \pm 1.14\ \mu M$

Compound 7b

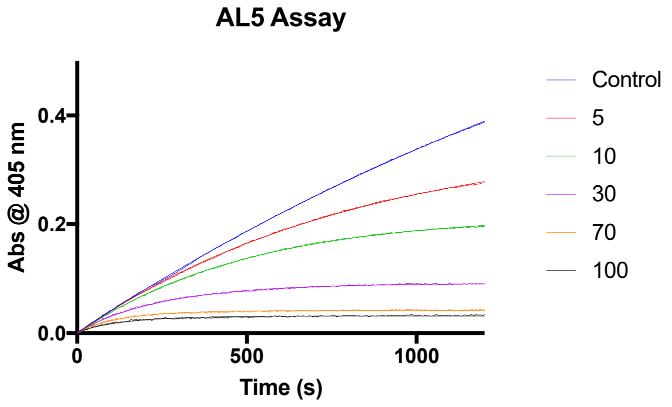

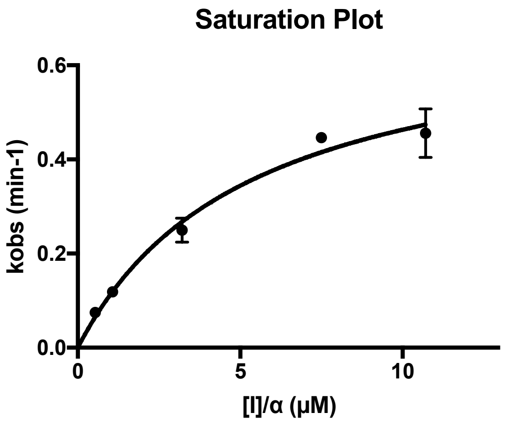

$k_{inact}/K_I = (133.9 \pm 38.4) \times 10^3\ M^{-1}min^{-1}$

$k_{inact} = 0.708 \pm 0.082\ min^{-1}$

$K_I = 5.29 \pm 1.39\ \mu M$

Compound 7c

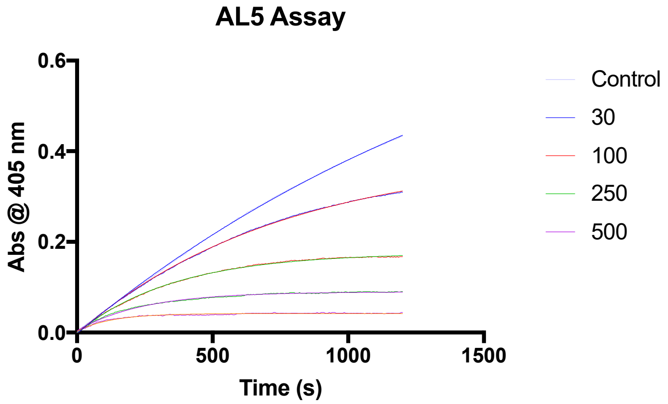

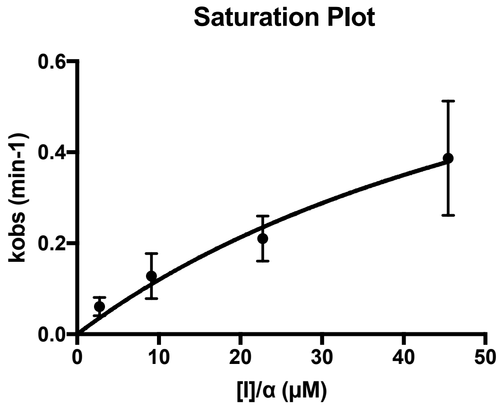

$k_{inact}/K_I = (17.2 \pm 15.5) \times 10^3\ M^{-1}min^{-1}$

$k_{inact} = 0.707 \pm 0.314\ min^{-1}$

$K_I = 41.1 \pm 32.3\ \mu M$

Compound 21a

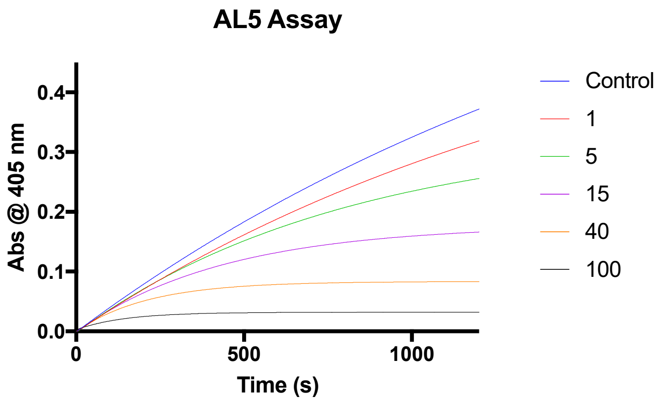

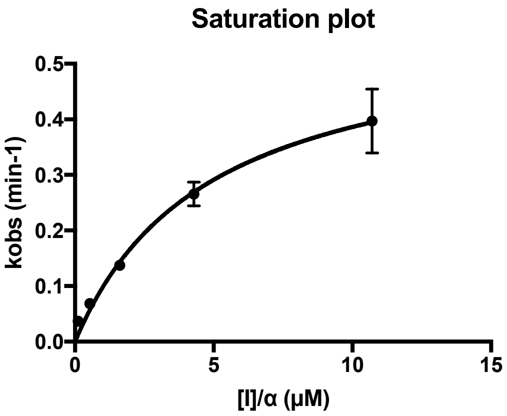

$k_{inact}/K_I = (118 \pm 27) \times 10^3\ M^{-1}min^{-1}$

$k_{inact} = 0.573 \pm 0.053\ min^{-1}$

$K_I = 4.85 \pm 1.02\ \mu M$

Compound 21b

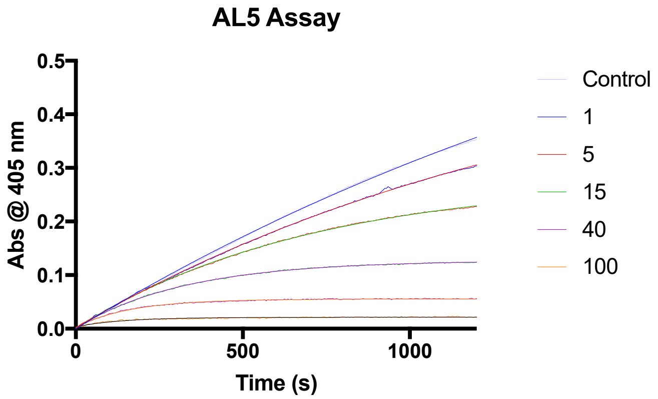

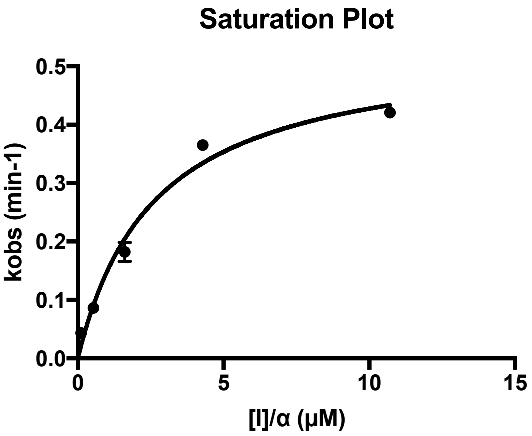

k_{inact}/K_I = (205 ± 60) × 10^3 $M^{-1}min^{-1}$

k_{inact} = 0.541 ± 0.054 min^{-1}

K_I = 2.64 ± 0.72 μM

Compound 21c

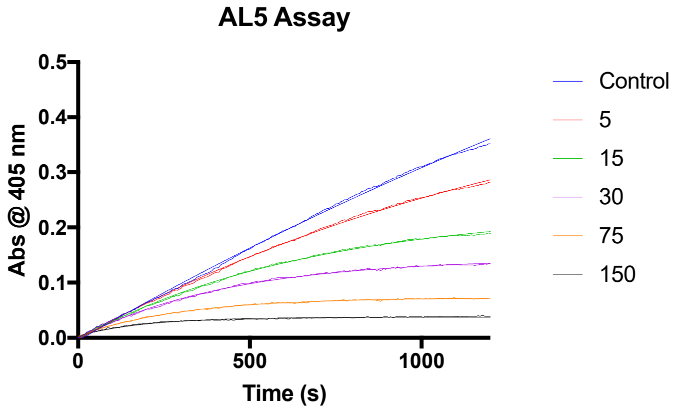

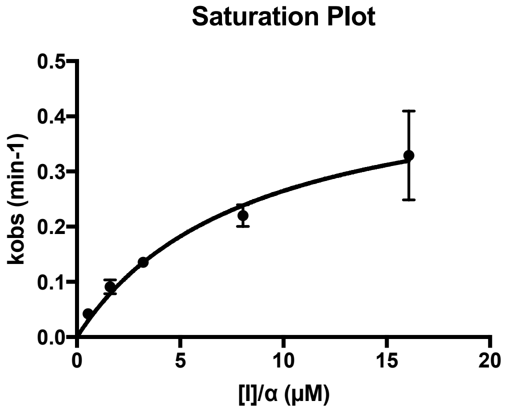

k_{inact}/K_I = (58.5 ± 16.2) × 10^3 $M^{-1}min^{-1}$

k_{inact} = 0.484 ± 0.057 min^{-1}

K_I = 8.28 ± 2.08 μM

Compound 21d

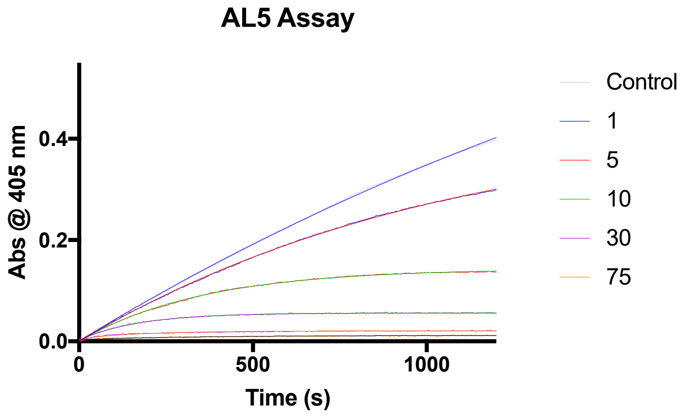

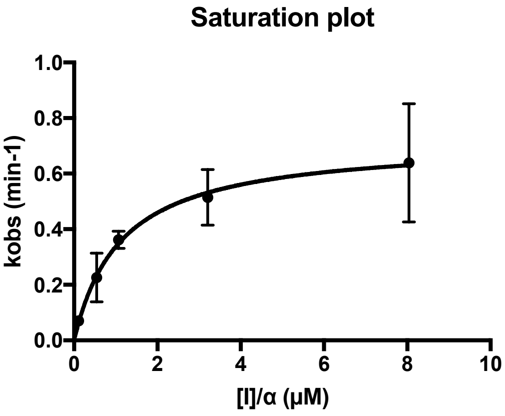

$k_{\mathrm{inact}}/K_{\mathrm{I}}$ = (636 ± 58) × 10^3 $M^{-1}min^{-1}$

k_{inact} = 0.719 ± 0.020 min^{-1}

K_{I} = 1.13 ± 0.099 μM

Compound 21e

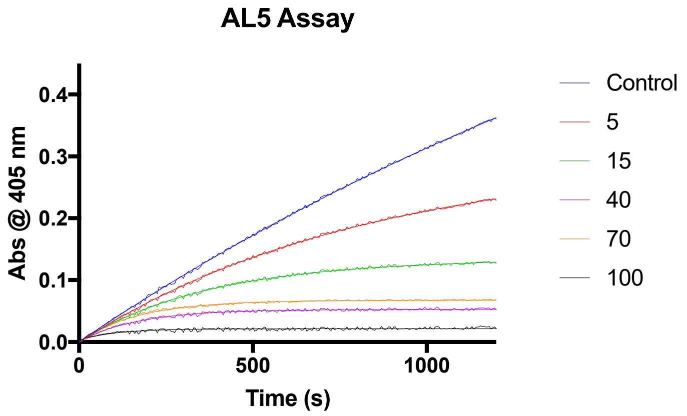

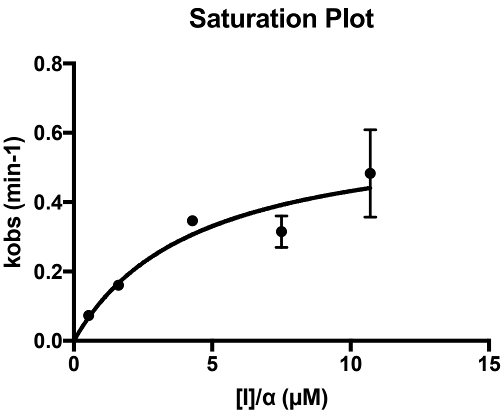

$k_{\mathrm{inact}}/K_{\mathrm{I}}$ = (141 ± 98) × 10^3 $M^{-1}min^{-1}$

k_{inact} = 0.623 ± 0.164 min^{-1}

K_{I} = 4.42 ± 2.83 μM

Compound 21f

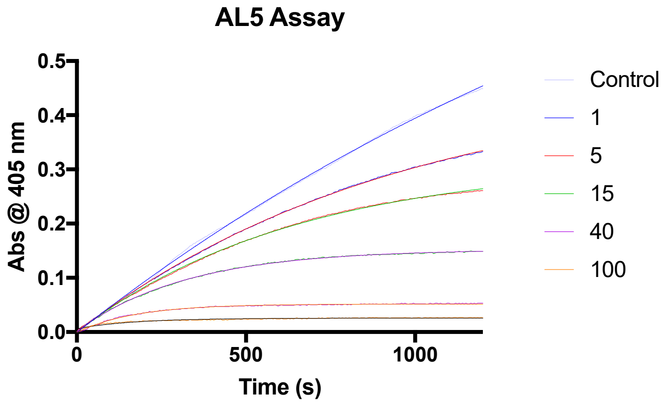

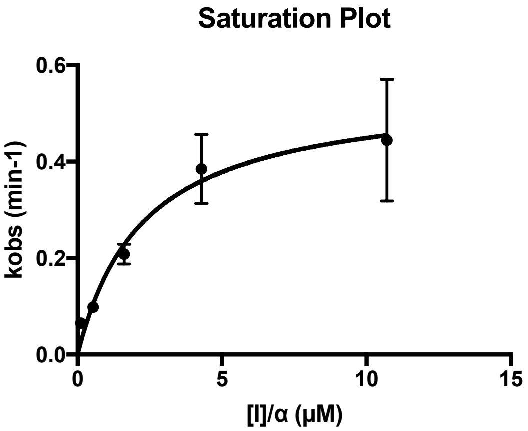

$k_{\text{inact}}/K_{\text{I}} = (241 \pm 74) \times 10^3\ \text{M}^{-1}\text{min}^{-1}$

$k_{\text{inact}} = 0.552 \pm 0.055\ \text{min}^{-1}$

$K_{\text{I}} = 2.29 \pm 0.66\ \mu\text{M}$

Compound 21g

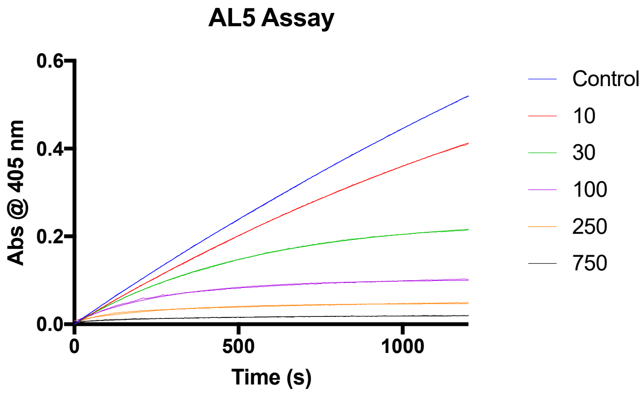

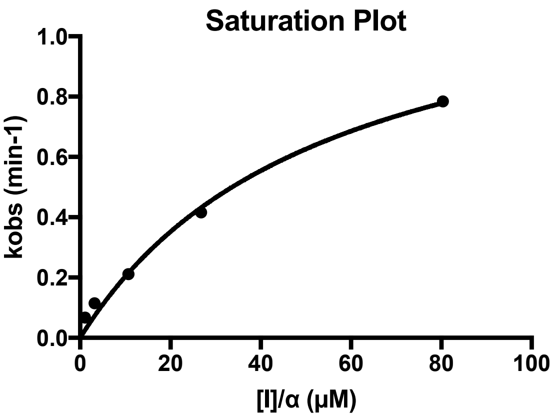

$k_{\text{inact}}/K_{\text{I}} = (24.1 \pm 7.3) \times 10^3\ \text{M}^{-1}\text{min}^{-1}$

$k_{\text{inact}} = 1.30 \pm 0.18\ \text{min}^{-1}$

$K_{\text{I}} = 54.1 \pm 14.6\ \mu\text{M}$

Compound 21h

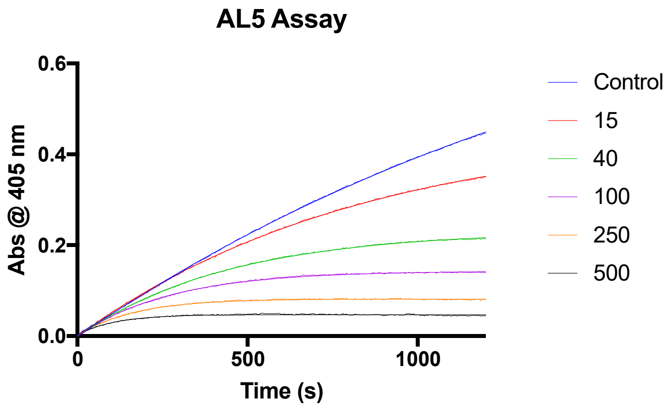

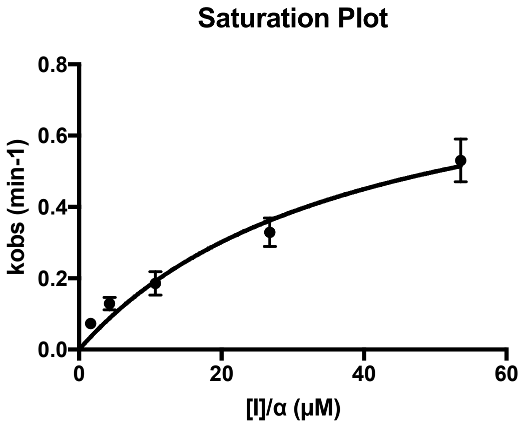

$k_{inact}/K_I = (22.8 \pm 11.7) \times 10^3\ M^{-1}min^{-1}$

$k_{inact} = 0.887 \pm 0.213\ min^{-1}$

$K_I = 38.9 \pm 17.7\ \mu M$

Compound 21i
Compound was too insoluble in assay conditions.

Compound 22a

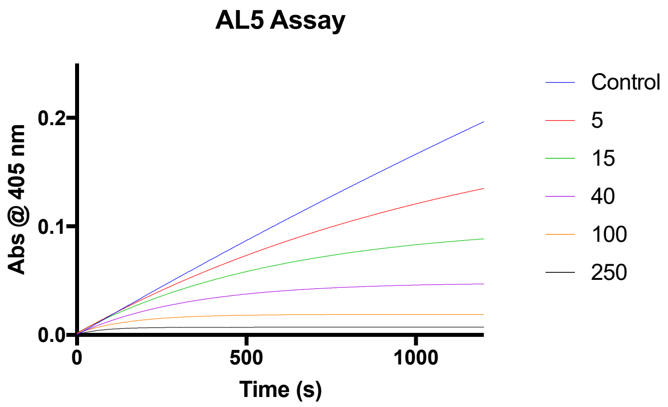

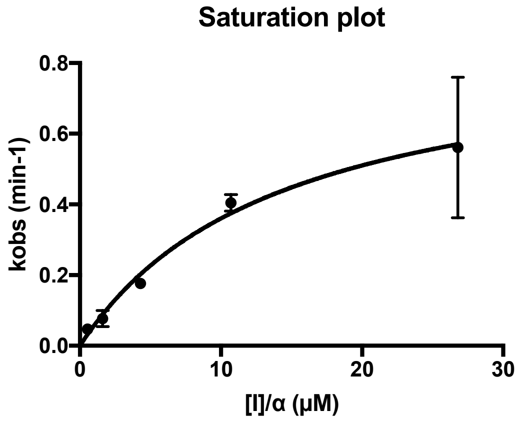

$k_{inact}/K_I = (61.3 \pm 15.9) \times 10^3\ M^{-1}min^{-1}$

$k_{inact} = 0.873 \pm 0.100\ min^{-1}$

$K_I = 14.2 \pm 3.3\ \mu M$

Compound 22b

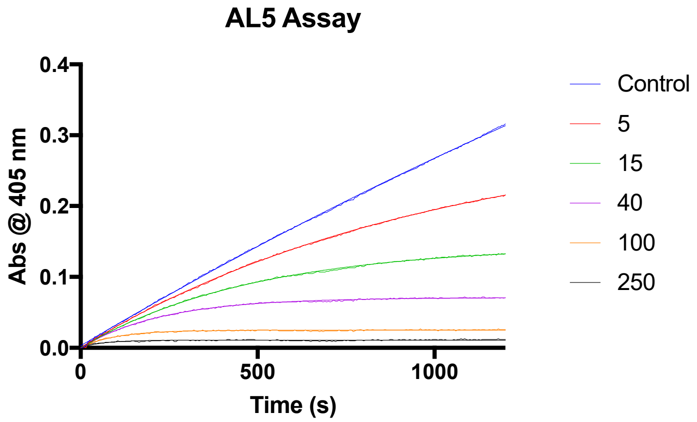

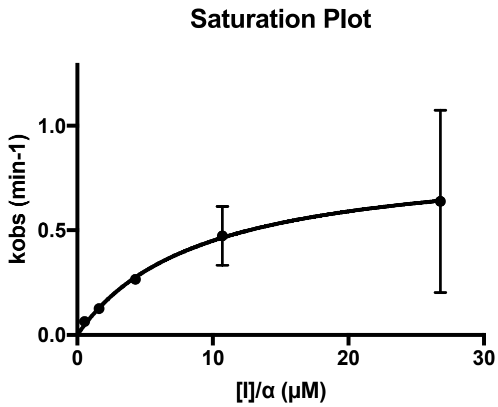

$k_{inact}/K_I = (94.8 \pm 9.0) \times 10^3\ M^{-1}min^{-1}$

$k_{inact} = 0.858 \pm 0.031\ min^{-1}$

$K_I = 9.06 \pm 0.80\ \mu M$

Compound 22c

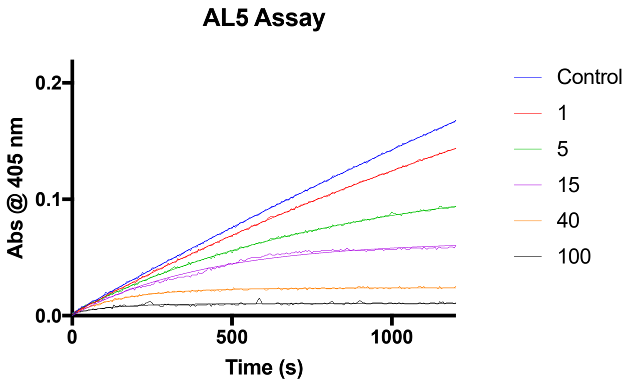

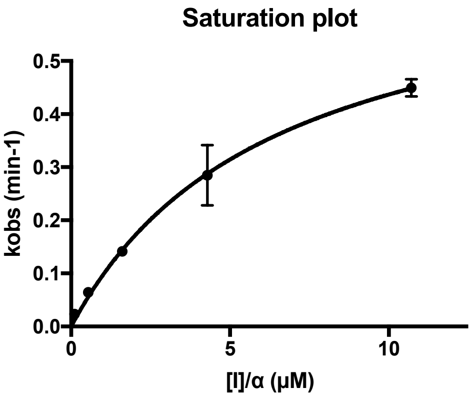

$k_{inact}/K_I = (112 \pm 14) \times 10^3\ M^{-1}min^{-1}$

$k_{inact} = 0.715 \pm 0.038\ min^{-1}$

$K_I = 6.38 \pm 0.70\ \mu M$

Compound 22d

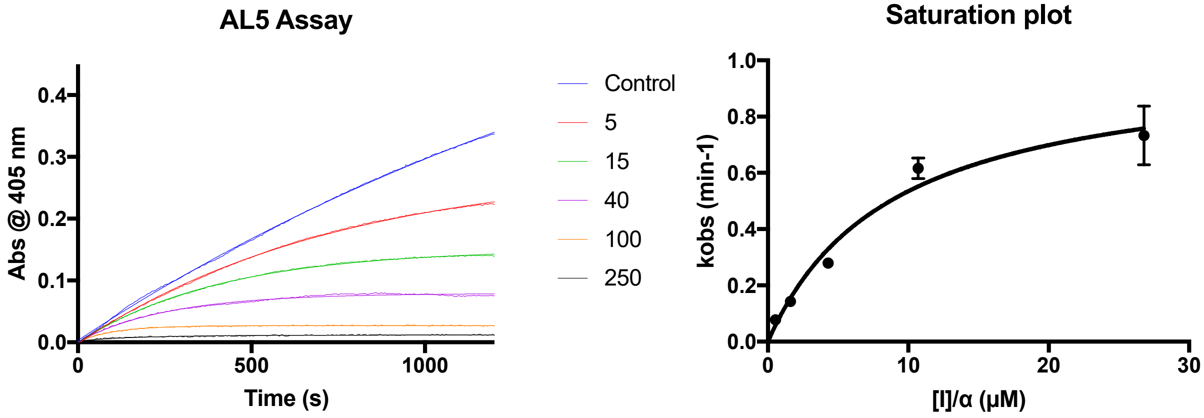

$k_{inact}/K_I = (114 \pm 38) \times 10^3\ M^{-1}min^{-1}$

$k_{inact} = 1.01 \pm 0.13\ min^{-1}$

$K_I = 8.84 \pm 2.72\ \mu M$

Compound 22e

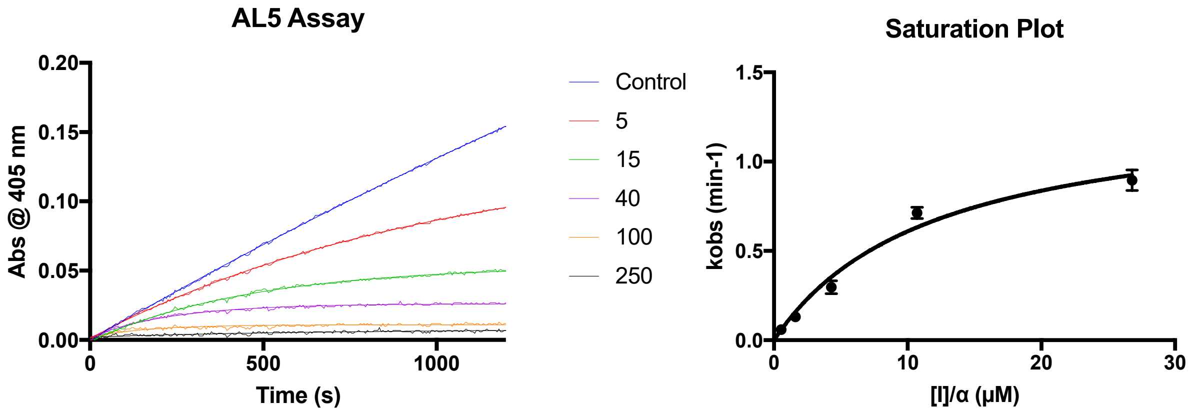

$k_{inact}/K_I = (112 \pm 41) \times 10^3\ M^{-1}min^{-1}$

$k_{inact} = 1.34 \pm 0.20\ min^{-1}$

$K_I = 11.9 \pm 4.0\ \mu M$

Compound 22f

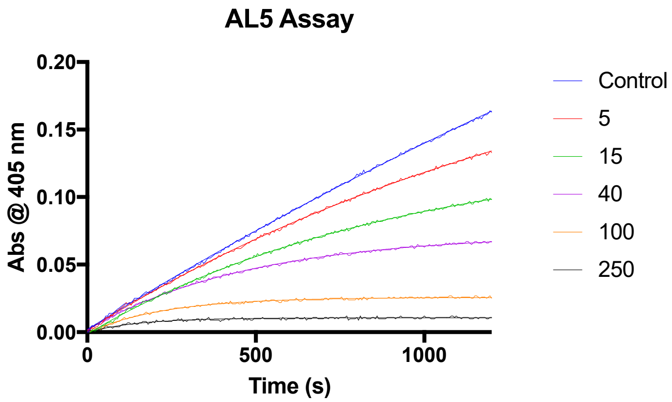

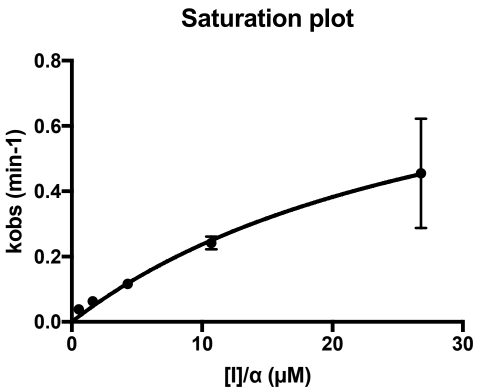

$k_{inact}/K_I = (31.2 \pm 10.0) \times 10^3\ M^{-1}min^{-1}$

$k_{inact} = 0.988 \pm 0.168\ min^{-1}$

$K_I = 31.7 \pm 8.6\ \mu M$

Compound 22g

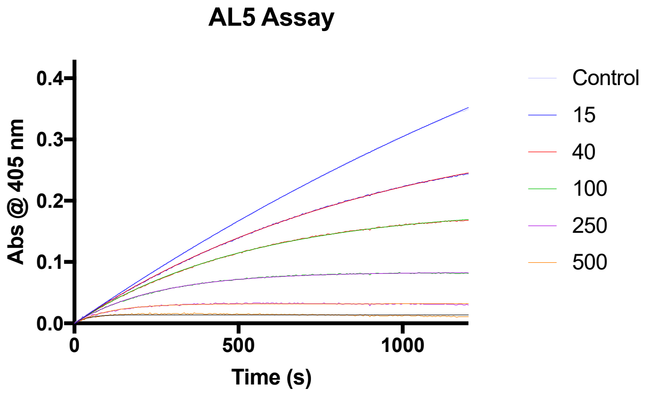

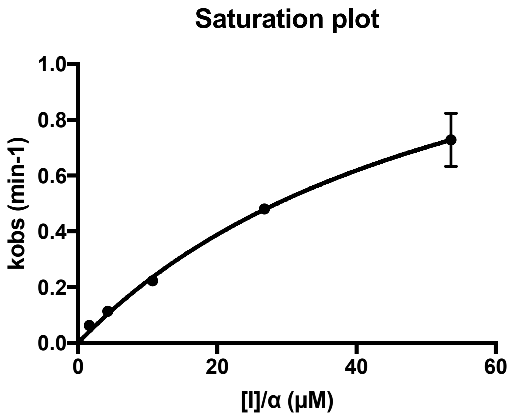

$k_{inact}/K_I = (26.1 \pm 4.9) \times 10^3\ M^{-1}min^{-1}$

$k_{inact} = 1.52 \pm 0.15\ min^{-1}$

$K_I = 58.1 \pm 9.4\ \mu M$

Compound 22h

Compound was too insoluble in assay conditions.

Compound 22i

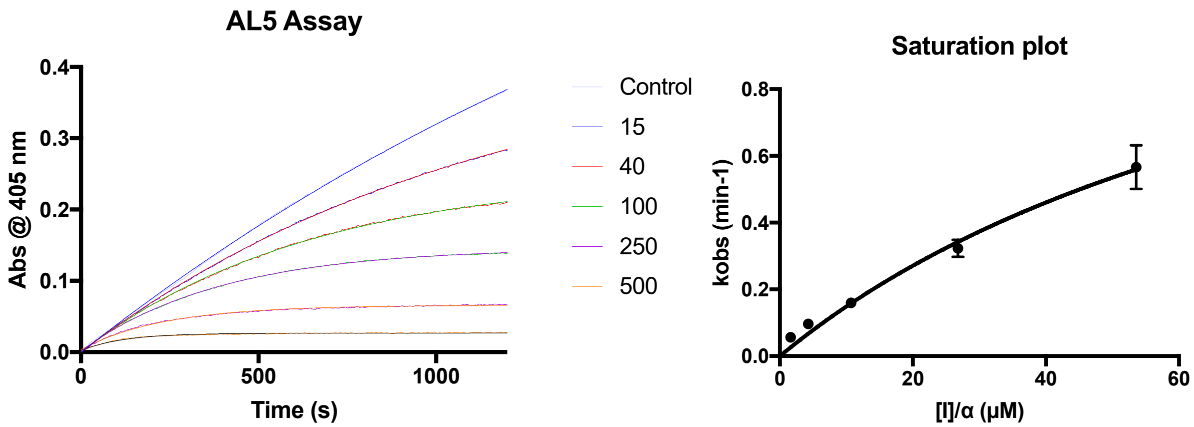

$k_{\text{inact}}/K_{\text{I}} = (16.5 \pm 9.4) \times 10^3\ \text{M}^{-1}\text{min}^{-1}$

$k_{\text{inact}} = 1.53 \pm 0.49\ \text{min}^{-1}$

$K_{\text{I}} = 92.8 \pm 43.3\ \mu\text{M}$

EB-2-16

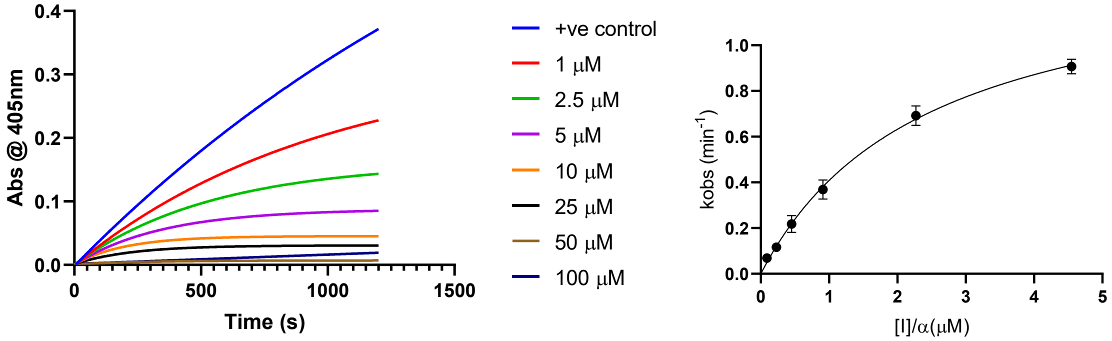

$k_{inact}/K_I = (576 \pm 50) \times 10^3\ \text{M}^{-1}\text{min}^{-1}$

$k_{inact} = 1.40 \pm 0.05\ \text{min}^{-1}$

$K_I = 2.43 \pm 0.19\ \mu\text{M}$

Compound 25a

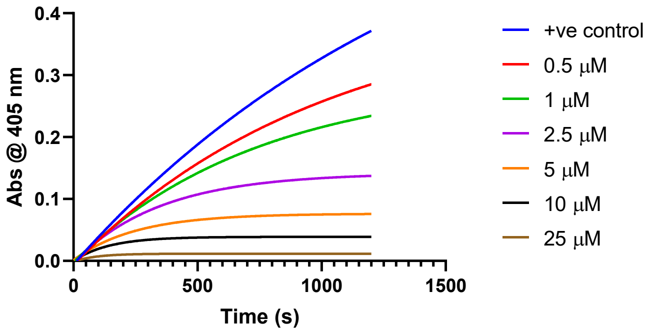

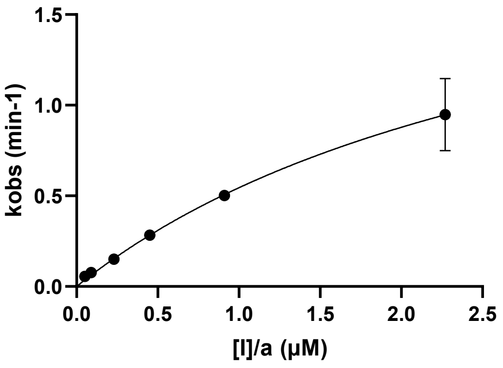

k_{inact}/K_I = (718 ± 91) × 10^3 $M^{-1}min^{-1}$

k_{inact} = 2.26 ± 0.16 min^{-1}

K_I = 3.15 ± 0.33 μM

Compound 25b

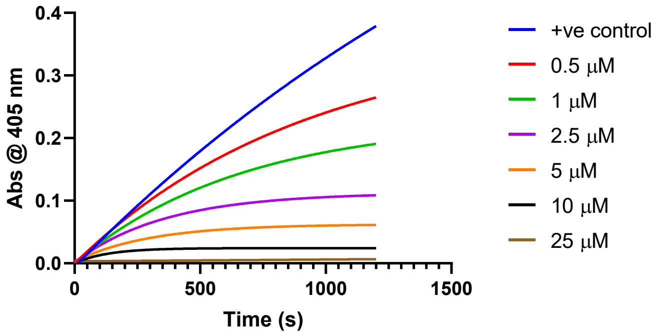

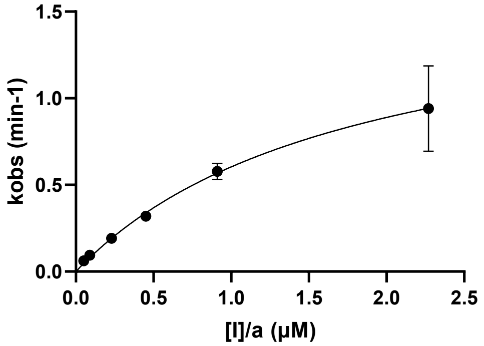

k_{inact}/K_I = (941 ± 101) × 10^3 $M^{-1}min^{-1}$

k_{inact} = 1.69 ± 0.09 min^{-1}

K_I = 1.79 ± 0.17 μM

Compound 25c

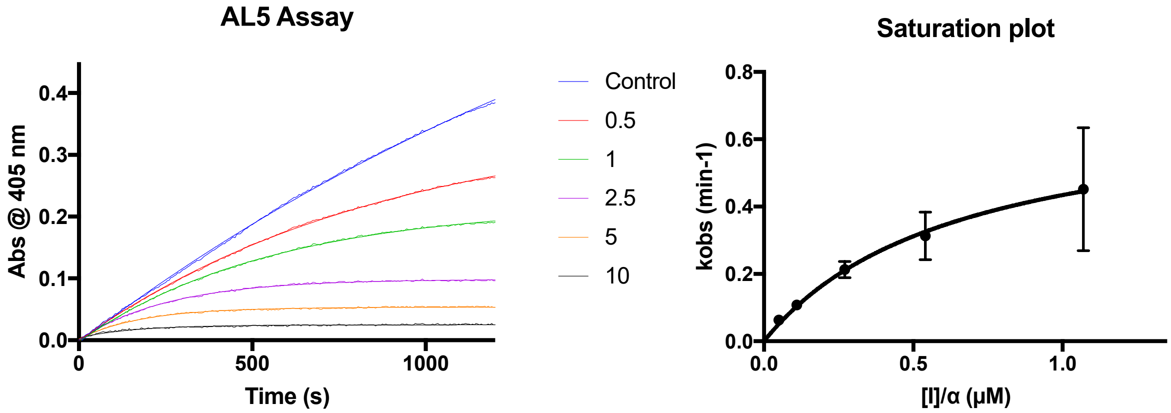

$k_{\mathrm{inact}}/K_{\mathrm{I}} = (1104 \pm 162) \times 10^3\ \mathrm{M}^{-1}\mathrm{min}^{-1}$

$k_{\mathrm{inact}} = 0.716 \pm 0.047\ \mathrm{min}^{-1}$

$K_{\mathrm{I}} = 0.648 \pm 0.085\ \mu\mathrm{M}$

Compound 25d

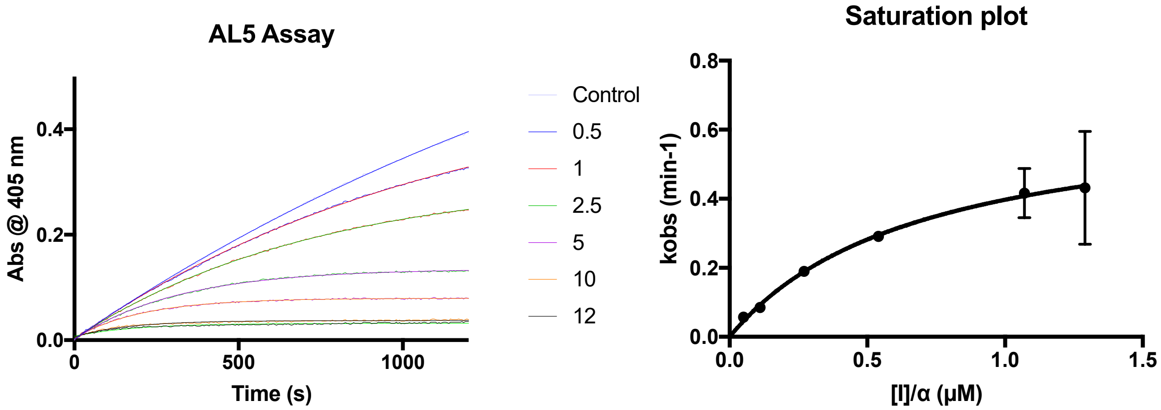

$k_{\mathrm{inact}}/K_{\mathrm{I}} = (972 \pm 103) \times 10^3\ \mathrm{M}^{-1}\mathrm{min}^{-1}$

$k_{\mathrm{inact}} = 0.671 \pm 0.030\ \mathrm{min}^{-1}$

$K_{\mathrm{I}} = 0.691 \pm 0.067\ \mu\mathrm{M}$

Compound 29

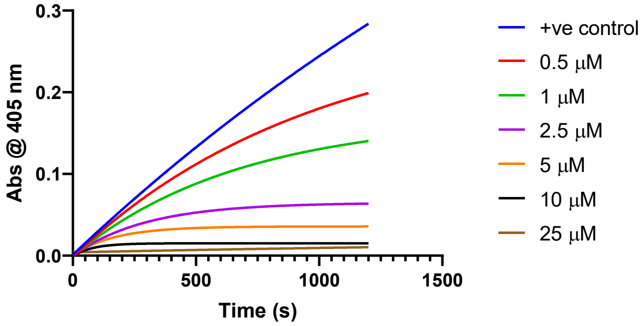

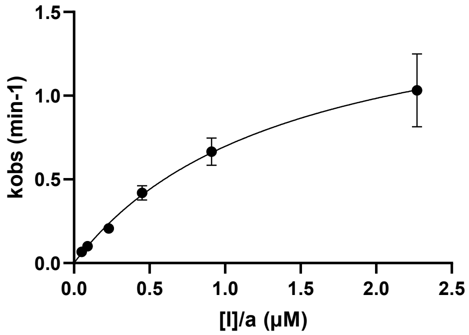

k_{inact}/K_{I} = (1195 ± 112) × 10^3 $M^{-1}min^{-1}$

k_{inact} = 1.67 ± 0.07 min^{-1}

K_{I} = 1.40 ± 0.12 µM

Compound 38

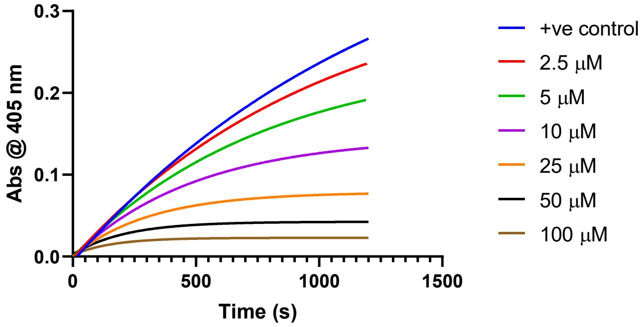

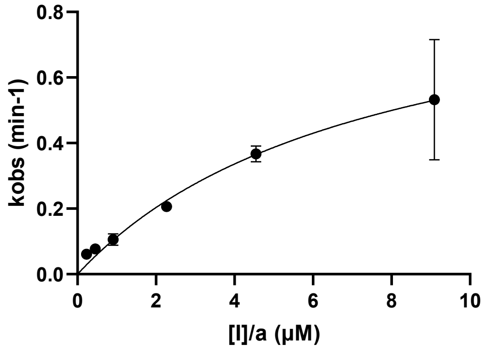

k_{inact}/K_I = (129 ± 36) × 10^3 $M^{-1}min^{-1}$

k_{inact} = 0.97 ± 0.14 min^{-1}

K_I = 7.56 ± 1.87 µM

HPLC Purity Chromatograms

Compound 6a

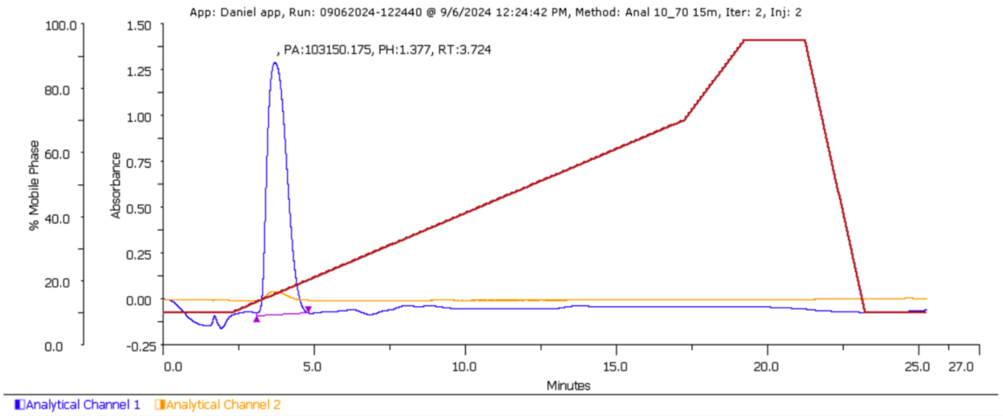

Sample Table

Injection Number	Peak Name	Retention Time (min)	Area (mAUmin x100)	Height (AU)	Sample Name	Sample Location	Fraction Site(s)	Area %	
2	1	3.724	103150.175	1.377	dw-prop	Sample Zone->161		100	

Compound 6b

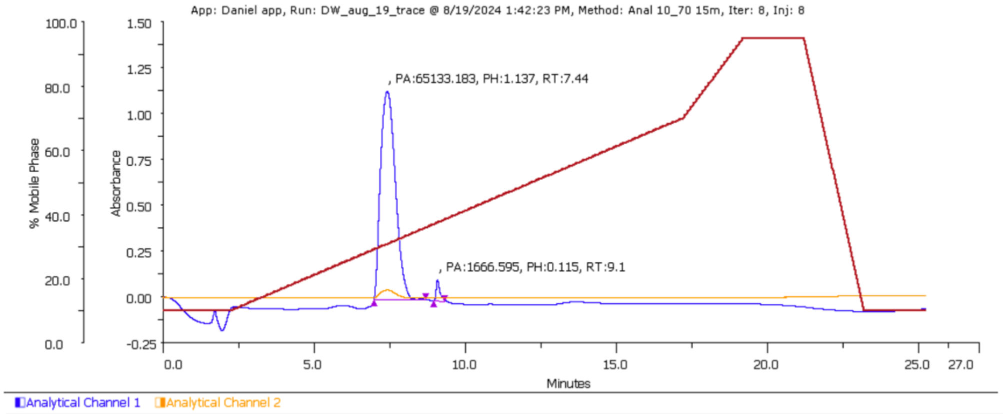

Sample Table

Injection Number	Peak Name	Retention Time (min)	Area (mAUmin x100)	Height (AU)	Sample Name	Sample Location	Fraction Site(s)	Area %	
8	1	7.44	65133.1833	1.137	DW-02-70	Sample Zone->156		97.505	
8	2	9.1	1666.5952	0.115	DW-02-70	Sample Zone->156		2.495	

Compound 6c

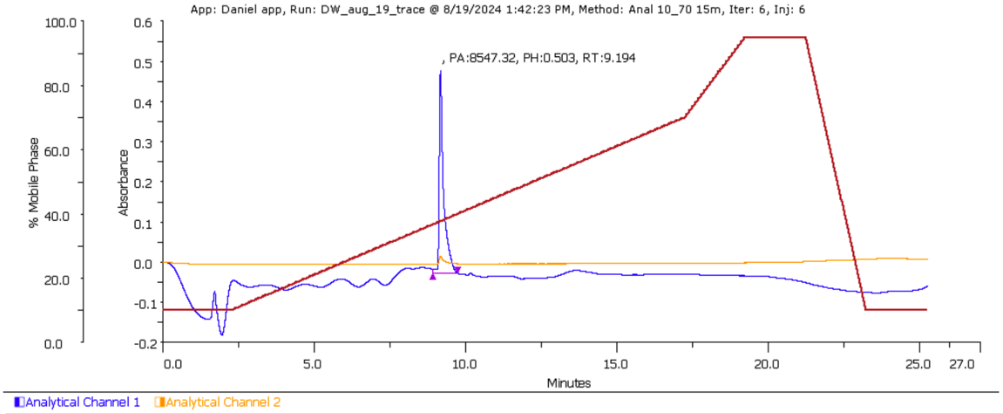

Sample Table

Injection Number	Peak Name	Retention Time (min)	Area (mAUmin x100)	Height (AU)	Sample Name	Sample Location	Fraction Site(s)	Area %	
6	1	9.194	8547.3201	0.503	DW-02-71	Sample Zone->157		100	

Compound 6d

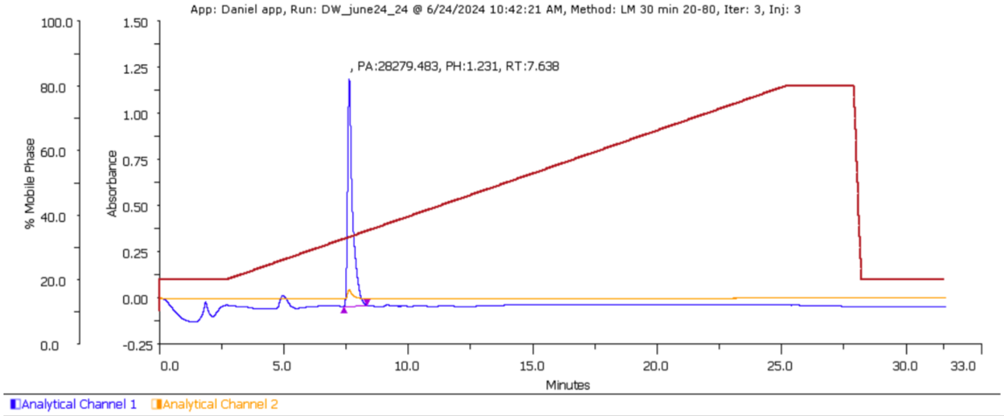

Sample Table

Injection Number	Peak Name	Retention Time (min)	Area (mAUmin x100)	Height (AU)	Sample Name	Sample Location	Fraction Site(s)	Area %	
3	1	7.638	28279.4827	1.231	DW-02-66	Sample Zone->161		100	

Compound 6e

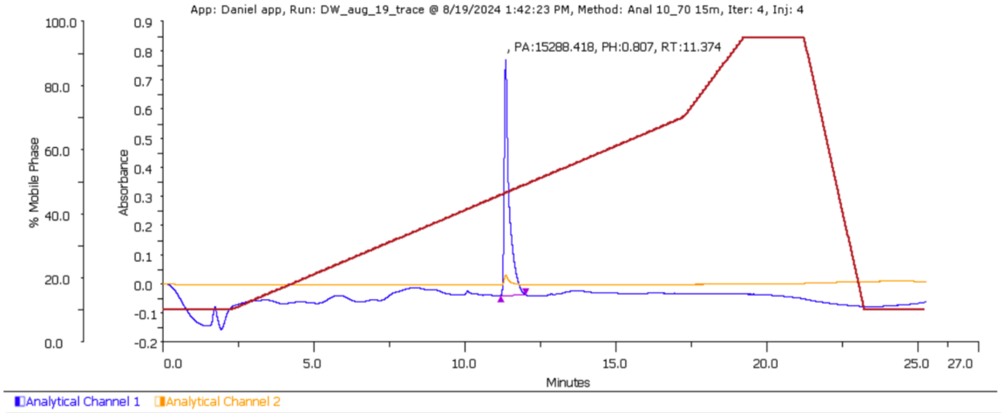

Sample Table

Injection Number	Peak Name	Retention Time (min)	Area (mAUmin x100)	Height (AU)	Sample Name	Sample Location	Fraction Site(s)	Area %	
4	1	11.374	15288.4178	0.807	DW-02-68	Sample Zone->163		100	

Compound 6f

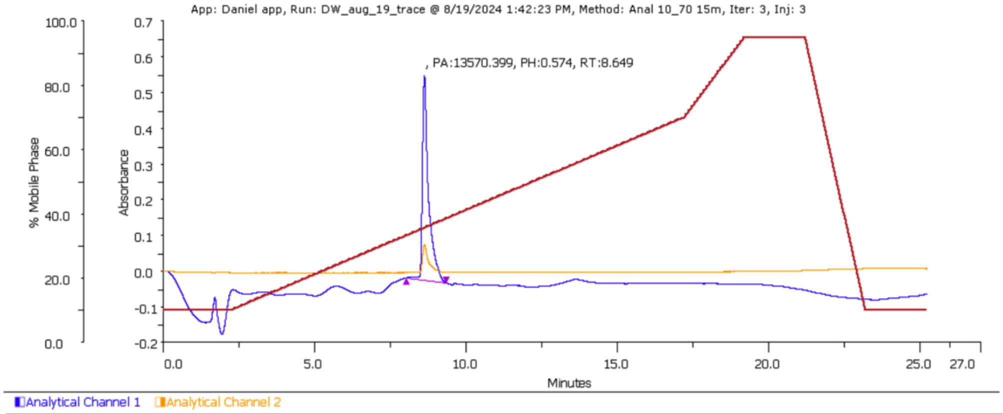

Sample Table

Injection Number	Peak Name	Retention Time (min)	Area (mAUmin x100)	Height (AU)	Sample Name	Sample Location	Fraction Site(s)	Area %	
3	1	8.649	13570.3993	0.574	DW-02-63	Sample Zone->162		100	

Compound 7a

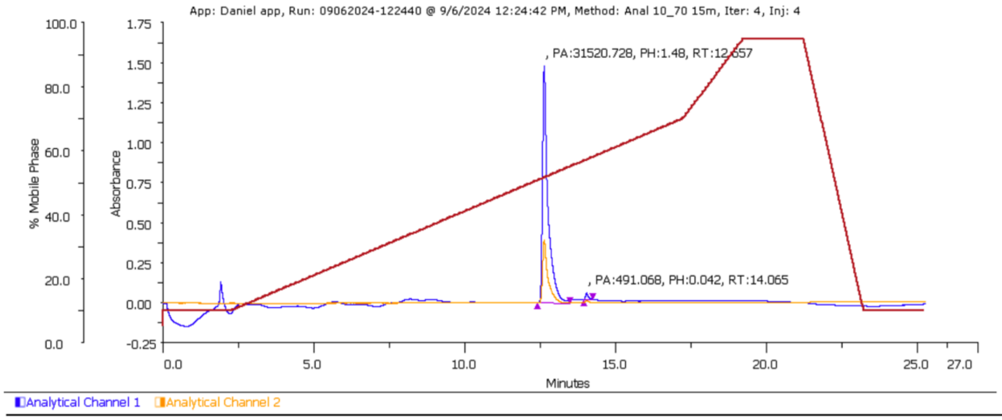

Sample Table

Injection Number	Peak Name	Retention Time (min)	Area (mAUmin x100)	Height (AU)	Sample Name	Sample Location	Fraction Site(s)	Area %	
4	1	12.657	31520.7283	1.48	dw-o-biphenyl	Sample Zone->162		98.466	
4	2	14.065	491.0679	0.042	dw-o-biphenyl	Sample Zone->162		1.534	

Compound 7b

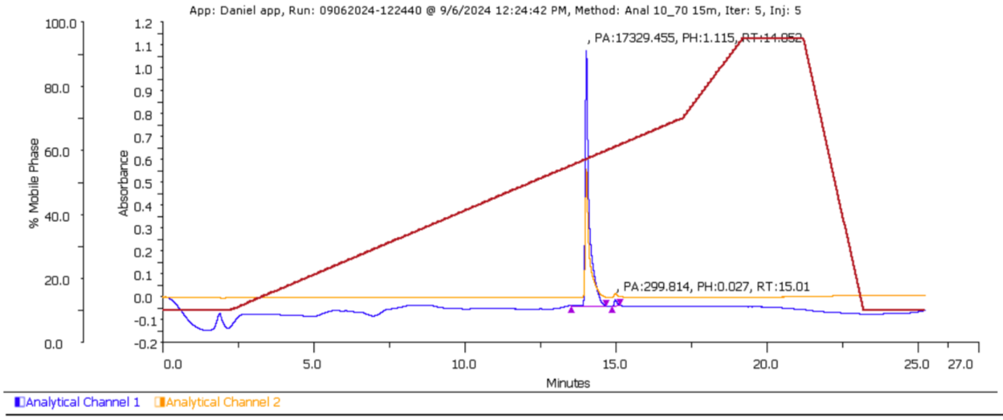

Sample Table

Injection Number	Peak Name	Retention Time (min)	Area (mAUmin x100)	Height (AU)	Sample Name	Sample Location	Fraction Site(s)	Area %	
5	1	14.052	17329.4546	1.115	dw-m-biphenyl	Sample Zone->163		98.299	
5	2	15.01	299.8137	0.027	dw-m-biphenyl	Sample Zone->163		1.701	

Compound 7c

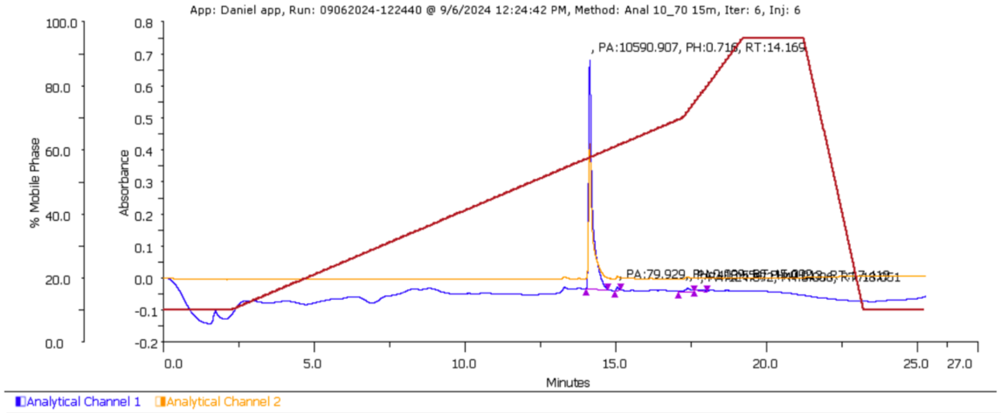

Sample Table

Injection Number	Peak Name	Retention Time (min)	Area (mAUmin x100)	Height (AU)	Sample Name	Sample Location	Fraction Site(s)	Area %	
6	1	14.169	10590.907	0.716	dw-p-biphenyl	Sample Zone->156		94.441	
6	2	15.092	79.9295	0.009	dw-p-biphenyl	Sample Zone->156		0.713	
6	3	17.419	418.5537	0.013	dw-p-biphenyl	Sample Zone->156		3.732	
6	4	18.051	124.8718	0.006	dw-p-biphenyl	Sample Zone->156		1.114	

Compound 21a

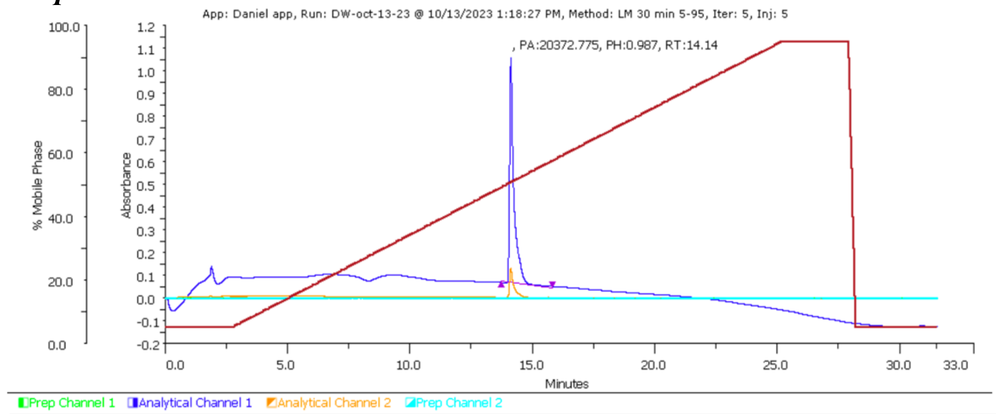

Sample Table

Injection Number	Peak Name	Retention Time (min)	Area (mAUmin x100)	Height (AU)	Sample Name	Sample Location	Fraction Site(s)	Area %	
5	1	14.14	20372.7747	0.987	DW-02-22	Sample Zone->156		100	

Compound 21b

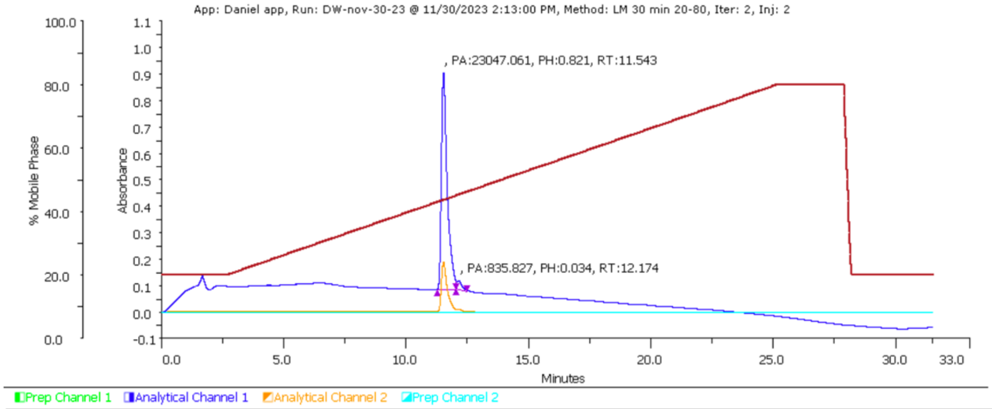

Sample Table

Injection Number	Peak Name	Retention Time (min)	Area (mAUmin x100)	Height (AU)	Sample Name	Sample Location	Fraction Site(s)	Area %	
2	1	11.543	23047.0611	0.821	DW-02-21	Sample Zone->161		96.5	
2	2	12.174	835.8268	0.034	DW-02-21	Sample Zone->161		3.5	

Compound 21c

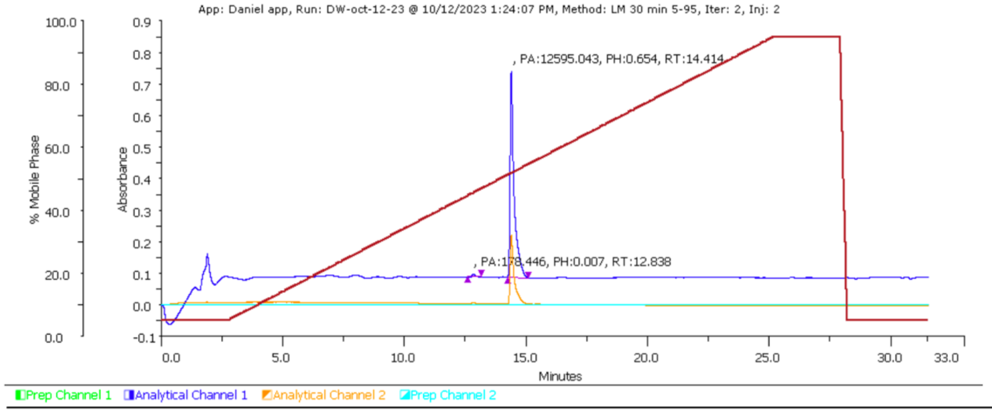

Sample Table

Injection Number	Peak Name	Retention Time (min)	Area (mAUmin x100)	Height (AU)	Sample Name	Sample Location	Fraction Site(s)	Area %	
2	1	12.838	178.446	0.007	DW-02-19	Sample Zone->161		1.397	
2	2	14.414	12595.0427	0.654	DW-02-19	Sample Zone->161		98.603	

Compound 21d

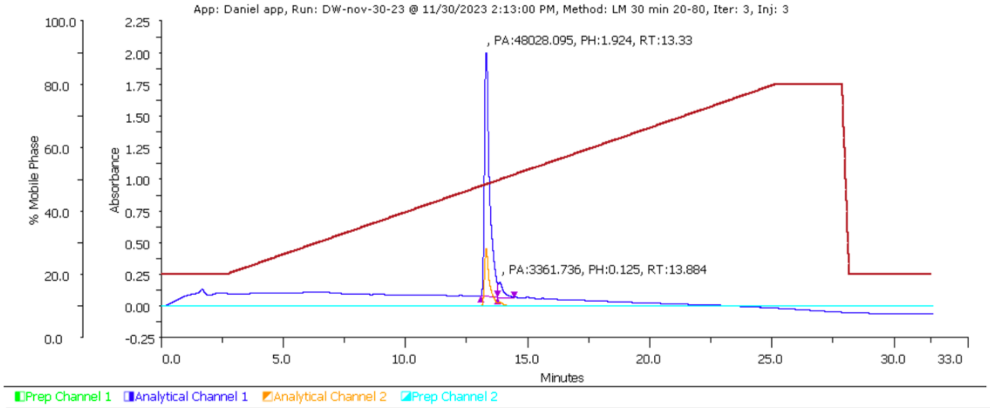

Sample Table

Injection Number	Peak Name	Retention Time (min)	Area (mAUmin x100)	Height (AU)	Sample Name	Sample Location	Fraction Site(s)	Area %	
3	1	13.33	48028.0948	1.924	DW-02-23	Sample Zone->162		93.458	
3	2	13.884	3361.736	0.125	DW-02-23	Sample Zone->162		6.542	

Compound 21e

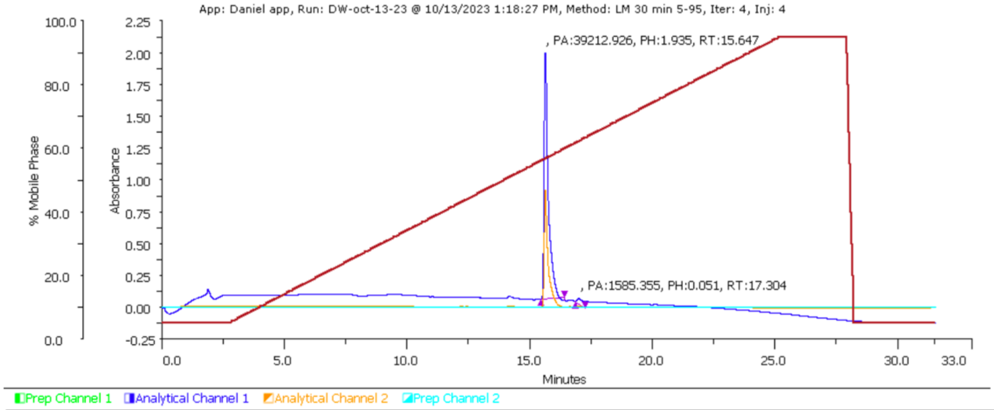

Sample Table

Injection Number	Peak Name	Retention Time (min)	Area (mAUmin x100)	Height (AU)	Sample Name	Sample Location	Fraction Site(s)	Area %	
4	1	15.647	39212.9255	1.935	DW-02-25	Sample Zone->163		96.114	
4	2	17.304	1585.3546	0.051	DW-02-25	Sample Zone->163		3.886	

Compound 21f

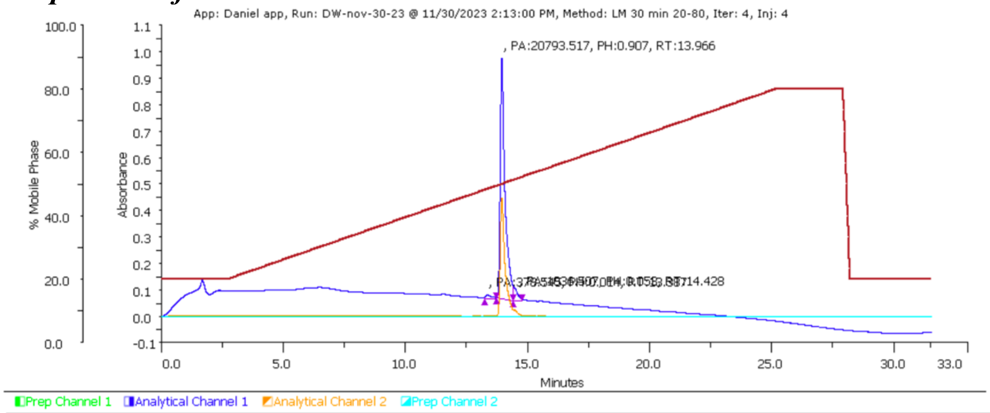

Sample Table

Injection Number	Peak Name	Retention Time (min)	Area (mAUmin x100)	Height (AU)	Sample Name	Sample Location	Fraction Site(s)	Area %	
4	1	13.387	378.5453	0.014	DW-02-24	Sample Zone->163		1.705	
4	2	13.966	20793.5167	0.907	DW-02-24	Sample Zone->163		93.628	
4	3	14.428	1036.5074	0.053	DW-02-24	Sample Zone->163		4.667	

Compound 21g

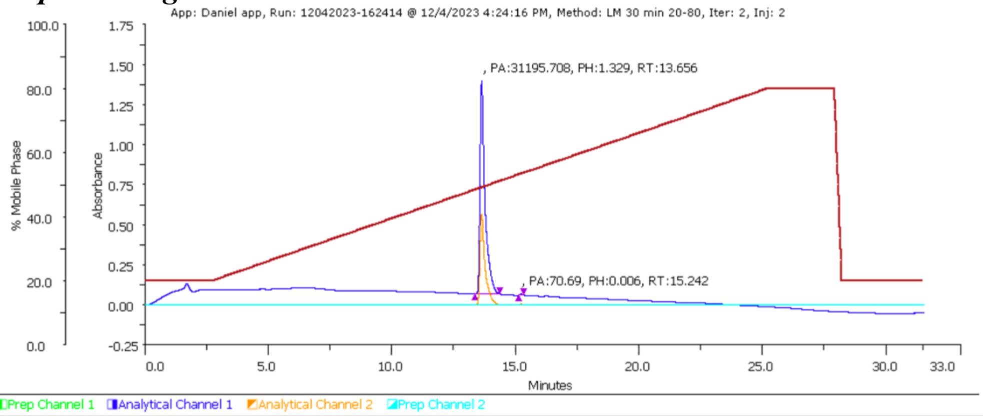

Sample Table

Injection Number	Peak Name	Retention Time (min)	Area (mAUmin x100)	Height (AU)	Sample Name	Sample Location	Fraction Site(s)	Area %	
2	1	13.656	31195.708	1.329	DW-02-27	Sample Zone->161		99.774	
2	2	15.242	70.6903	0.006	DW-02-27	Sample Zone->161		0.226	

Compound 21h

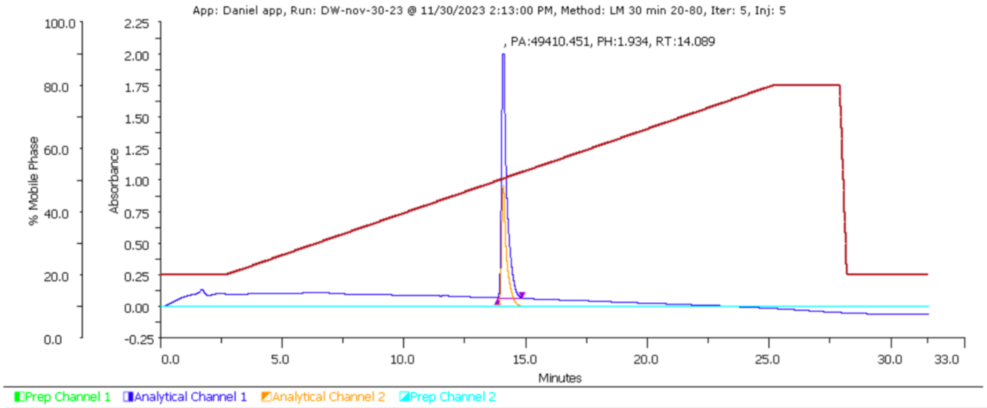

Sample Table

Injection Number	Peak Name	Retention Time (min)	Area (mAUmin x100)	Height (AU)	Sample Name	Sample Location	Fraction Site(s)	Area %	
5	1	14.089	49410.4508	1.934	DW-02-28	Sample Zone->156		100	

Compound 21i

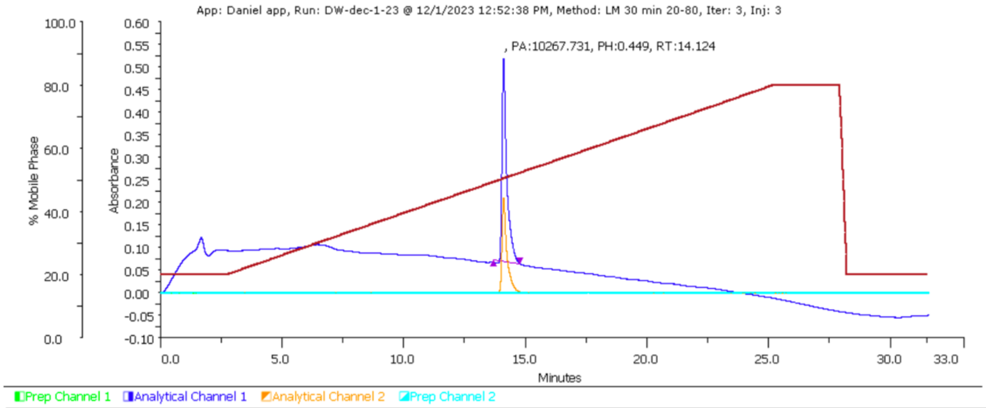

Sample Table

Injection Number	Peak Name	Retention Time (min)	Area (mAUmin x100)	Height (AU)	Sample Name	Sample Location	Fraction Site(s)	Area %	
3	1	14.124	10267.7315	0.449	DW-02-31	Sample Zone->161		100	

Compound 22a

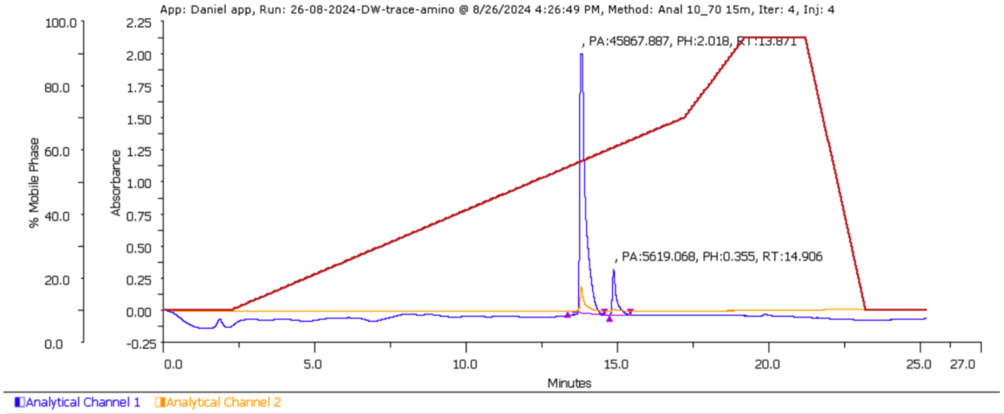

Sample Table

Injection Number	Peak Name	Retention Time (min)	Area (mAUmin x100)	Height (AU)	Sample Name	Sample Location	Fraction Site(s)	Area %	
4	1	13.871	45867.8868	2.018	DW--CB-09	Sample Zone->159		89.086	
4	2	14.906	5619.0684	0.355	DW--CB-09	Sample Zone->159		10.914	

Compound 22b

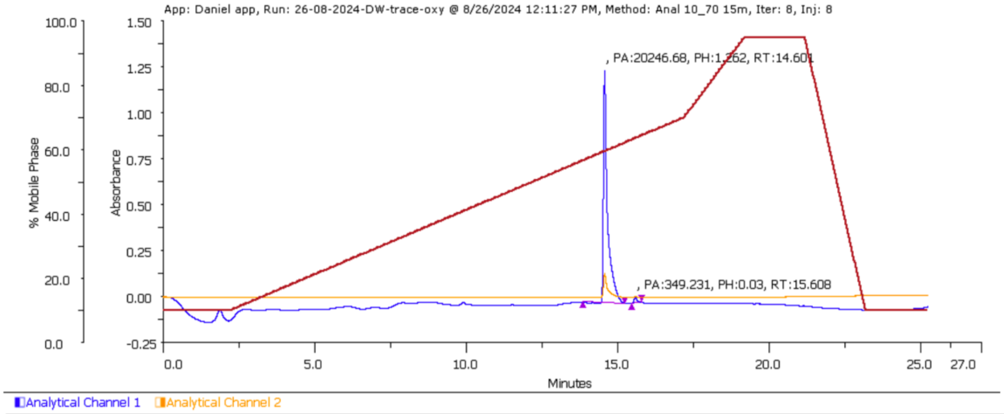

Sample Table

Injection Number	Peak Name	Retention Time (min)	Area (mAUmin x100)	Height (AU)	Sample Name	Sample Location	Fraction Site(s)	Area %	
8	1	14.601	20246.6798	1.262	DW-CB-08	Sample Zone->158		98.304	
8	2	15.608	349.231	0.03	DW-CB-08	Sample Zone->158		1.696	

Compound 22c

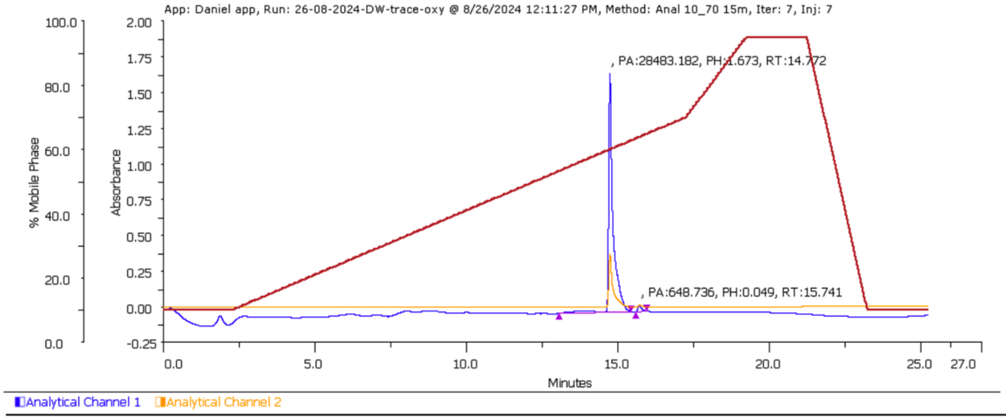

Sample Table

Injection Number	Peak Name	Retention Time (min)	Area (mAUmin x100)	Height (AU)	Sample Name	Sample Location	Fraction Site(s)	Area %	
7	1	14.772	28483.1819	1.673	DW-02-18	Sample Zone->159		97.773	
7	2	15.741	648.736	0.049	DW-02-18	Sample Zone->159		2.227	

Compound 22d

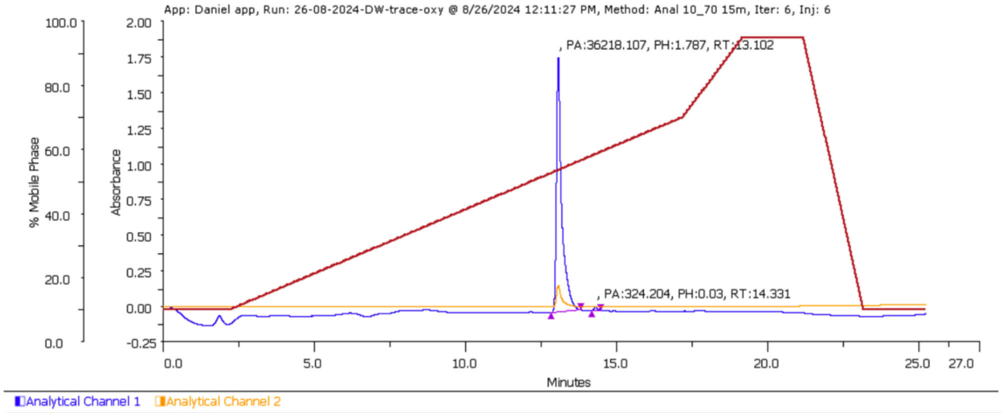

Sample Table

Injection Number	Peak Name	Retention Time (min)	Area (mAUmin x100)	Height (AU)	Sample Name	Sample Location	Fraction Site(s)	Area %	
6	1	13.102	36218.1072	1.787	DW-02-54	Sample Zone->163		99.113	
6	2	14.331	324.2039	0.03	DW-02-54	Sample Zone->163		0.887	

Compound 22e

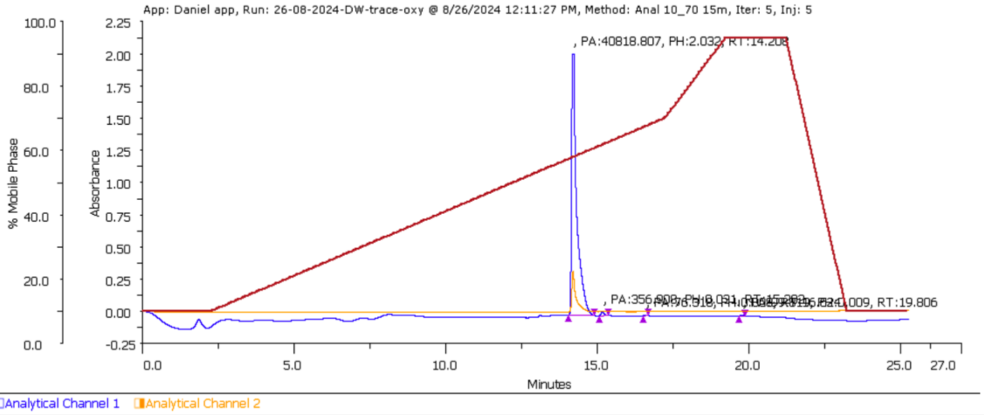

Sample Table

Injection Number	Peak Name	Retention Time (min)	Area (mAUmin x100)	Height (AU)	Sample Name	Sample Location	Fraction Site(s)	Area %	
5	1	14.208	40818.8067	2.032	DW-02-42	Sample Zone->162		98.711	
5	2	15.202	356.9077	0.031	DW-02-42	Sample Zone->162		0.863	
5	3	16.624	76.3176	0.008	DW-02-42	Sample Zone->162		0.185	
5	4	19.806	99.8193	0.009	DW-02-42	Sample Zone->162		0.241	

Compound 22f

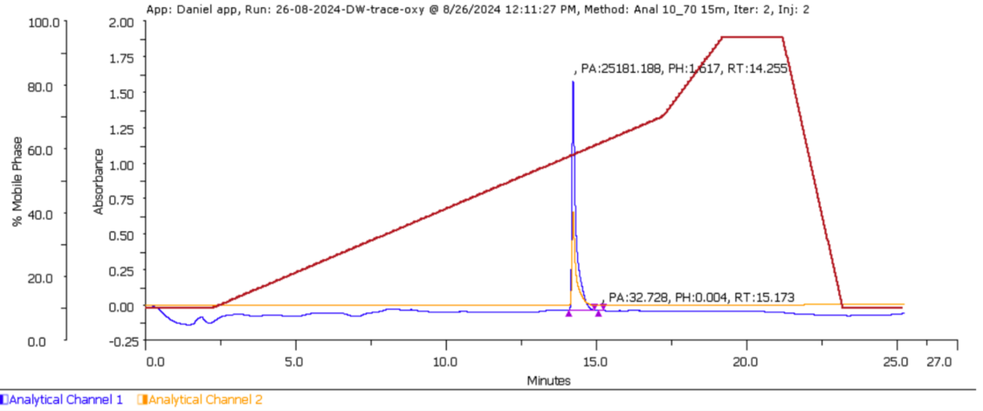

Sample Table

Injection Number	Peak Name	Retention Time (min)	Area (mAUmin x100)	Height (AU)	Sample Name	Sample Location	Fraction Site(s)	Area %	
2	1	14.255	25181.1882	1.617	DW-02-37	Sample Zone->161		99.87	
2	2	15.173	32.7283	0.004	DW-02-37	Sample Zone->161		0.13	

Compound 22g

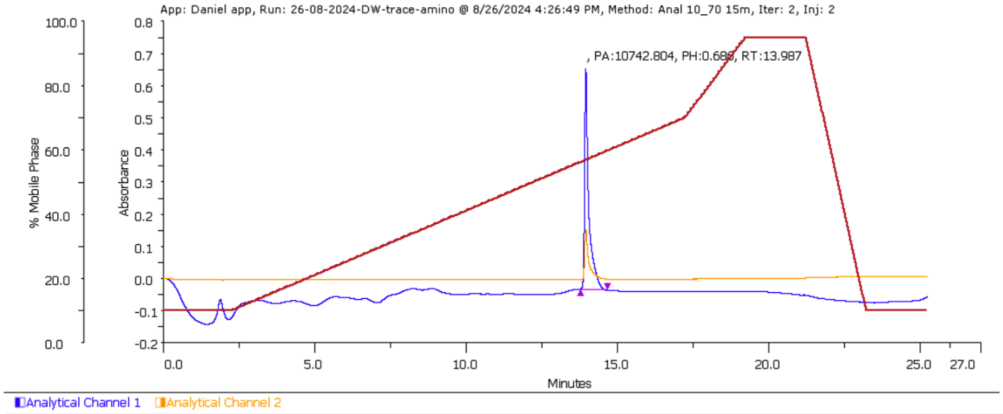

Sample Table

Injection Number	Peak Name	Retention Time (min)	Area (mAUmin x100)	Height (AU)	Sample Name	Sample Location	Fraction Site(s)	Area %	
2	1	13.987	10742.8037	0.686	DW-02-47	Sample Zone->162		100	

Compound 22h

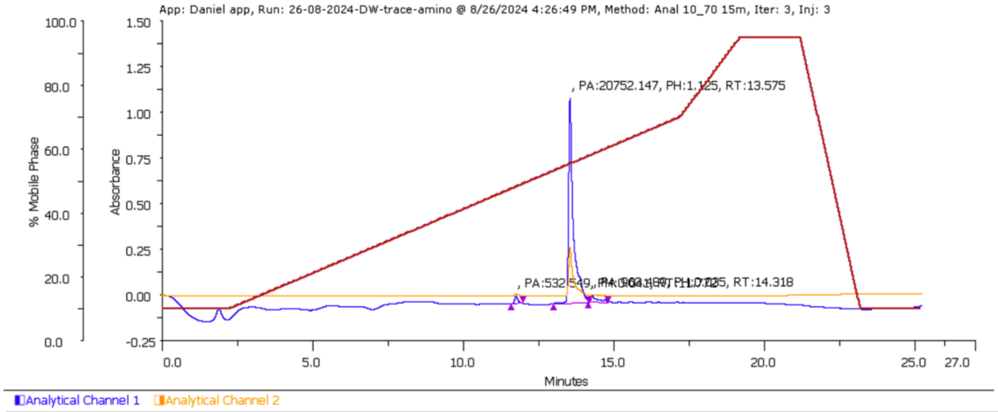

Sample Table

Injection Number	Peak Name	Retention Time (min)	Area (mAUmin x100)	Height (AU)	Sample Name	Sample Location	Fraction Site(s)	Area %	
3	1	11.772	532.549	0.041	DW-0-49	Sample Zone->163		2.394	
3	2	13.575	20752.1468	1.125	DW-0-49	Sample Zone->163		93.276	
3	3	14.318	963.4888	0.035	DW-0-49	Sample Zone->163		4.331	

Compound 22i

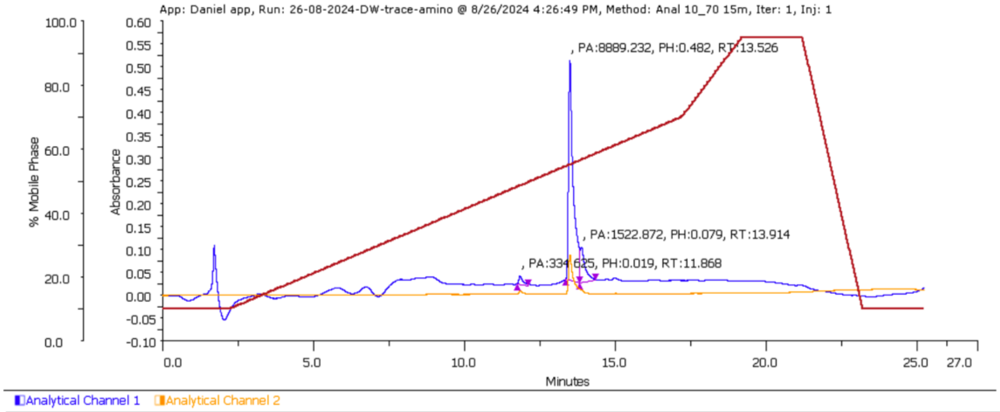

Sample Table

Injection Number	Peak Name	Retention Time (min)	Area (mAUmin x100)	Height (AU)	Sample Name	Sample Location	Fraction Site(s)	Area %	
1	1	11.868	334.6248	0.019	DW-02-32	Sample Zone->161		3.114	
1	2	13.526	8889.2319	0.482	DW-02-32	Sample Zone->161		82.716	
1	3	13.914	1522.872	0.079	DW-02-32	Sample Zone->161		14.171	

EB-2-16

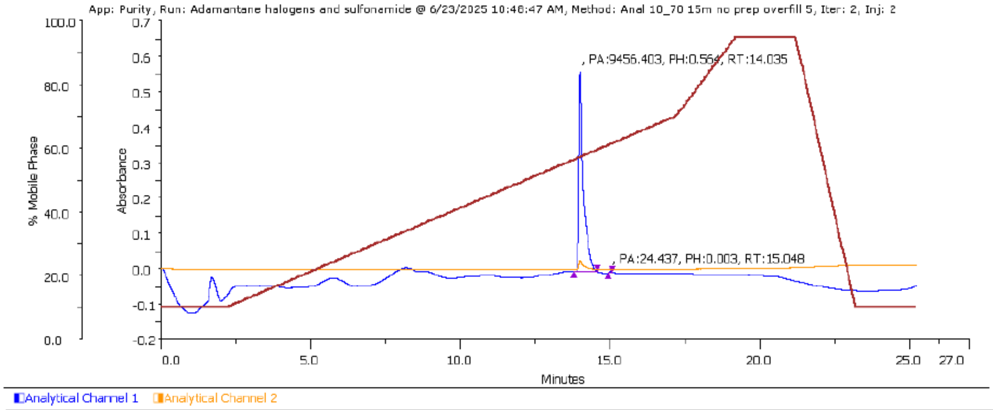

Sample Table

Injection Number	Peak Name	Retention Time (min)	Area (mAUmin x100)	Height (AU)	Sample Name	Sample Location	Fraction Site(s)	Area %	
2	1	14.035	9456.4034	0.564	Griffin	Sample Zone->156		99.742	
2	2	15.048	24.4373	0.003	Griffin	Sample Zone->156		0.258	

Compound 25a

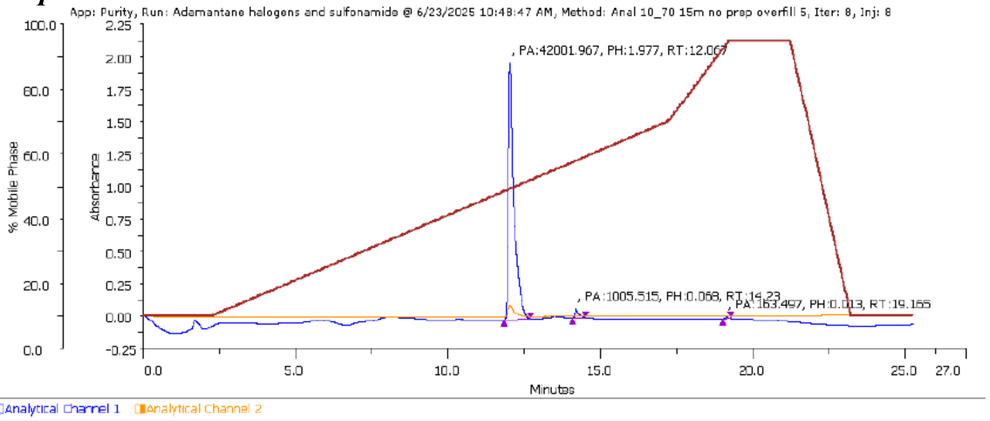

Sample Table

Injection Number	Peak Name	Retention Time (min)	Area (mAUmin x100)	Height (AU)	Sample Name	Sample Location	Fraction Site(s)	Area %	
8	1	12.067	42001.9668	1.977	ST-1-187	Sample Zone->159		97.292	
8	2	14.23	1005.5149	0.068	ST-1-187	Sample Zone->159		2.329	
8	3	19.165	163.4966	0.013	ST-1-187	Sample Zone->159		0.379	

Compound 25b

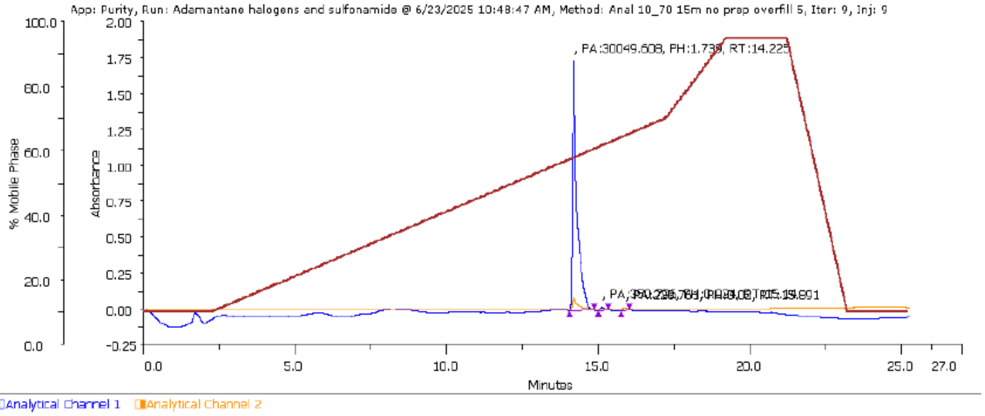

Sample Table

Injection Number	Peak Name	Retention Time (min)	Area (mAUmin x100)	Height (AU)	Sample Name	Sample Location	Fraction Site(s)	Area %	
9	1	14.225	30049.6078	1.739	ST-1-191	Sample Zone->163		98.124	
9	2	15.14	350.7964	0.024	ST-1-191	Sample Zone->163		1.145	
9	3	15.891	223.7609	0.02	ST-1-191	Sample Zone->163		0.731	

Compound 25c

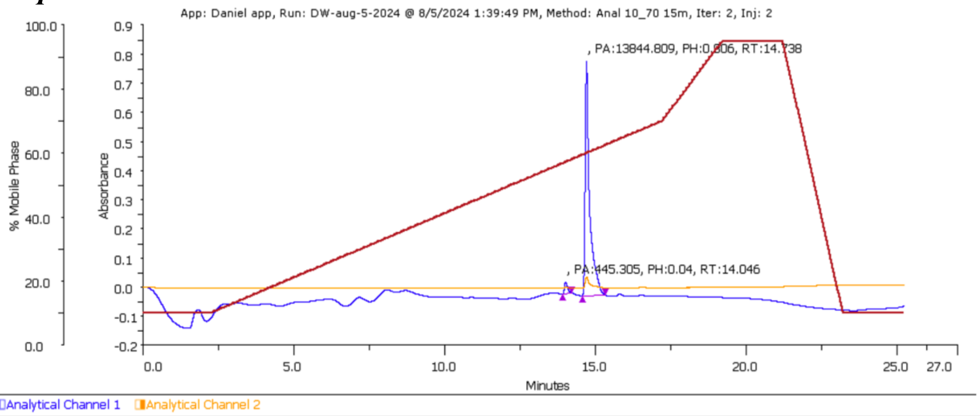

Sample Table

Injection Number	Peak Name	Retention Time (min)	Area (mAUmin x100)	Height (AU)	Sample Name	Sample Location	Fraction Site(s)	Area %	
2	1	14.046	445.305	0.04	DW-02-77	Sample Zone->161		3.116	
2	2	14.738	13844.8091	0.806	DW-02-77	Sample Zone->161		96.884	

Compound 25d

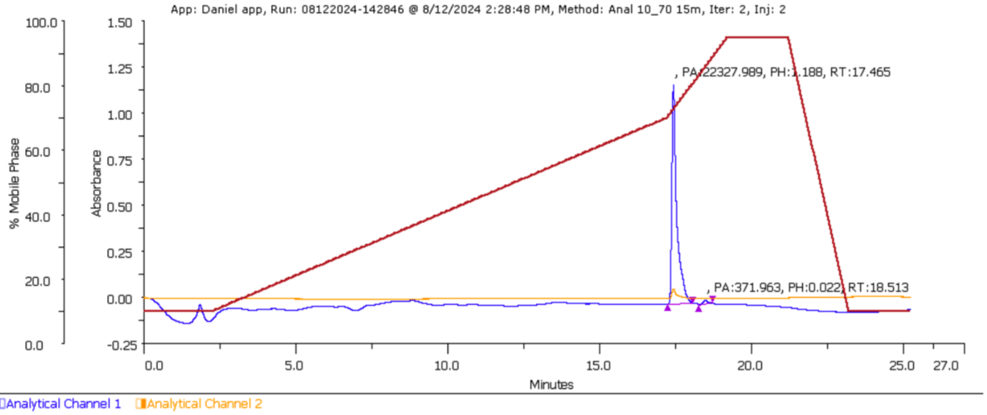

Sample Table

Injection Number	Peak Name	Retention Time (min)	Area (mAUmin x100)	Height (AU)	Sample Name	Sample Location	Fraction Site(s)	Area %	
2	1	17.465	22327.989	1.188	DW-02-80	Sample Zone->161		98.361	
2	2	18.513	371.9635	0.022	DW-02-80	Sample Zone->161		1.639	

Compound 29

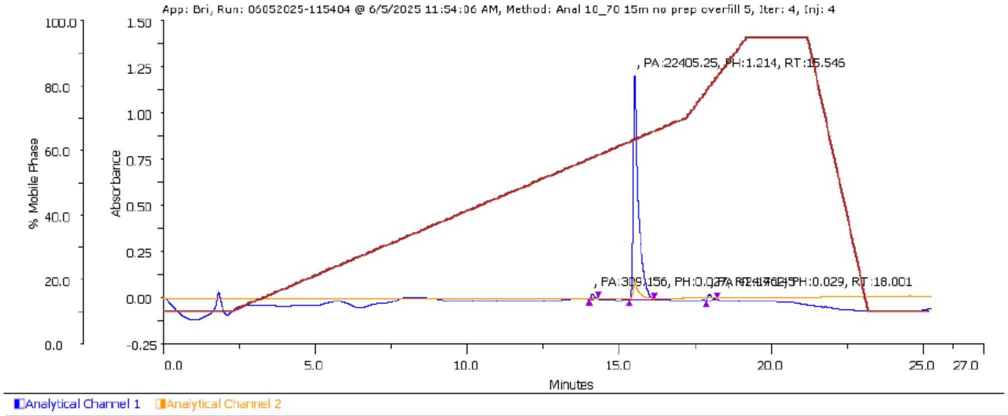

Sample Table

Injection Number	Peak Name	Retention Time (min)	Area (mAUmin x100)	Height (AU)	Sample Name	Sample Location	Fraction Site(s)	Area %	
4	1	14.145	309.1559	0.027	ST-1-159	Sample Zone->159		1.336	
4	2	15.546	22405.2498	1.214	ST-1-159	Sample Zone->159		96.828	
4	3	18.001	424.7622	0.029	ST-1-159	Sample Zone->159		1.836	

Compound 38

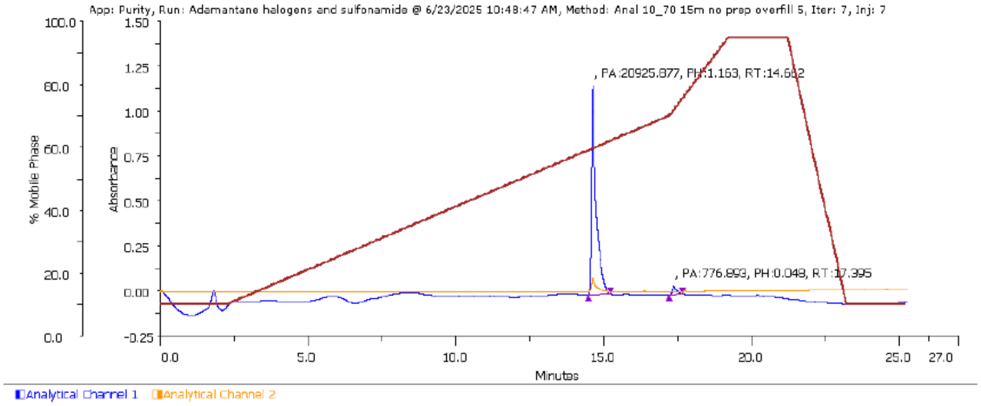

Sample Table

Injection Number	Peak Name	Retention Time (min)	Area (mAUmin x100)	Height (AU)	Sample Name	Sample Location	Fraction Site(s)	Area %	
7	1	14.662	20925.8773	1.163	ST-1-185	Sample Zone->158		96.42	
7	2	17.395	776.8933	0.048	ST-1-185	Sample Zone->158		3.58	

^{1}H and ^{13}C NMR Spectra

Compound 3

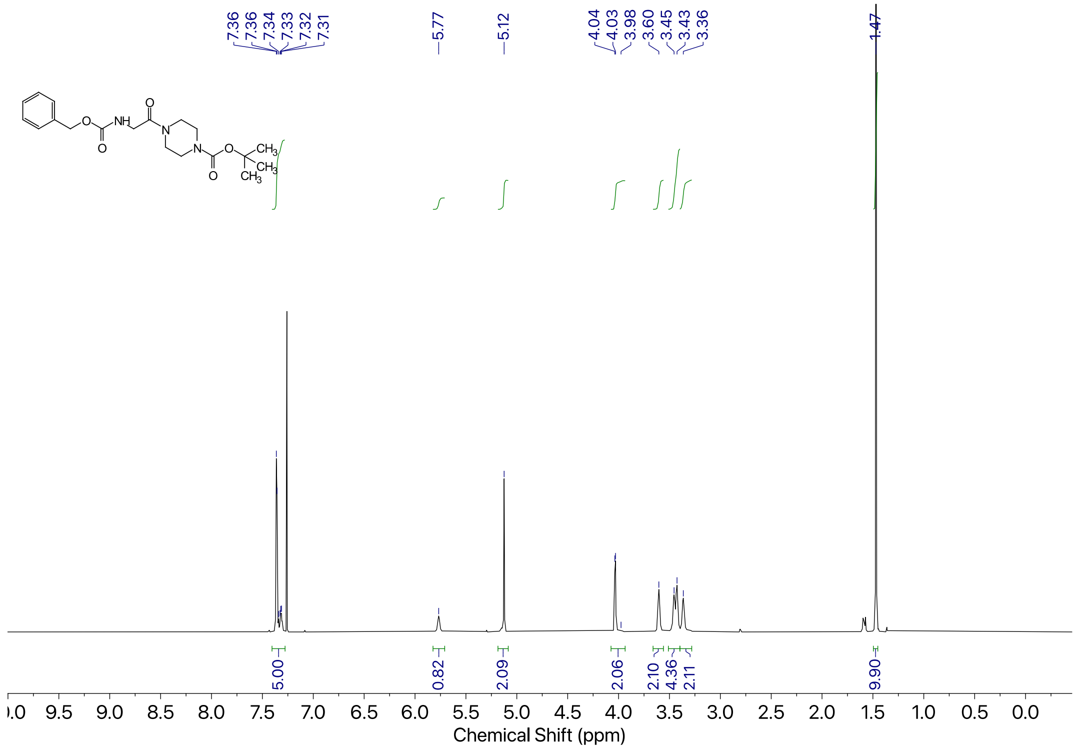

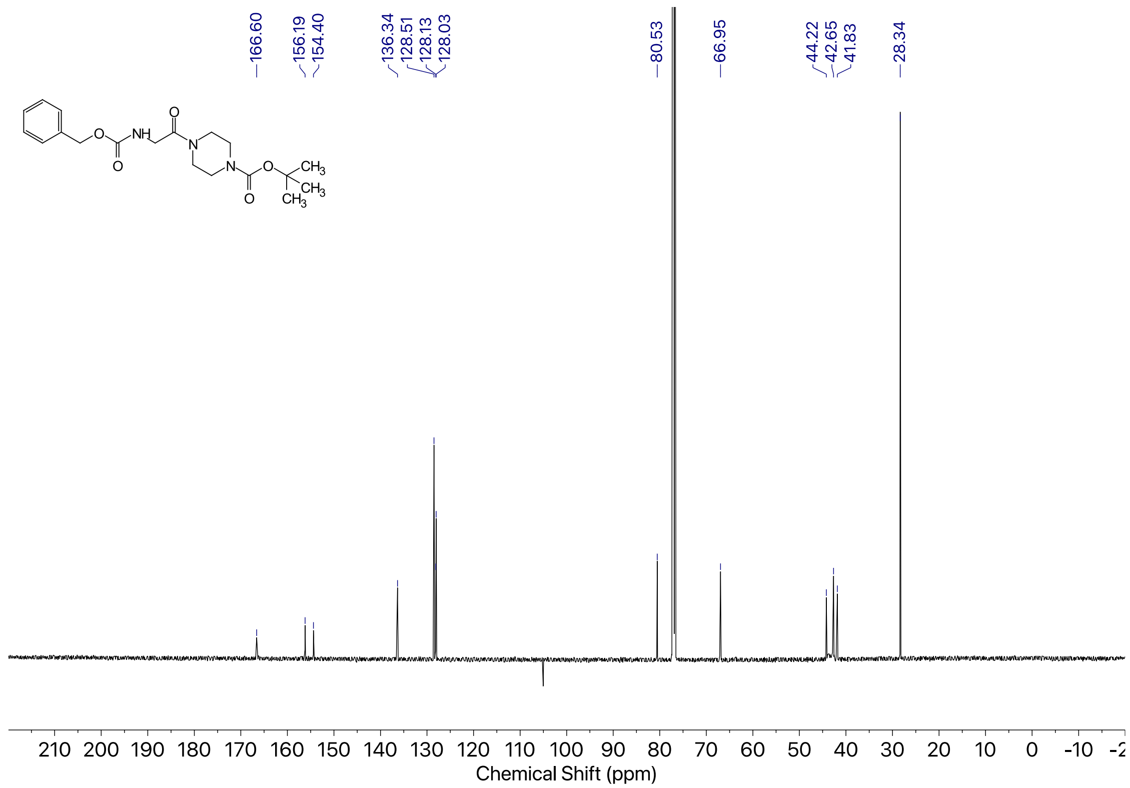

Compound 4

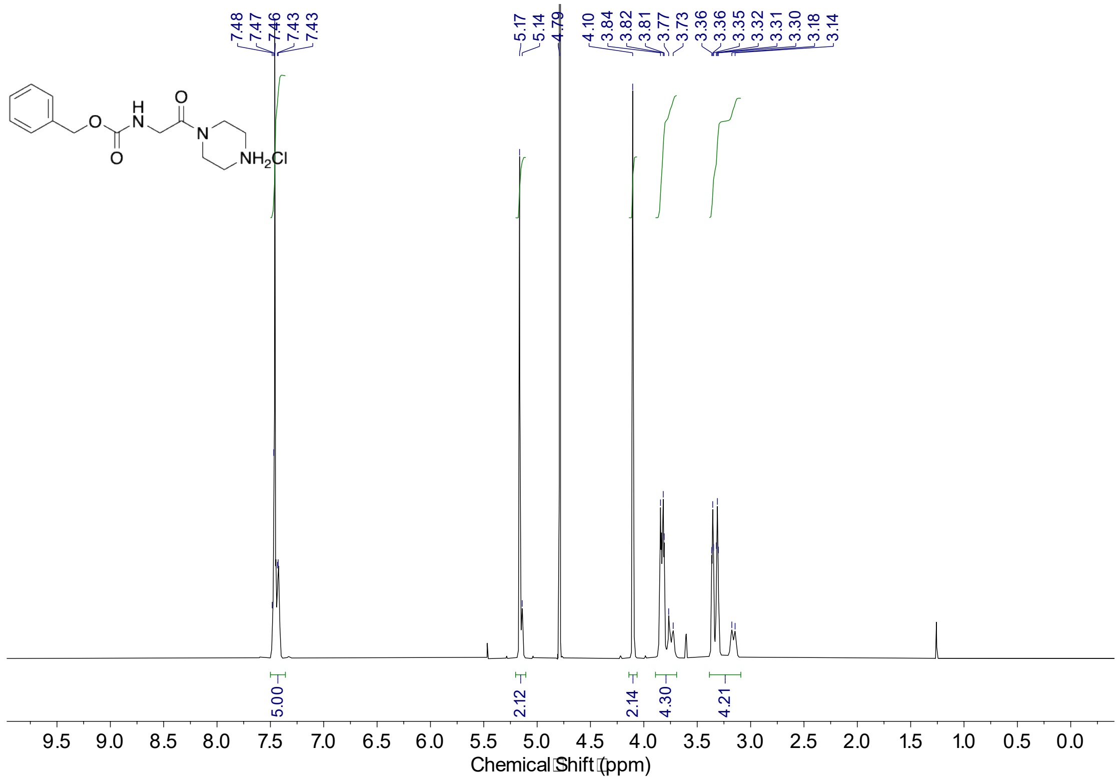

7.48
7.47
7.46
7.43
7.43
5.17
5.14
4.79
4.10
3.84
3.82
3.81
3.77
3.73
3.36
3.36
3.35
3.32
3.31
3.30
3.18
3.14
NH2Cl
5.00
2.12
2.14
4.30
4.21
9.5 9.0 8.5 8.0 7.5 7.0 6.5 6.0 5.5 5.0 4.5 4.0 3.5 3.0 2.5 2.0 1.5 1.0 0.5 0.0
Chemical Shift (ppm)

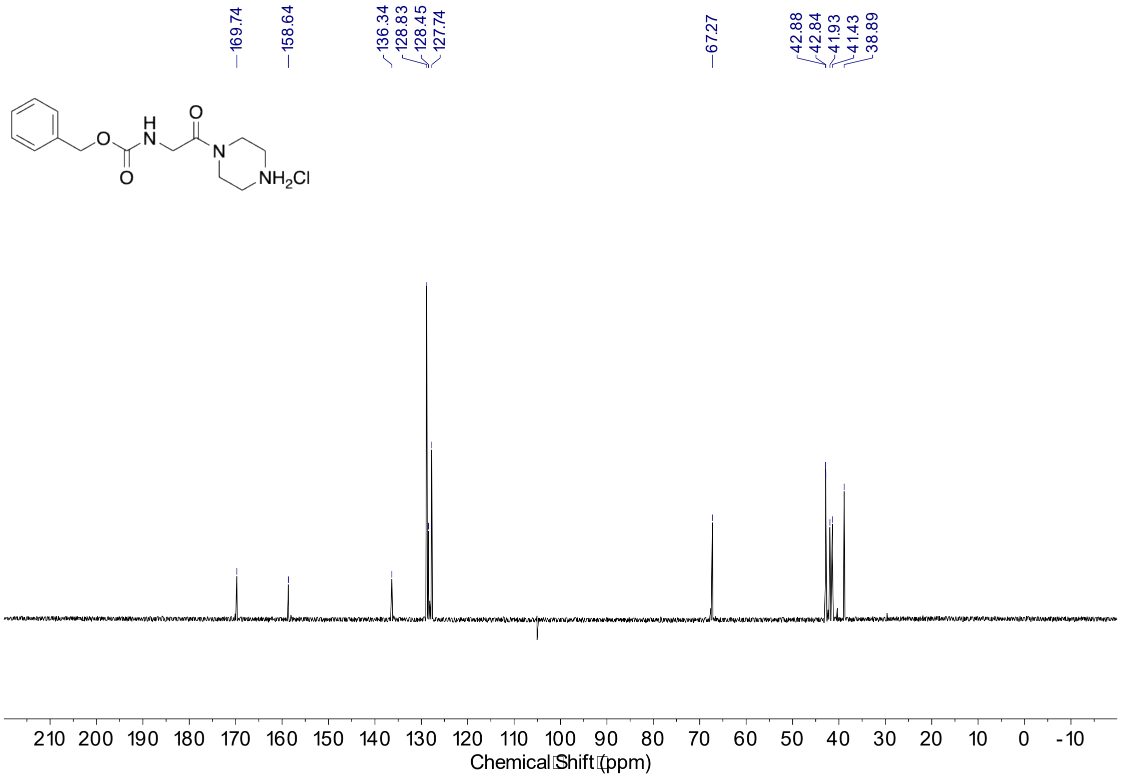

169.74
158.64
136.34
128.83
128.45
127.74
67.27
42.88
42.84
41.93
41.43
38.89
NH2Cl
210 200 190 180 170 160 150 140 130 120 110 100 90 80 70 60 50 40 30 20 10 0 -10
Chemical Shift (ppm)

Compound 5a

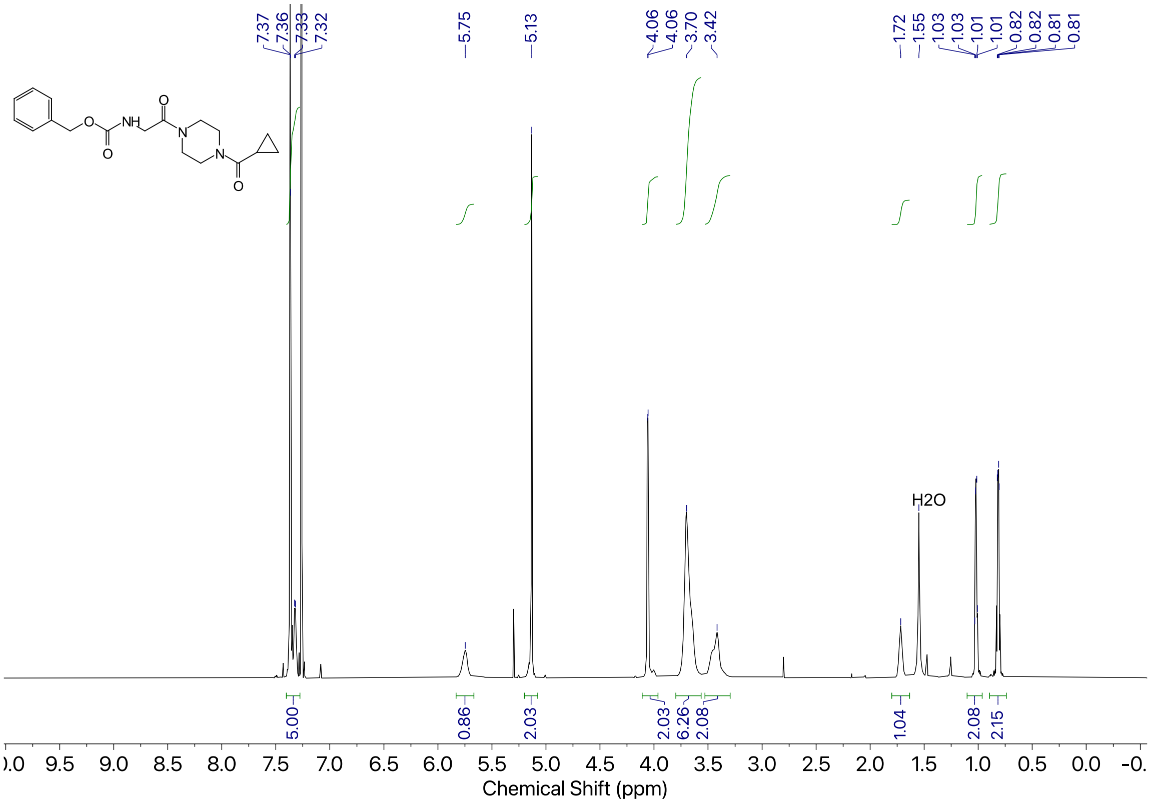

7.37
7.36
7.33
7.32
5.75
5.13
4.06
4.06
3.70
3.42
1.72
1.55
1.03
1.03
1.01
1.01
0.82
0.82
0.81
0.81
H2O
5.00
0.86
2.03
2.03
6.26
2.08
1.04
2.08
2.15
Chemical Shift (ppm)

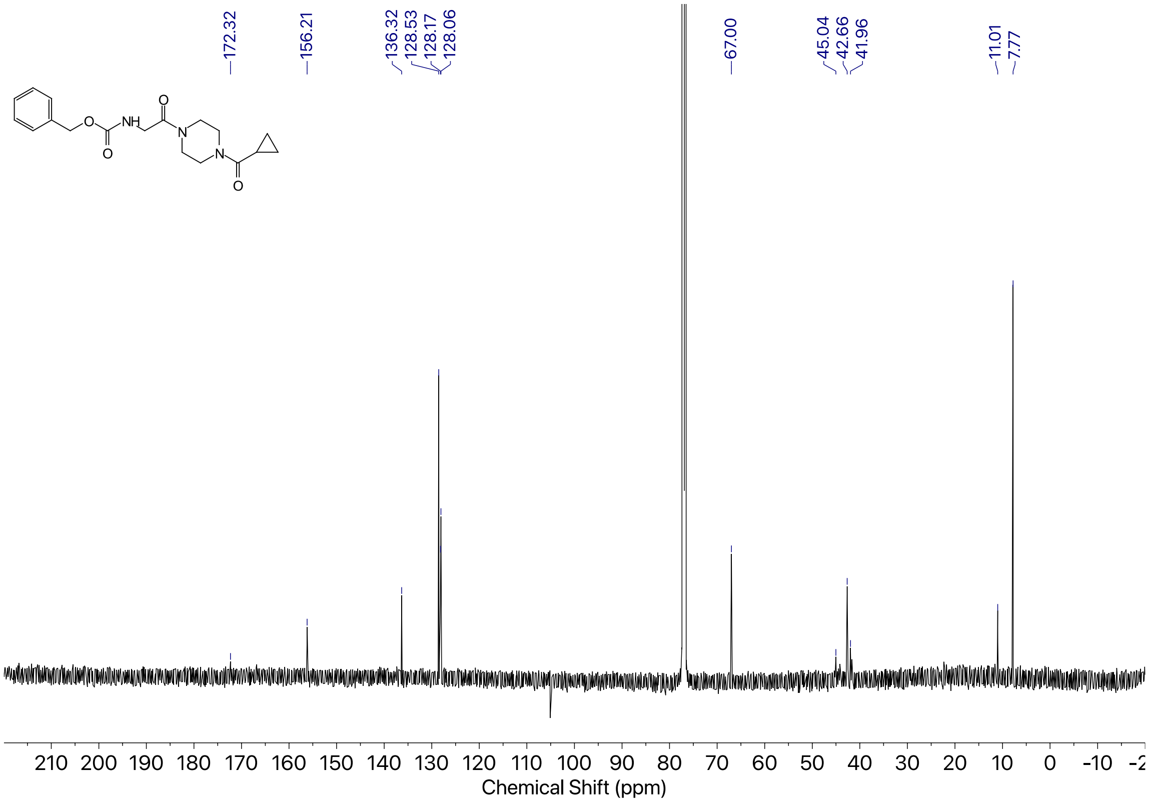

172.32
156.21
136.32
128.53
128.17
128.06
67.00
45.04
42.66
41.96
11.01
7.77
Chemical Shift (ppm)

Compound 5b

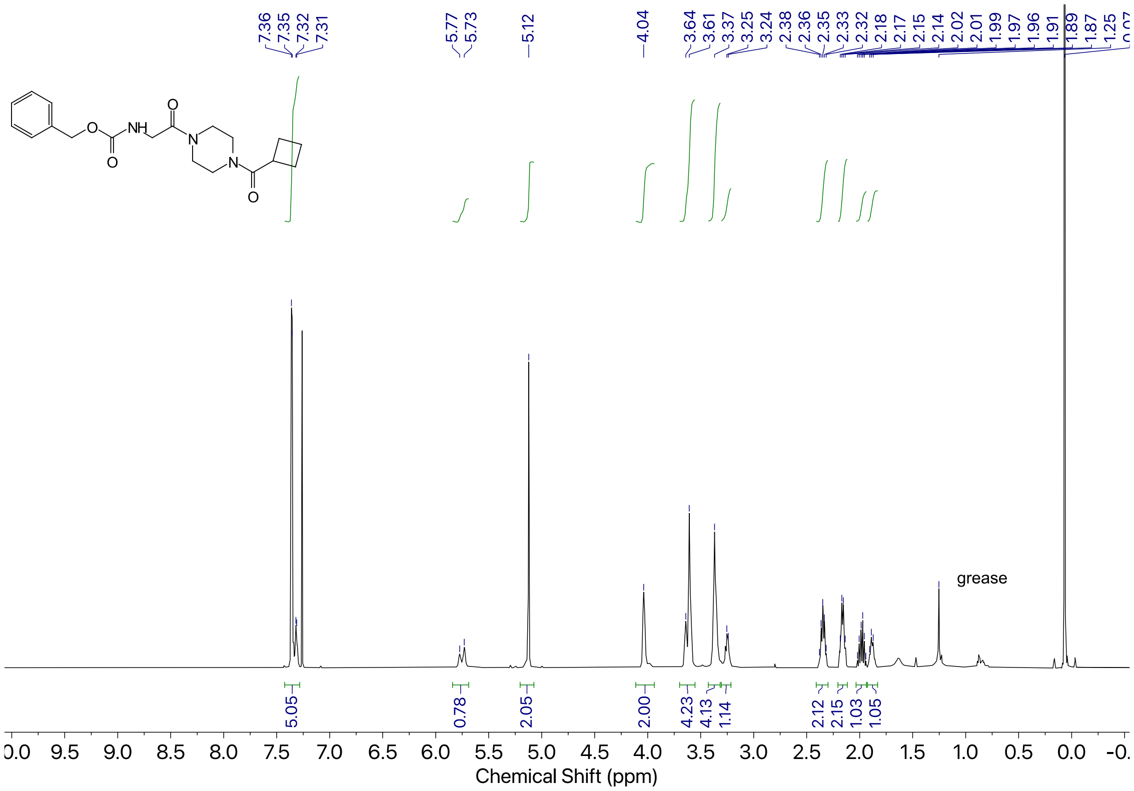

7.36
7.35
7.32
7.31
5.77
5.73
5.12
4.04
3.64
3.61
3.37
3.25
3.24
2.38
2.36
2.35
2.33
2.32
2.18
2.17
2.15
2.14
2.02
2.01
1.99
1.97
1.96
1.91
1.89
1.87
1.25
grease
5.05
0.78
2.05
2.00
4.23
4.13
1.14
2.12
2.15
1.03
1.05
Chemical Shift (ppm)

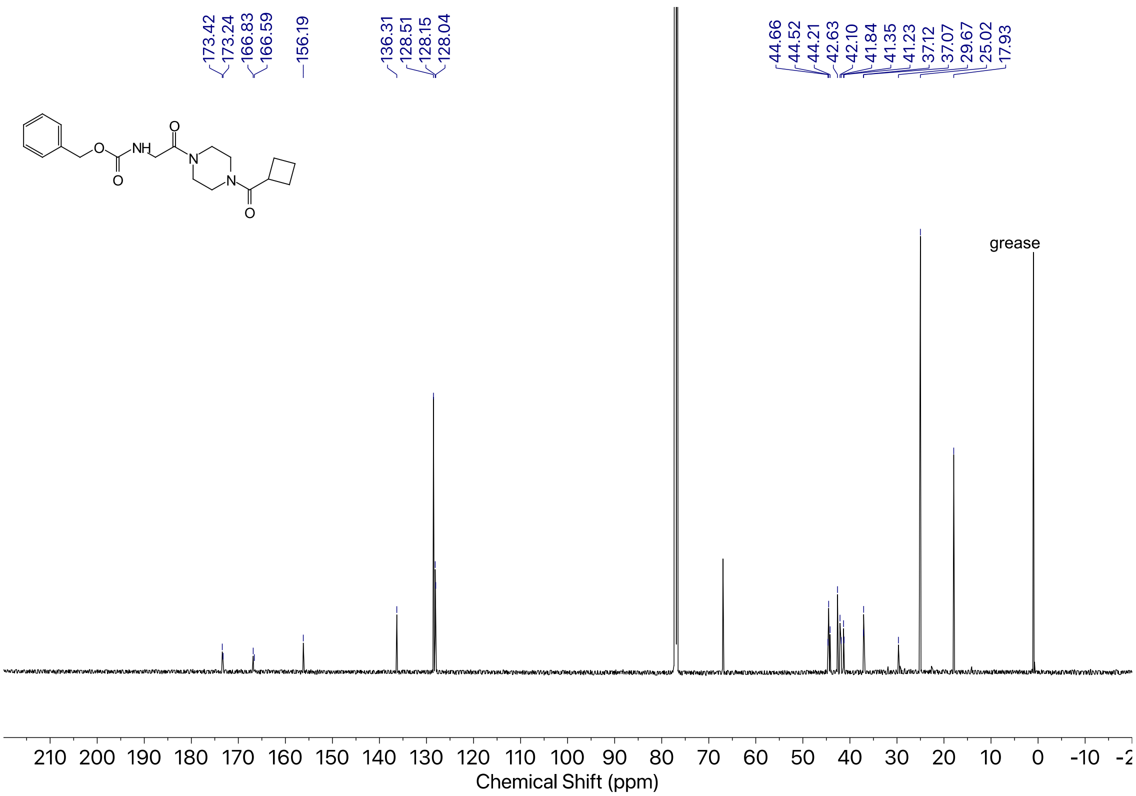

173.42
173.24
166.83
166.59
156.19
136.31
128.51
128.15
128.04
44.66
44.52
44.21
42.63
42.10
41.84
41.35
41.23
37.12
37.07
29.67
25.02
17.93
grease
Chemical Shift (ppm)

Compound 5c

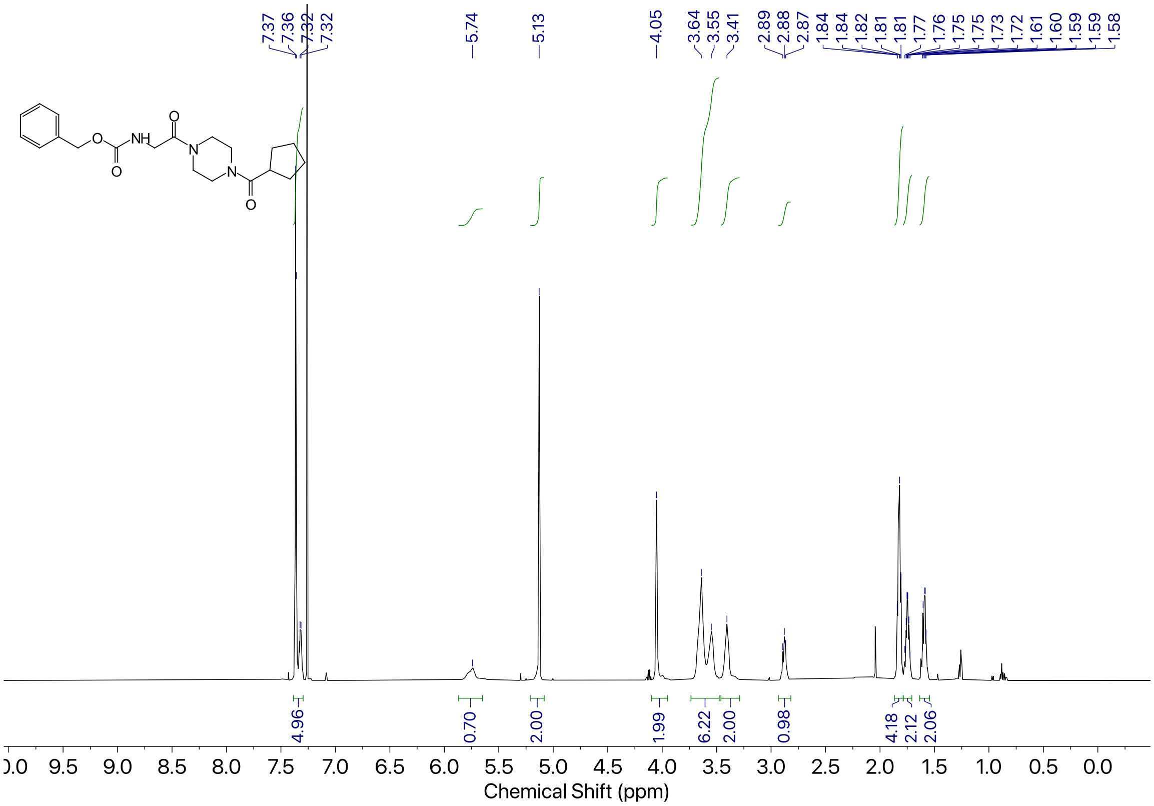
7.37
7.36
7.32
7.32
5.74
5.13
4.05
3.64
3.55
3.41
2.89
2.88
2.87
1.84
1.84
1.82
1.81
1.81
1.77
1.76
1.75
1.75
1.73
1.72
1.61
1.60
1.59
1.59
1.58
4.96
0.70
2.00
1.99
6.22
2.00
0.98
4.18
2.12
2.06
Chemical Shift (ppm)

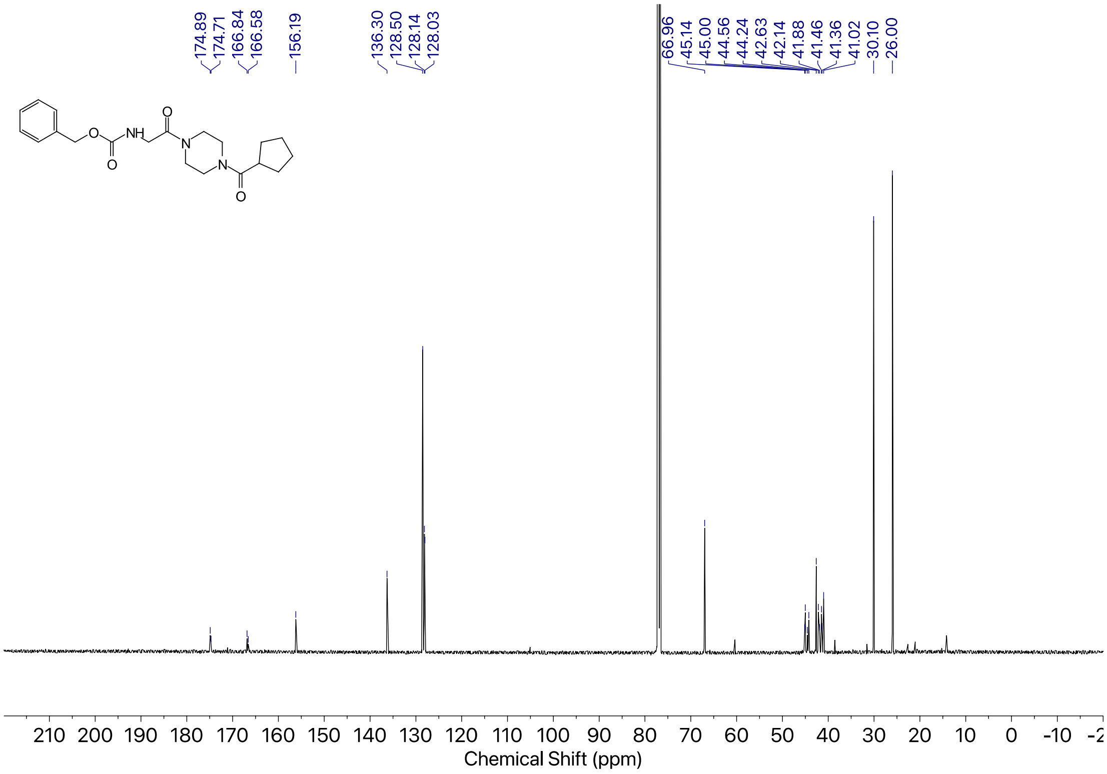
174.89
174.71
166.84
166.58
156.19
136.30
128.50
128.14
128.03
66.96
45.14
45.00
44.56
44.24
42.63
42.14
41.88
41.46
41.36
41.02
30.10
26.00
Chemical Shift (ppm)

Compound 5d

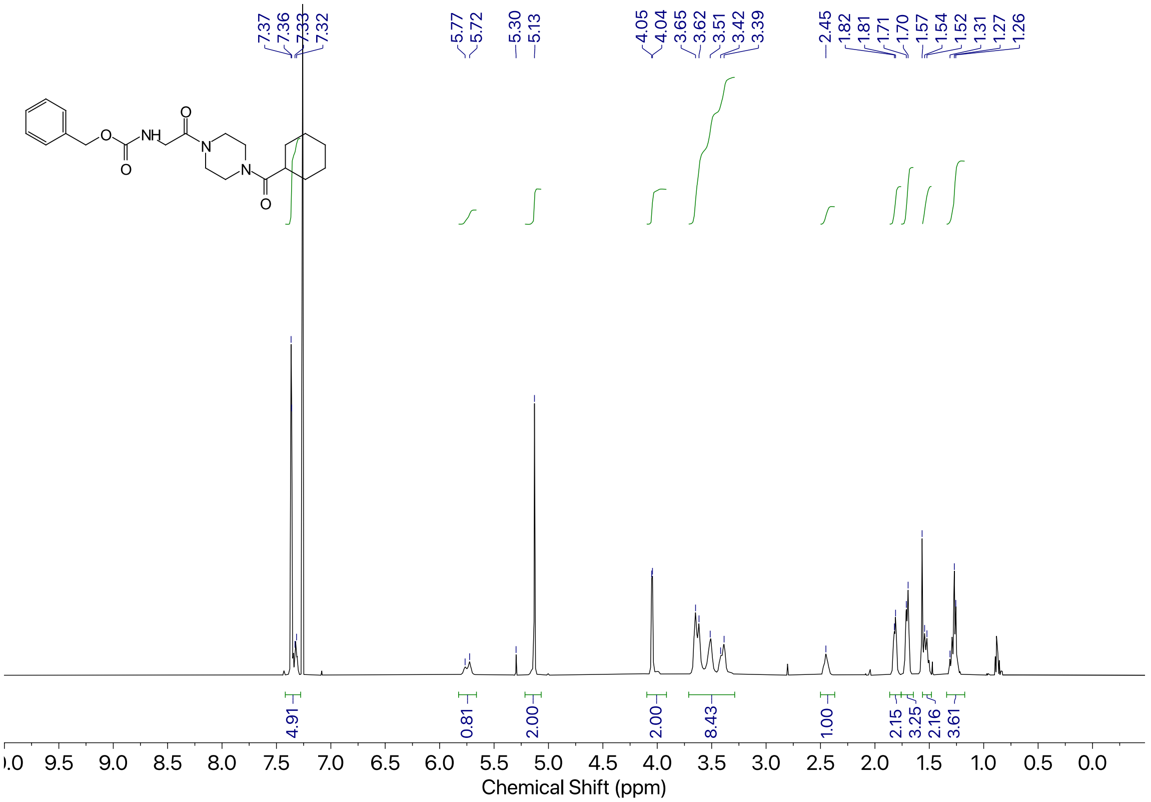

7.37
7.36
7.33
7.32
5.77
5.72
5.30
5.13
4.05
4.04
3.65
3.62
3.51
3.42
3.39
2.45
1.82
1.81
1.71
1.70
1.57
1.54
1.52
1.31
1.27
1.26
4.91
0.81
2.00
2.00
8.43
1.00
2.15
3.25
2.16
3.61
Chemical Shift (ppm)

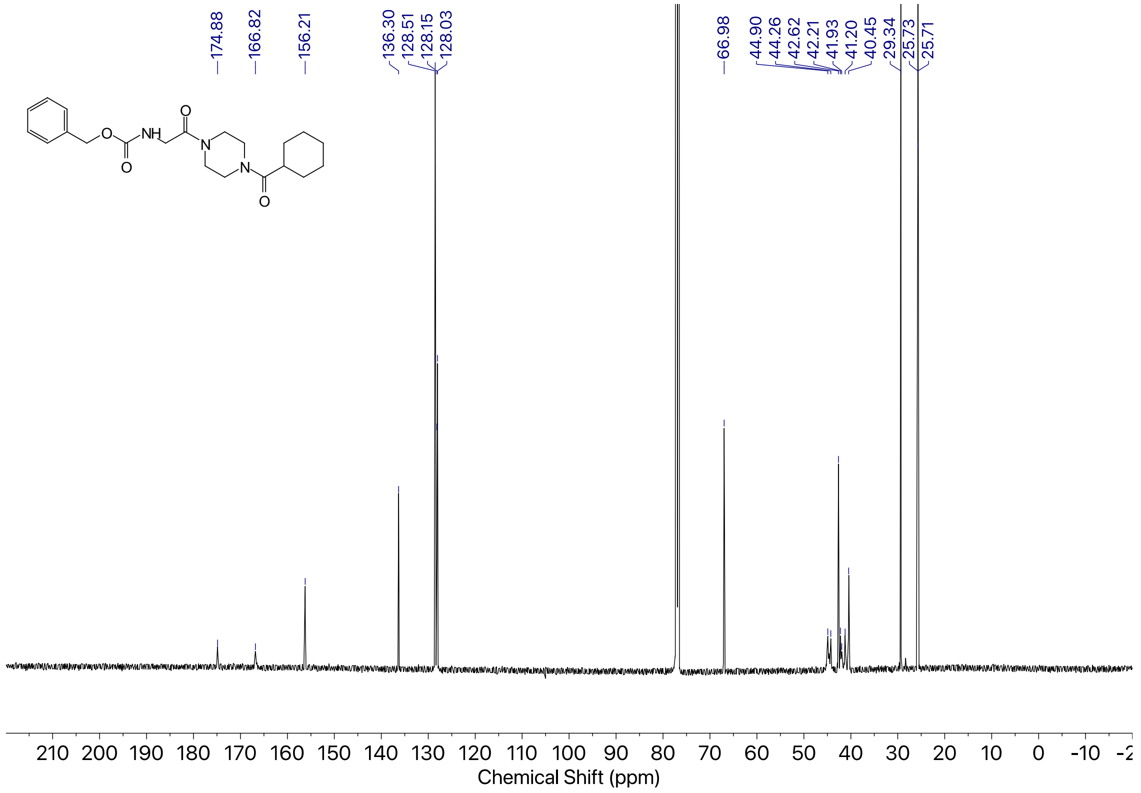

174.88
166.82
156.21
136.30
128.51
128.15
128.03
66.98
44.90
44.26
42.62
42.21
41.93
41.20
40.45
29.34
25.73
25.71
Chemical Shift (ppm)

Compound 5e

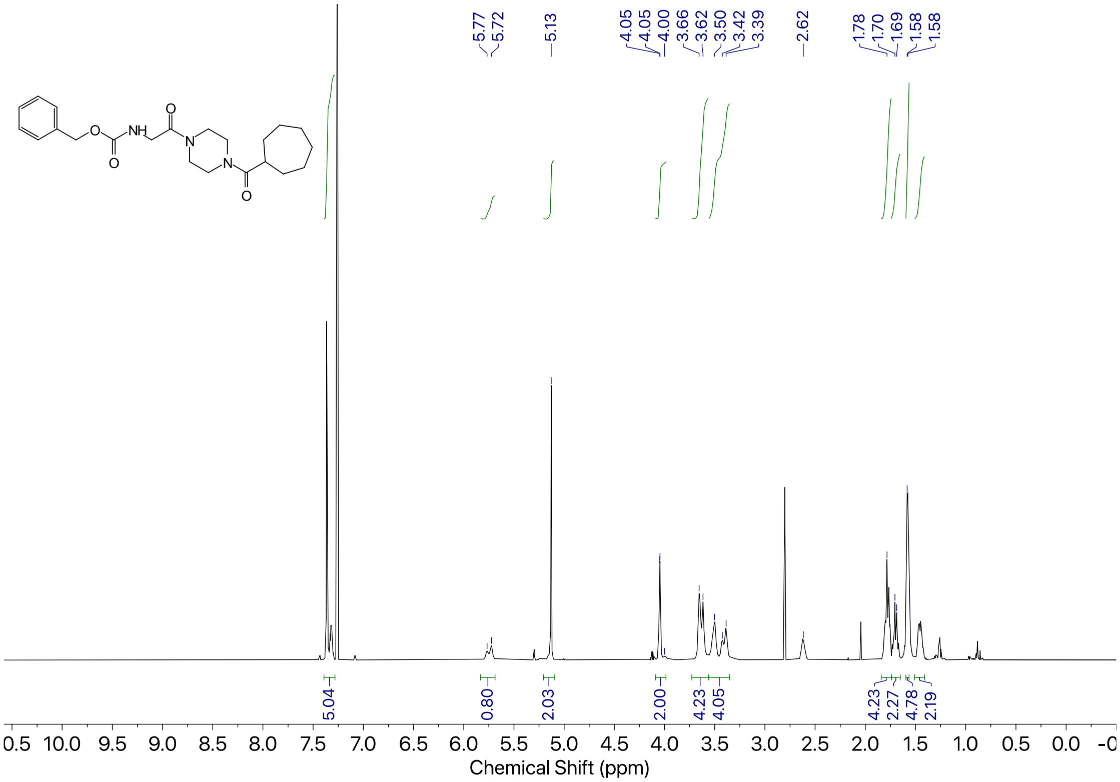

5.77
5.72
5.13
4.05
4.05
4.00
3.66
3.62
3.50
3.42
3.39
2.62
1.78
1.70
1.69
1.58
1.58
O
NH
O
O
N
N
O
5.04
0.80
2.03
2.00
4.23
4.05
4.23
2.27
4.78
2.19
0.5
10.0
9.5
9.0
8.5
8.0
7.5
7.0
6.5
6.0
5.5
5.0
4.5
4.0
3.5
3.0
2.5
2.0
1.5
1.0
0.5
0.0
Chemical Shift (ppm)

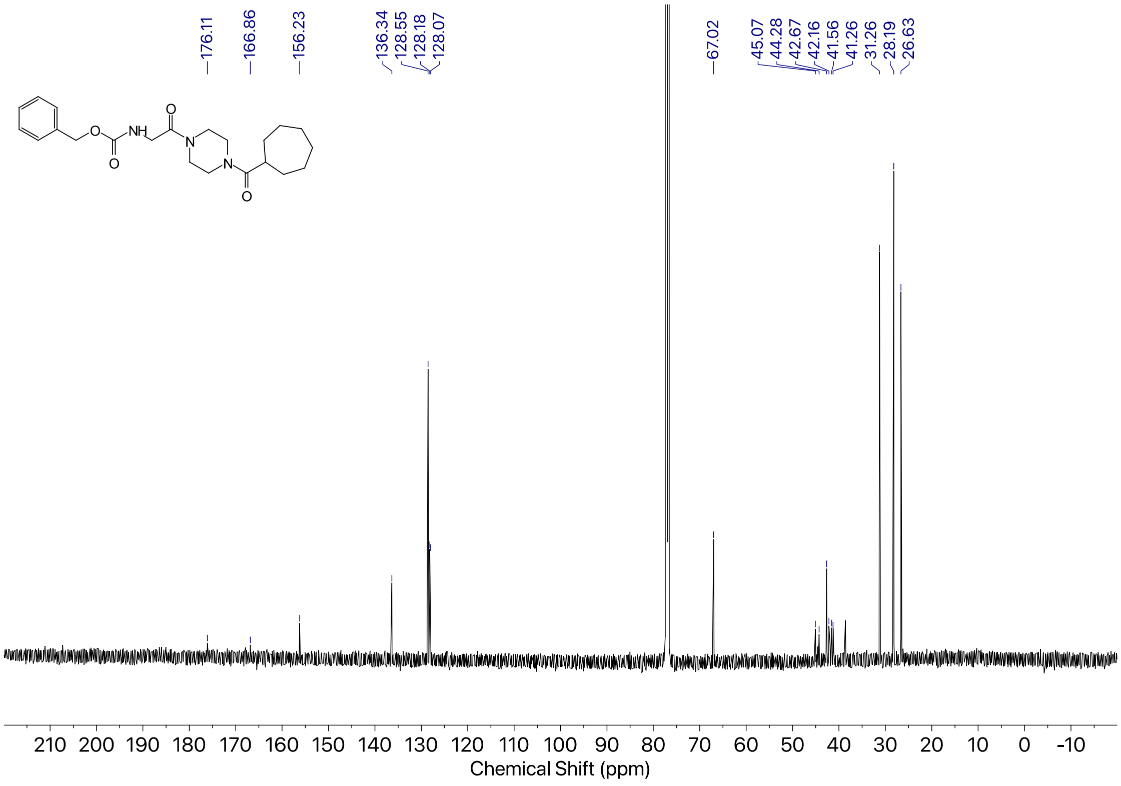

176.11
166.86
156.23
136.34
128.55
128.18
128.07
67.02
45.07
44.28
42.67
42.16
41.56
41.26
31.26
28.19
26.63
O
NH
O
O
N
N
O
210
200
190
180
170
160
150
140
130
120
110
100
90
80
70
60
50
40
30
20
10
0
-10
Chemical Shift (ppm)

Compound 5f

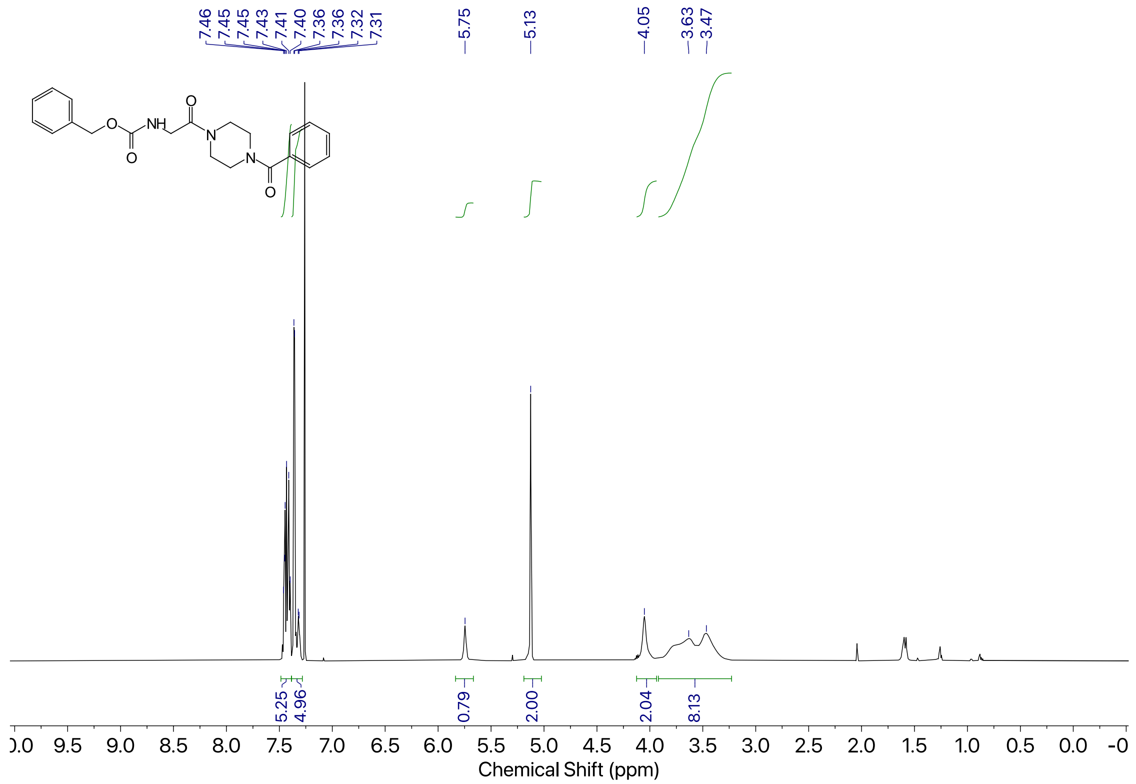
7.46
7.45
7.45
7.43
7.41
7.40
7.36
7.36
7.32
7.31
5.75
5.13
4.05
3.63
3.47
5.25
4.96
0.79
2.00
2.04
8.13
Chemical Shift (ppm)

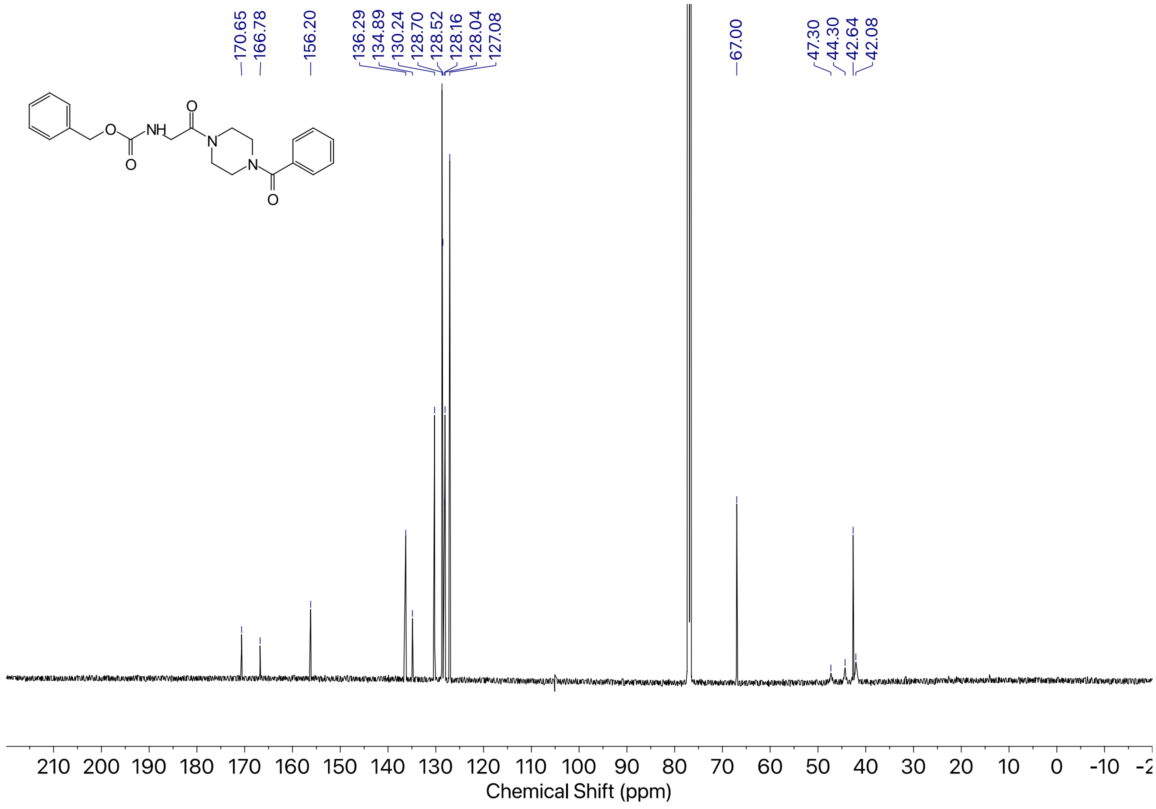
170.65
166.78
156.20
136.29
134.89
130.24
128.70
128.52
128.16
128.04
127.08
67.00
47.30
44.30
42.64
42.08
Chemical Shift (ppm)

Compound 6a

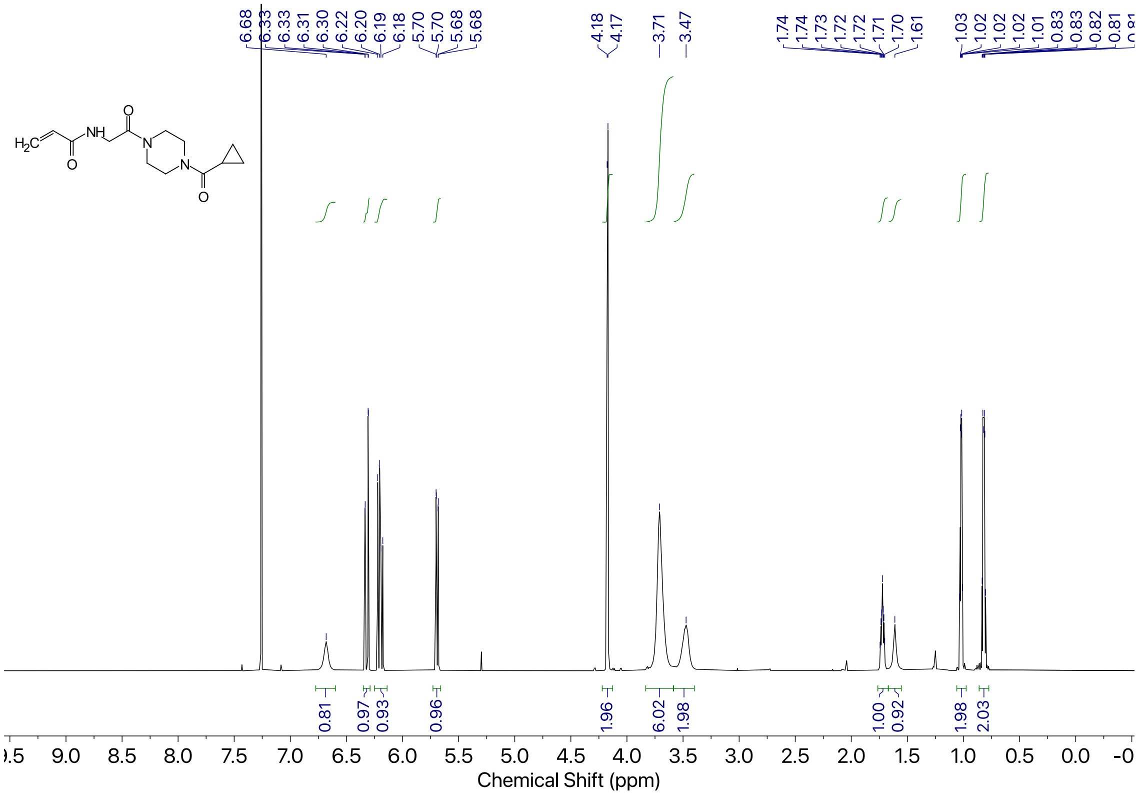
6.68
6.33
6.33
6.31
6.30
6.22
6.20
6.19
6.18
5.70
5.70
5.68
5.68
4.18
4.17
3.71
3.47
1.74
1.74
1.73
1.72
1.72
1.71
1.70
1.61
1.03
1.02
1.02
1.02
1.01
0.83
0.83
0.82
0.81
H2C
NH
O
O
N
N
O
0.81
0.97
0.93
0.96
1.96
6.02
1.98
1.00
0.92
1.98
2.03
9.0 8.5 8.0 7.5 7.0 6.5 6.0 5.5 5.0 4.5 4.0 3.5 3.0 2.5 2.0 1.5 1.0 0.5 0.0
Chemical Shift (ppm)

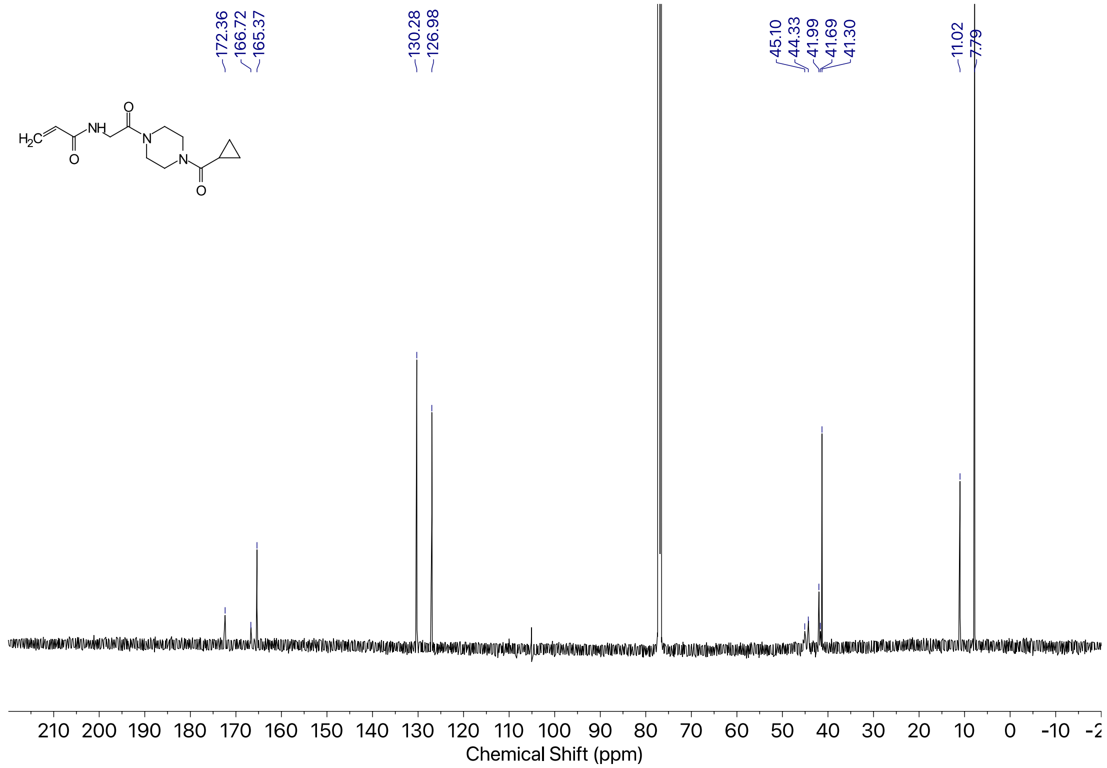
172.36
166.72
165.37
130.28
126.98
45.10
44.33
41.99
41.69
41.30
11.02
7.79
H2C
NH
O
O
N
N
O
210 200 190 180 170 160 150 140 130 120 110 100 90 80 70 60 50 40 30 20 10 0 -10
Chemical Shift (ppm)

Compound 6b

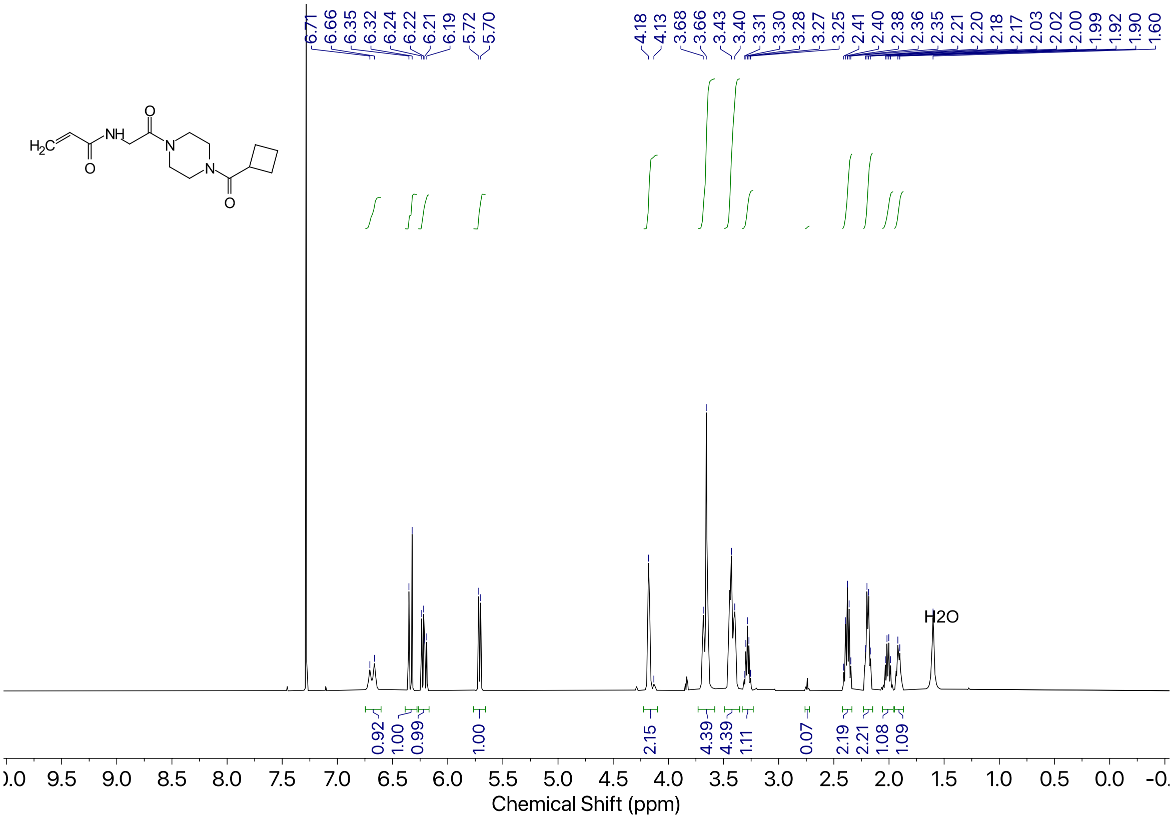

6.71
6.66
6.35
6.32
6.24
6.22
6.21
6.19
5.72
5.70
4.18
4.13
3.68
3.66
3.43
3.40
3.31
3.30
3.28
3.27
3.25
2.41
2.40
2.38
2.36
2.35
2.21
2.20
2.18
2.17
2.03
2.02
2.00
1.99
1.92
1.90
1.60
H2O
0.92
1.00
0.99
1.00
2.15
4.39
4.39
1.11
0.07
2.19
2.21
1.08
1.09
Chemical Shift (ppm)

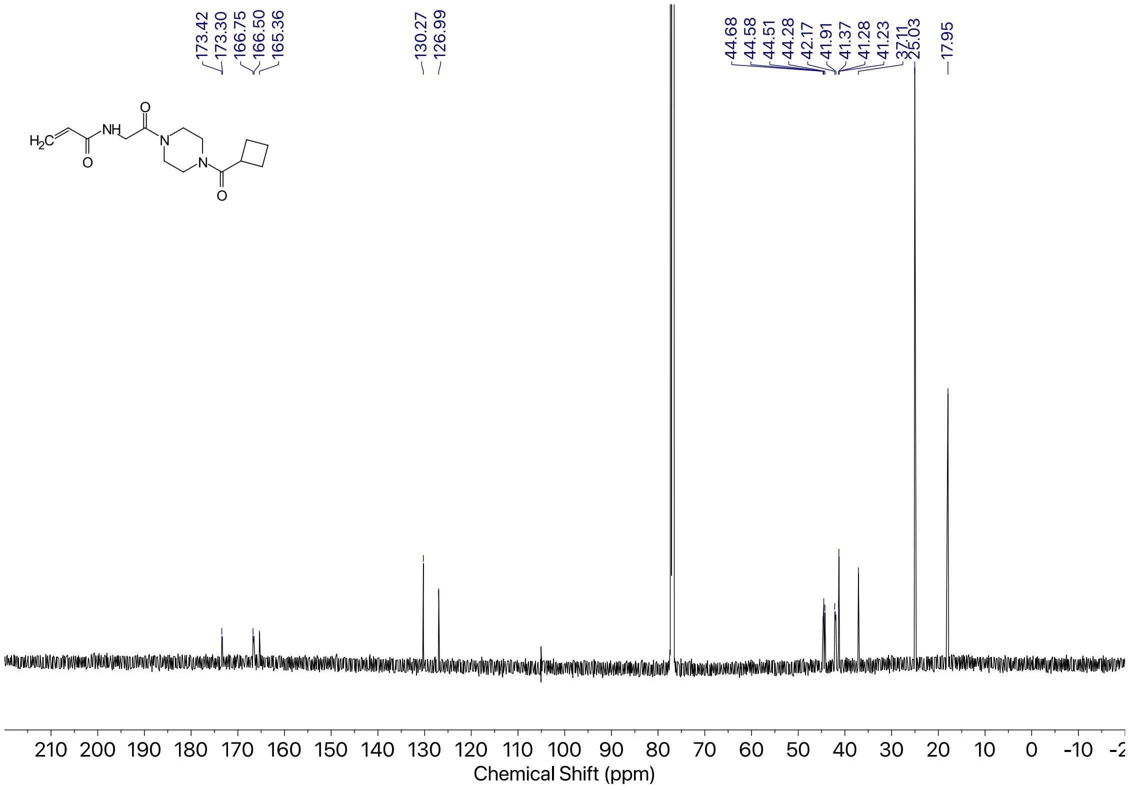

173.42
173.30
166.75
166.50
165.36
130.27
126.99
44.68
44.58
44.51
44.28
42.17
41.91
41.37
41.28
41.23
37.11
25.03
17.95
Chemical Shift (ppm)

Compound 6c

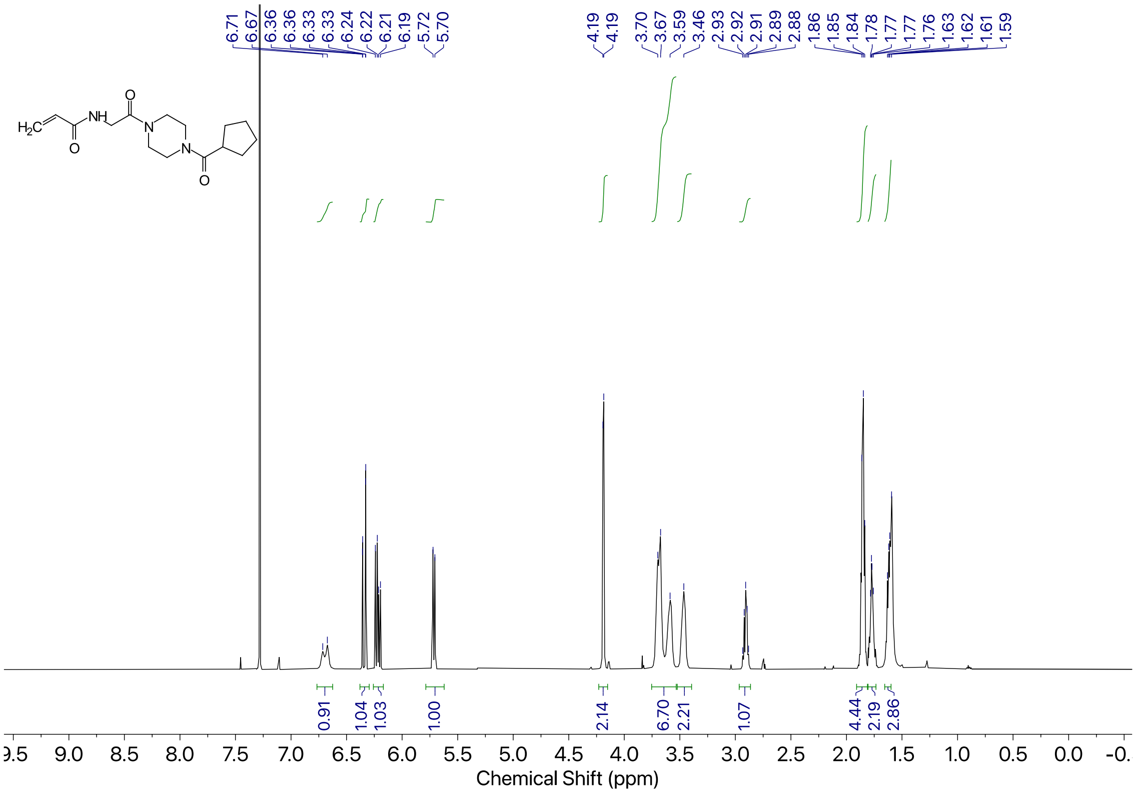

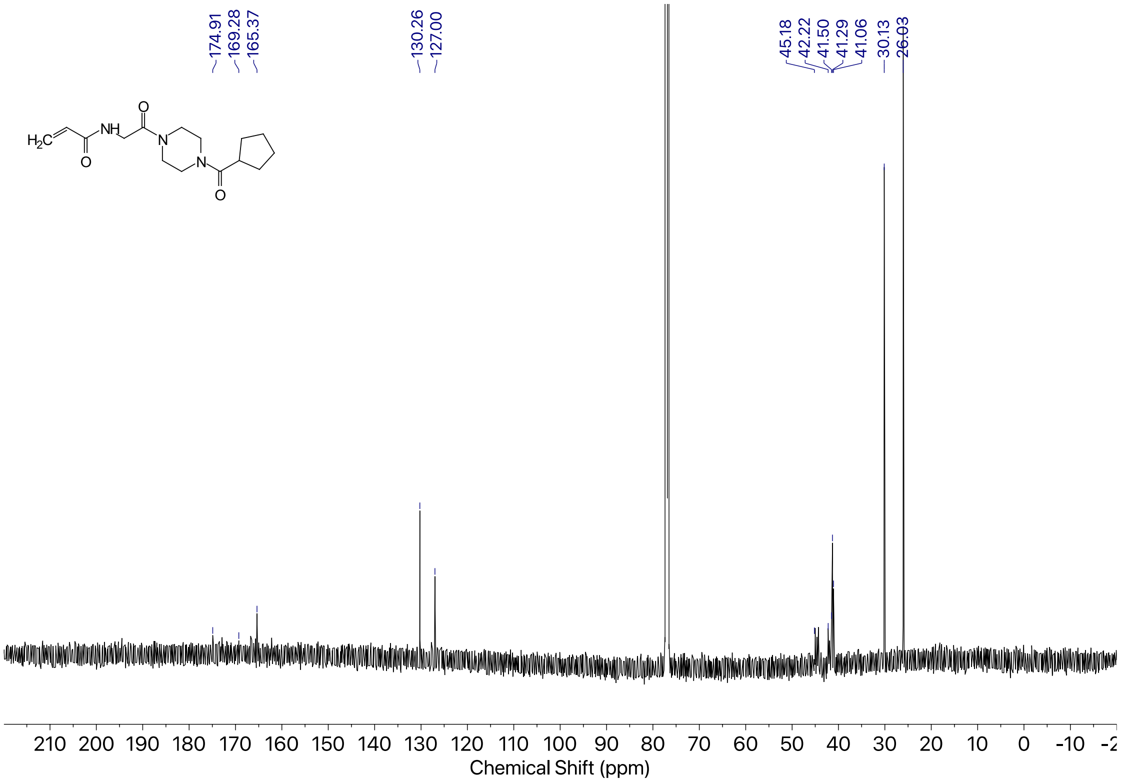

Compound 6d

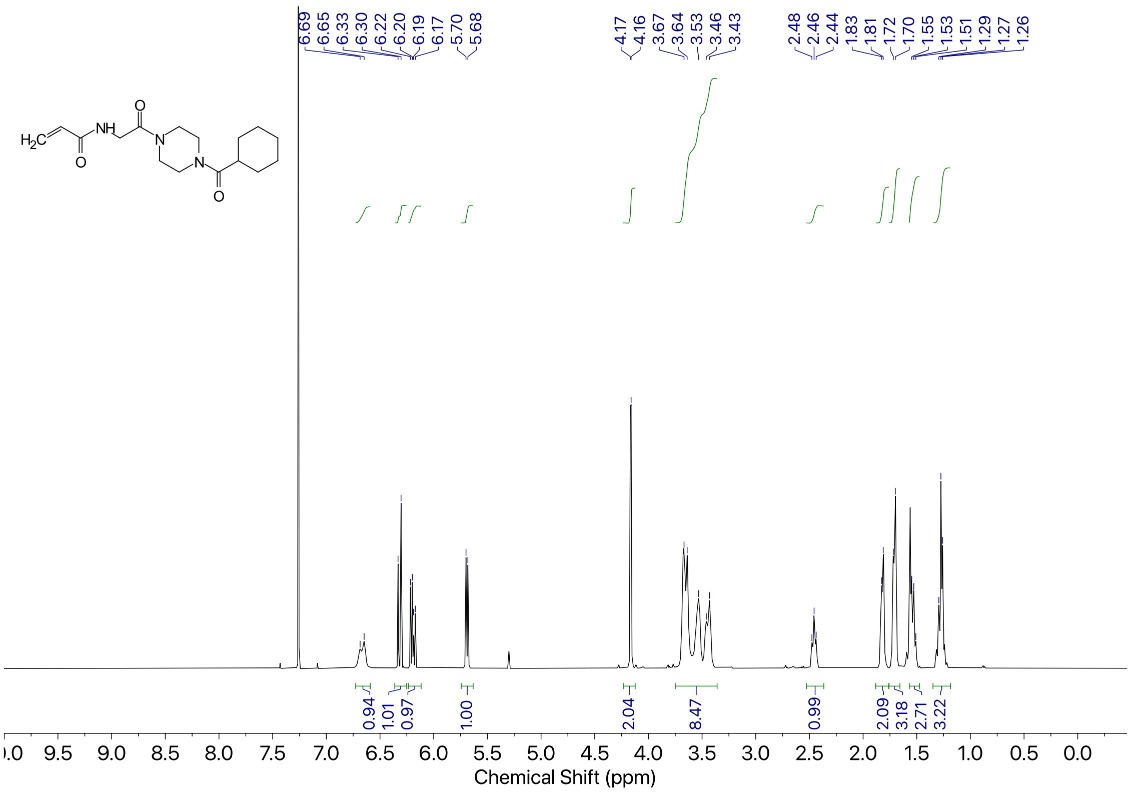

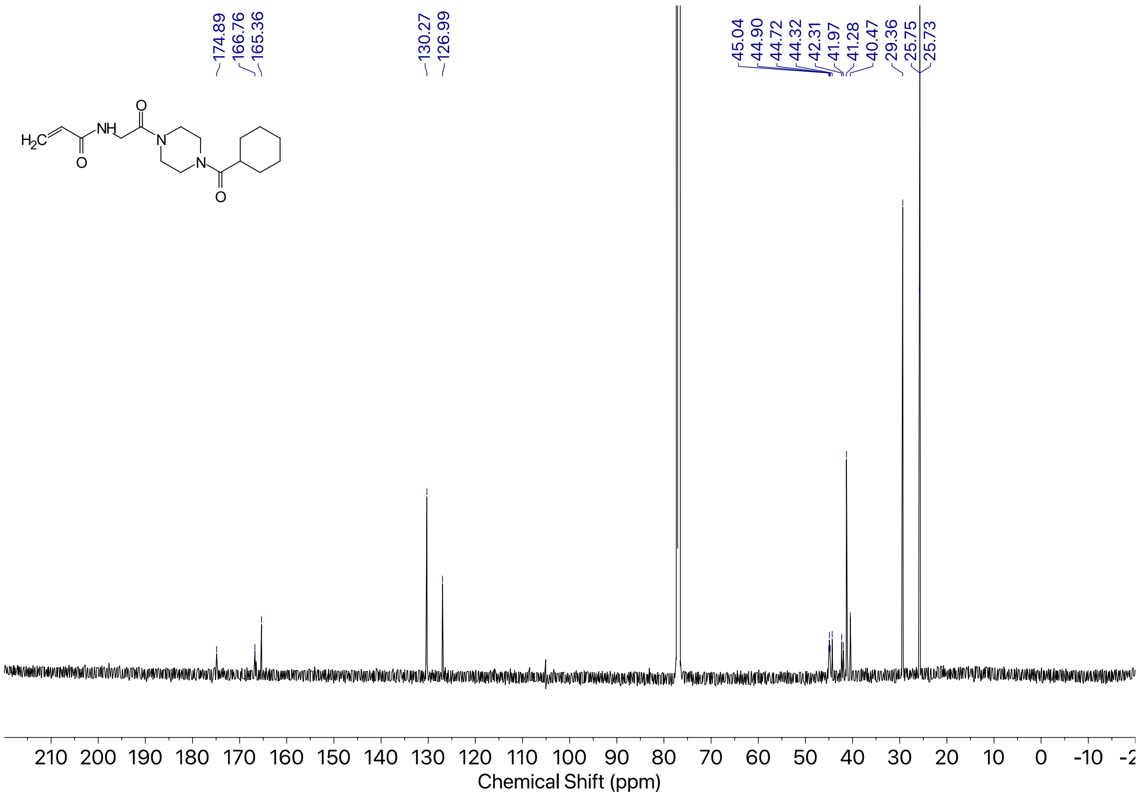

Compound 6e

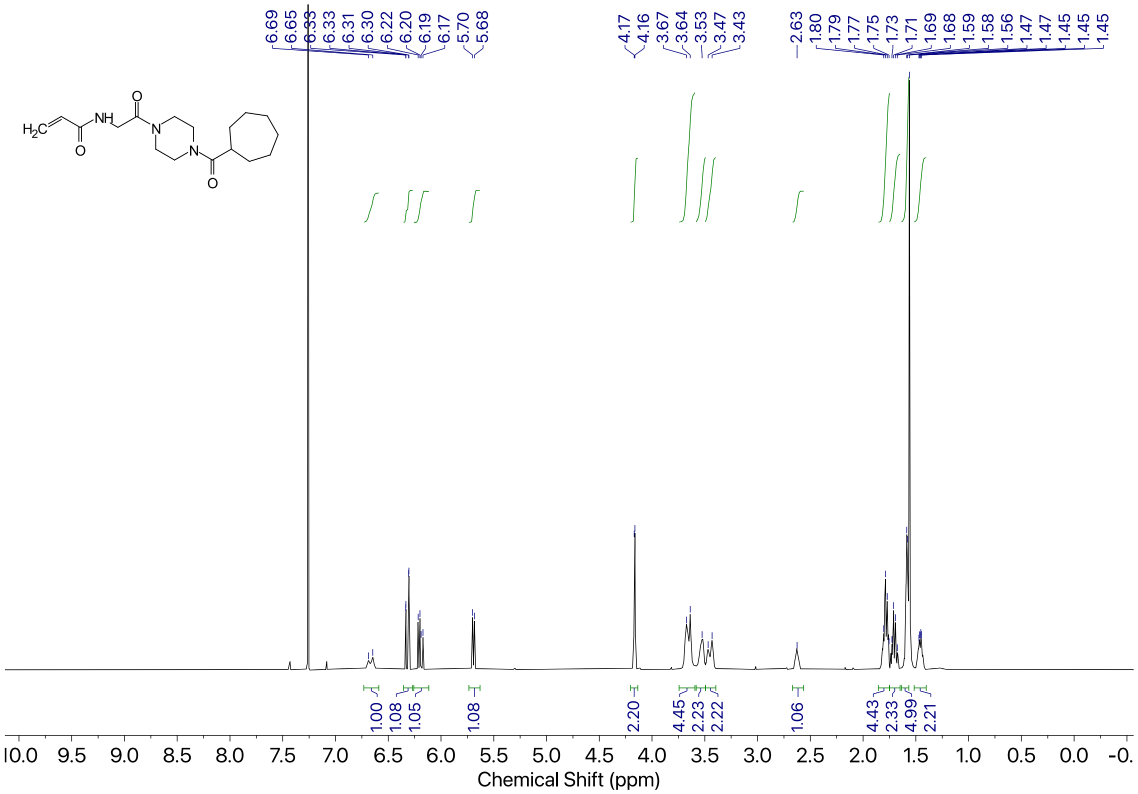

6.69
6.65
6.33
6.33
6.31
6.30
6.22
6.20
6.19
6.17
5.70
5.68
4.17
4.16
3.67
3.64
3.53
3.47
3.43
2.63
1.80
1.79
1.77
1.75
1.73
1.71
1.69
1.68
1.59
1.58
1.56
1.47
1.47
1.45
1.45
1.45
1.00
1.08
1.05
1.08
2.20
4.45
2.23
2.22
1.06
4.43
2.33
4.99
2.21
Chemical Shift (ppm)

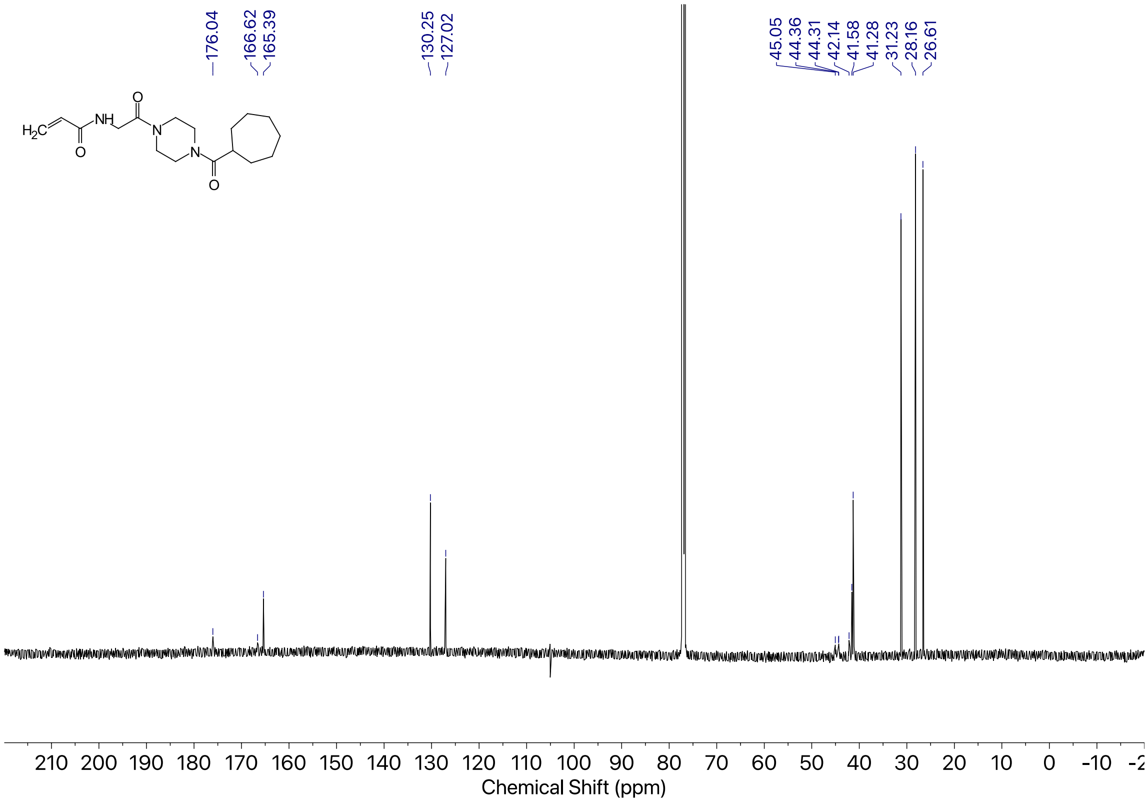

176.04
166.62
165.39
130.25
127.02
45.05
44.36
44.31
42.14
41.58
41.28
31.23
28.16
26.61
Chemical Shift (ppm)

Compound 6f

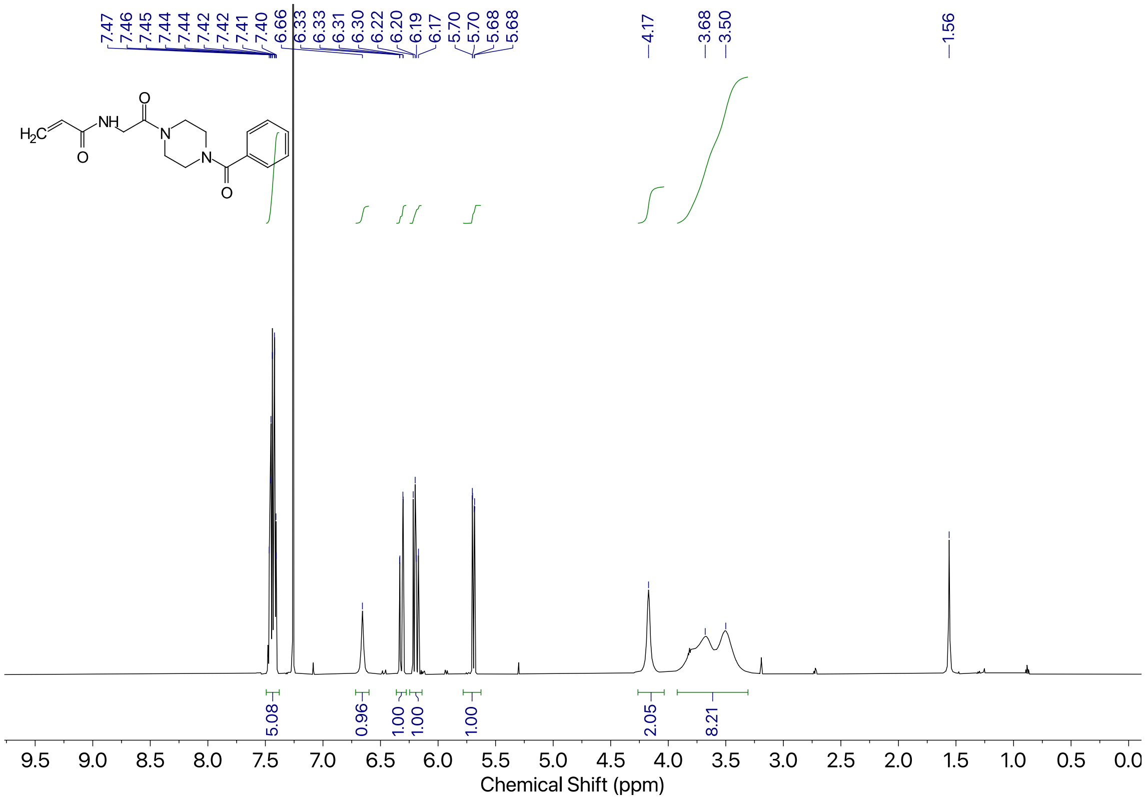

7.47
7.46
7.45
7.44
7.44
7.42
7.42
7.41
7.40
6.66
6.33
6.33
6.31
6.30
6.22
6.20
6.19
6.17
5.70
5.70
5.68
5.68
4.17
3.68
3.50
1.56
5.08
0.96
1.00
1.00
1.00
2.05
8.21
9.5 9.0 8.5 8.0 7.5 7.0 6.5 6.0 5.5 5.0 4.5 4.0 3.5 3.0 2.5 2.0 1.5 1.0 0.5 0.0
Chemical Shift (ppm)

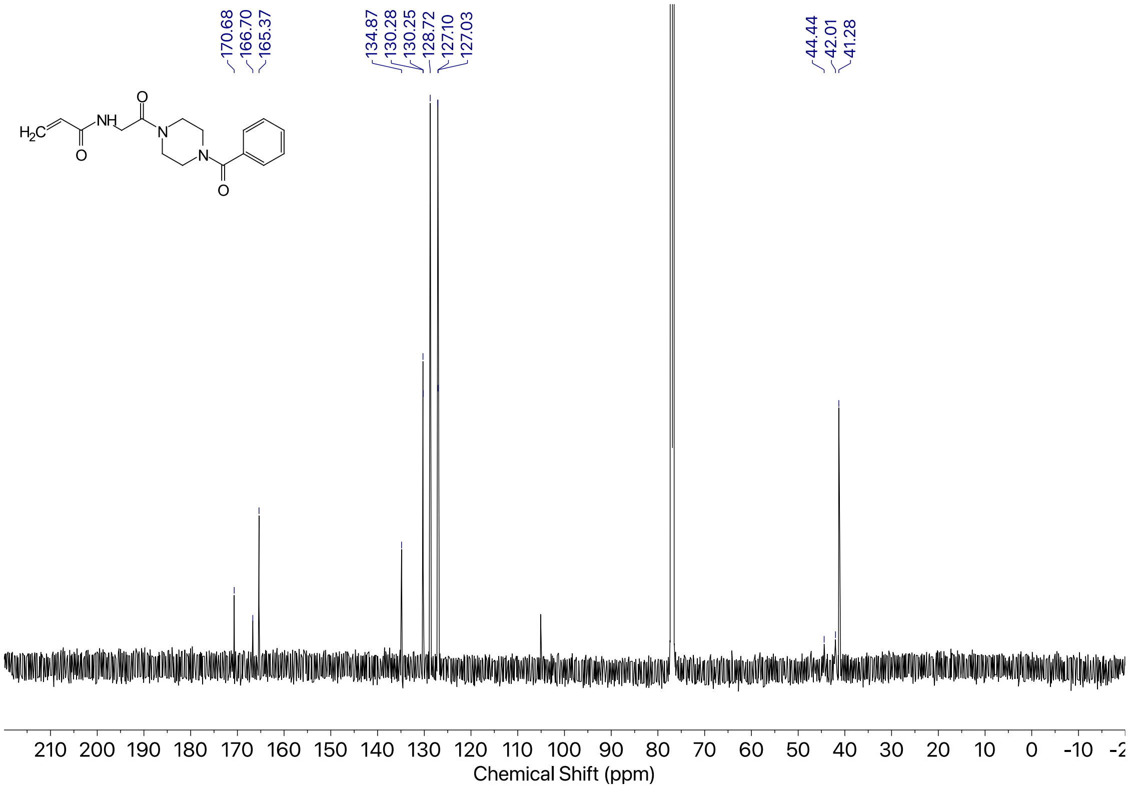

170.68
166.70
165.37
134.87
130.28
130.25
128.72
127.10
127.03
44.44
42.01
41.28
210 200 190 180 170 160 150 140 130 120 110 100 90 80 70 60 50 40 30 20 10 0 -10
Chemical Shift (ppm)

Compound 7a

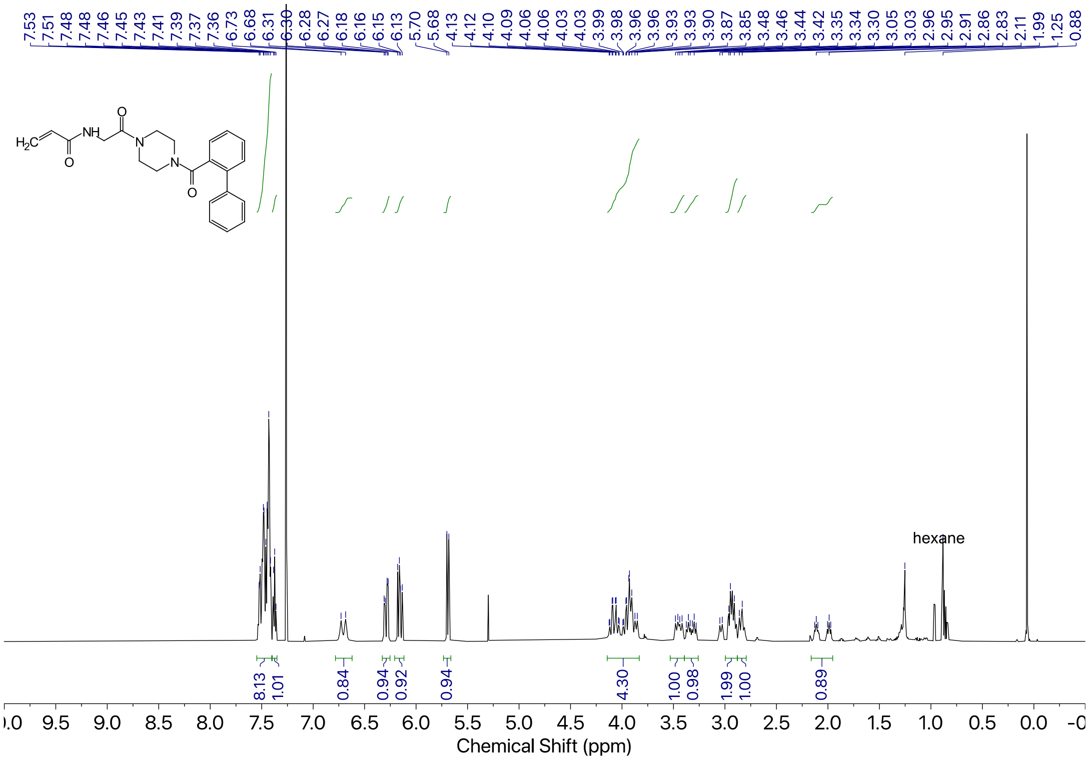
7.53
7.51
7.48
7.48
7.46
7.45
7.43
7.41
7.39
7.37
7.36
6.73
6.68
6.31
6.30
6.28
6.27
6.18
6.16
6.15
6.13
5.70
5.68
4.13
4.12
4.10
4.09
4.06
4.06
4.03
4.03
3.99
3.98
3.96
3.96
3.93
3.93
3.90
3.87
3.85
3.48
3.46
3.44
3.42
3.35
3.34
3.30
3.05
3.03
2.96
2.95
2.91
2.86
2.83
2.11
1.99
1.25
0.88
H2C
NH
O
N
N
hexane
8.13
1.01
0.84
0.94
0.92
0.94
4.30
1.00
0.98
1.99
1.00
0.89
Chemical Shift (ppm)

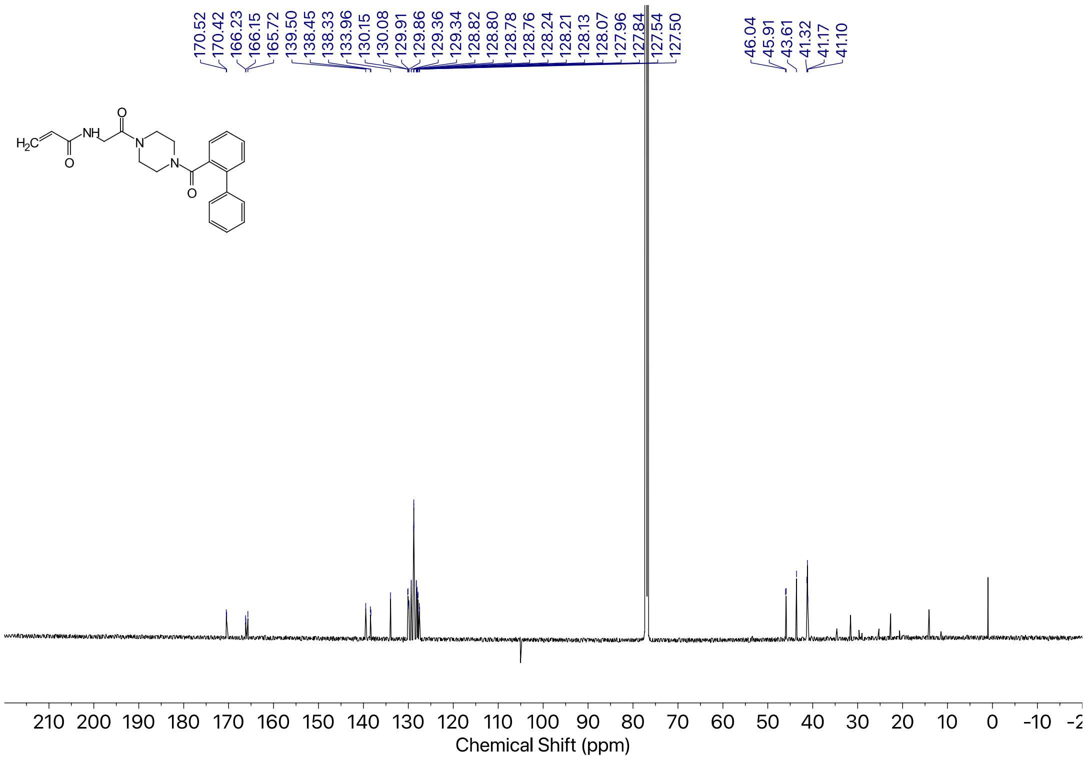
170.52
170.42
166.23
166.15
165.72
139.50
138.45
138.33
133.96
130.15
130.08
129.91
129.86
129.36
129.34
128.82
128.80
128.78
128.76
128.24
128.21
128.13
128.07
127.96
127.84
127.54
127.50
46.04
45.91
43.61
41.32
41.17
41.10
H2C
NH
O
N
N
Chemical Shift (ppm)

Compound 7b

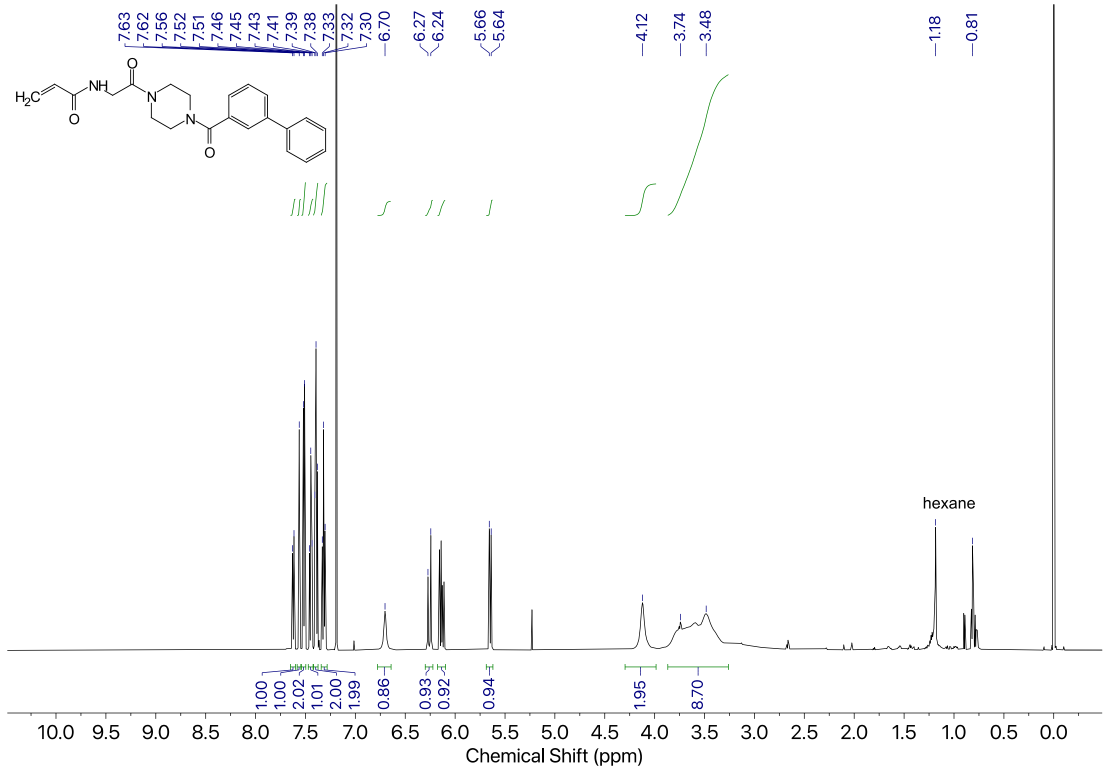
7.63
7.62
7.56
7.52
7.51
7.46
7.45
7.43
7.41
7.39
7.38
7.33
7.32
7.30
6.70
6.27
6.24
5.66
5.64
4.12
3.74
3.48
1.18
0.81
hexane
1.00
1.00
2.02
1.01
2.00
1.99
0.86
0.93
0.92
0.94
1.95
8.70
10.0 9.5 9.0 8.5 8.0 7.5 7.0 6.5 6.0 5.5 5.0 4.5 4.0 3.5 3.0 2.5 2.0 1.5 1.0 0.5 0.0
Chemical Shift (ppm)

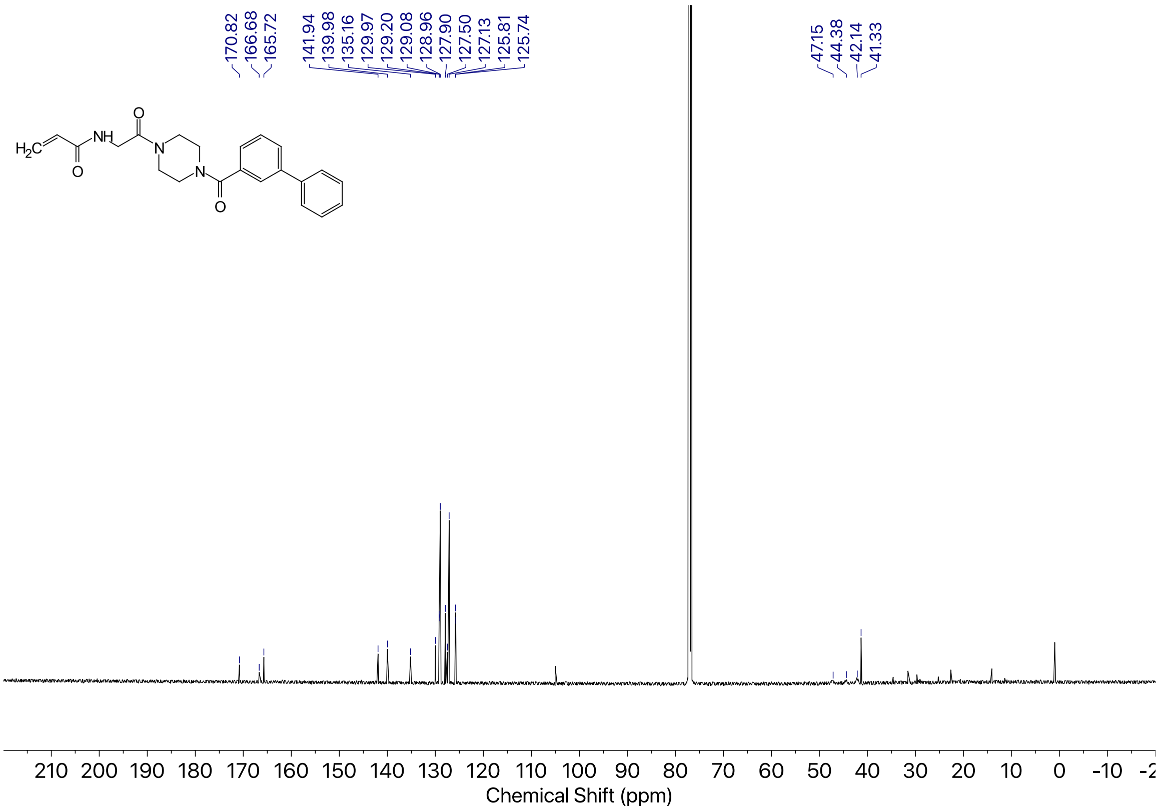
170.82
166.68
165.72
141.94
139.98
135.16
129.97
129.20
129.08
128.96
127.90
127.50
127.13
125.81
125.74
47.15
44.38
42.14
41.33
210 200 190 180 170 160 150 140 130 120 110 100 90 80 70 60 50 40 30 20 10 0 -10
Chemical Shift (ppm)

Compound 7c

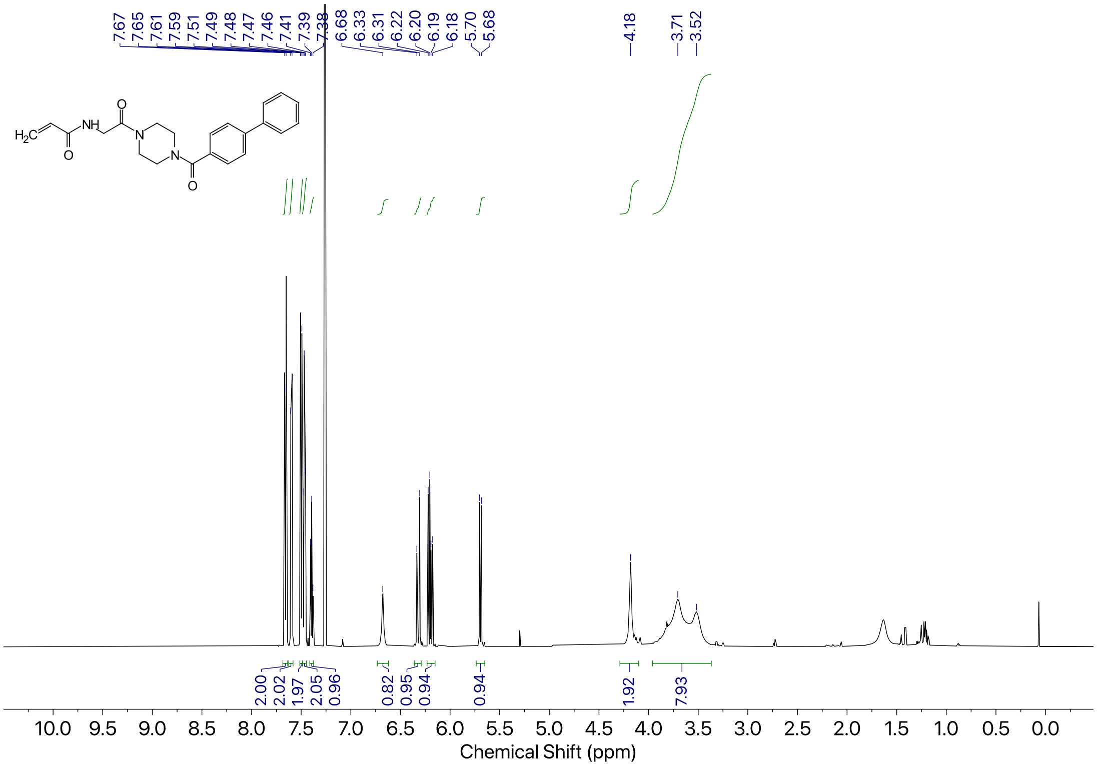

7.67
7.65
7.61
7.59
7.51
7.49
7.48
7.47
7.46
7.41
7.39
7.38
6.68
6.33
6.31
6.22
6.20
6.19
6.18
5.70
5.68
4.18
3.71
3.52
NH
O
O
N
N
O
2.00
2.02
1.97
2.05
0.96
0.82
0.95
0.94
0.94
1.92
7.93
10.0 9.5 9.0 8.5 8.0 7.5 7.0 6.5 6.0 5.5 5.0 4.5 4.0 3.5 3.0 2.5 2.0 1.5 1.0 0.5 0.0
Chemical Shift (ppm)

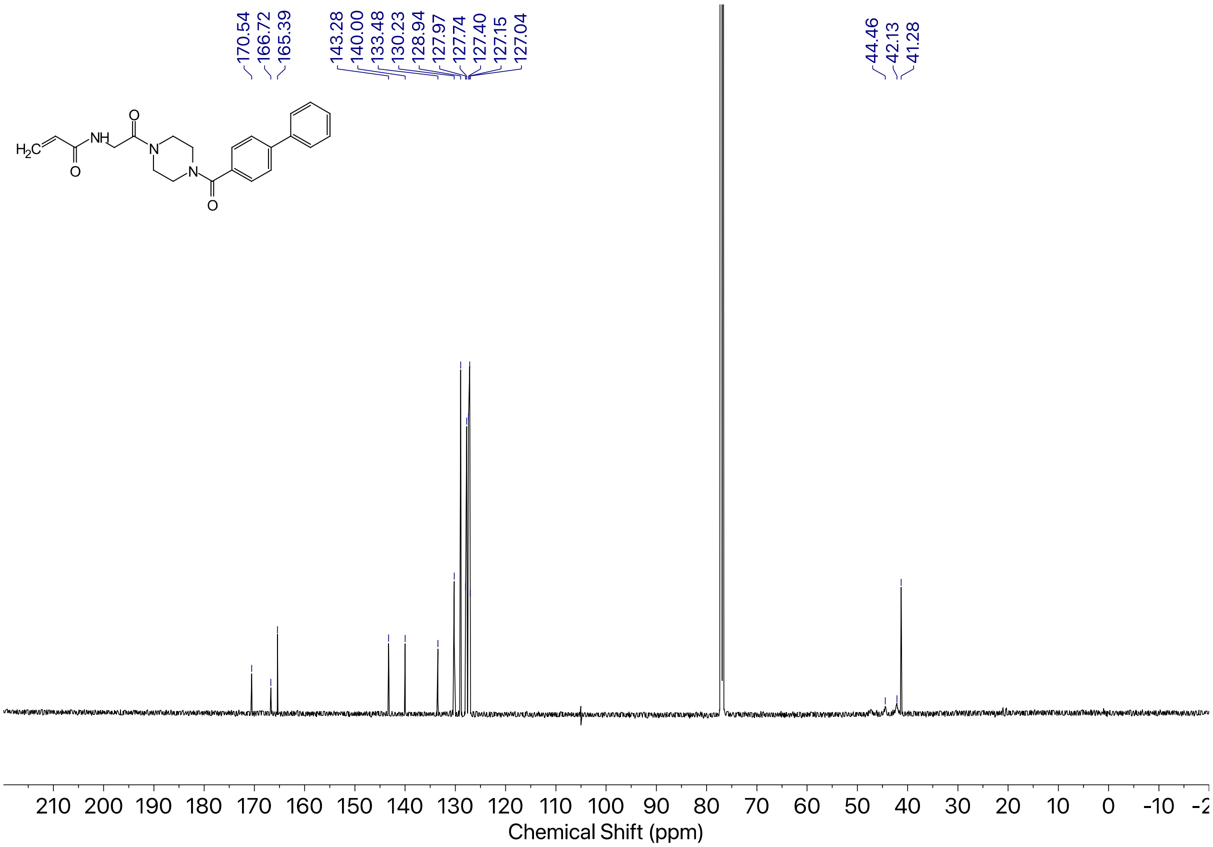

170.54
166.72
165.39
143.28
140.00
133.48
130.23
128.94
127.97
127.74
127.40
127.15
127.04
44.46
42.13
41.28
NH
O
O
N
N
O
210 200 190 180 170 160 150 140 130 120 110 100 90 80 70 60 50 40 30 20 10 0 -10
Chemical Shift (ppm)

Compound 10a

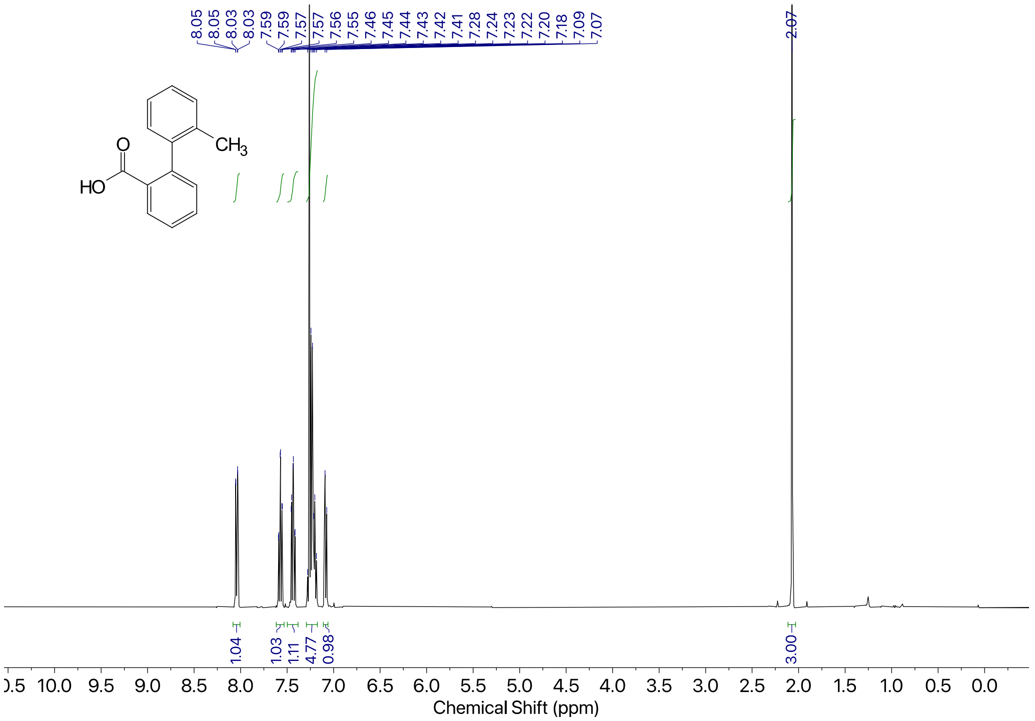

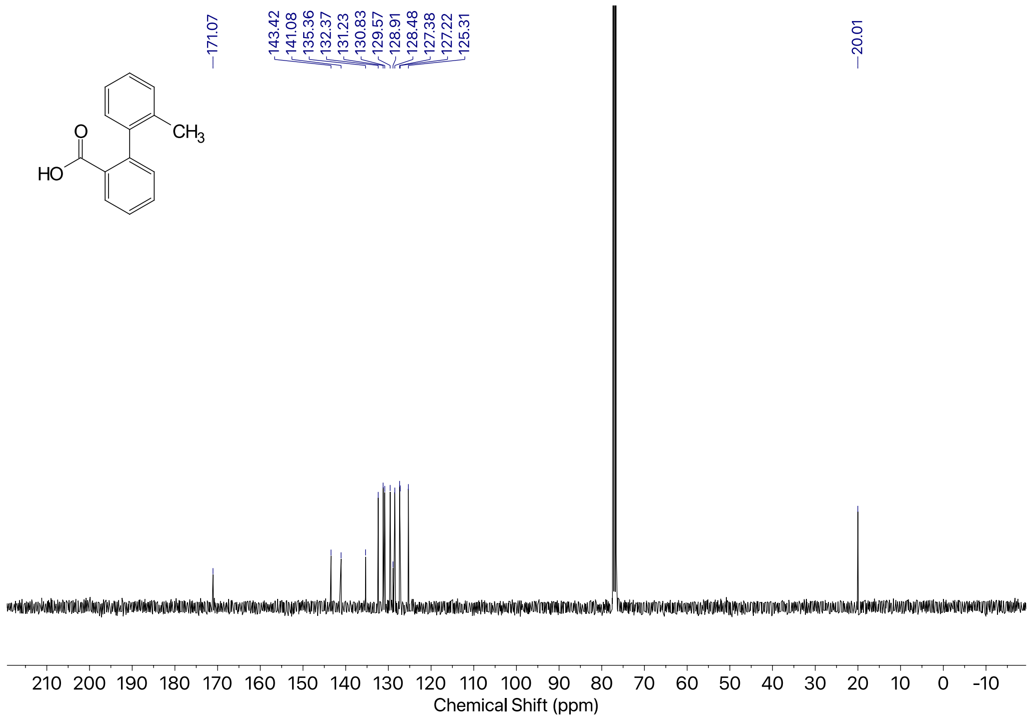

Compound 10b

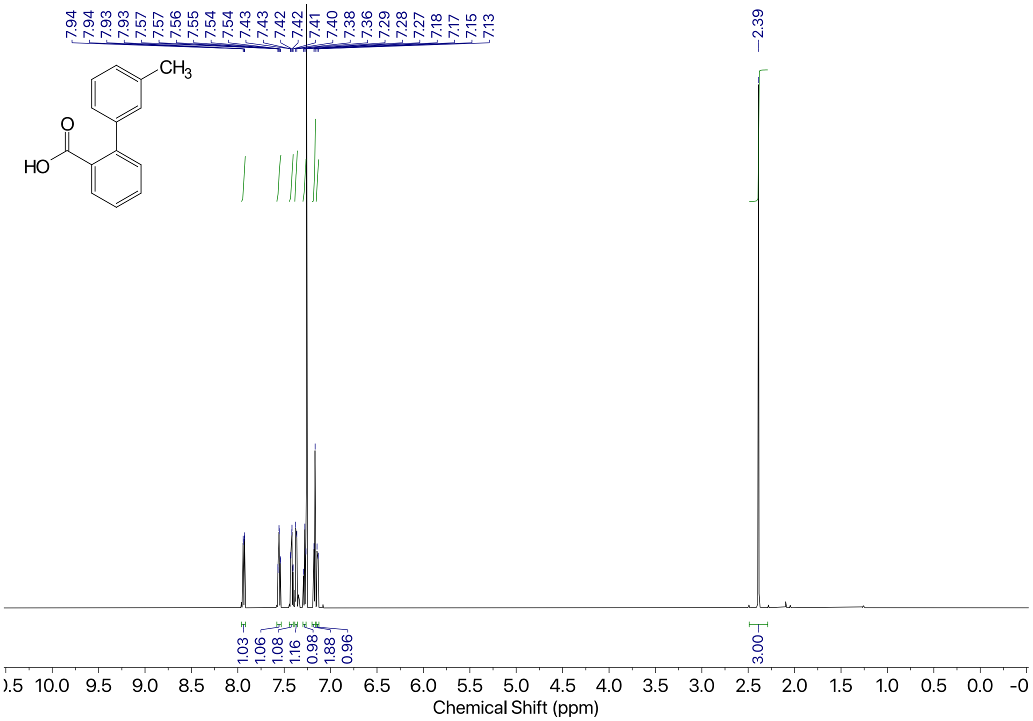

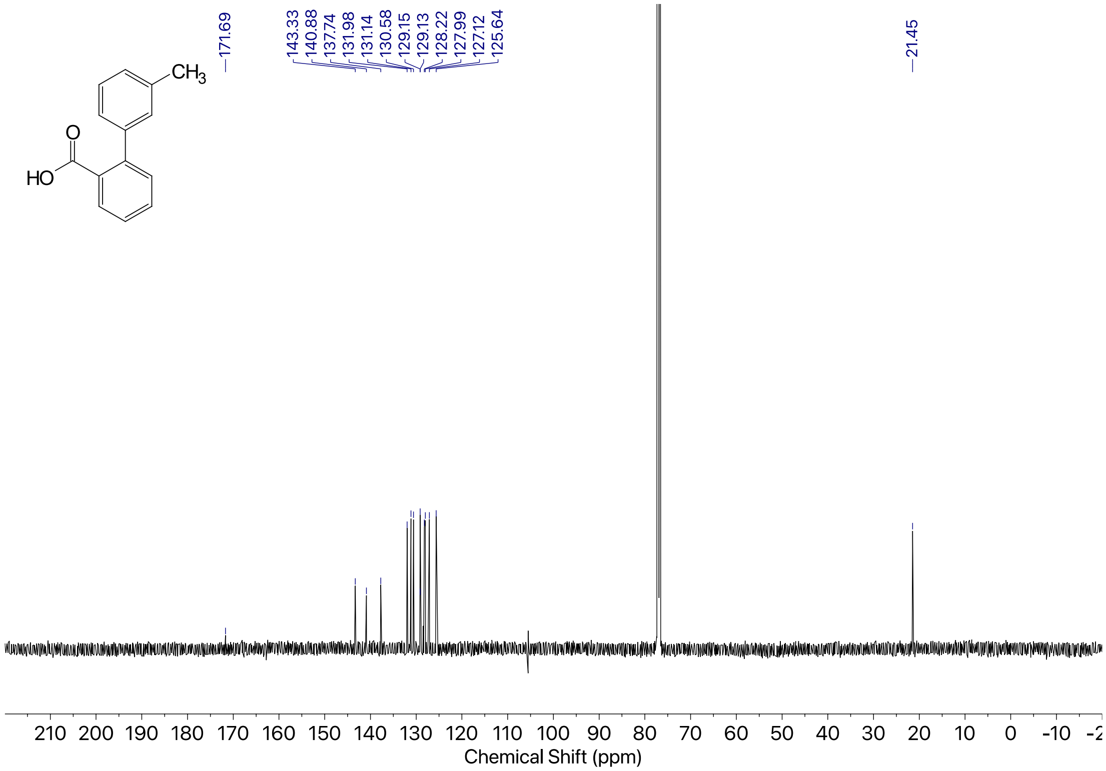

Compound 10c

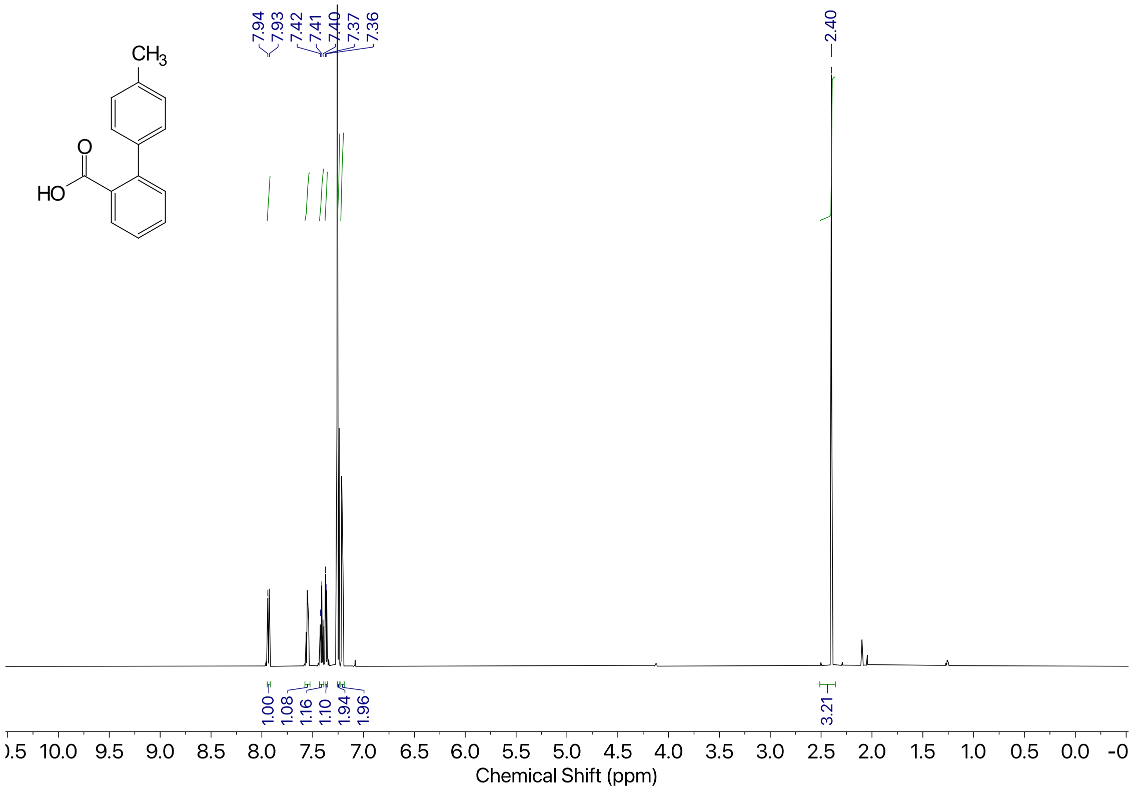

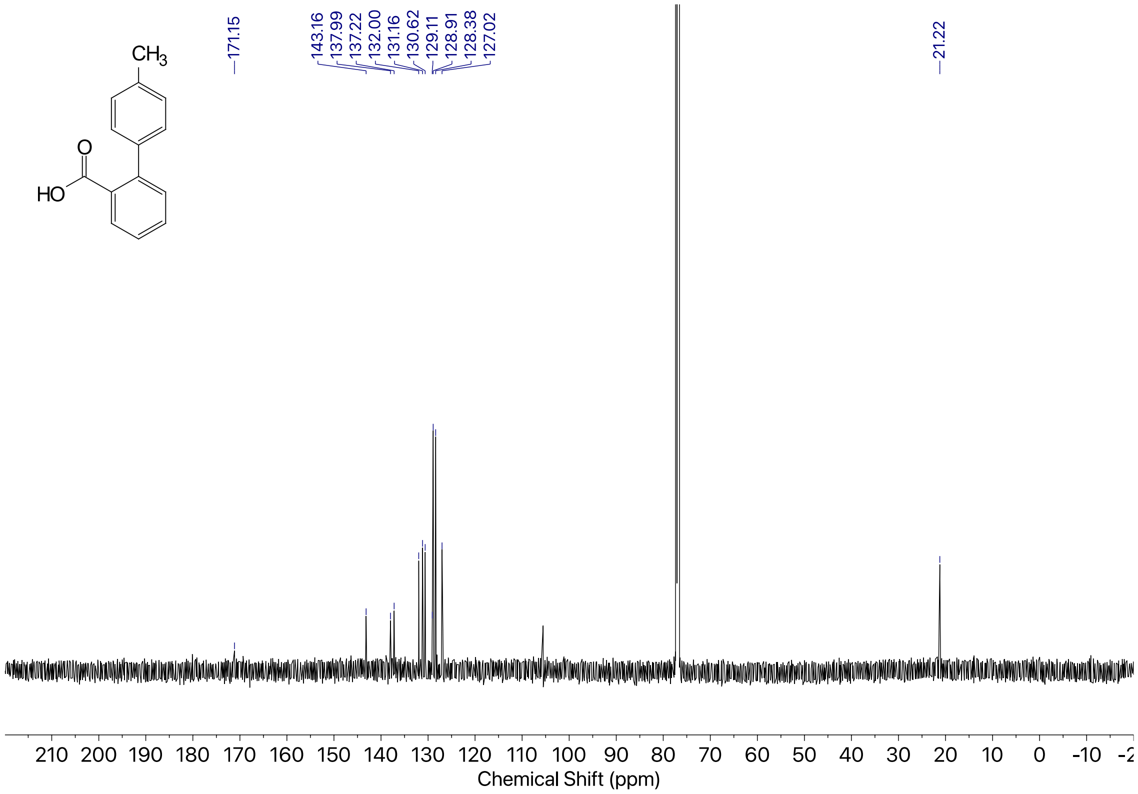

Compound 10d

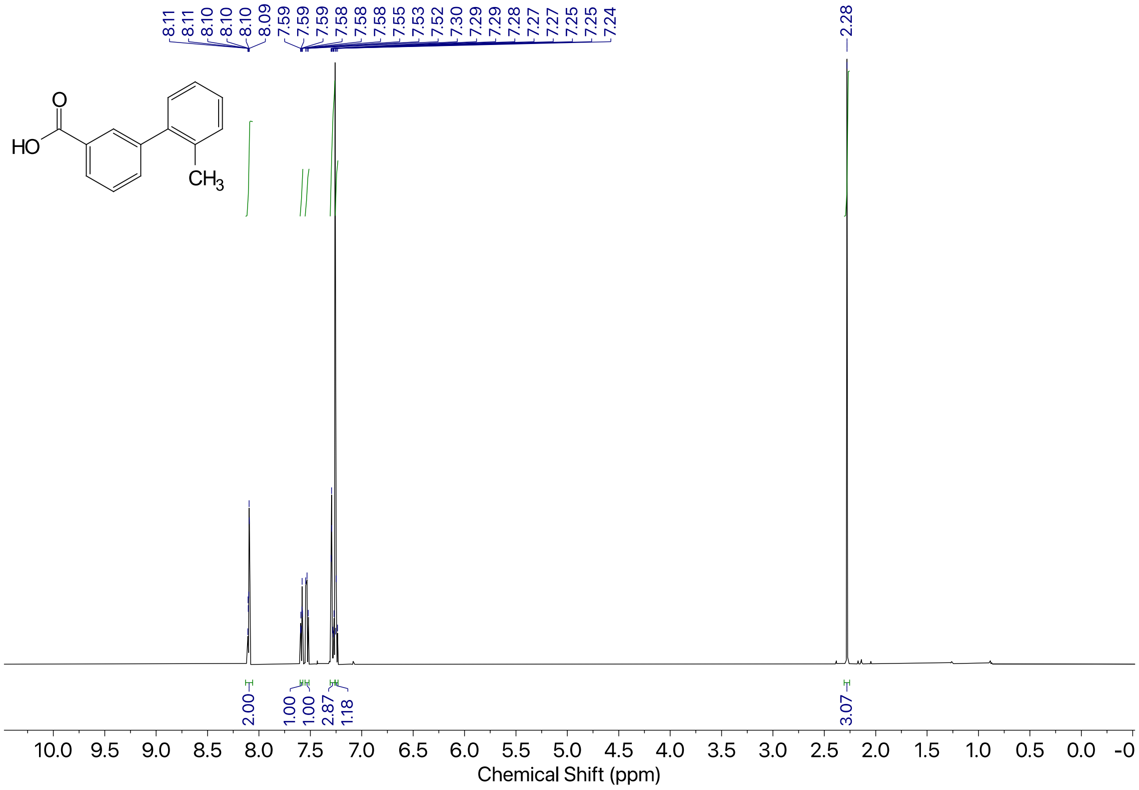

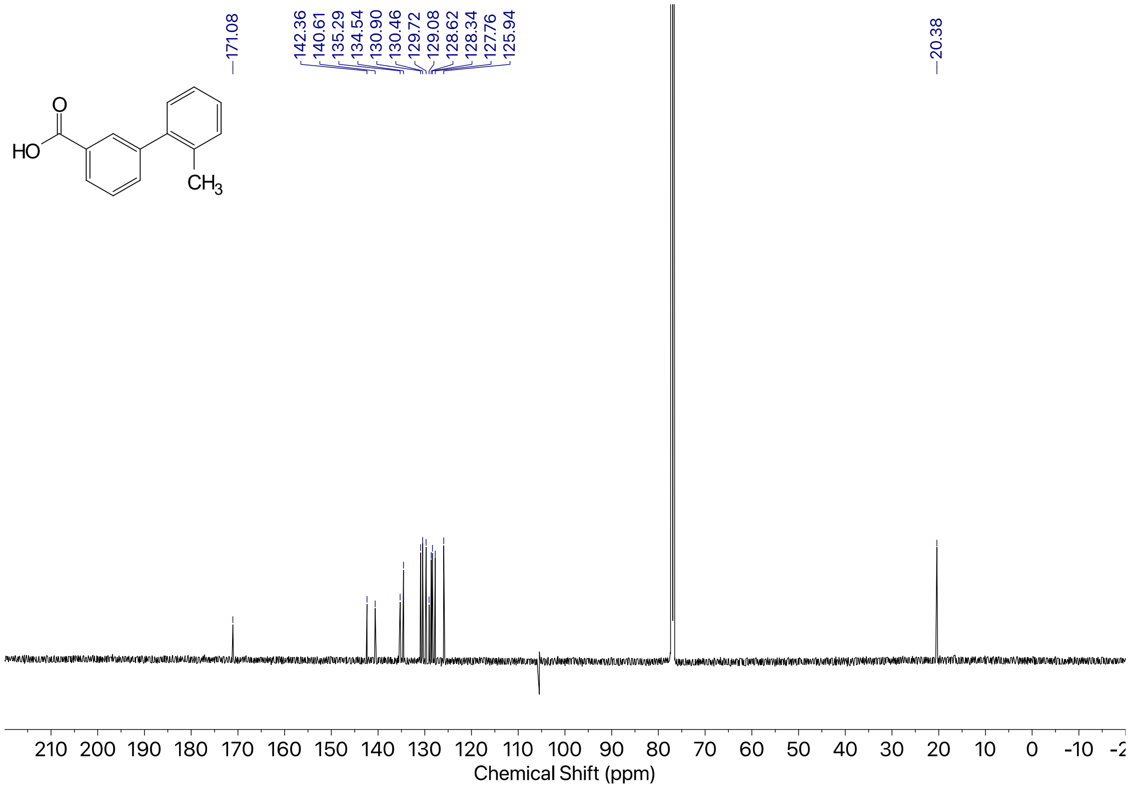

Compound 10e

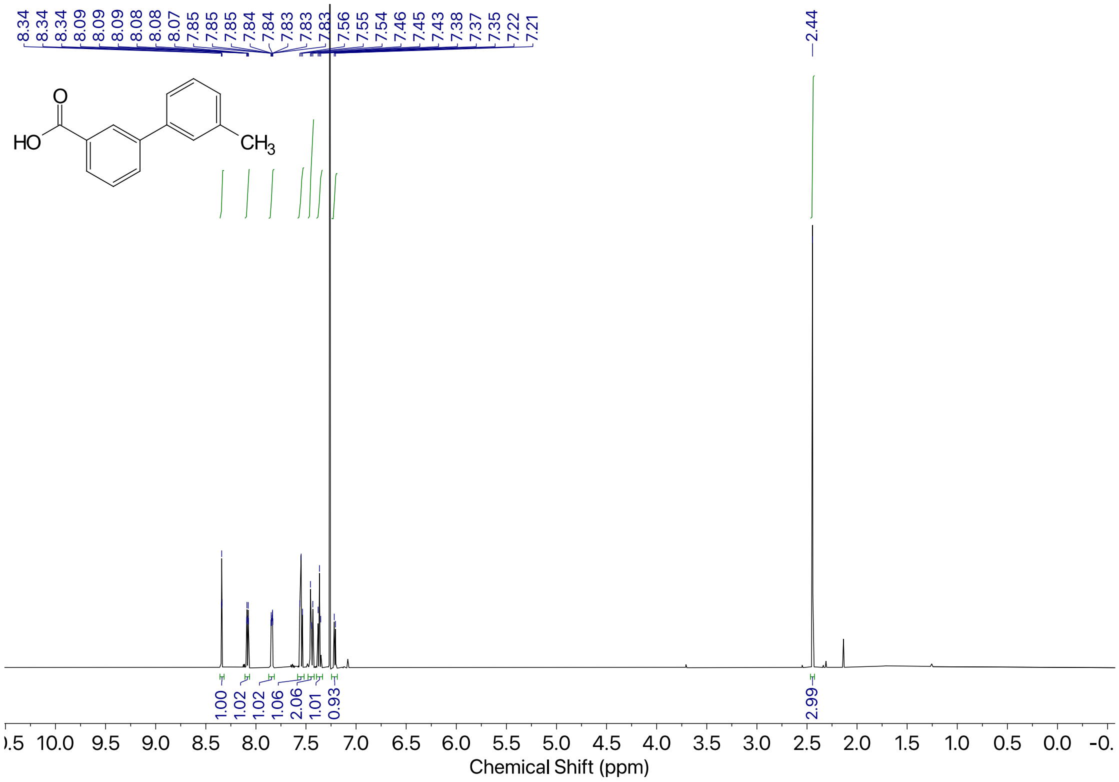

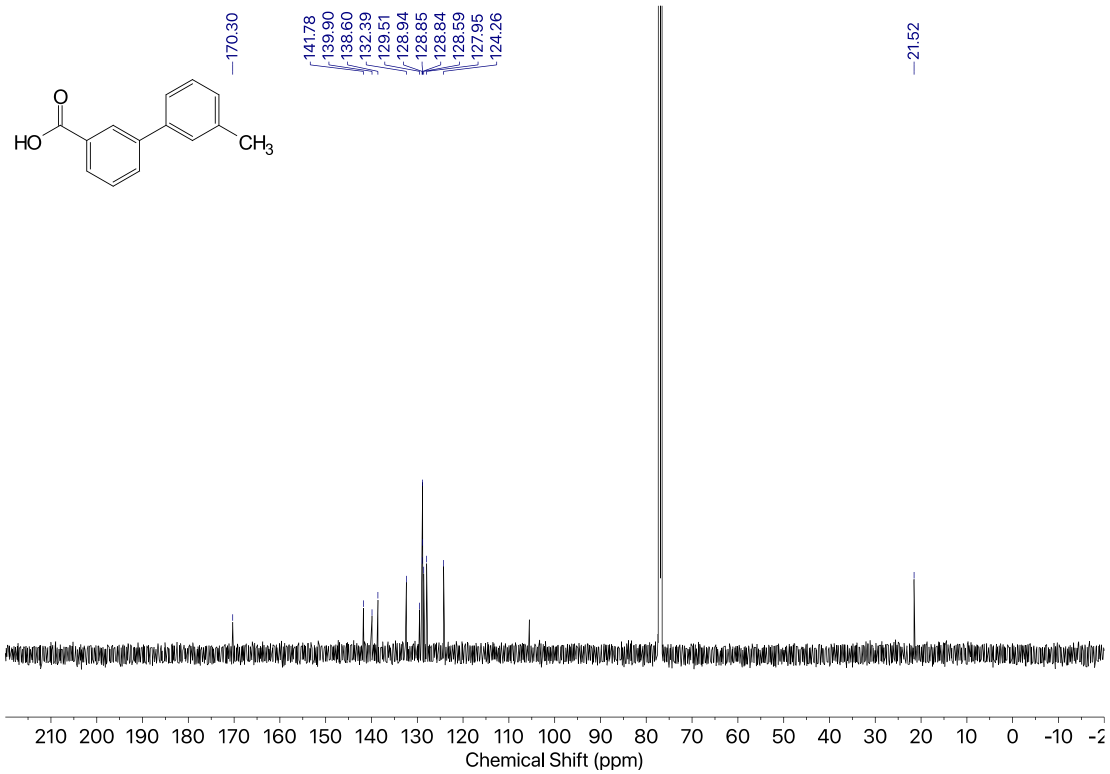

Compound 10f

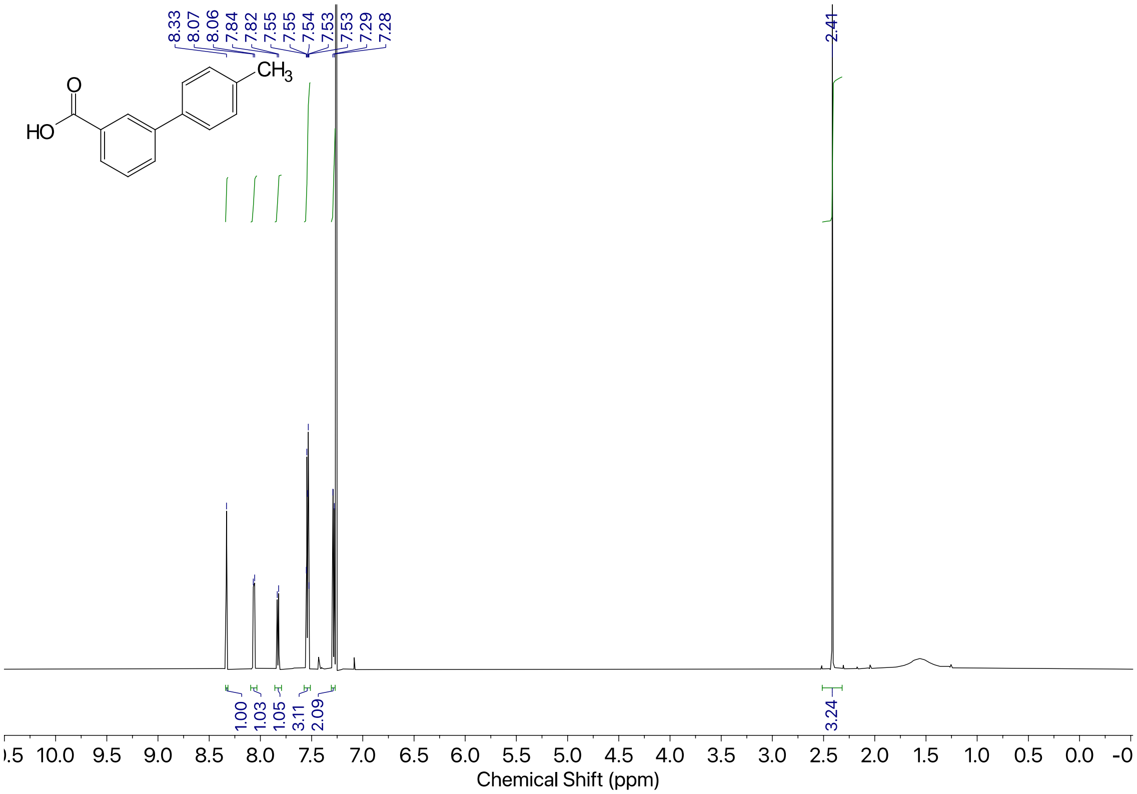

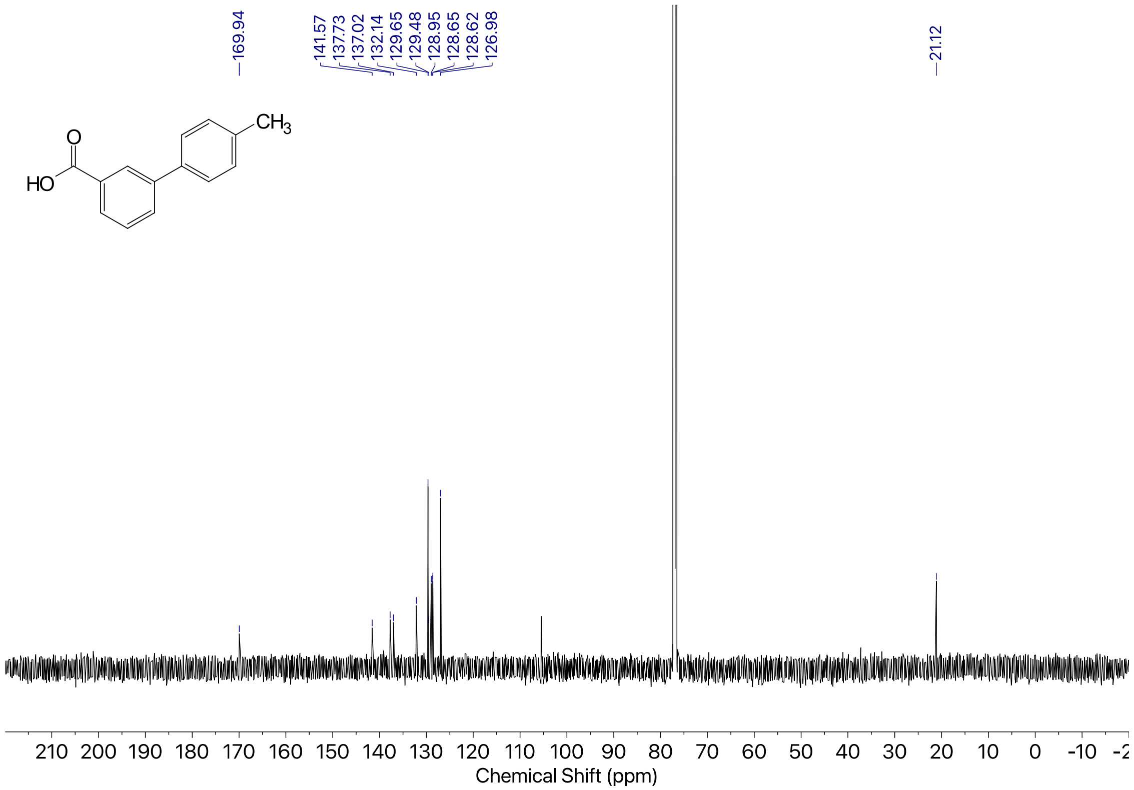

Compound 10g

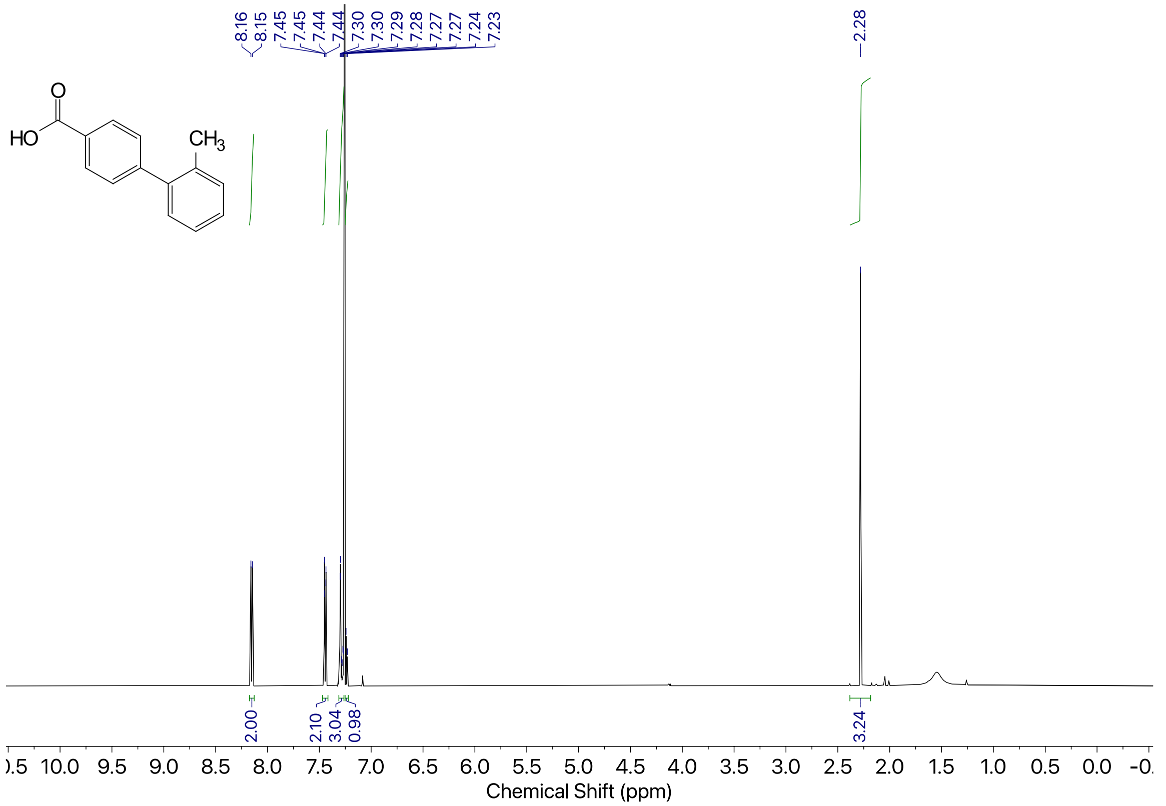

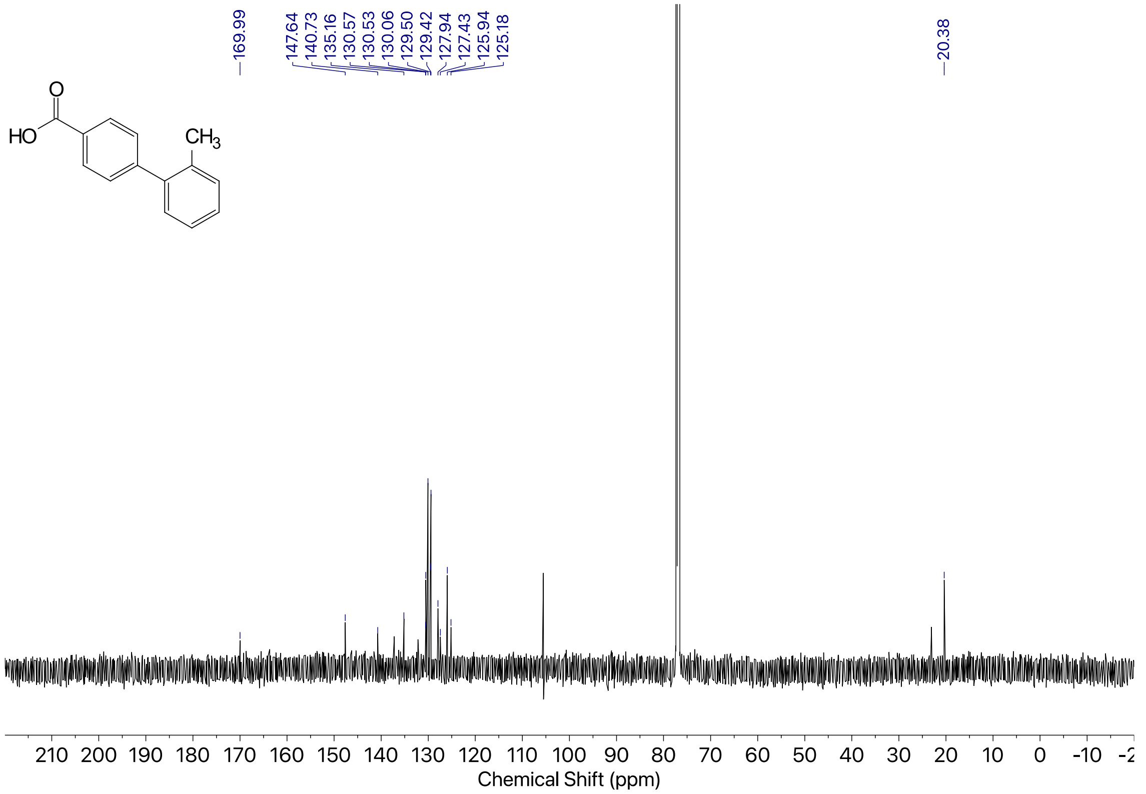

Compound 10h

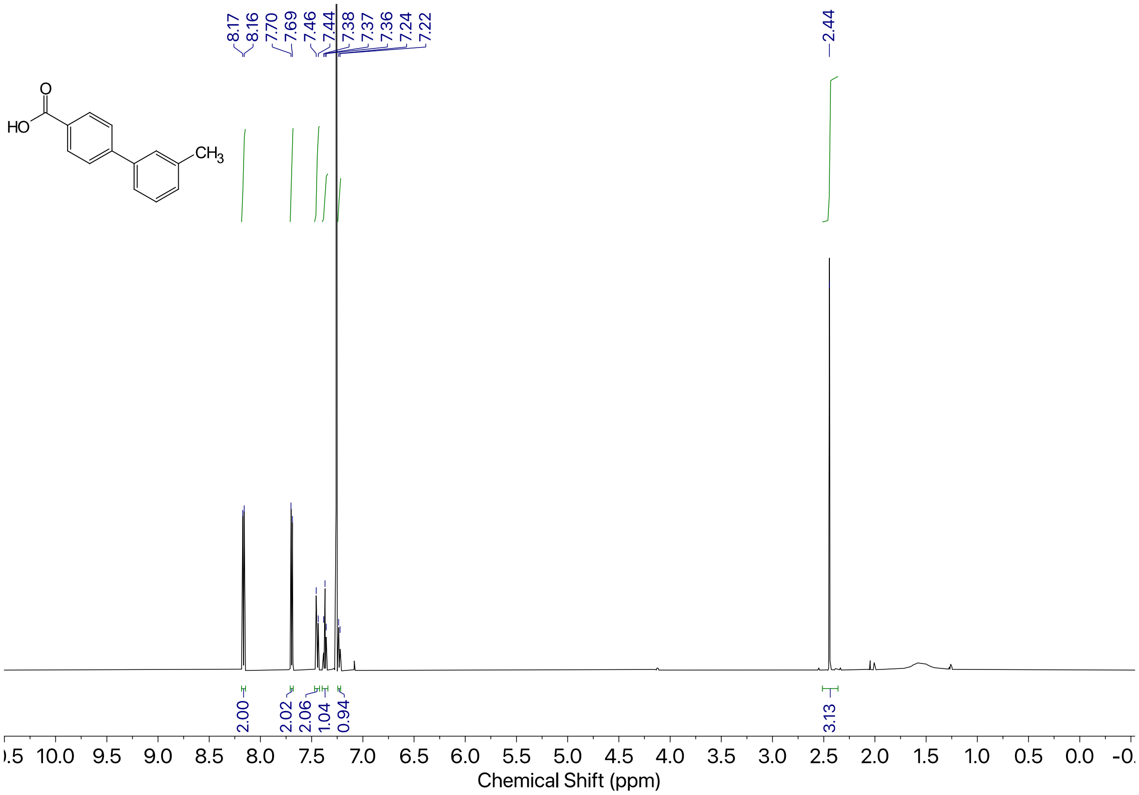

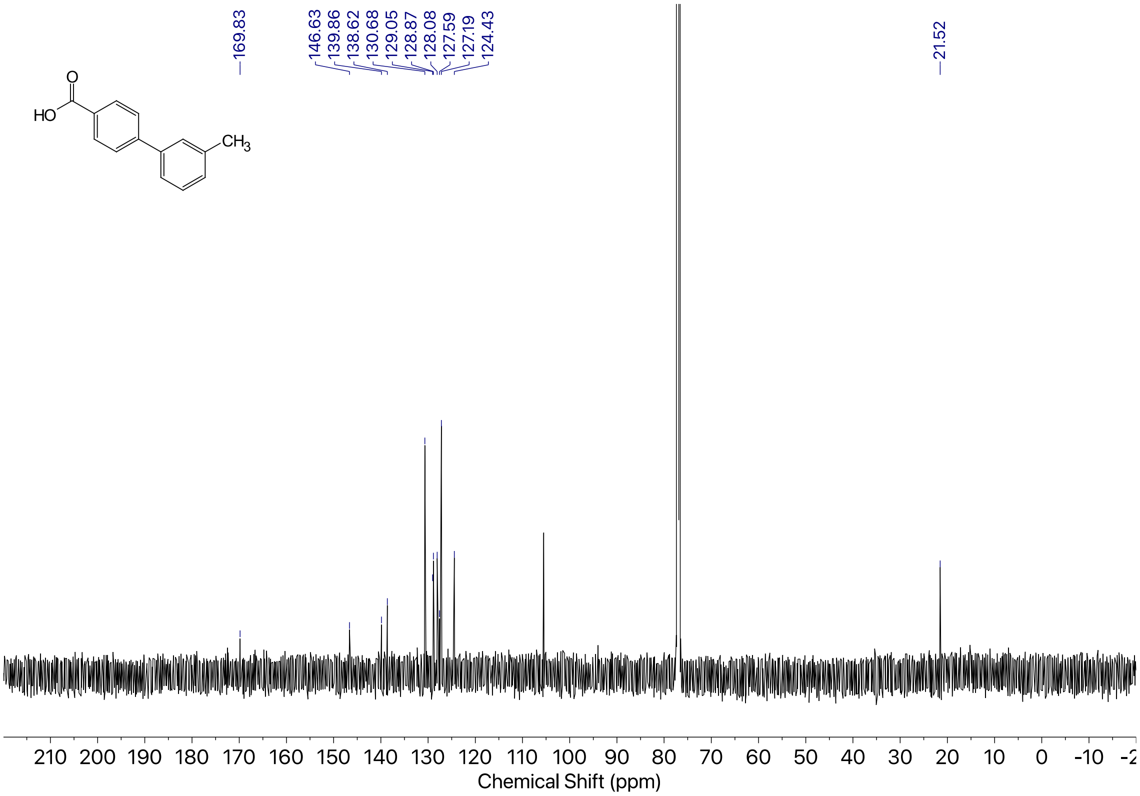

Compound 10i

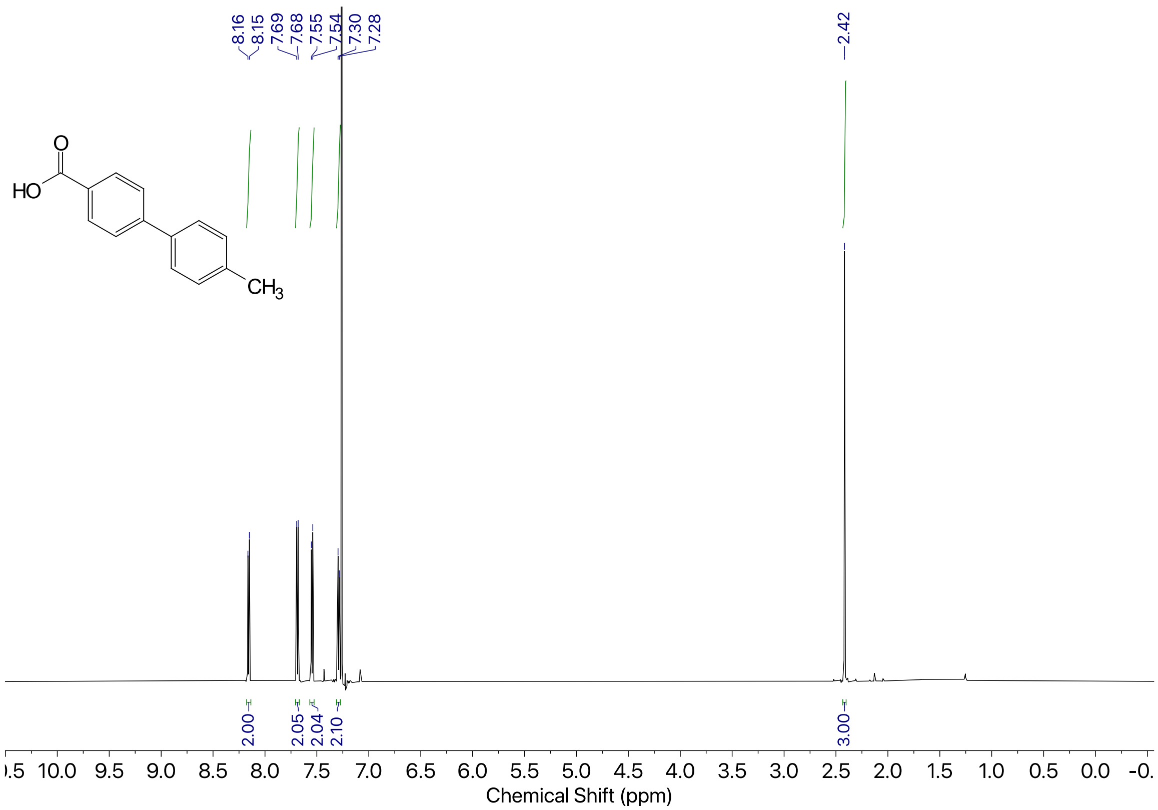

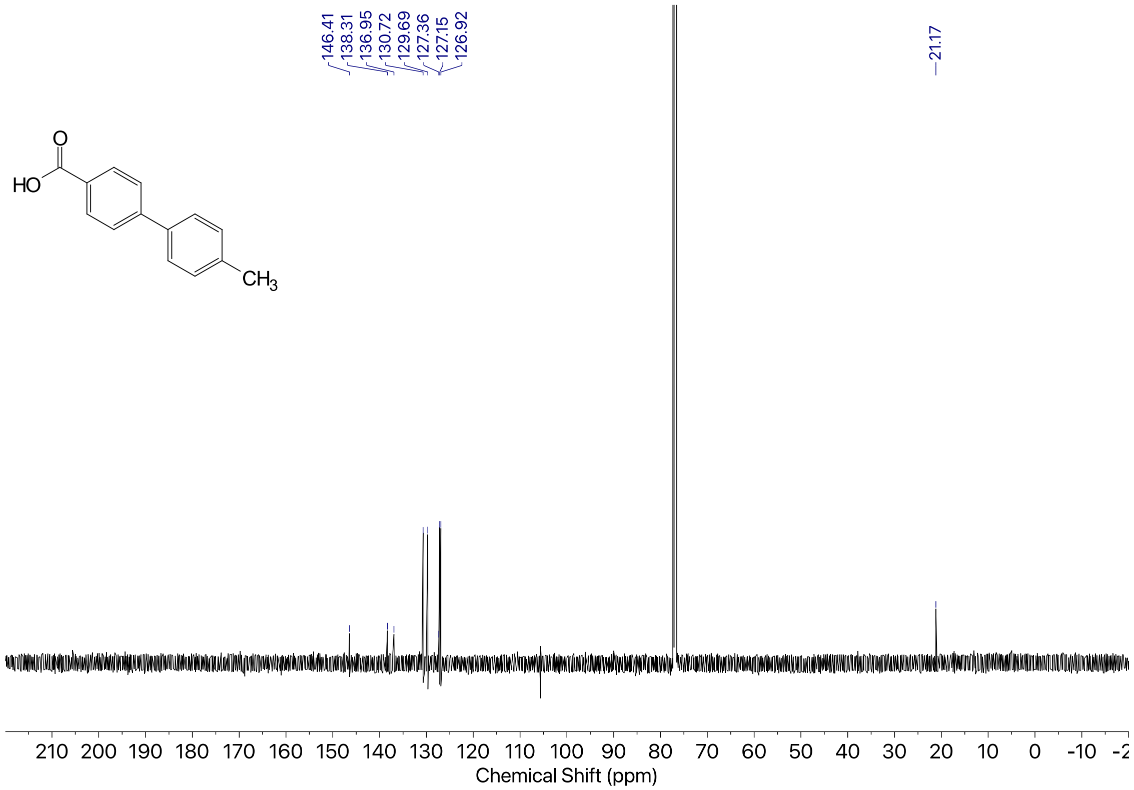

Compound 11a

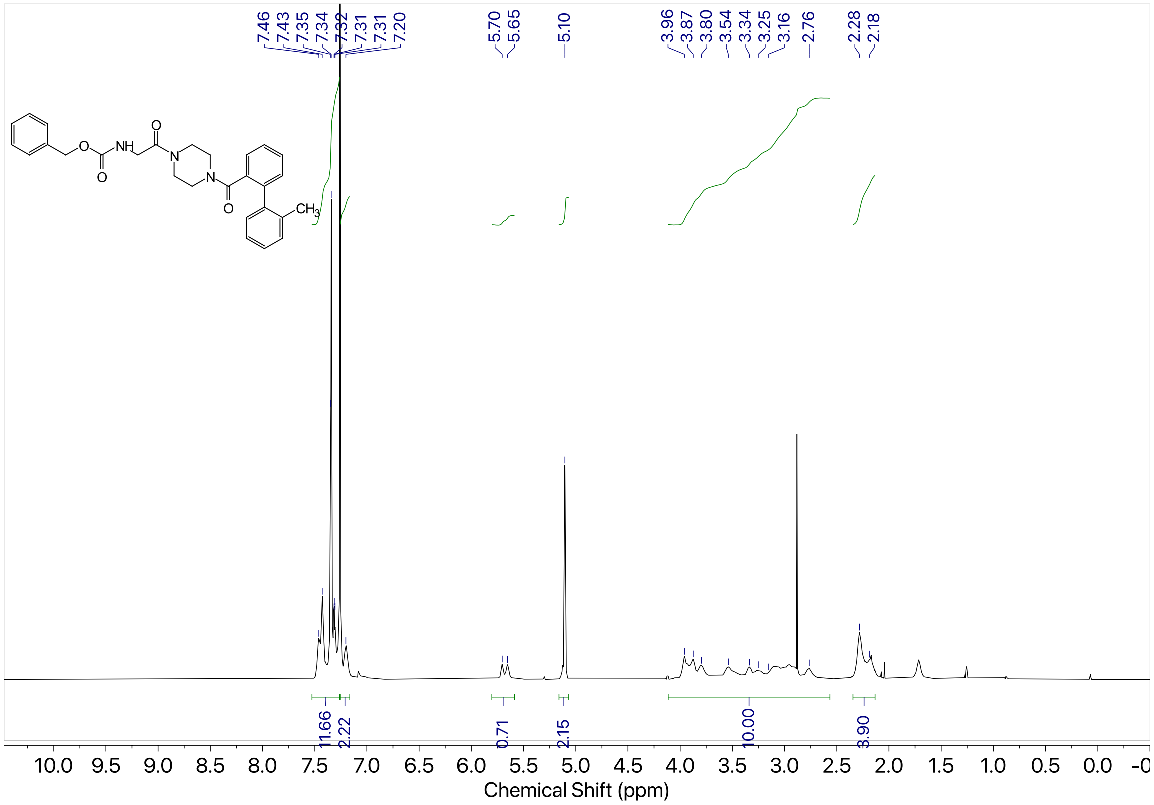

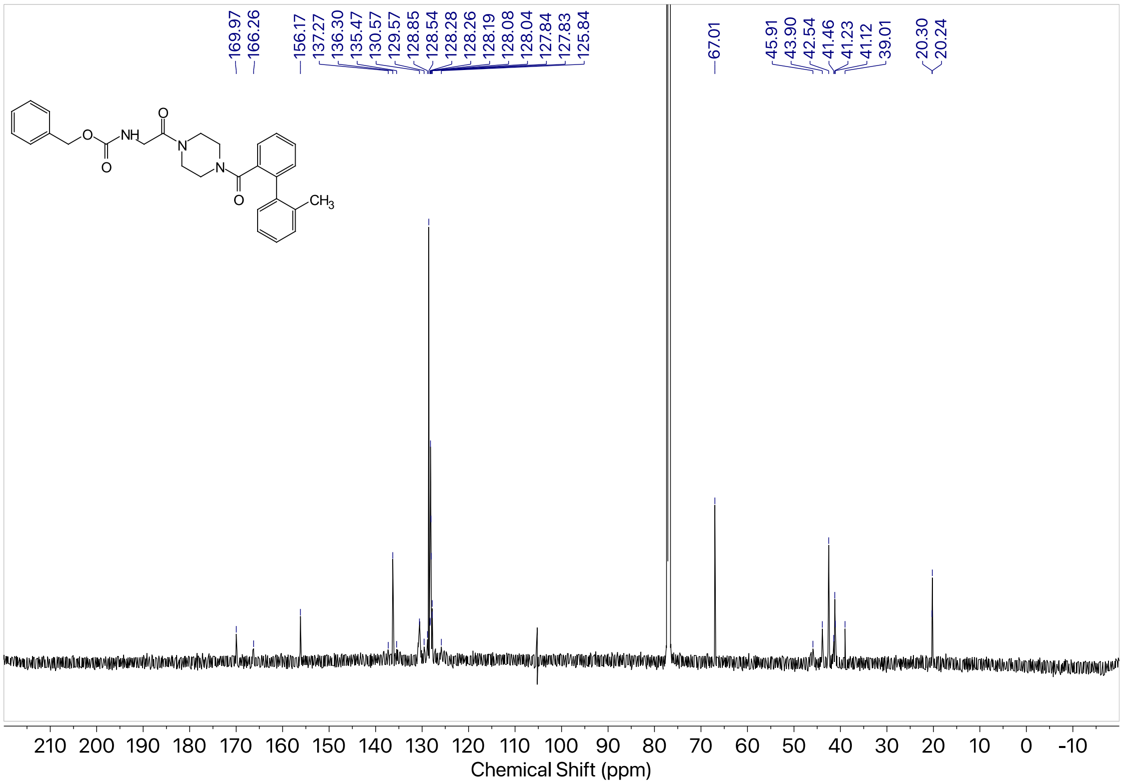

Compound 11b

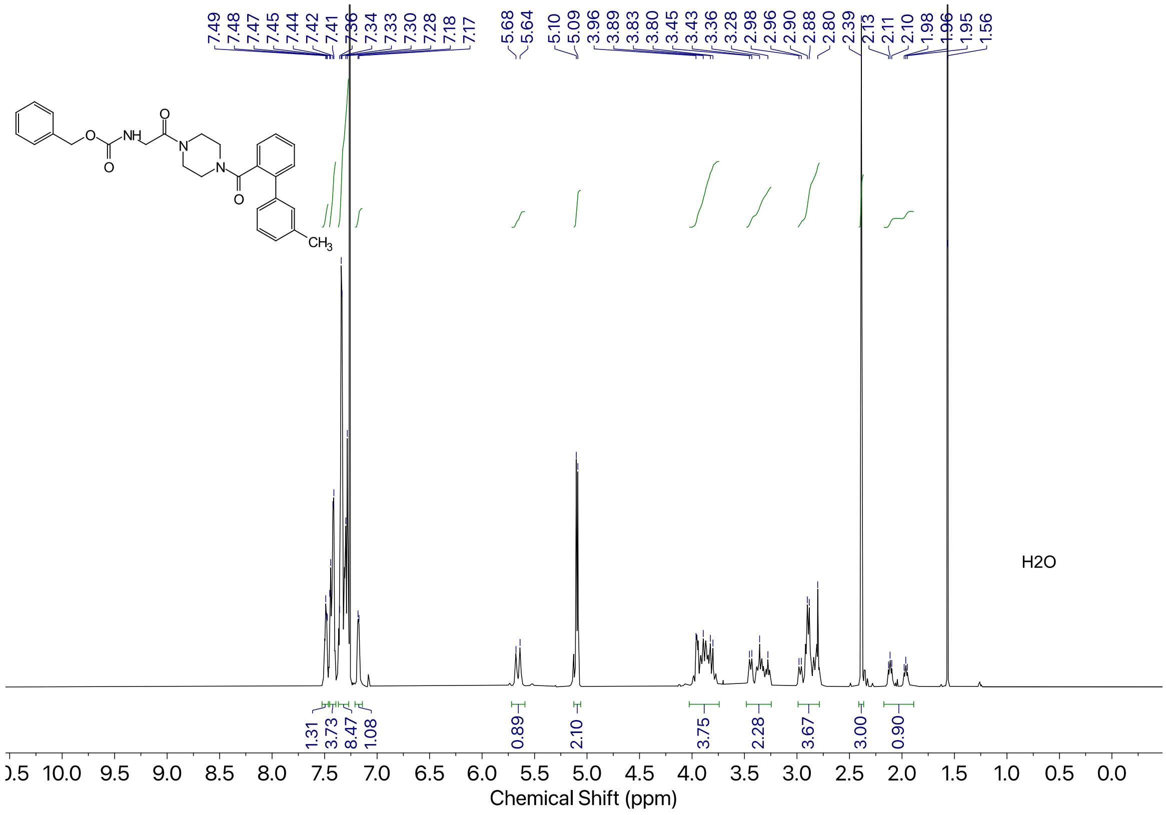

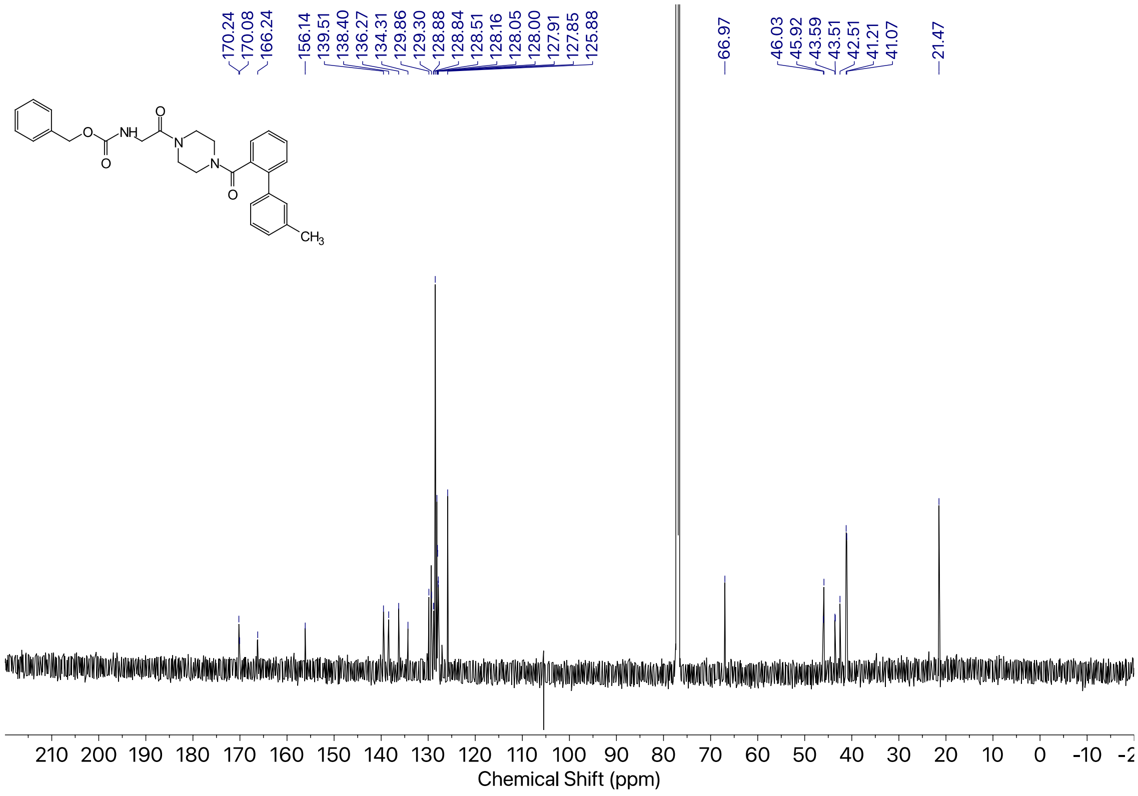

Compound 11c

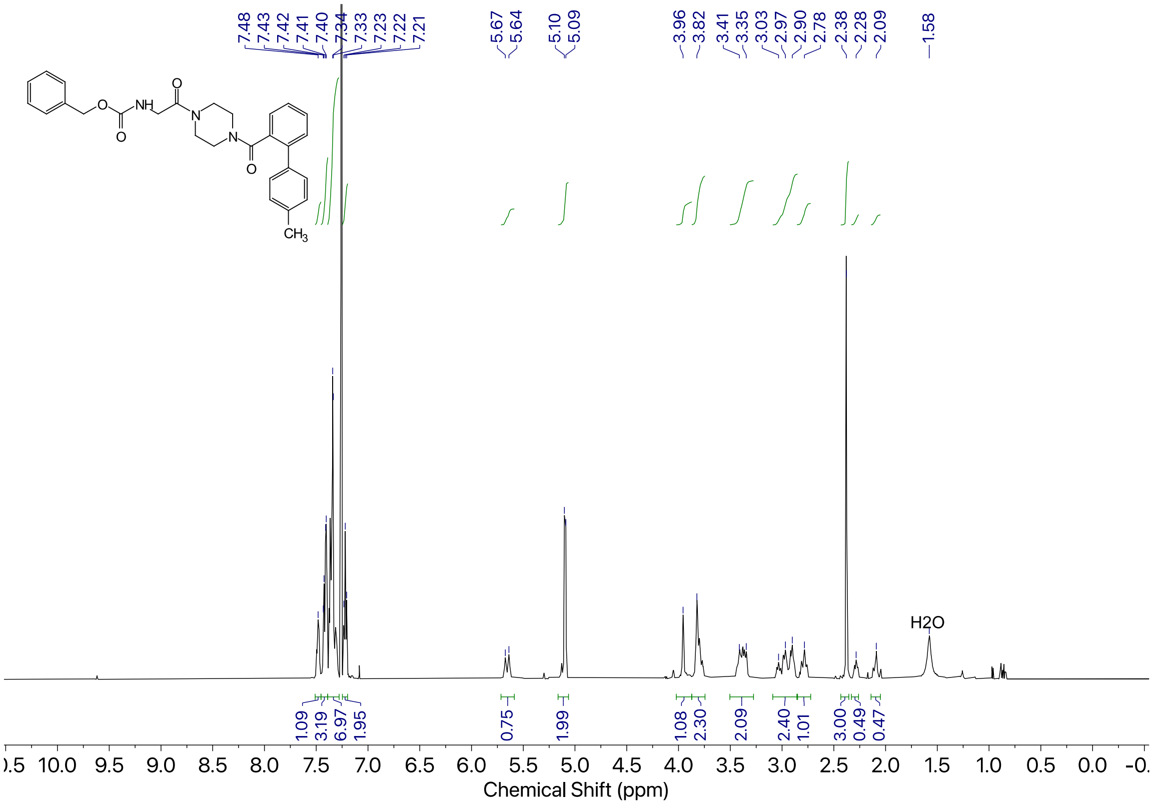

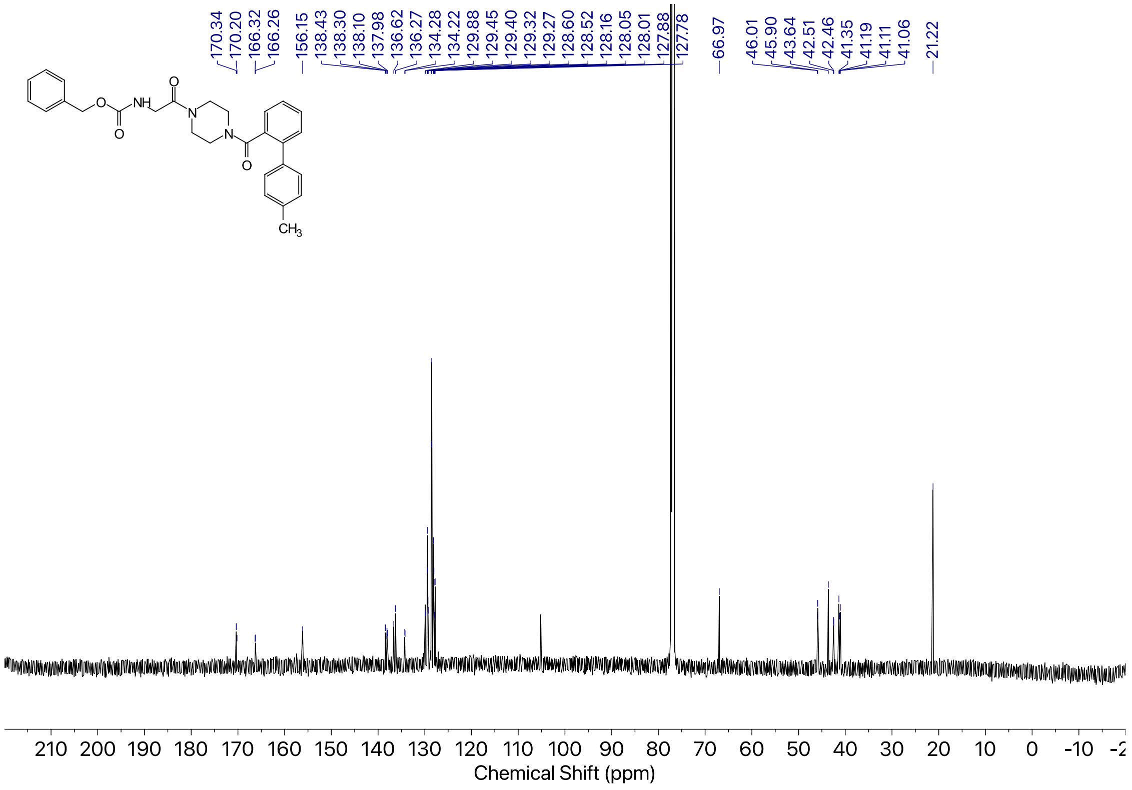

Compound 11d

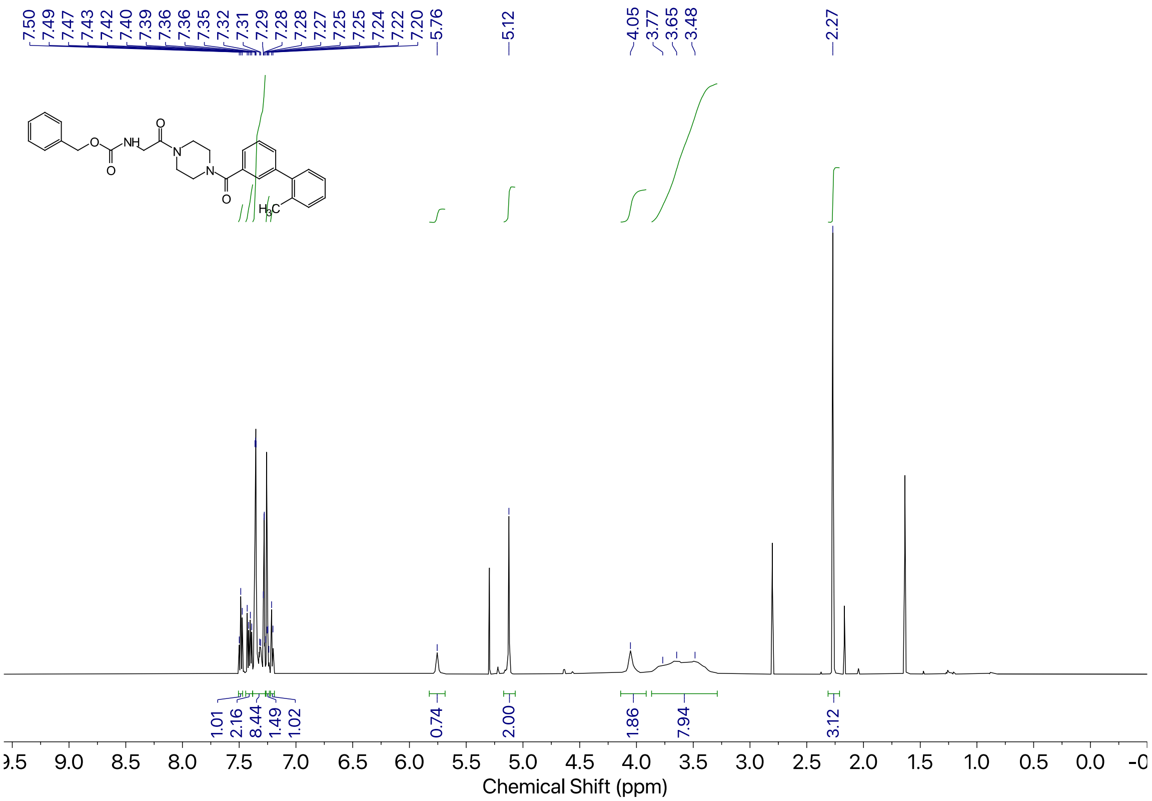

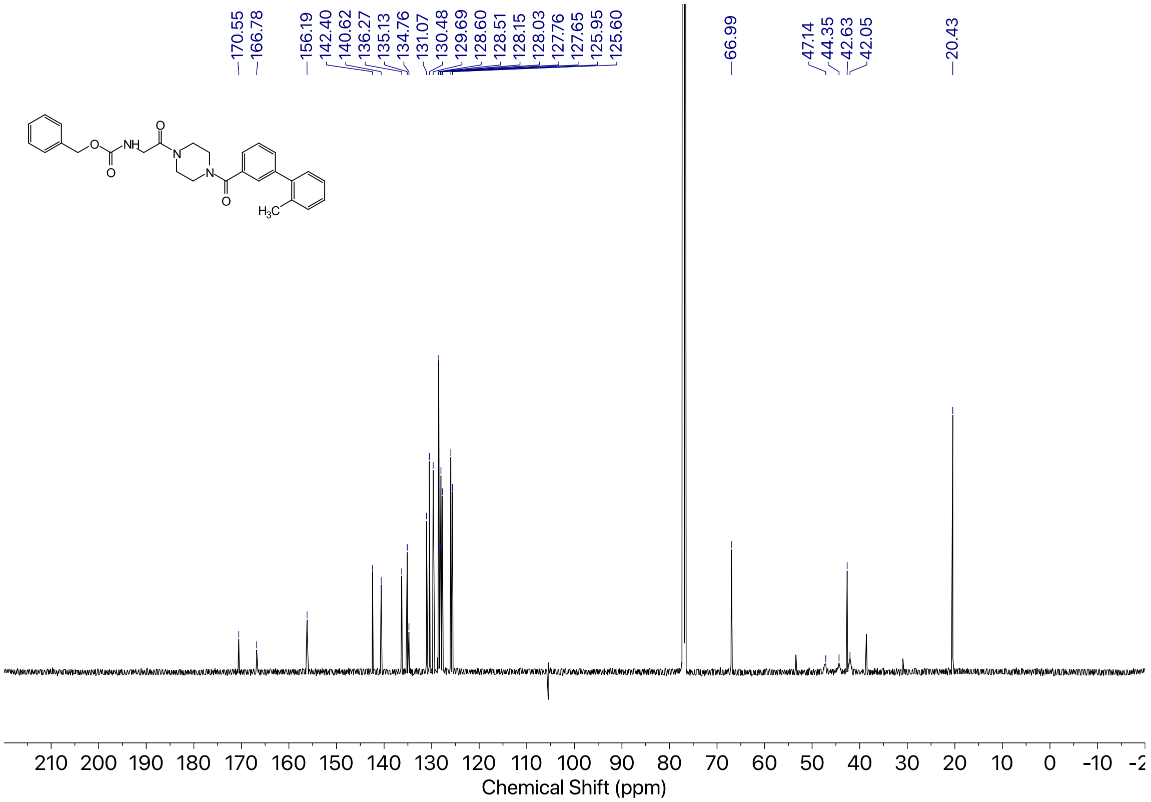

Compound 11e

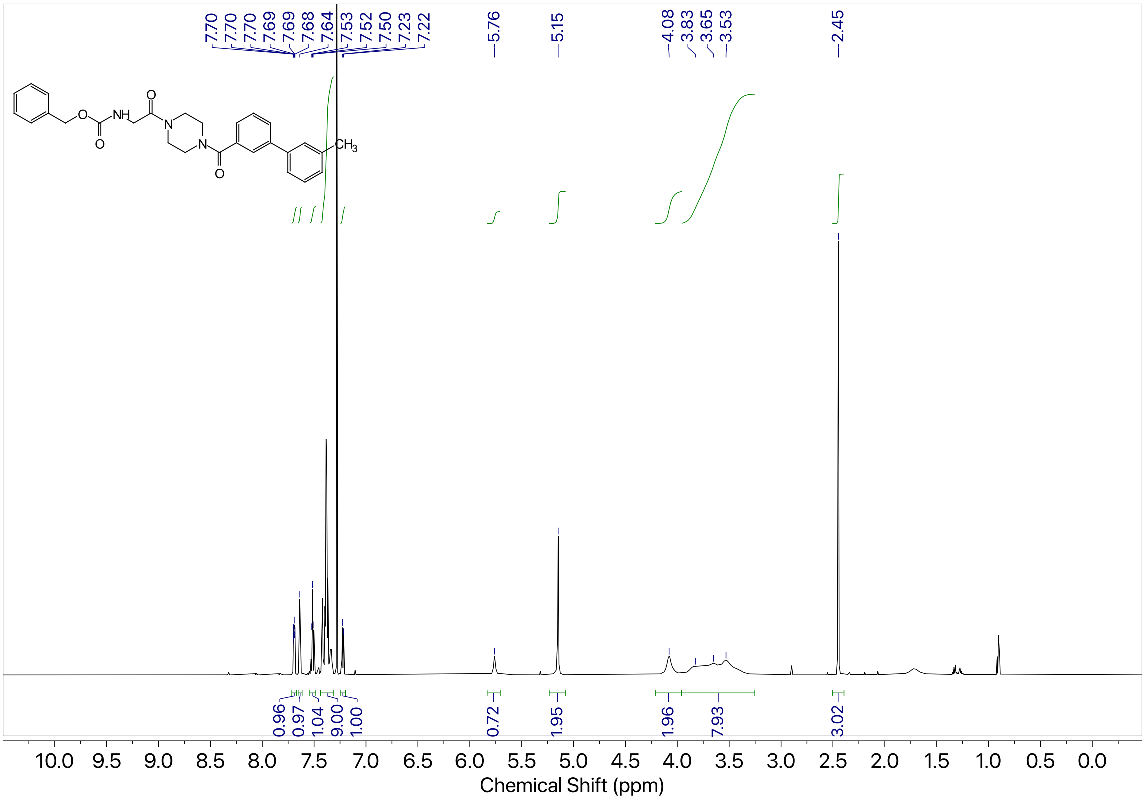

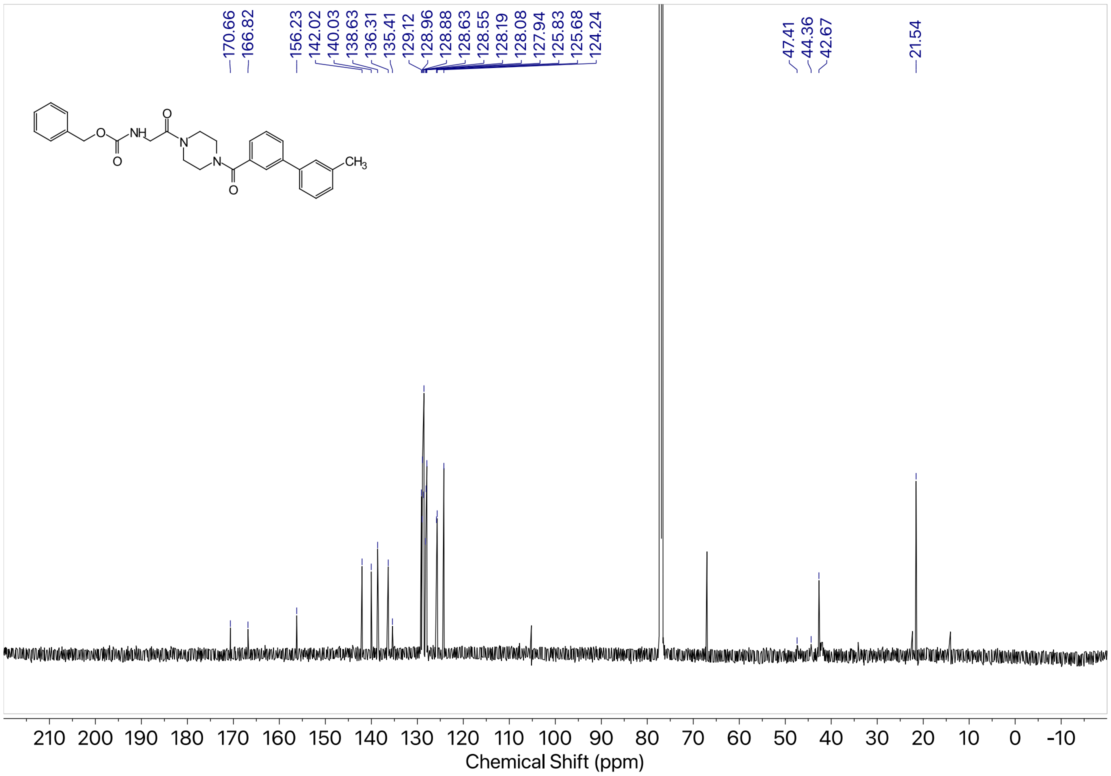

Compound 11f

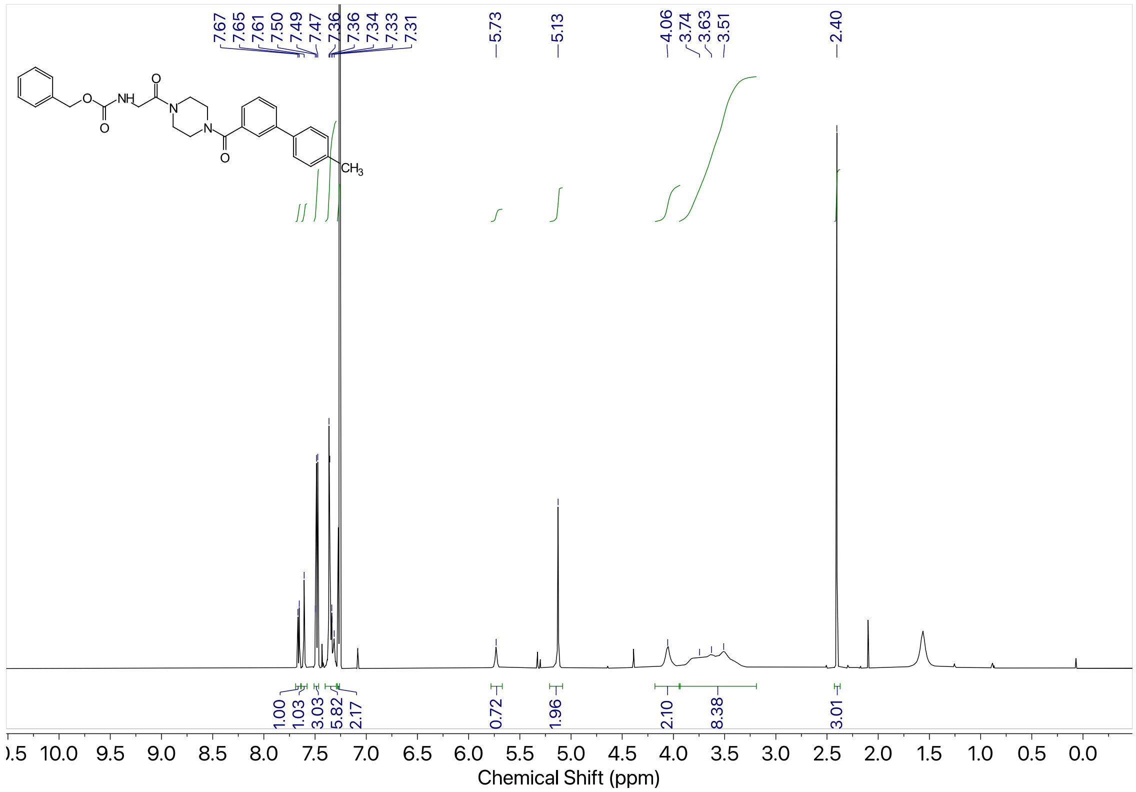

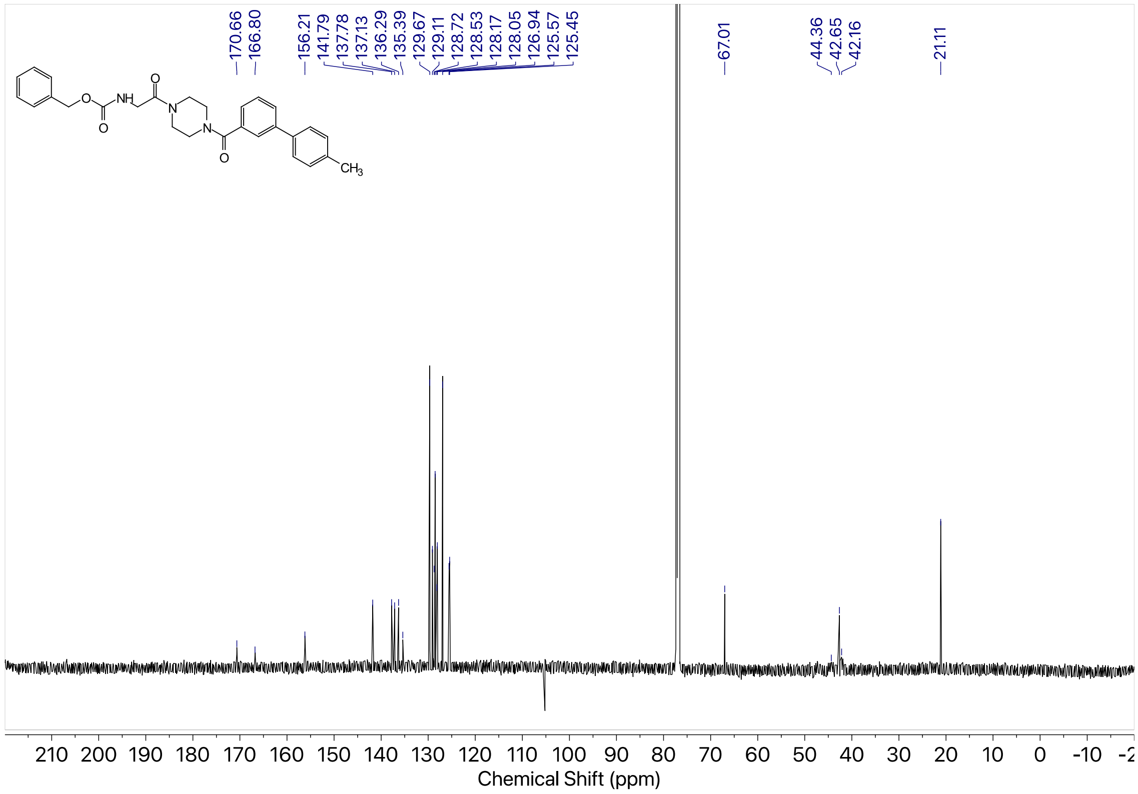

Compound 11g

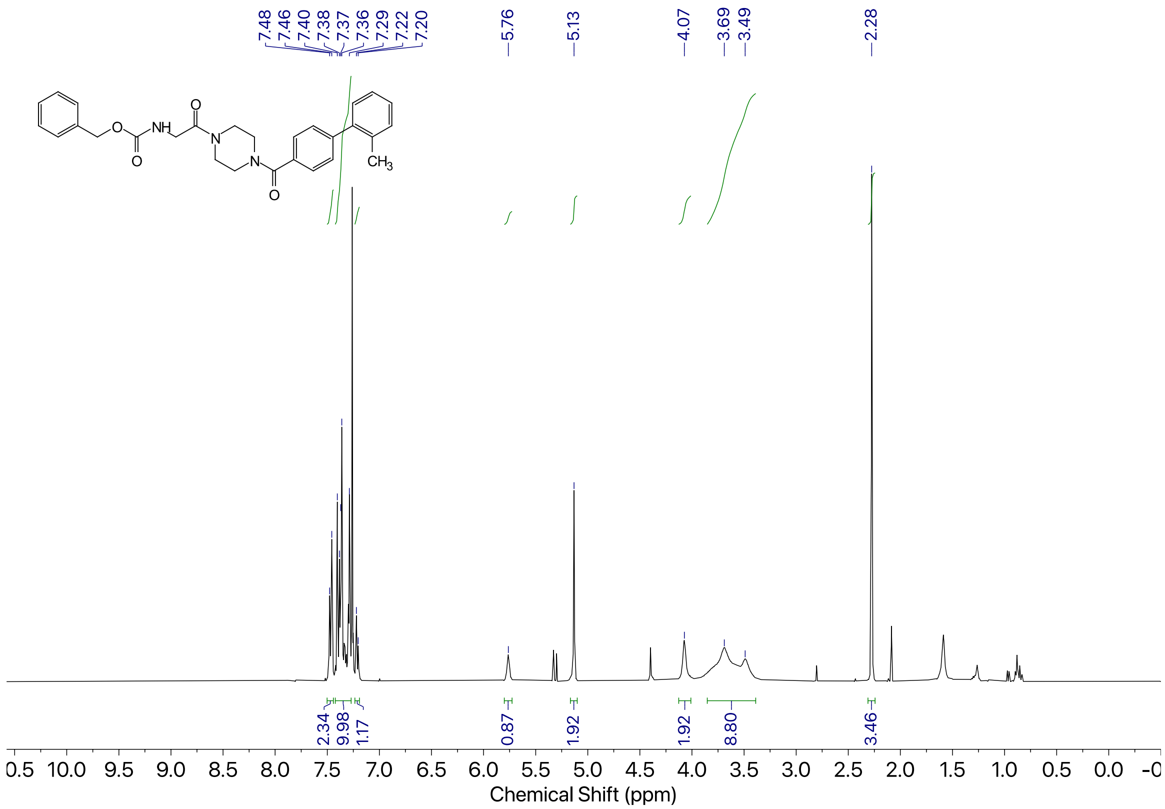

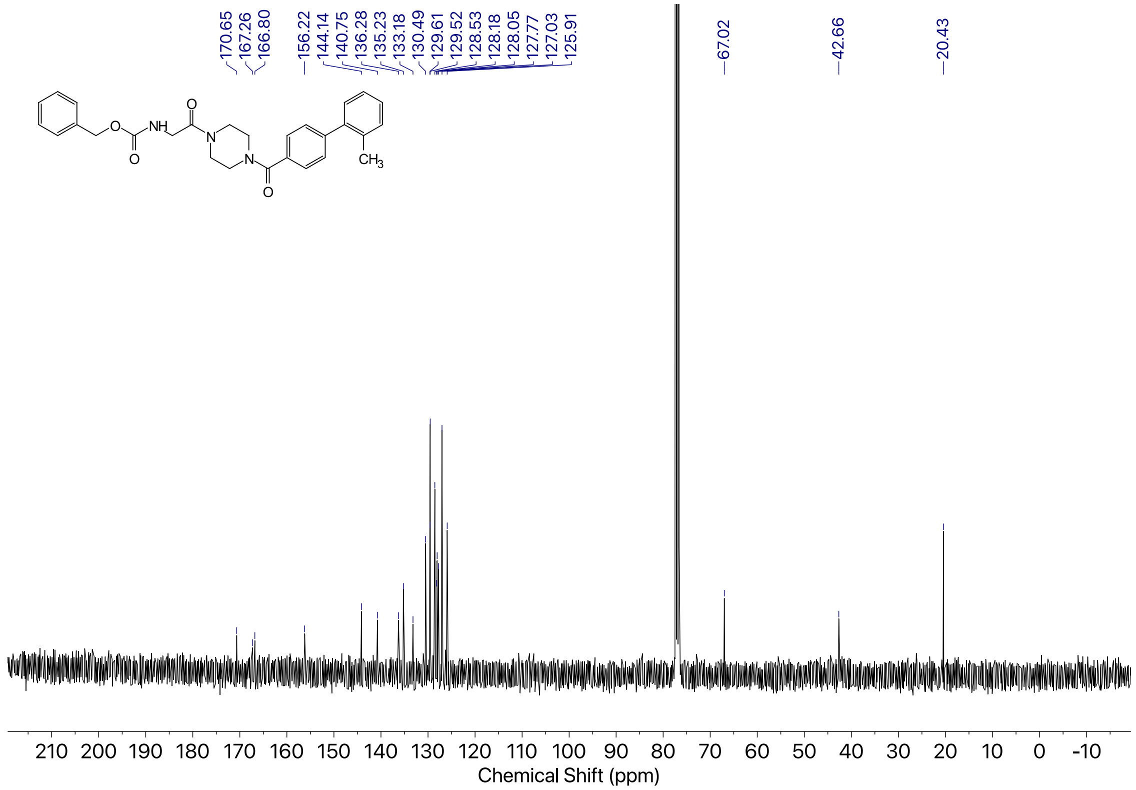

Compound 11h

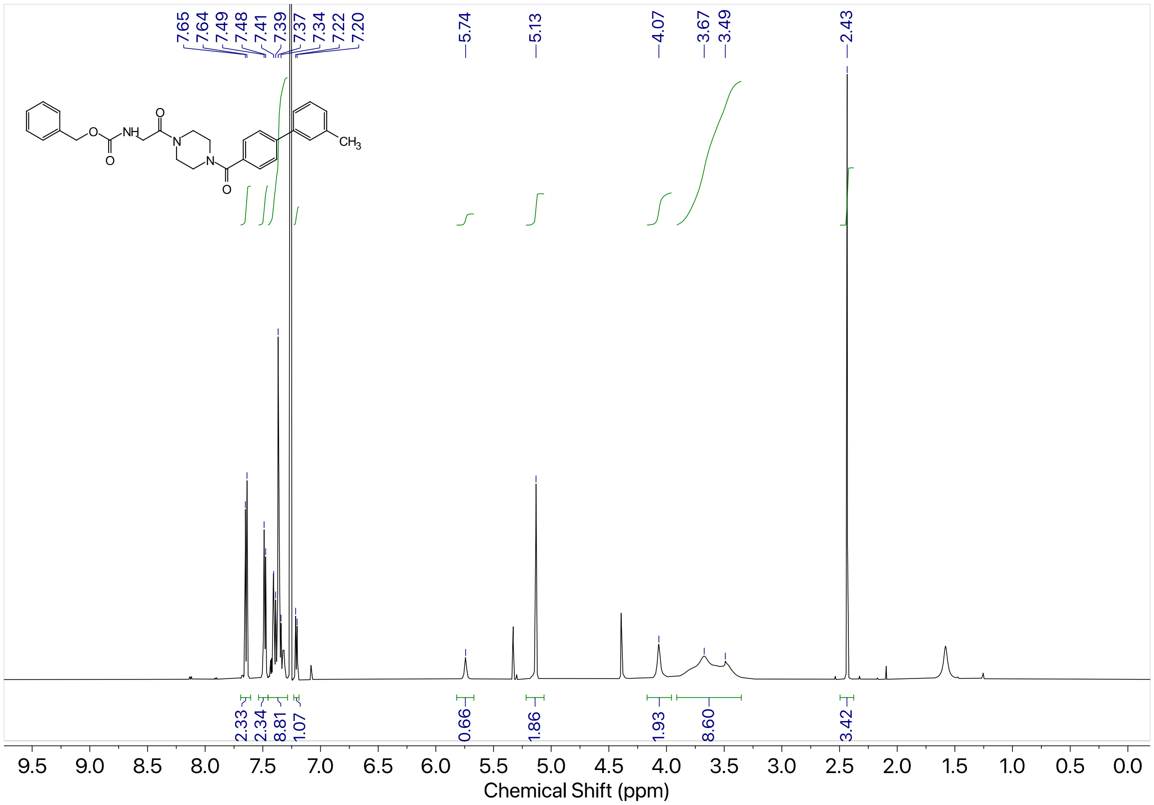

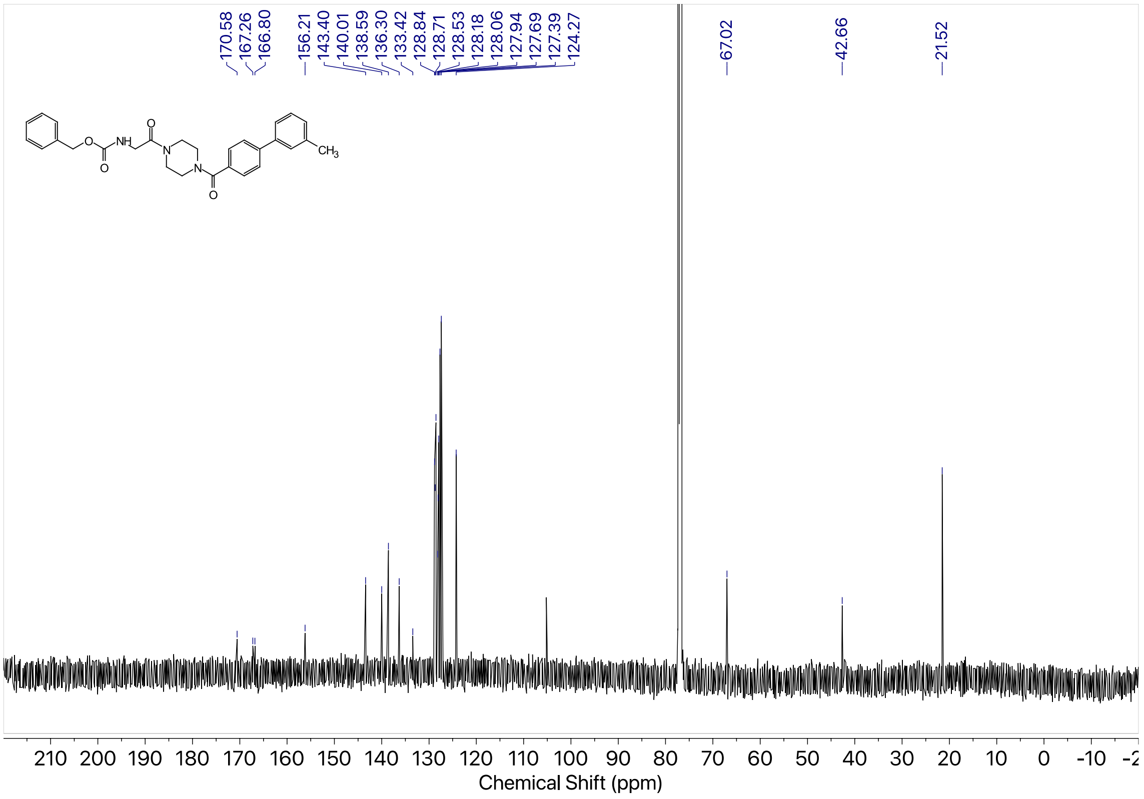

Compound 11i

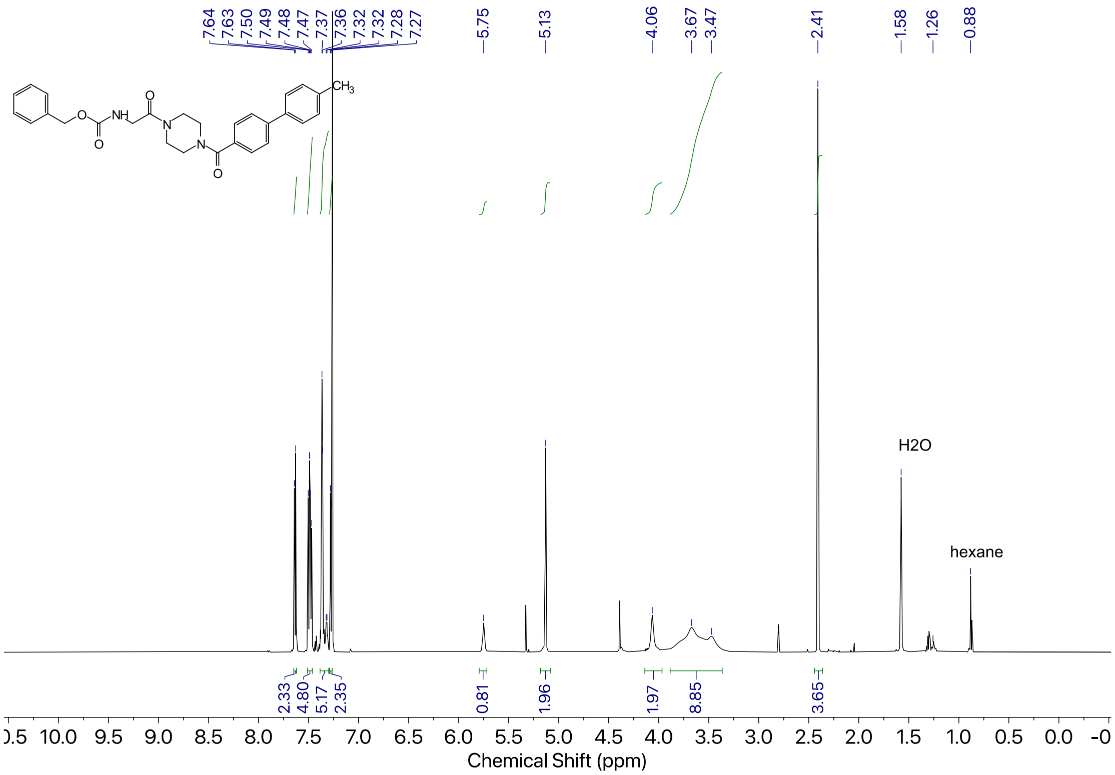

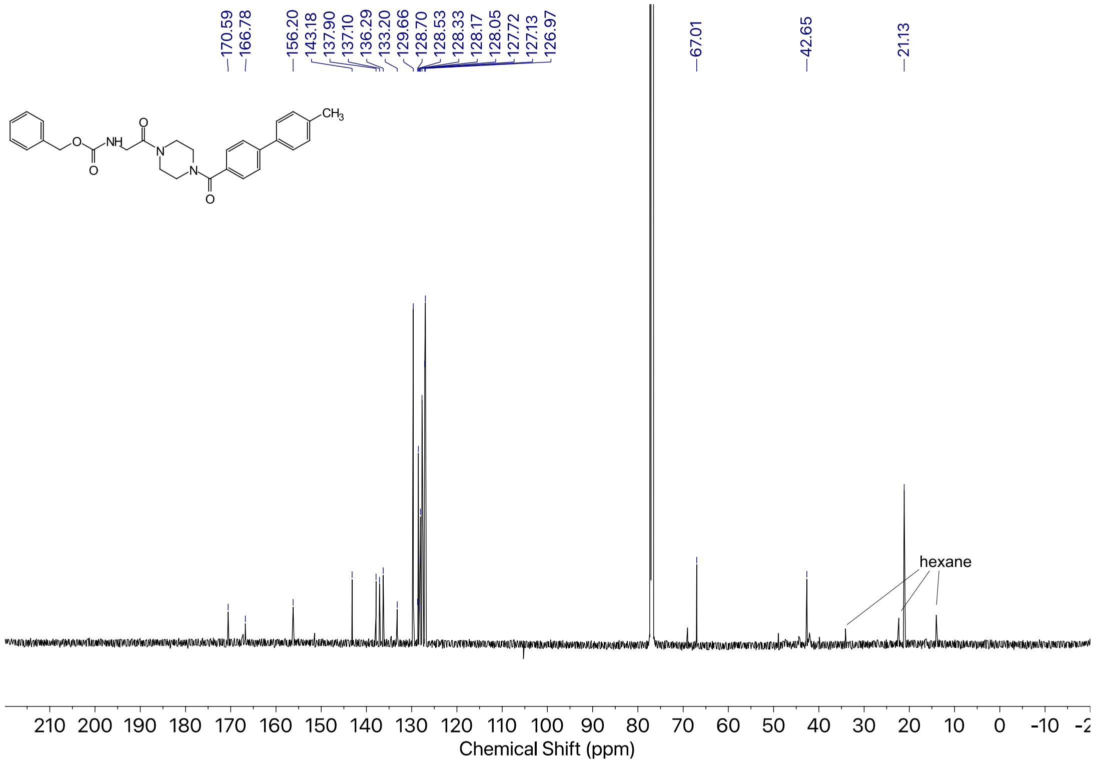

Compound 14

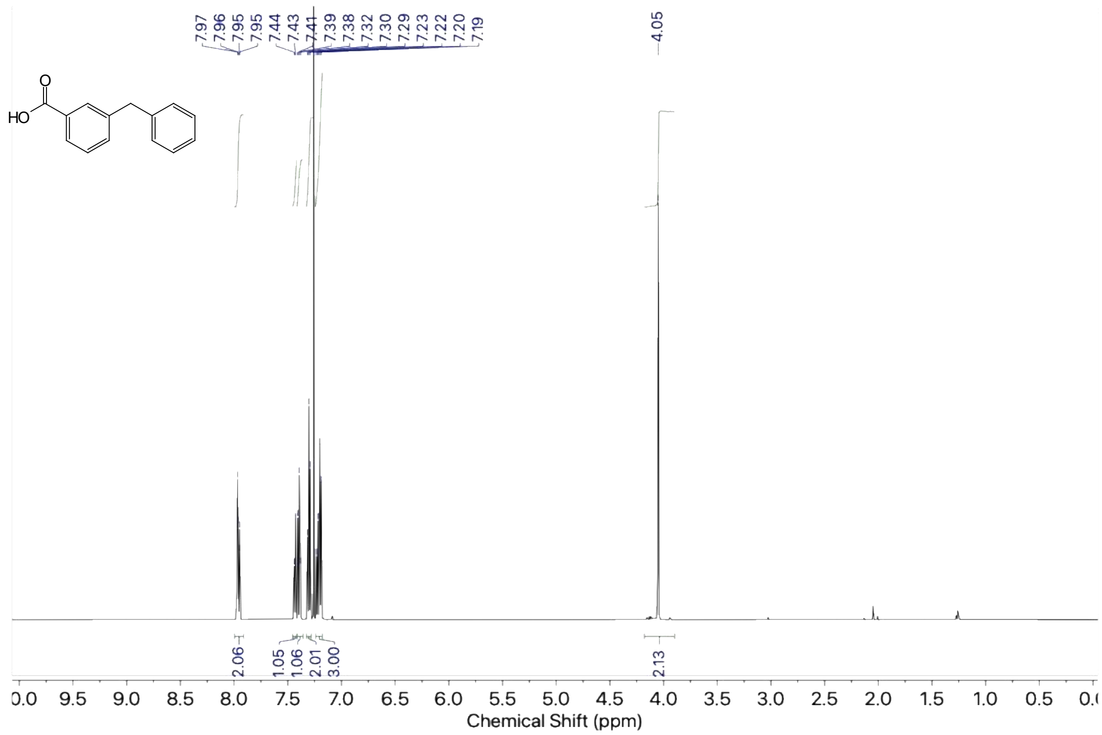
HO
O
7.97
7.96
7.95
7.95
7.44
7.43
7.41
7.39
7.38
7.32
7.30
7.29
7.23
7.22
7.20
7.19
4.05
2.06
1.05
1.06
2.01
3.00
2.13
Chemical Shift (ppm)

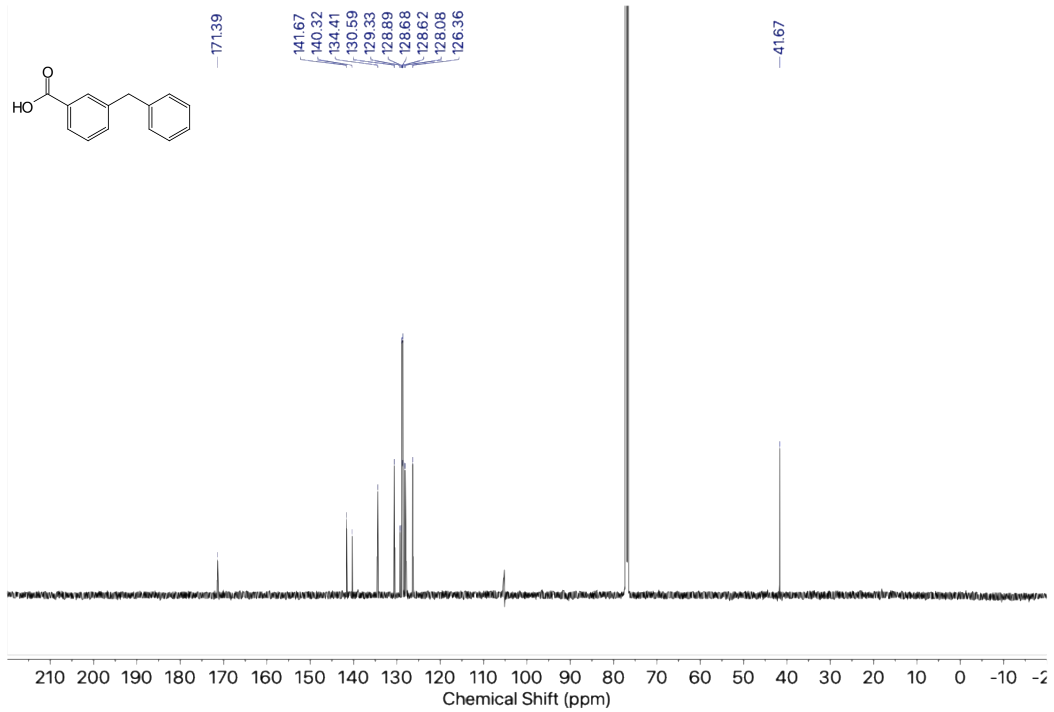
HO
O
171.39
141.67
140.32
134.41
130.59
129.33
128.89
128.68
128.62
128.08
126.36
41.67
Chemical Shift (ppm)

Compound 16

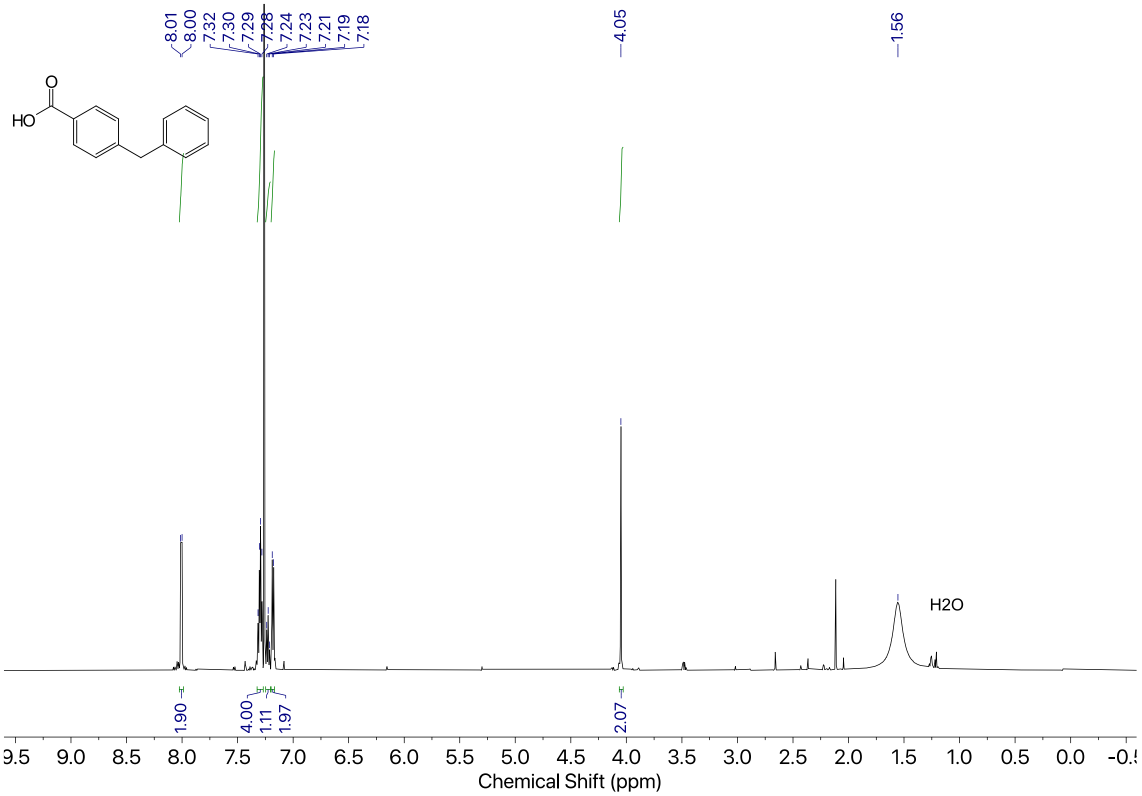

8.01
8.00
7.32
7.30
7.29
7.28
7.24
7.23
7.21
7.19
7.18
4.05
1.56
HO
O
H2O
1.90
4.00
1.11
1.97
2.07
9.5 9.0 8.5 8.0 7.5 7.0 6.5 6.0 5.5 5.0 4.5 4.0 3.5 3.0 2.5 2.0 1.5 1.0 0.5 0.0
Chemical Shift (ppm)

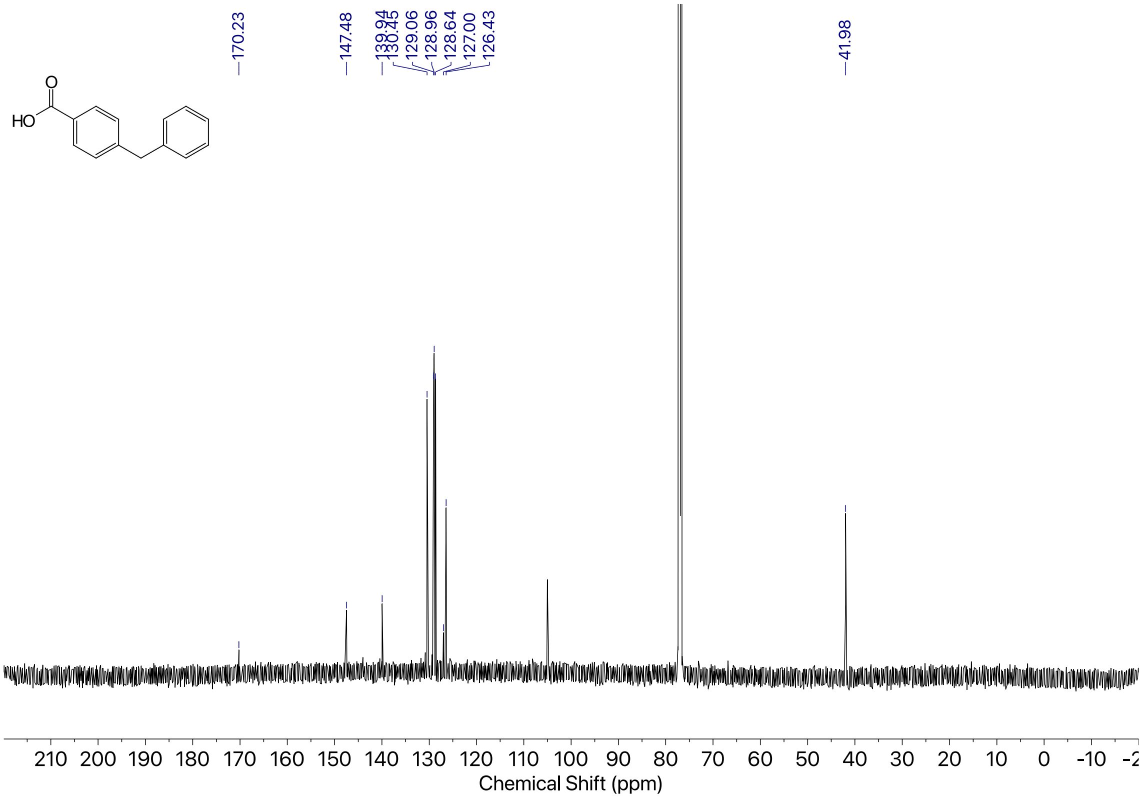

170.23
147.48
139.94
130.45
129.06
128.96
128.64
127.00
126.43
41.98
HO
O
210 200 190 180 170 160 150 140 130 120 110 100 90 80 70 60 50 40 30 20 10 0 -10
Chemical Shift (ppm)

Compound 18a

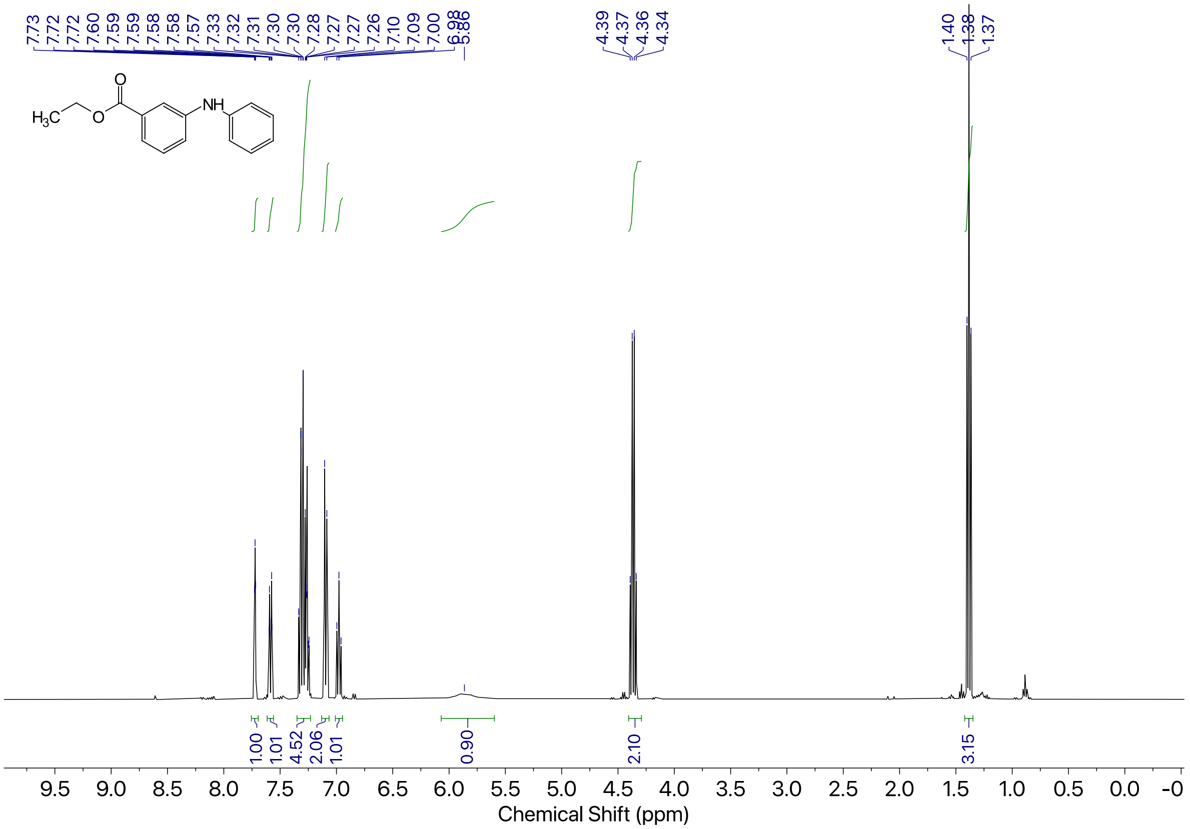

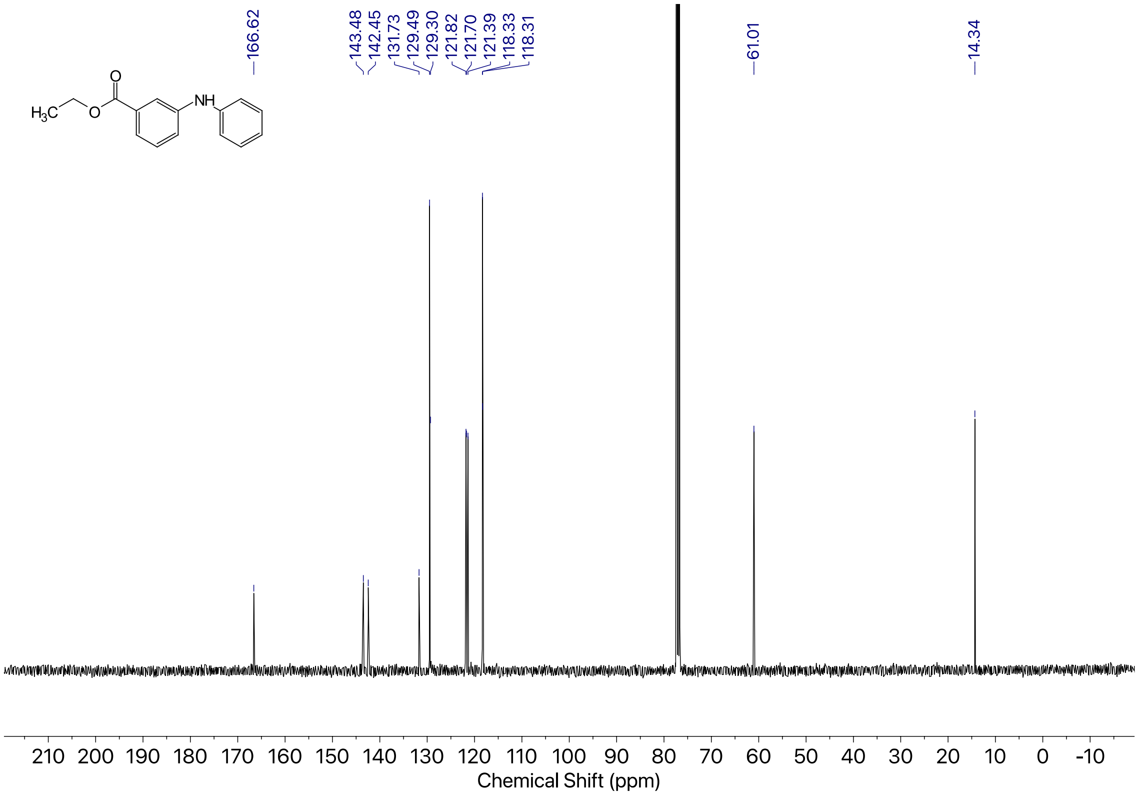

Compound 18b

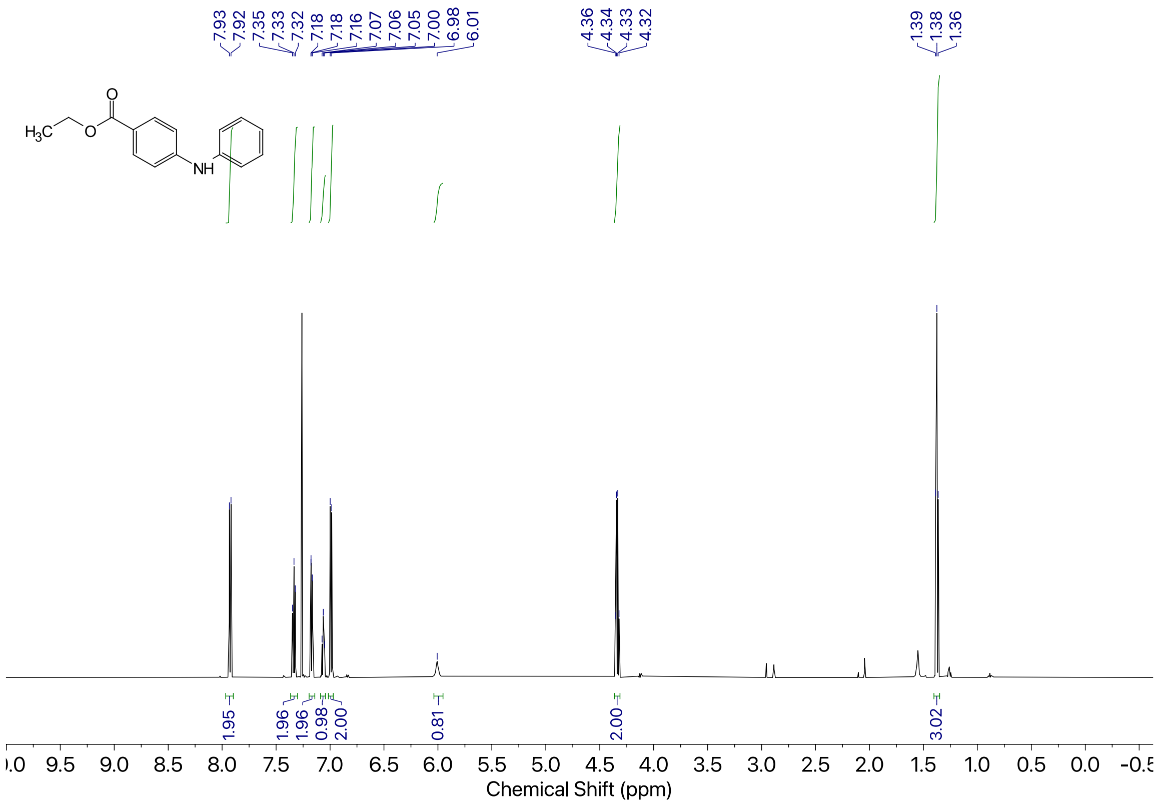

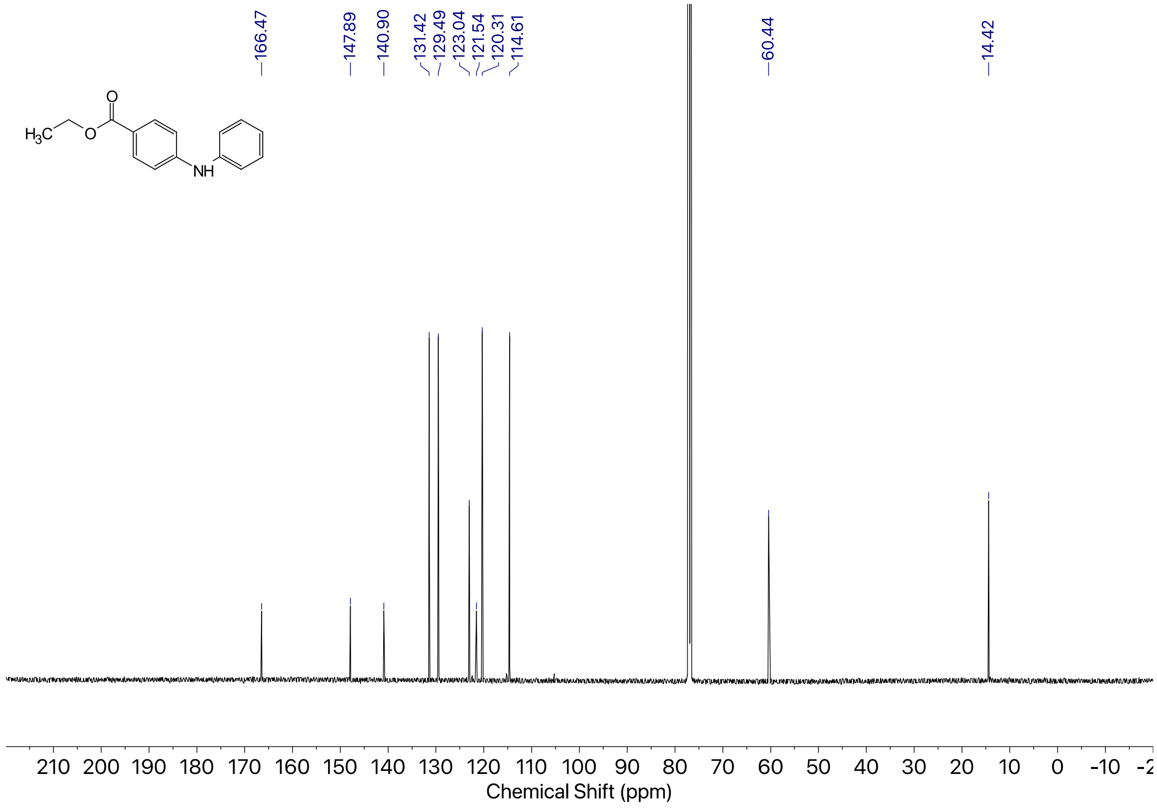

Compound 19a

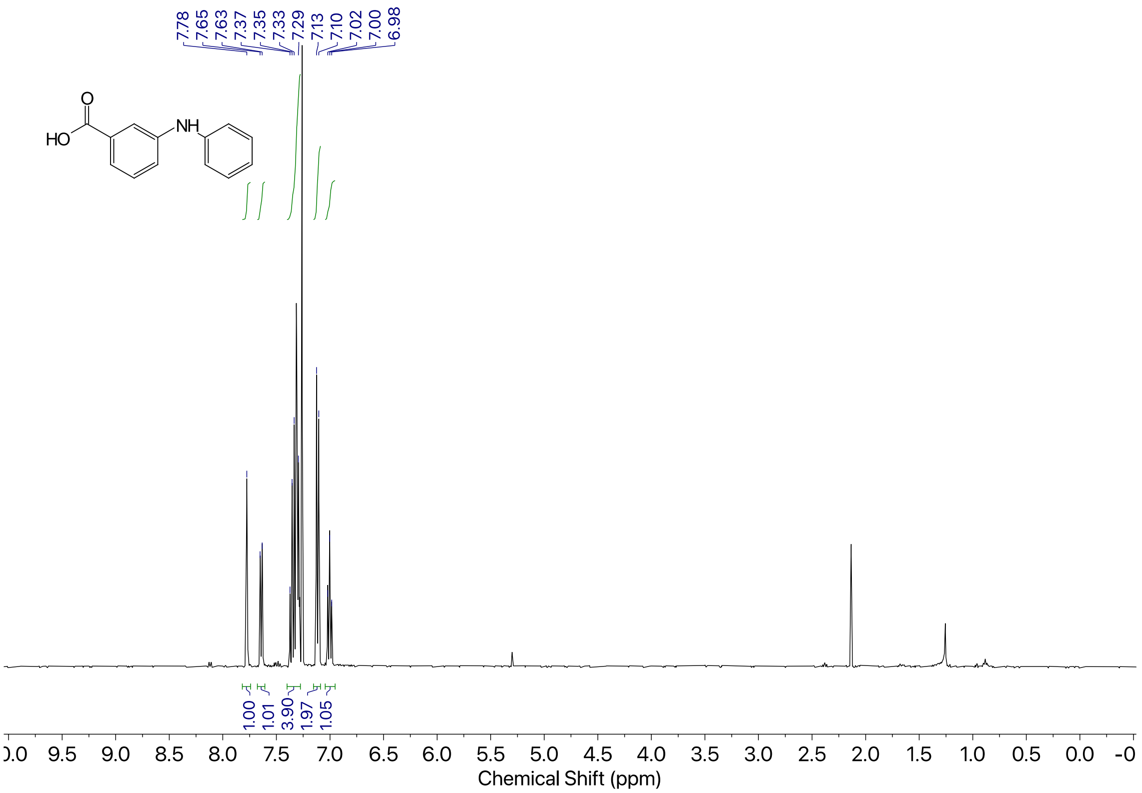

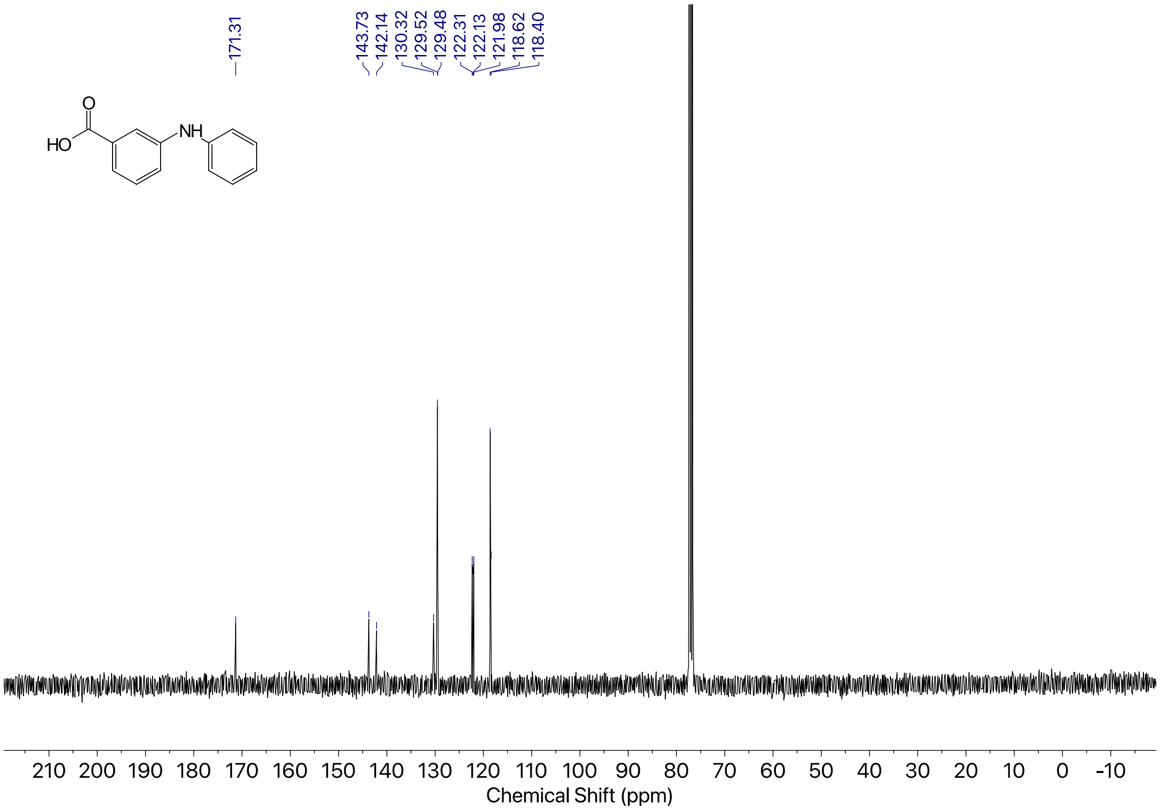

Compound 19b

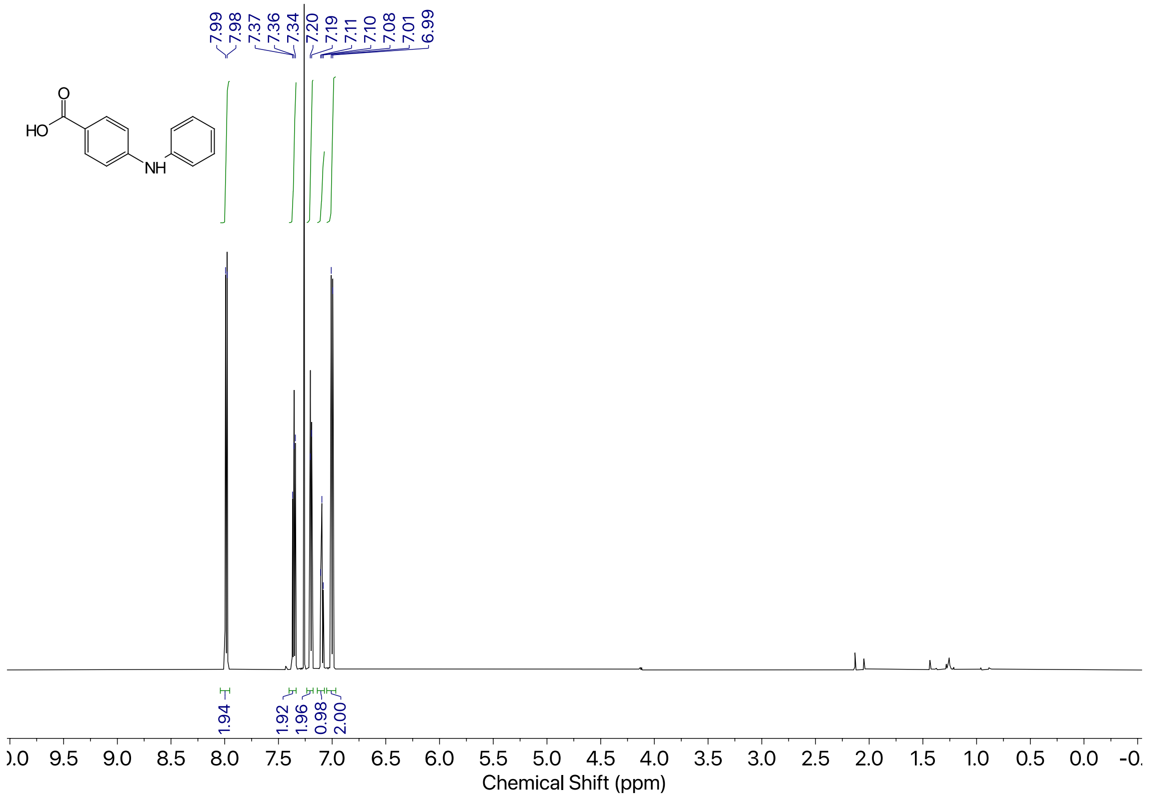

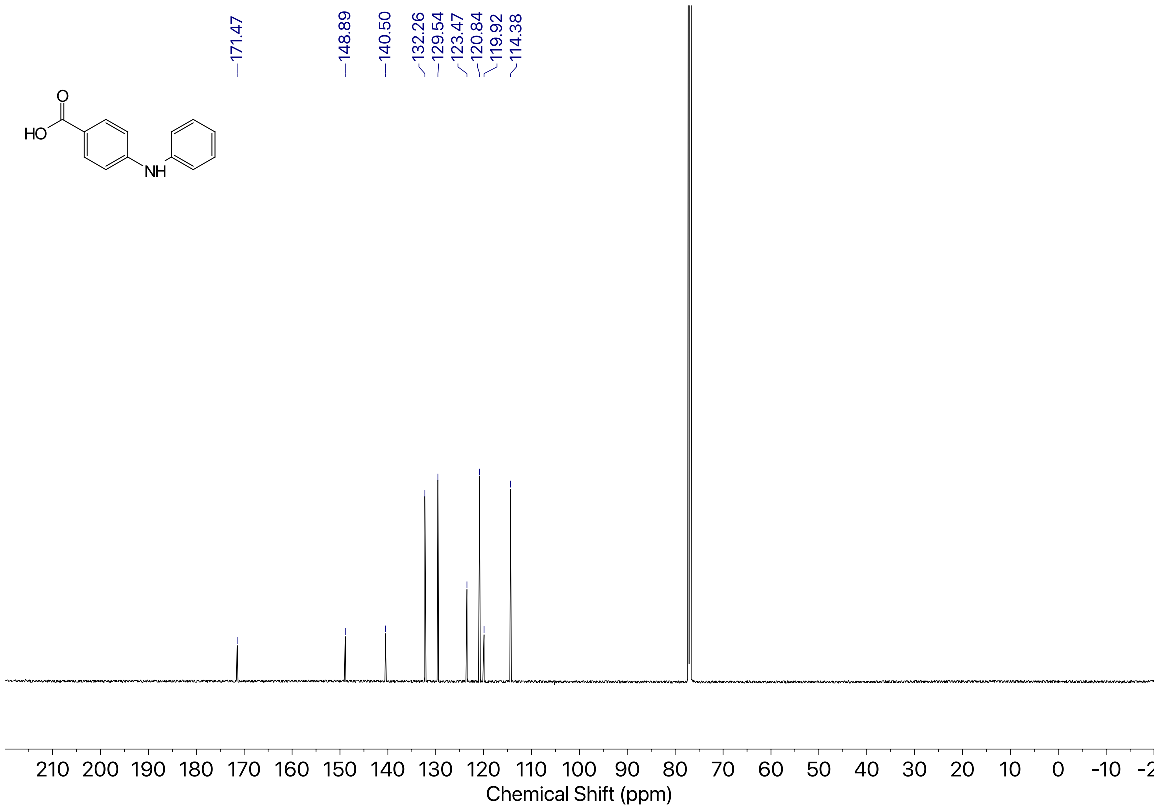

Compound 20a

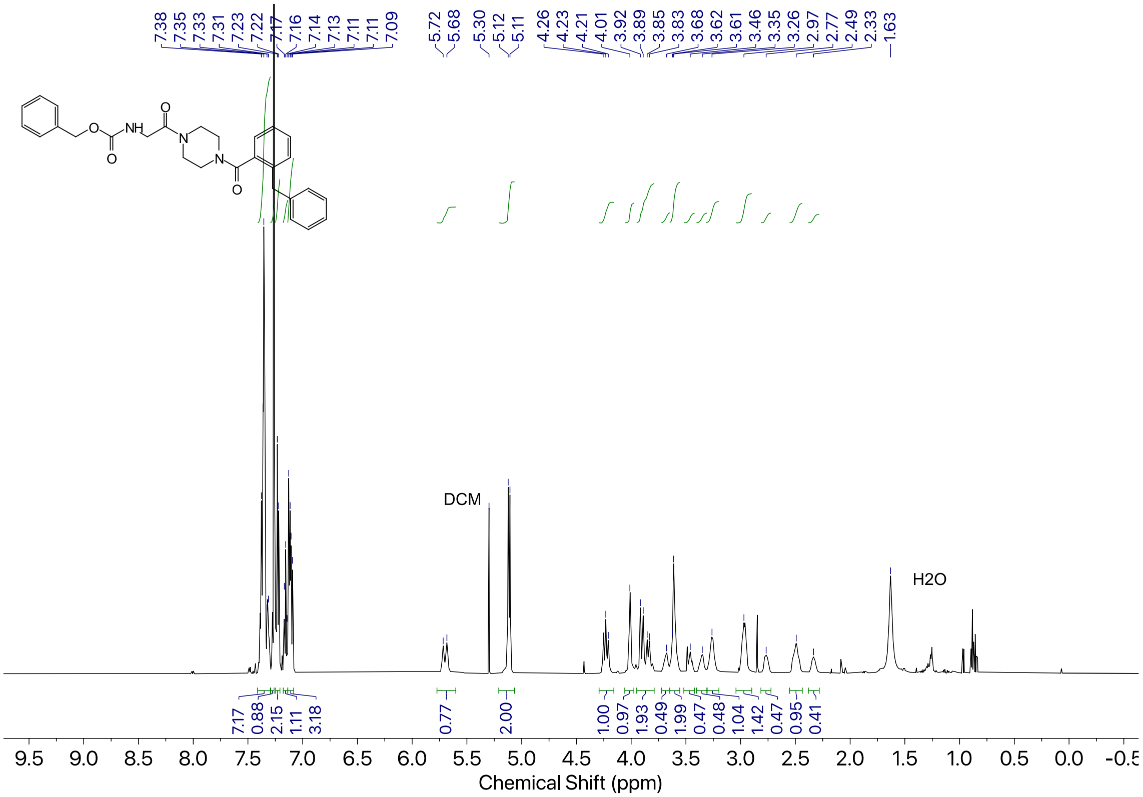

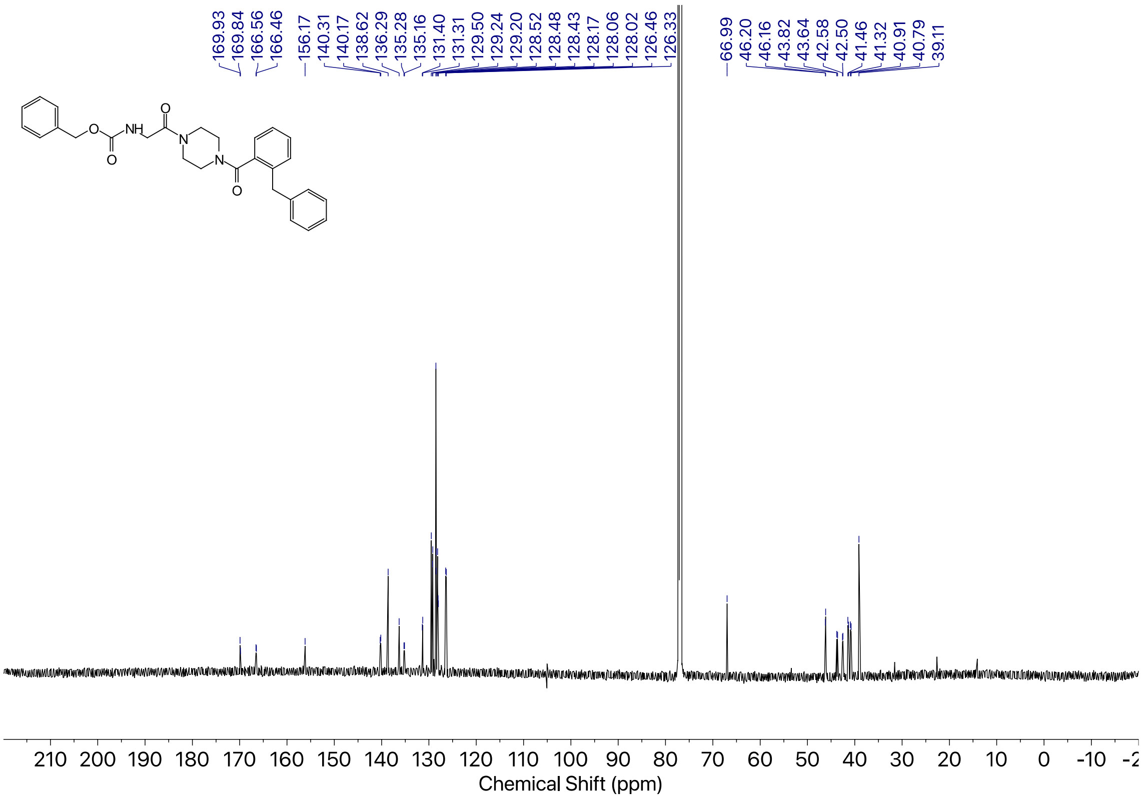

Compound 20b

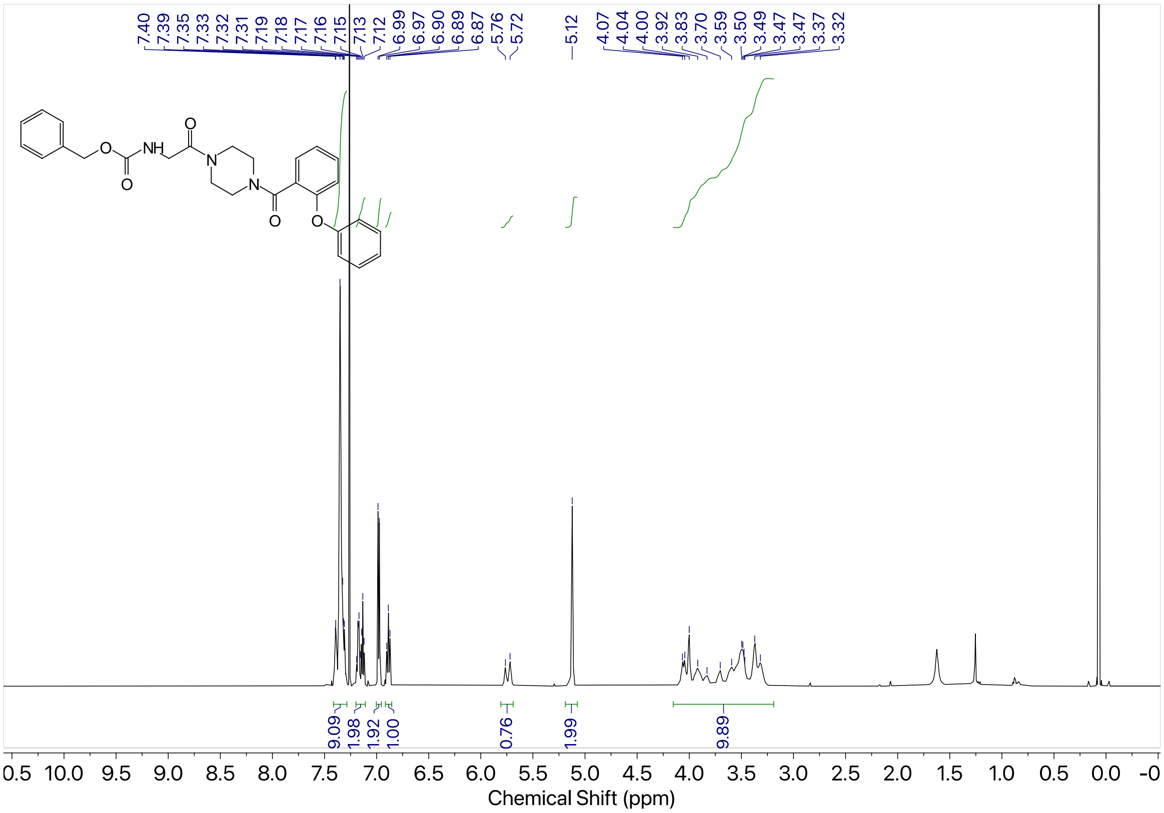

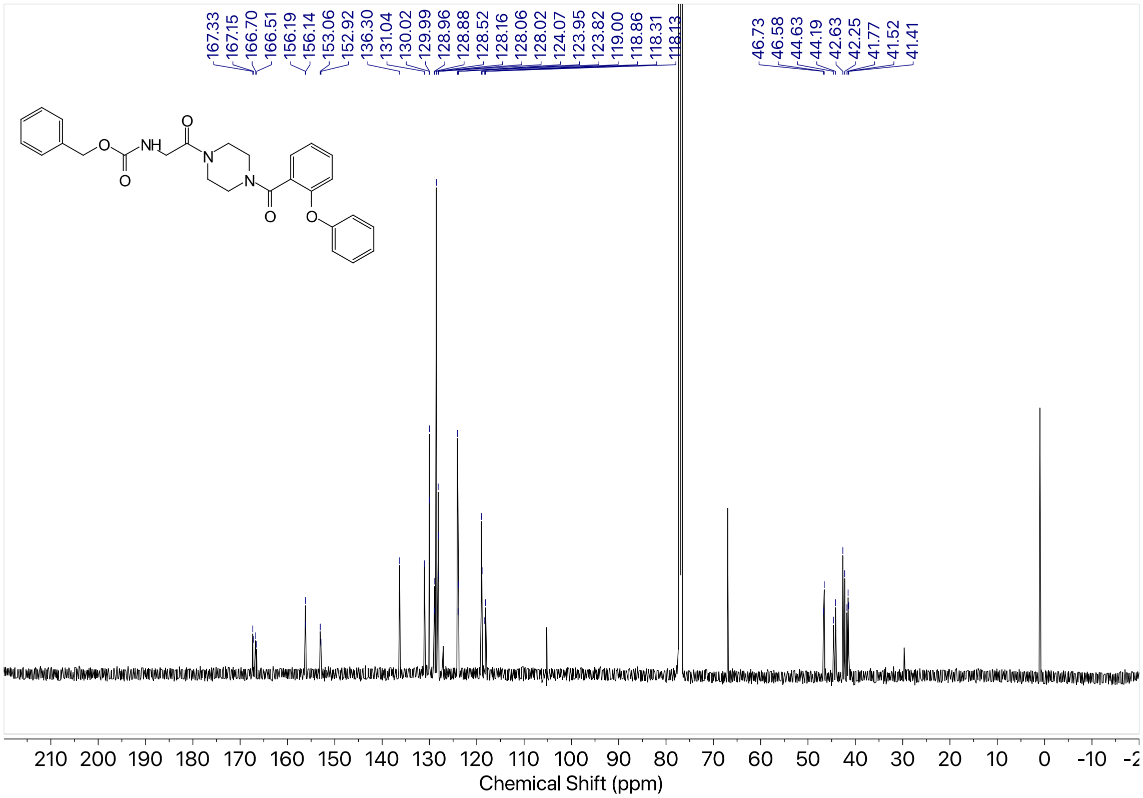

Compound 20c

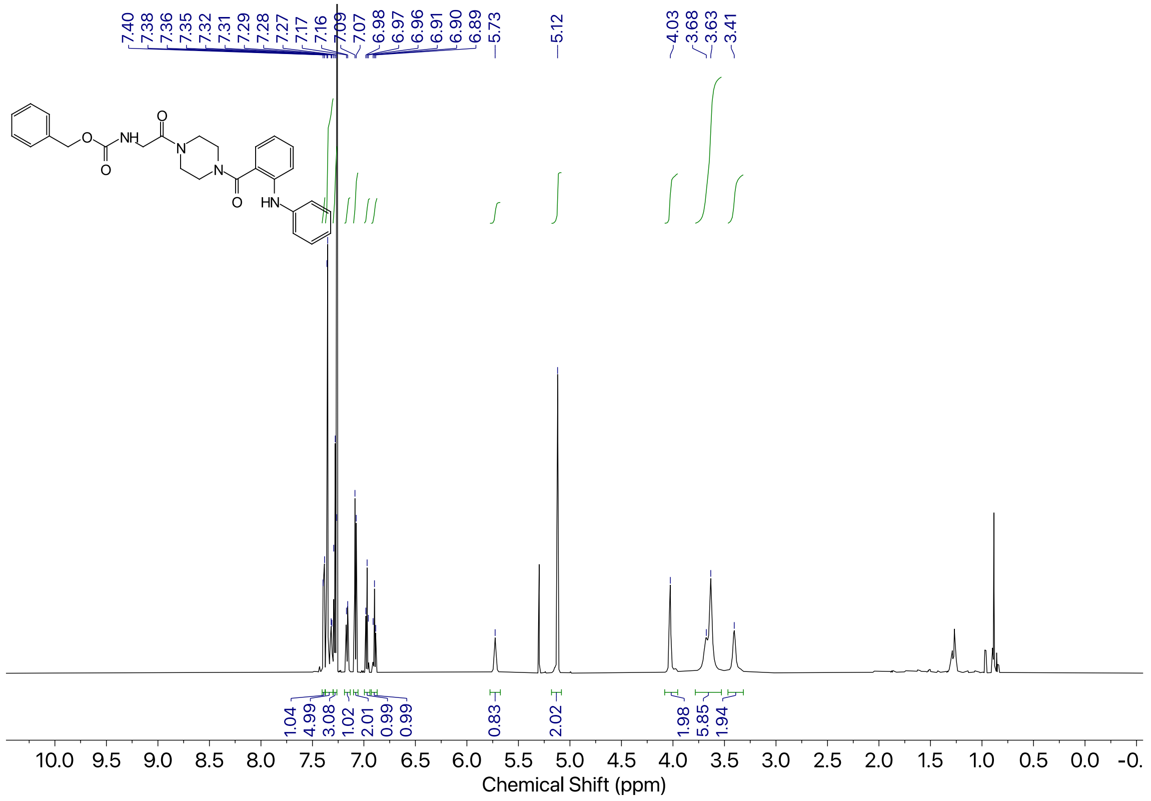

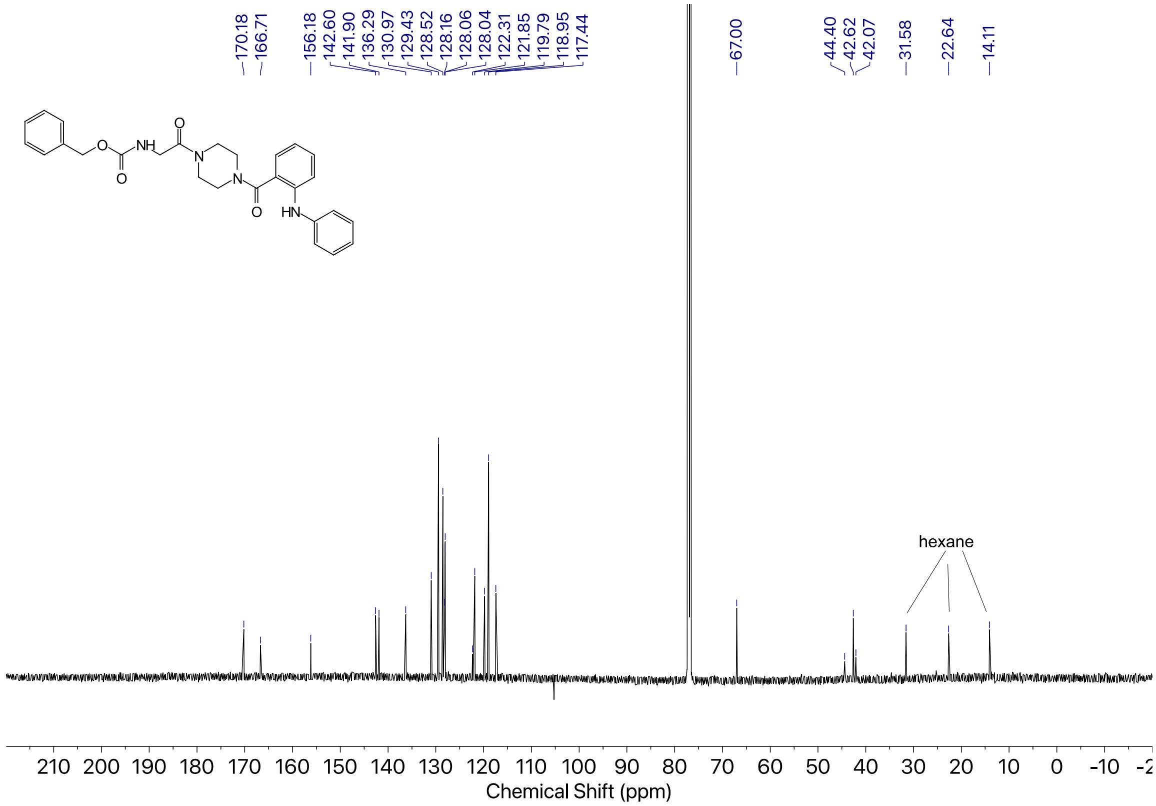

Compound 20d

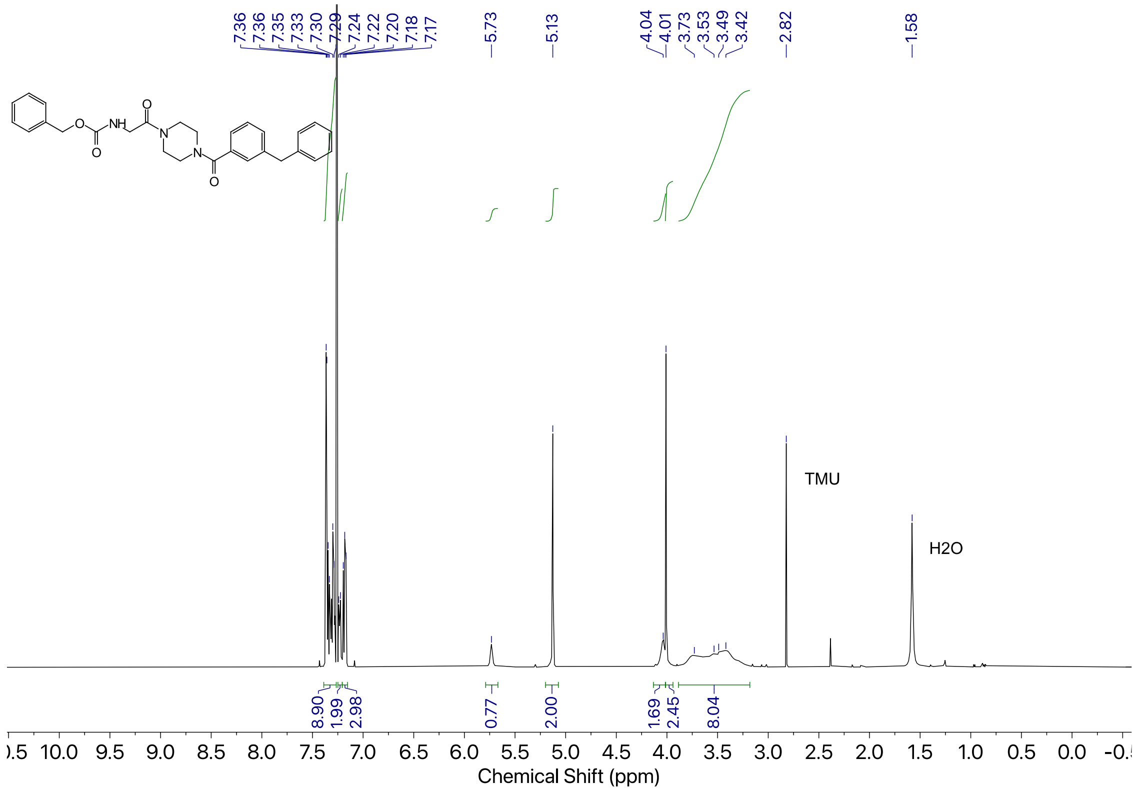

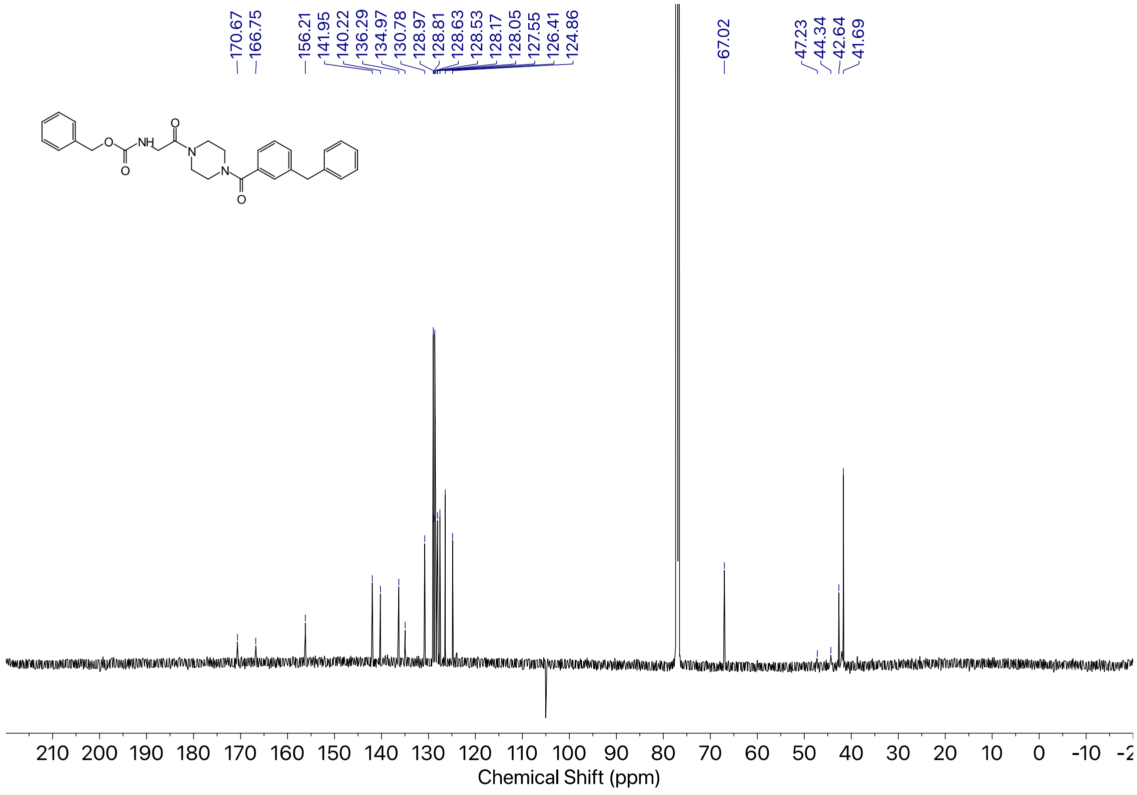

Compound 20e

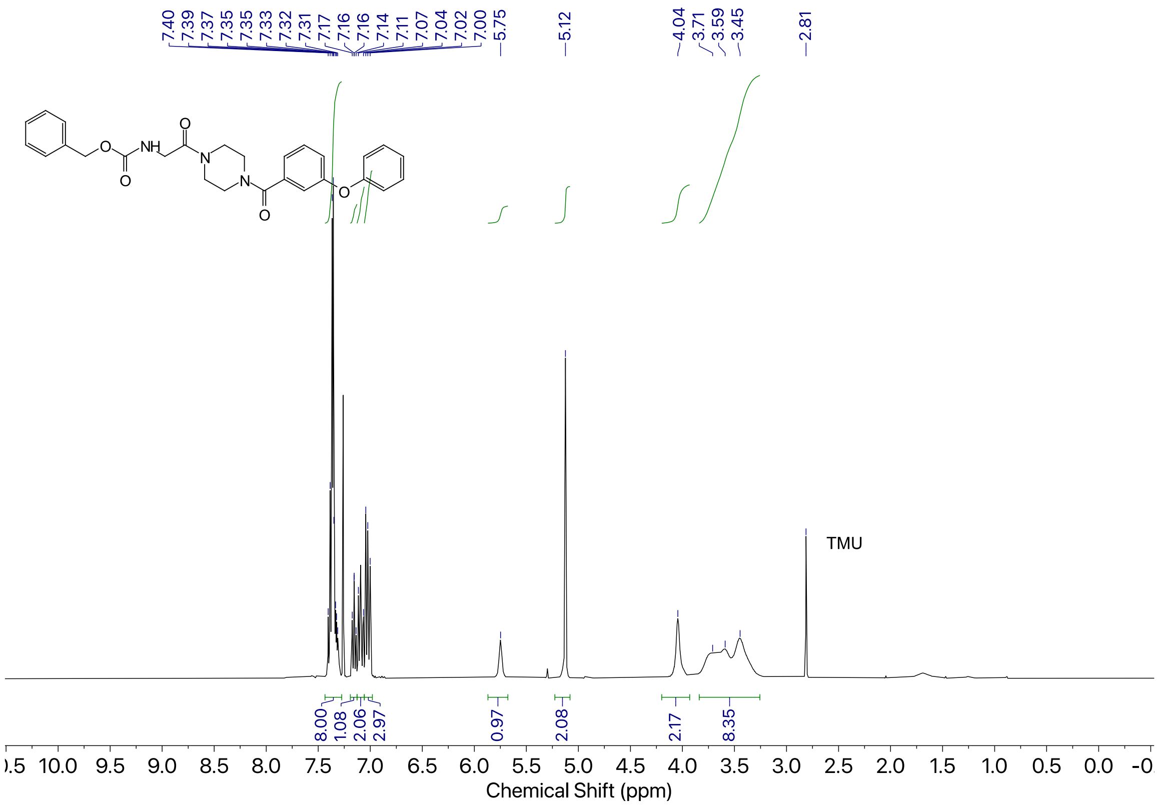

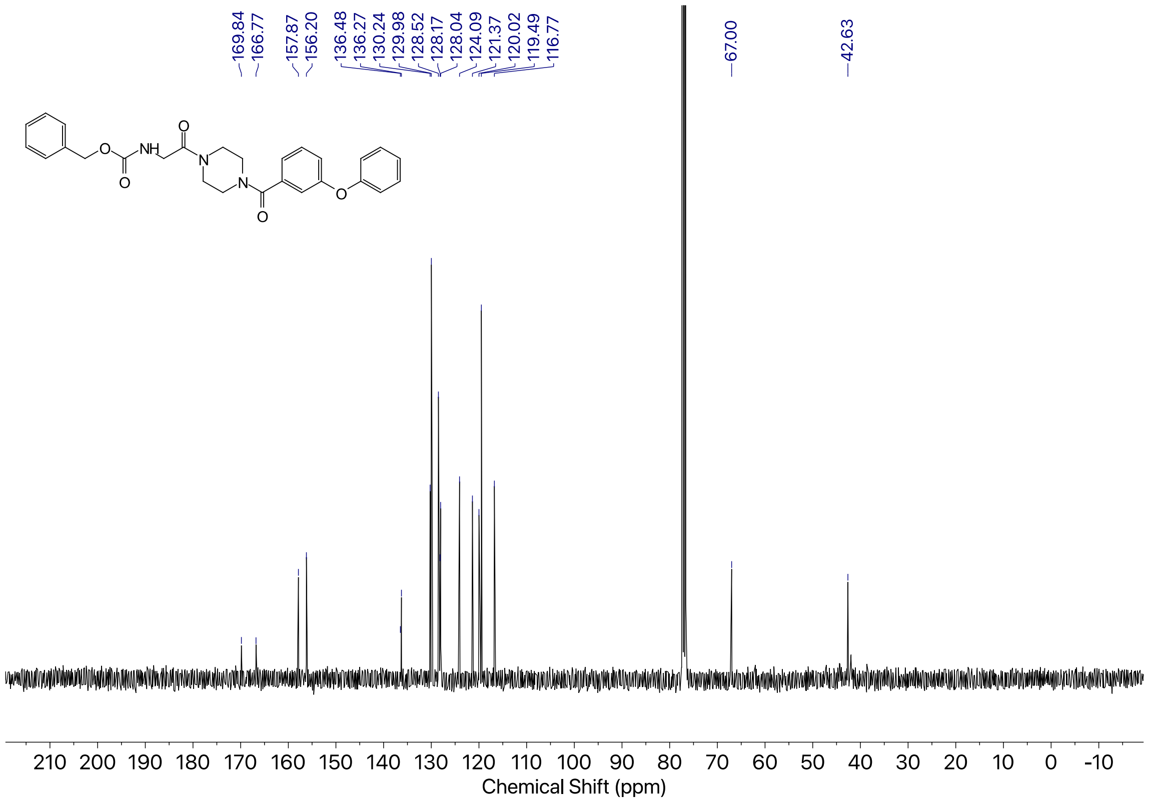

Compound 20f

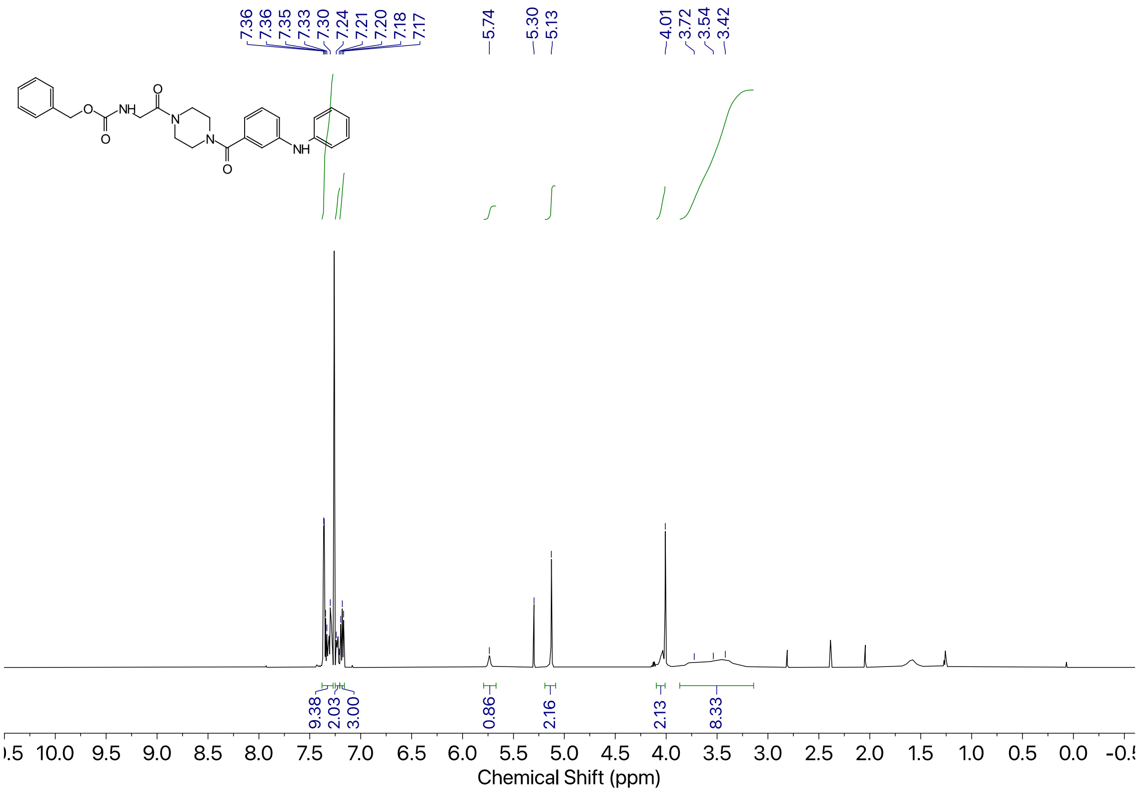

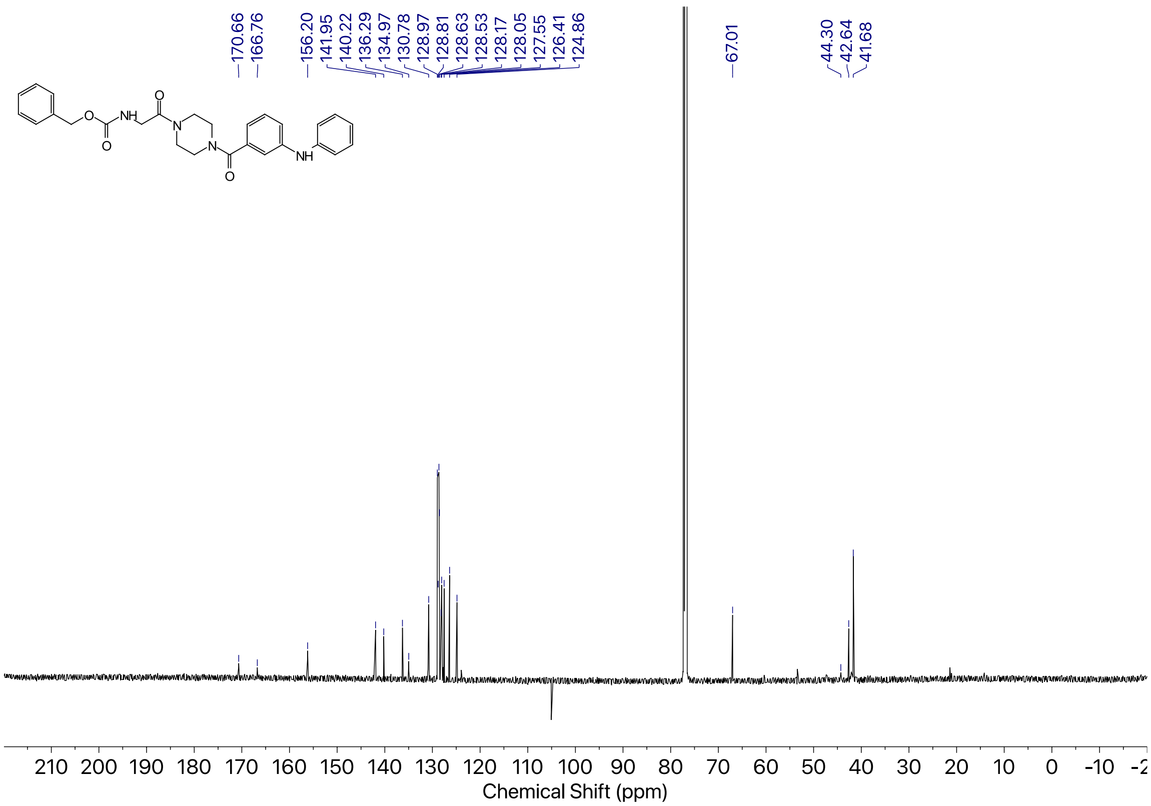

Compound 20g

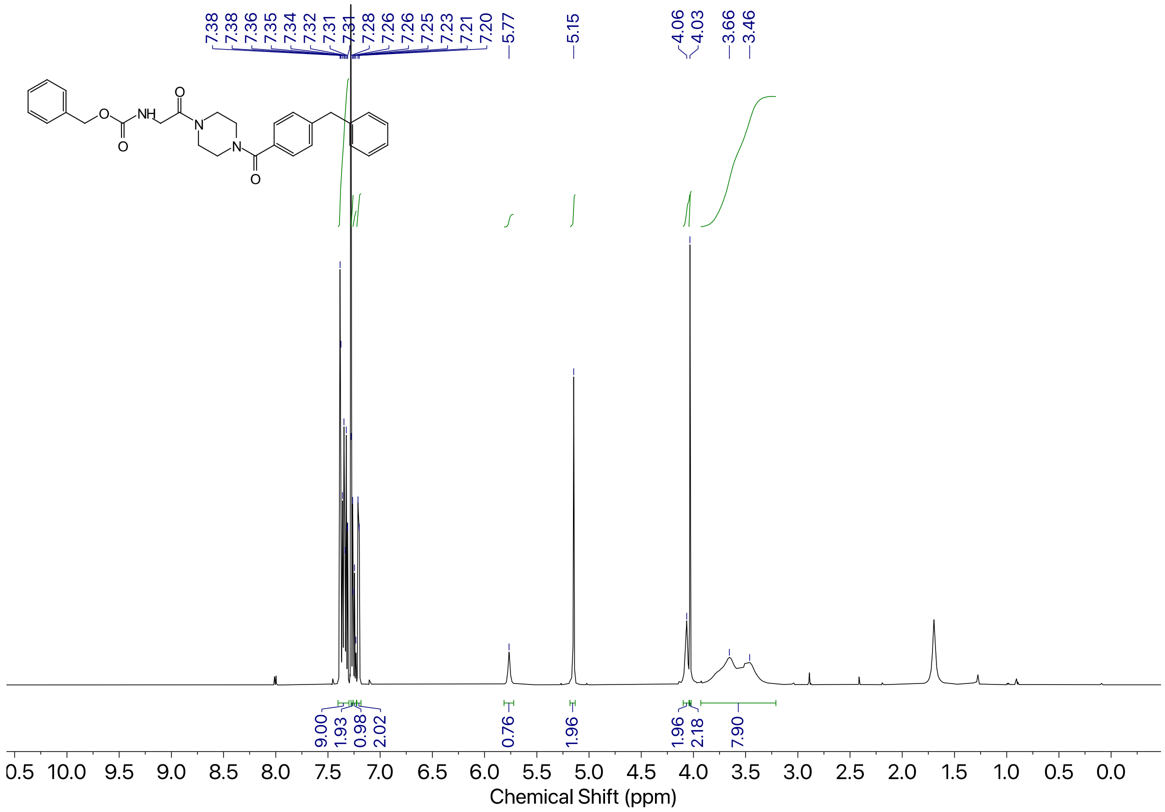

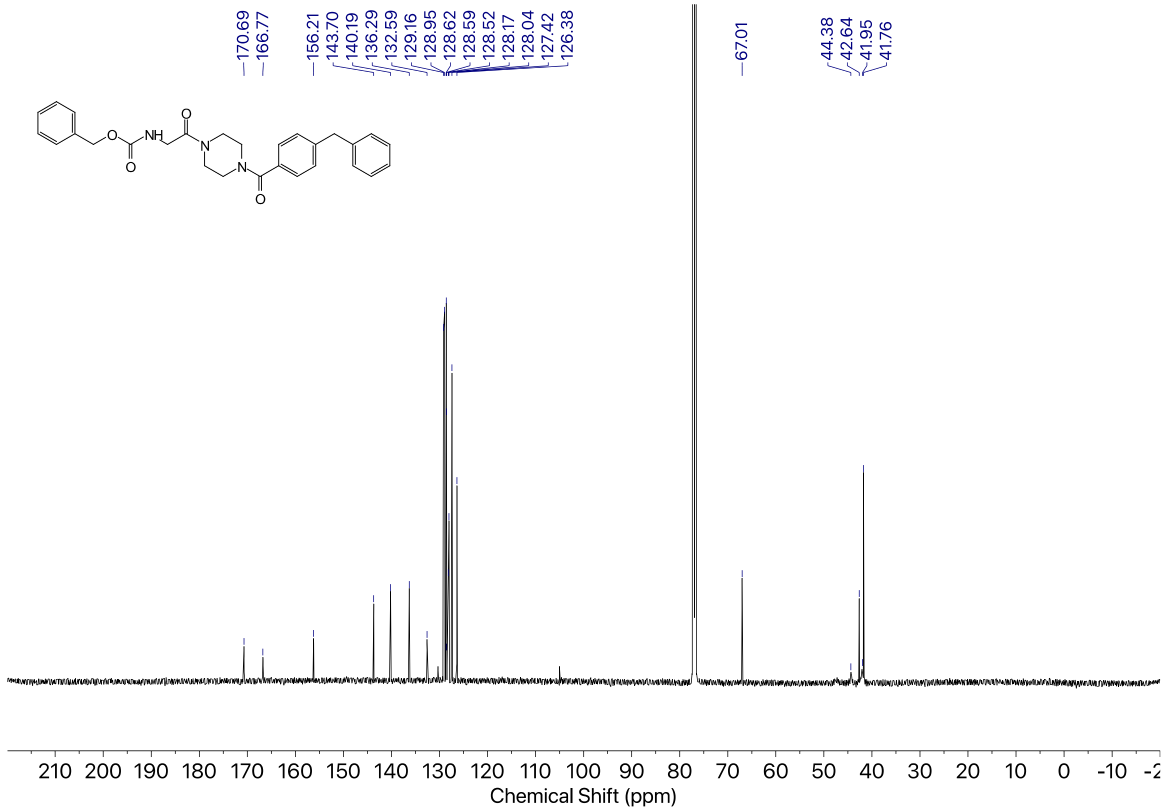

Compound 20h

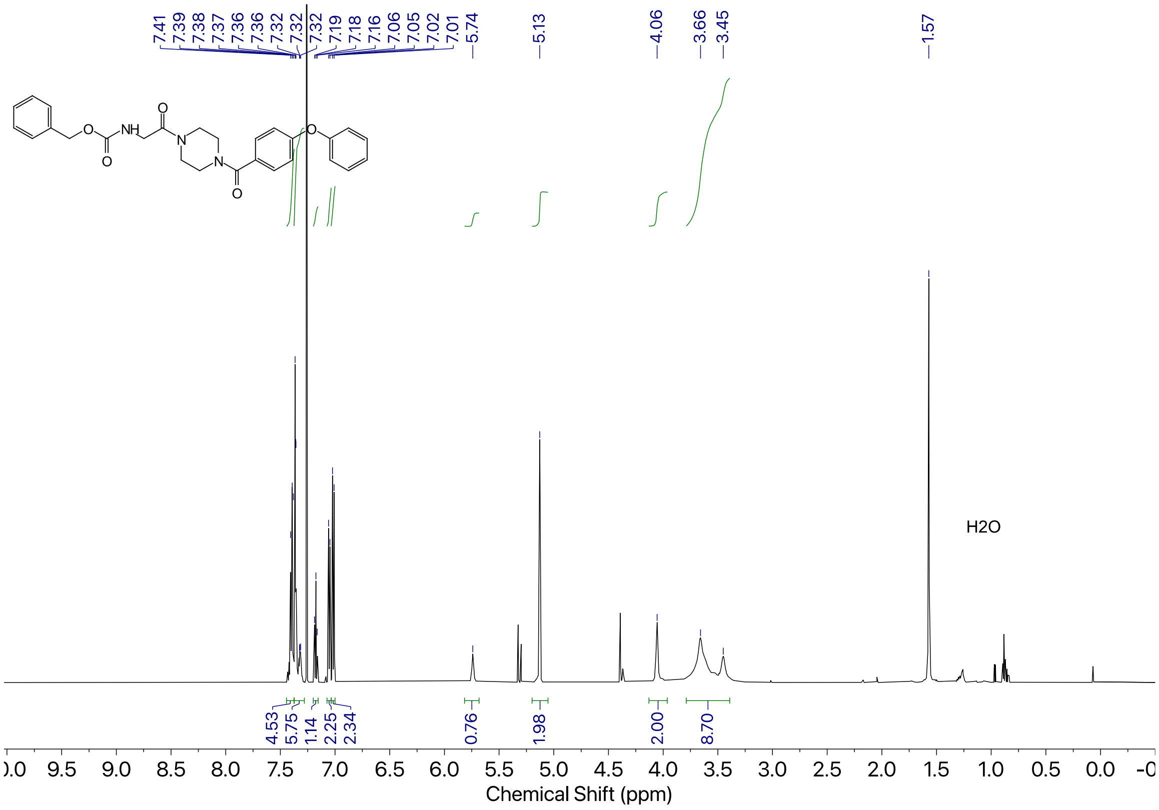

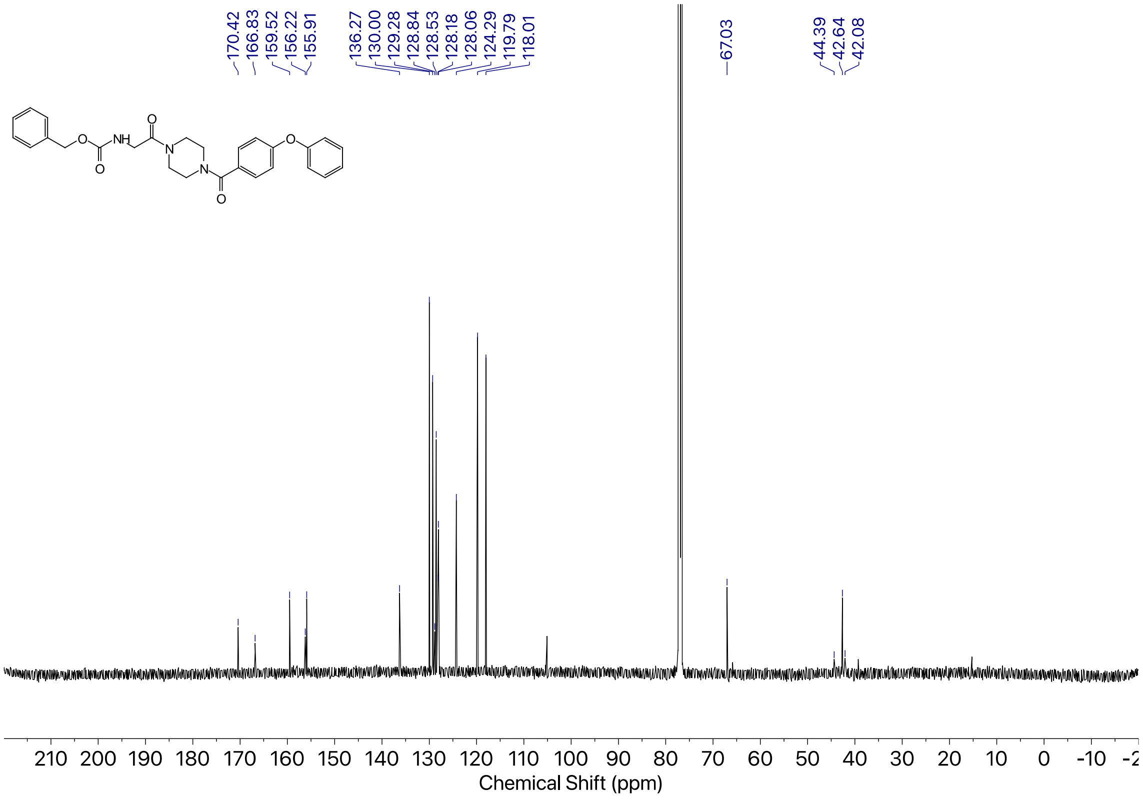

Compound 20i

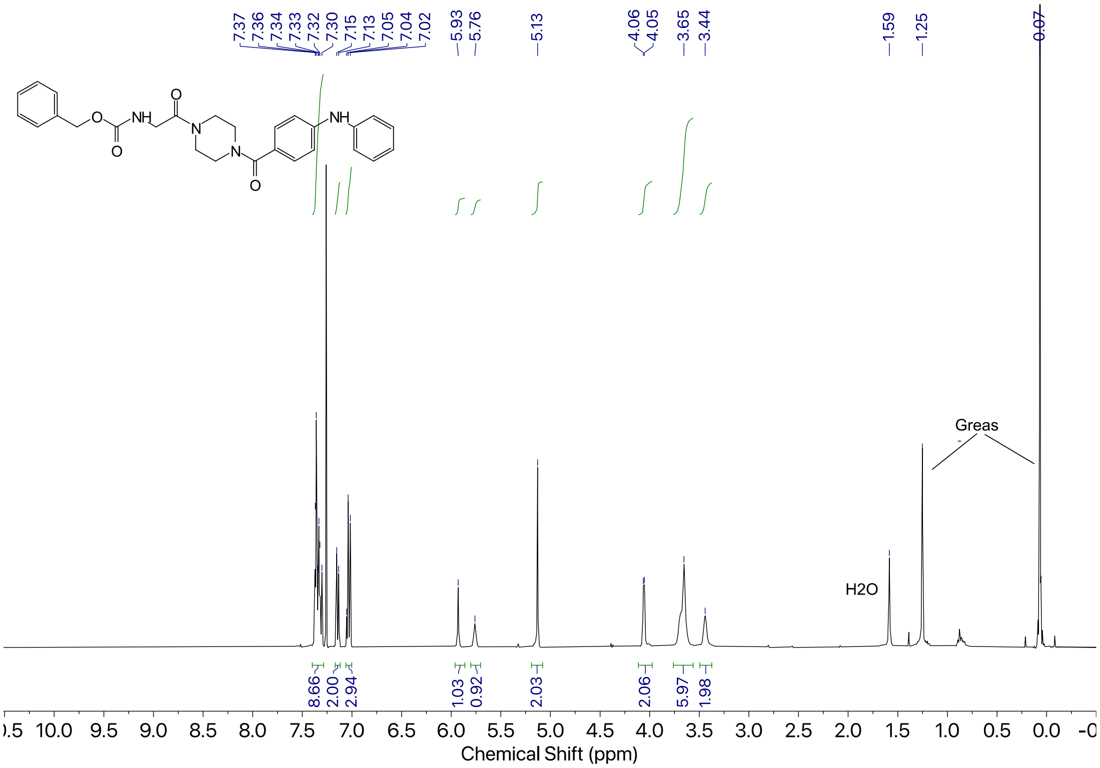

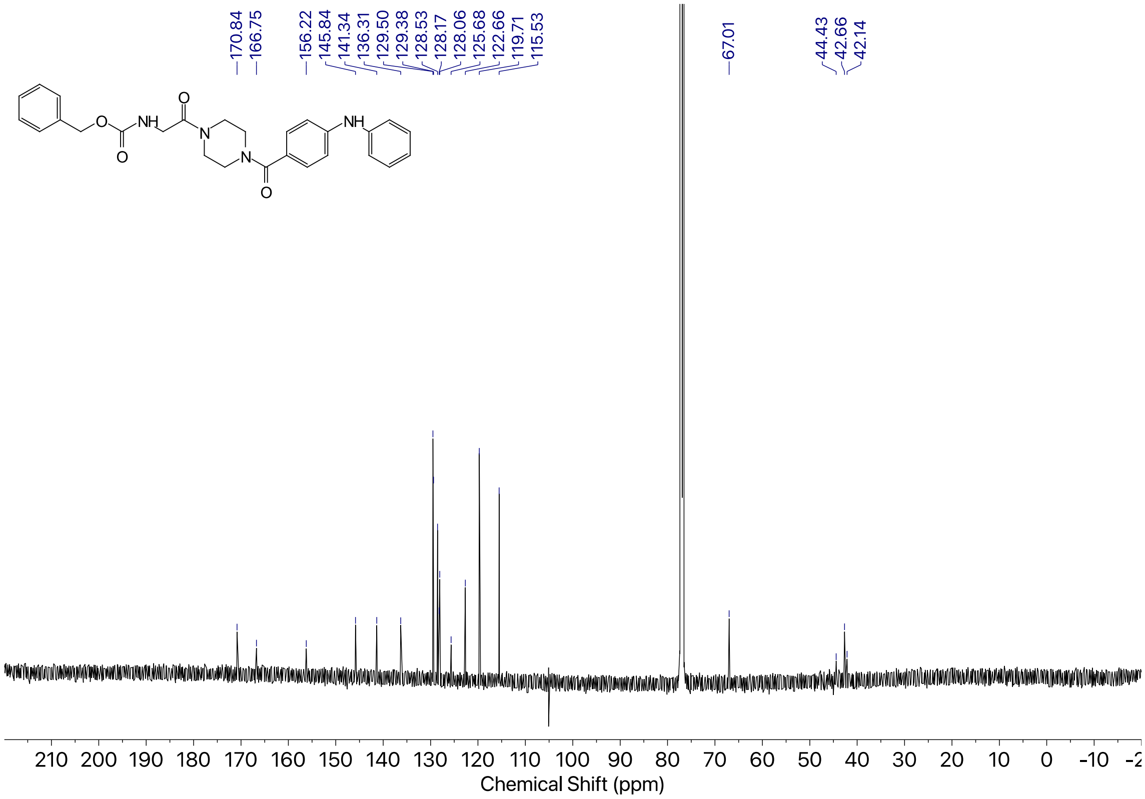

Compound 21a

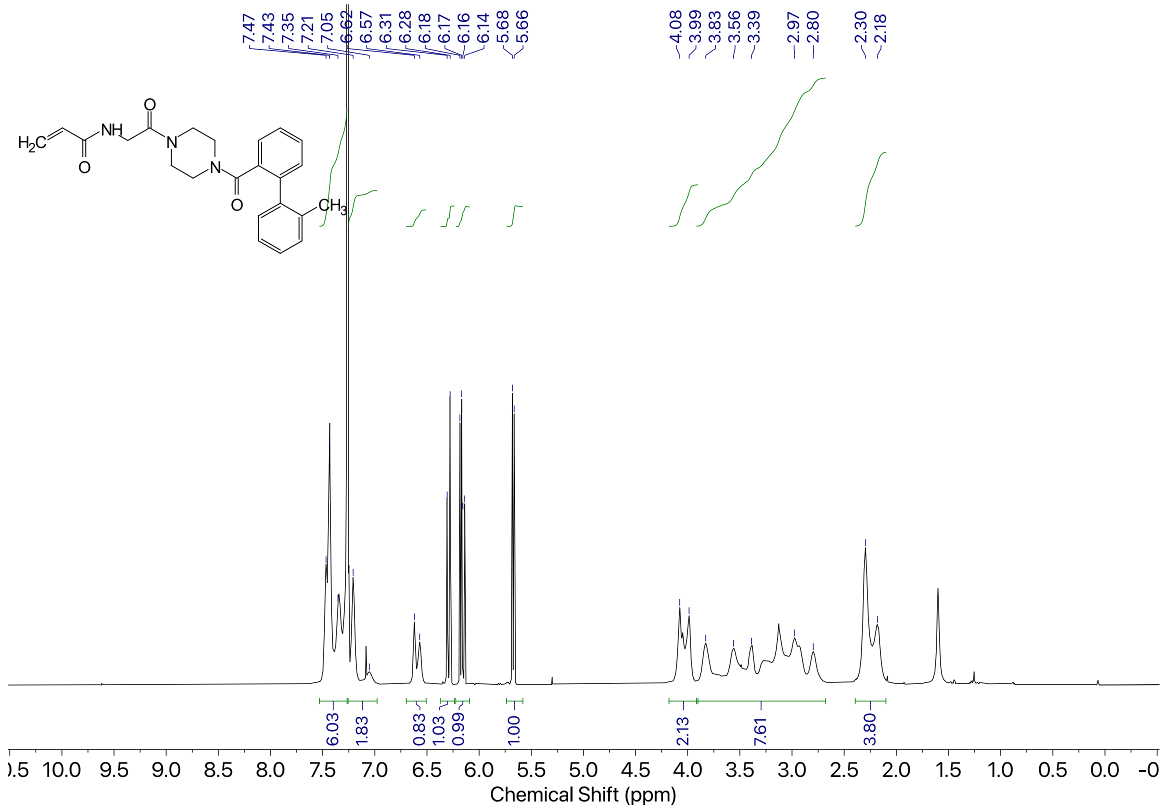

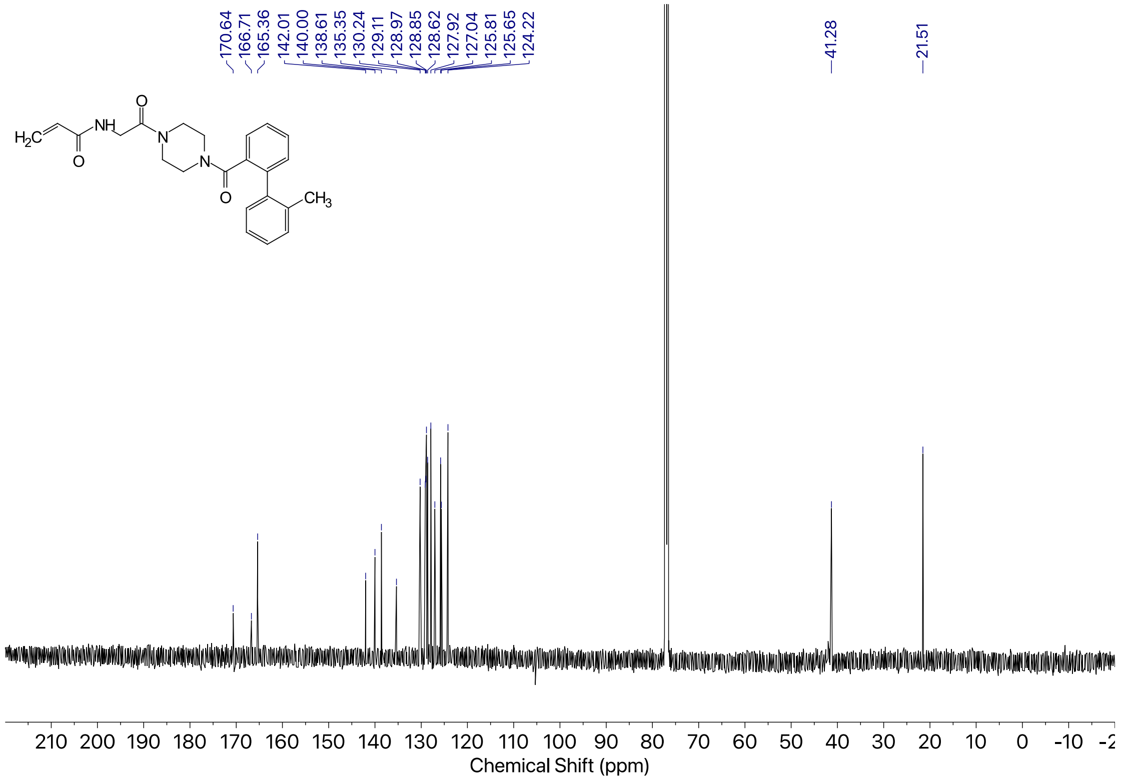

Compound 21b

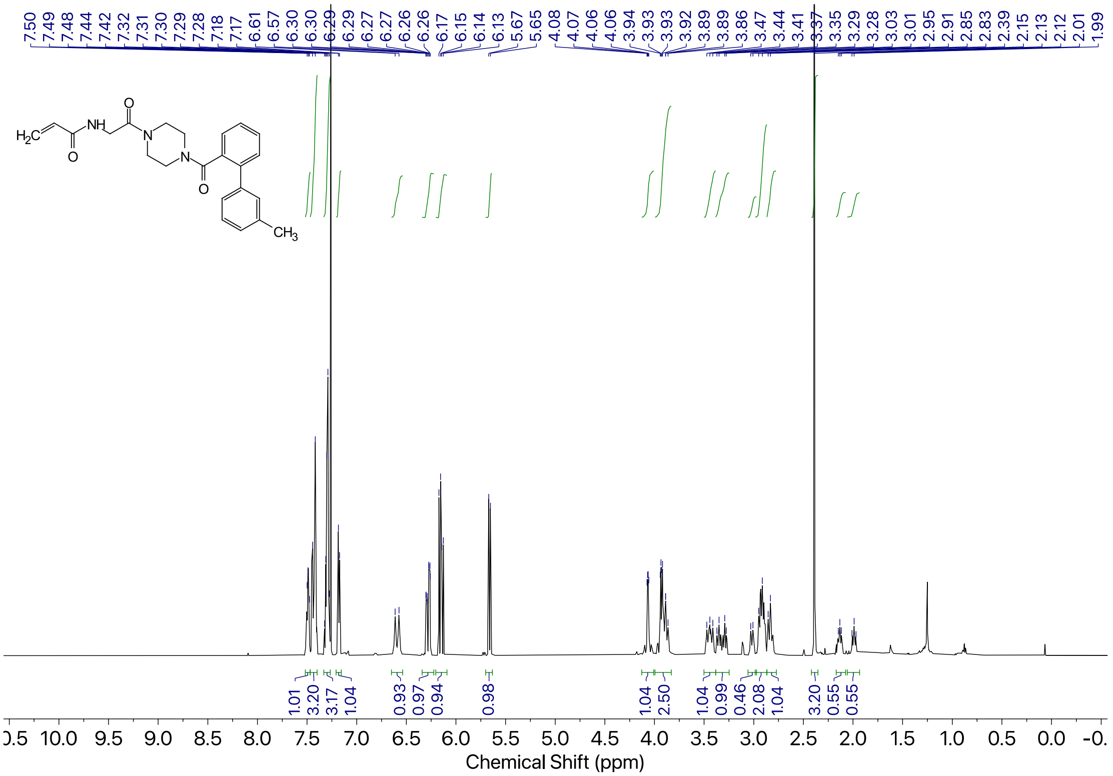

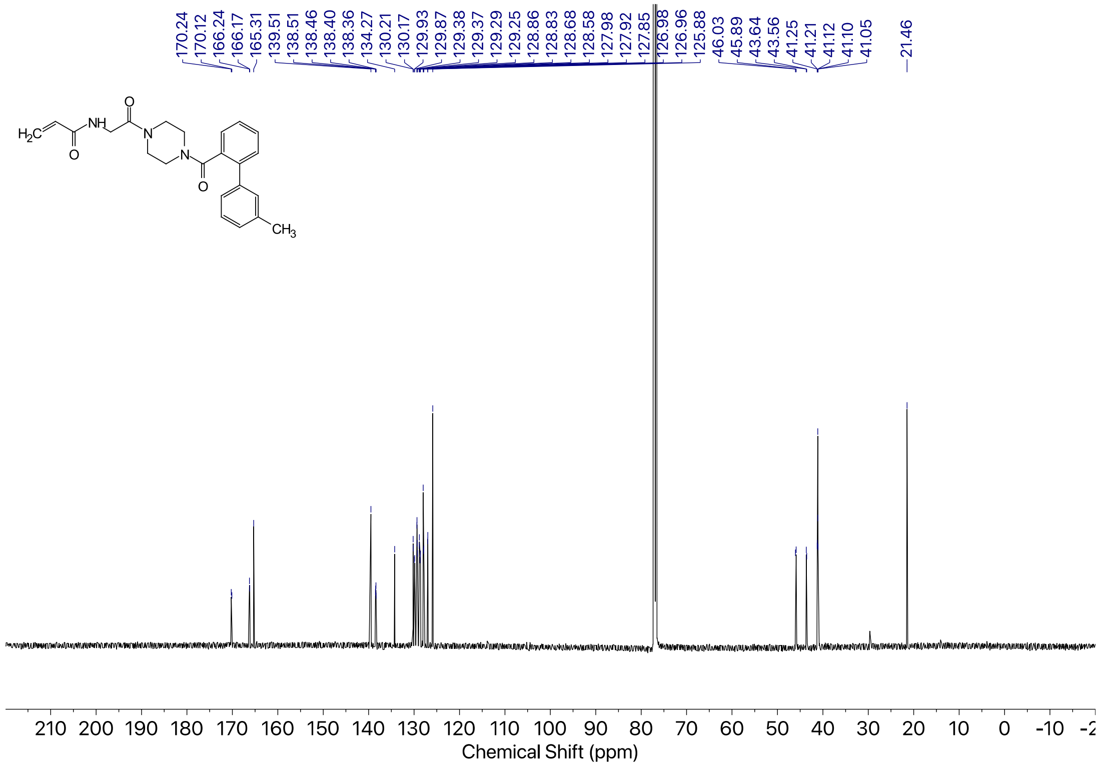

Compound 21c

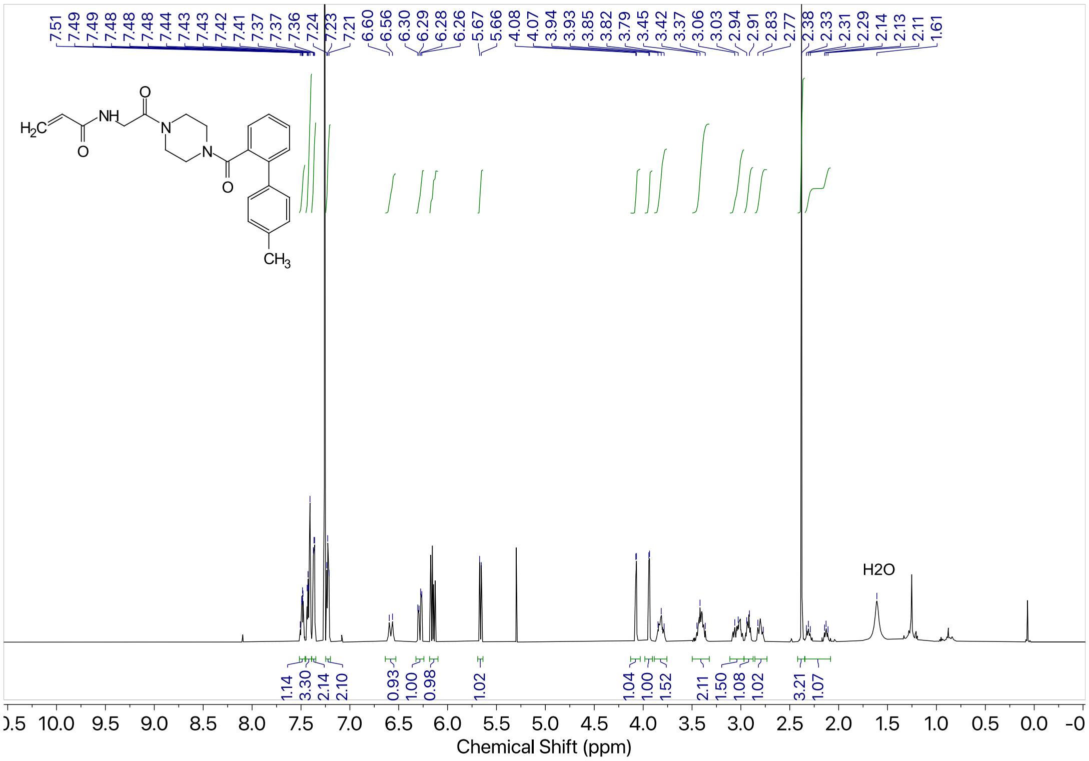

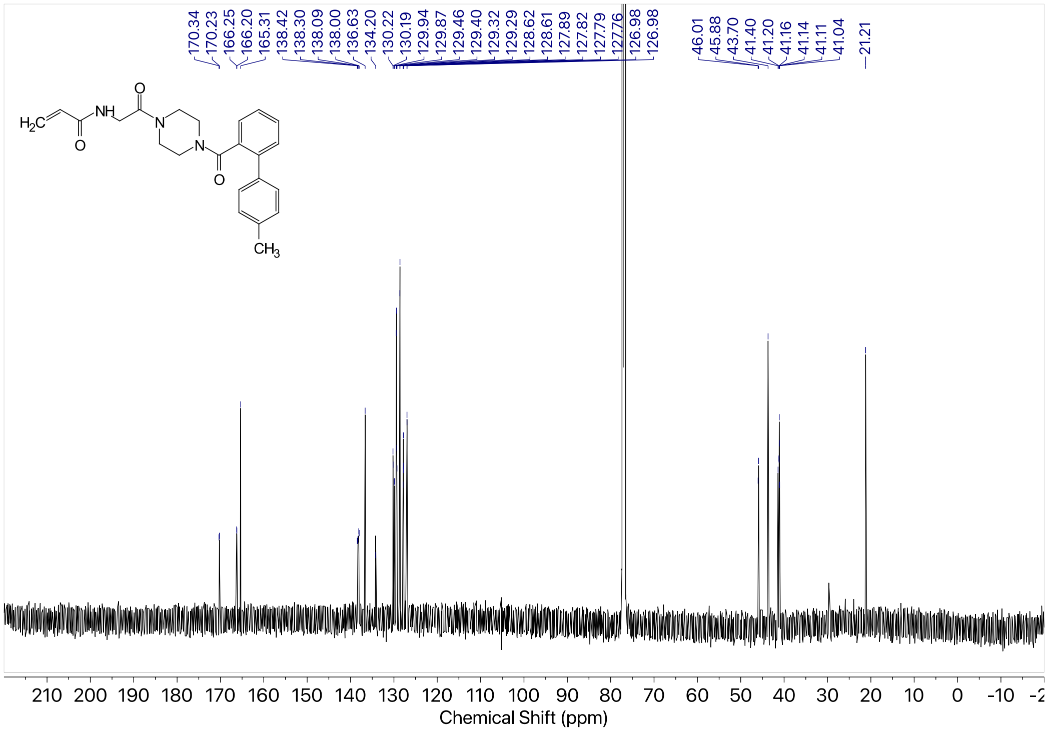

Compound 21d

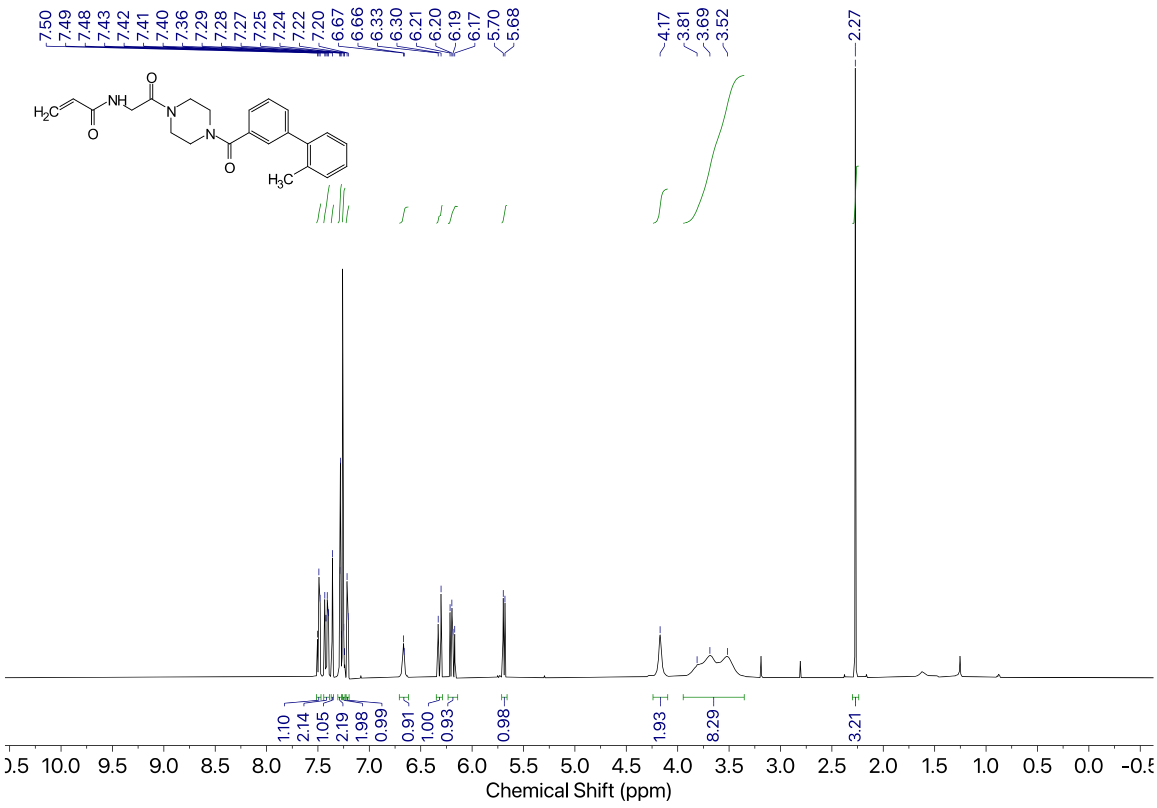

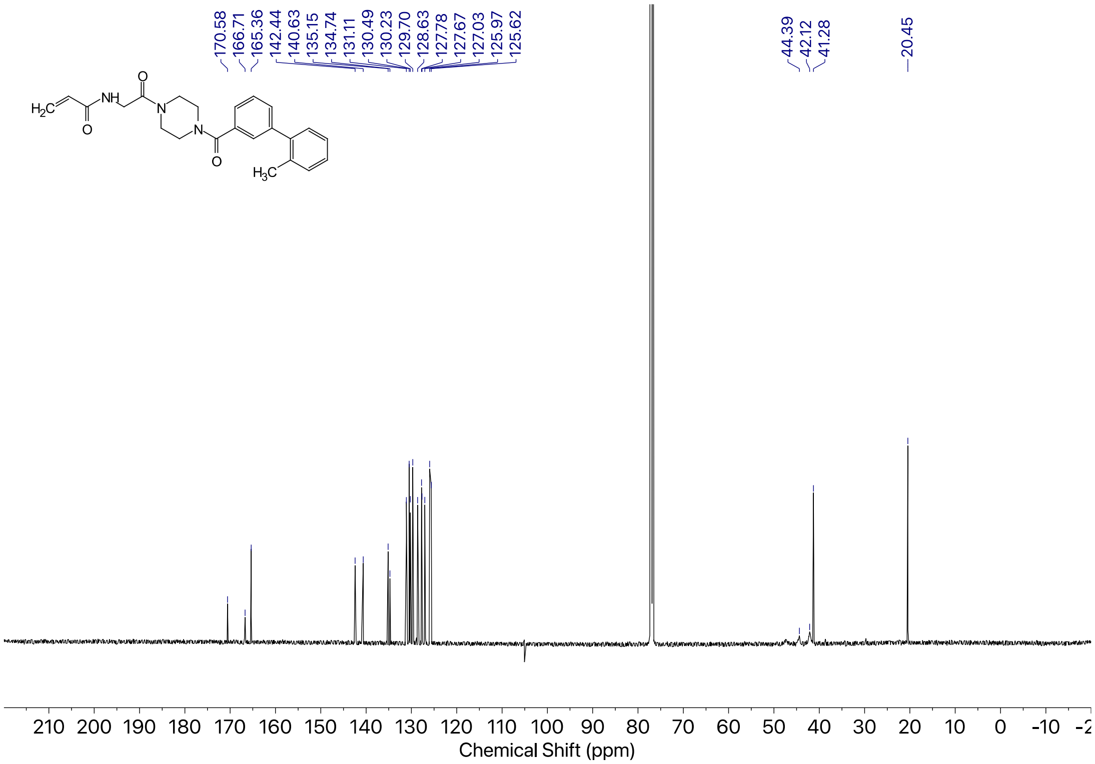

Compound 21e

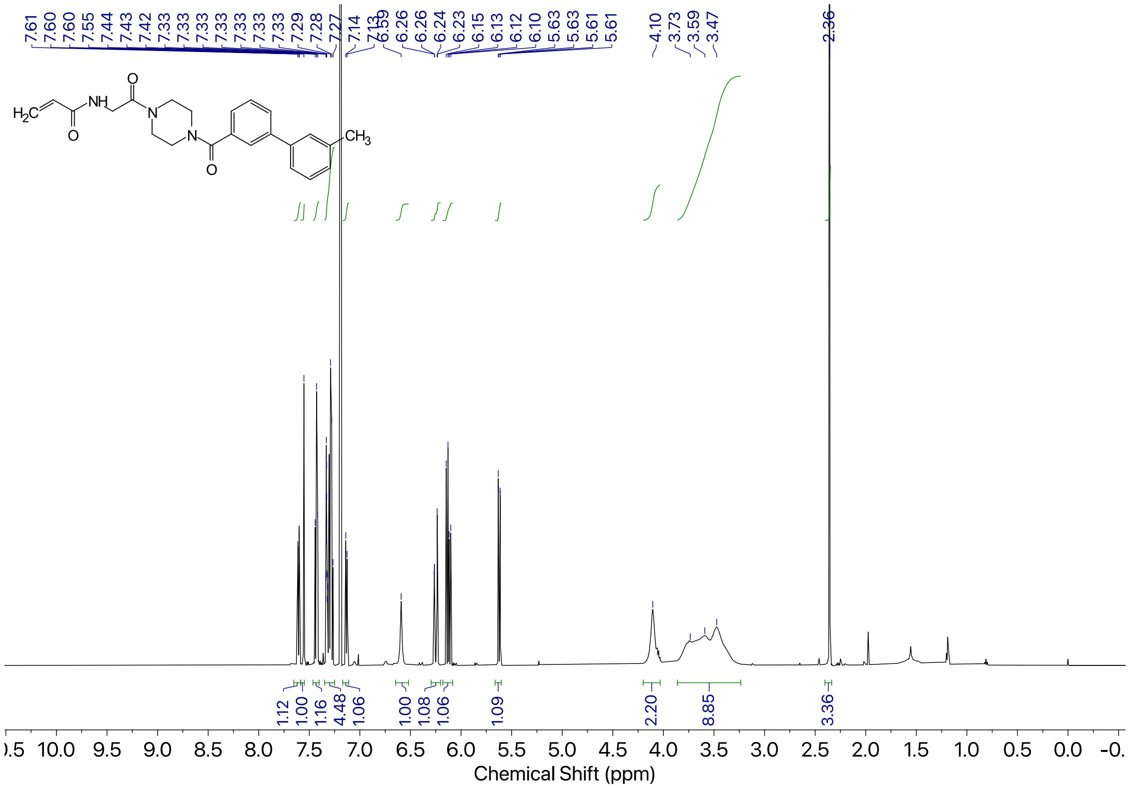

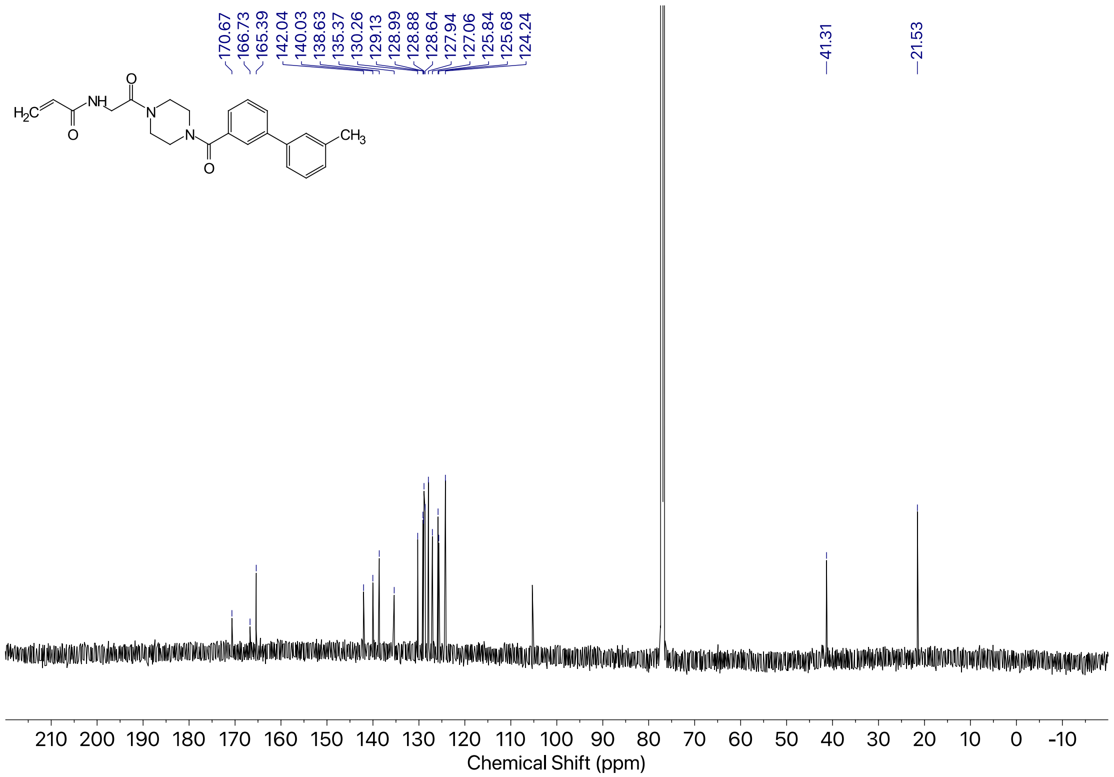

Compound 21f

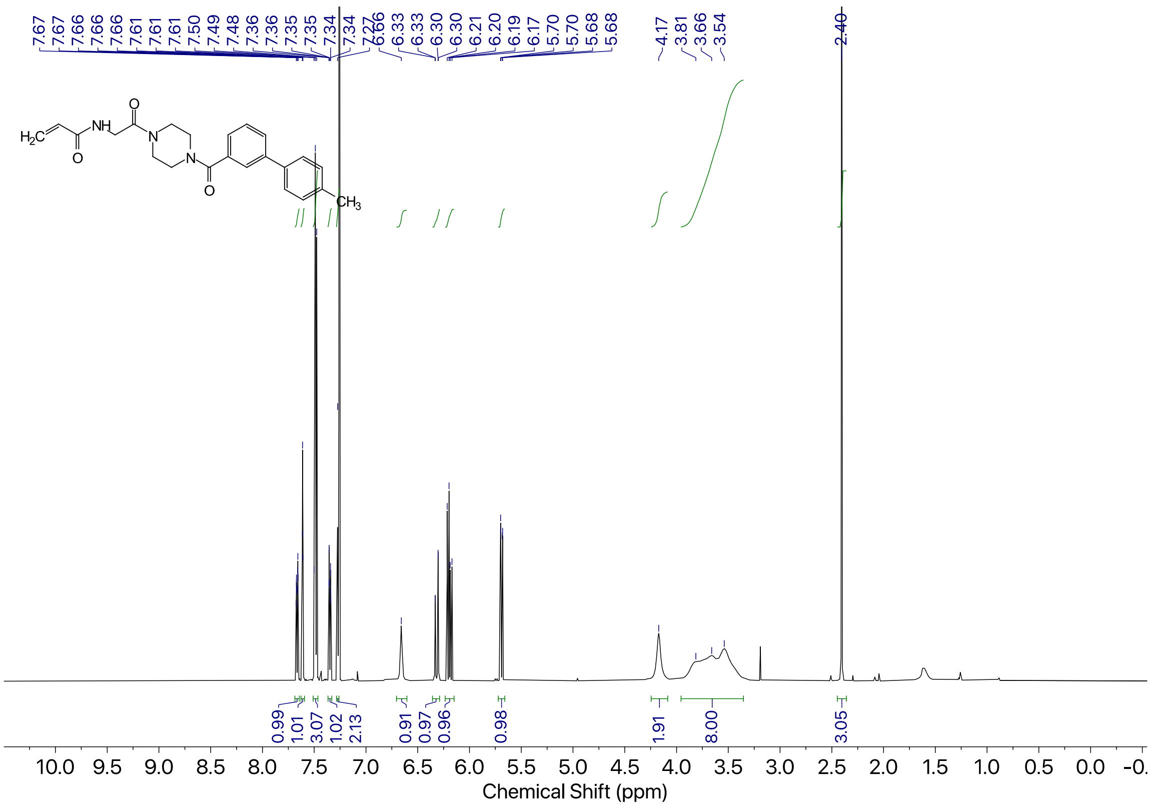

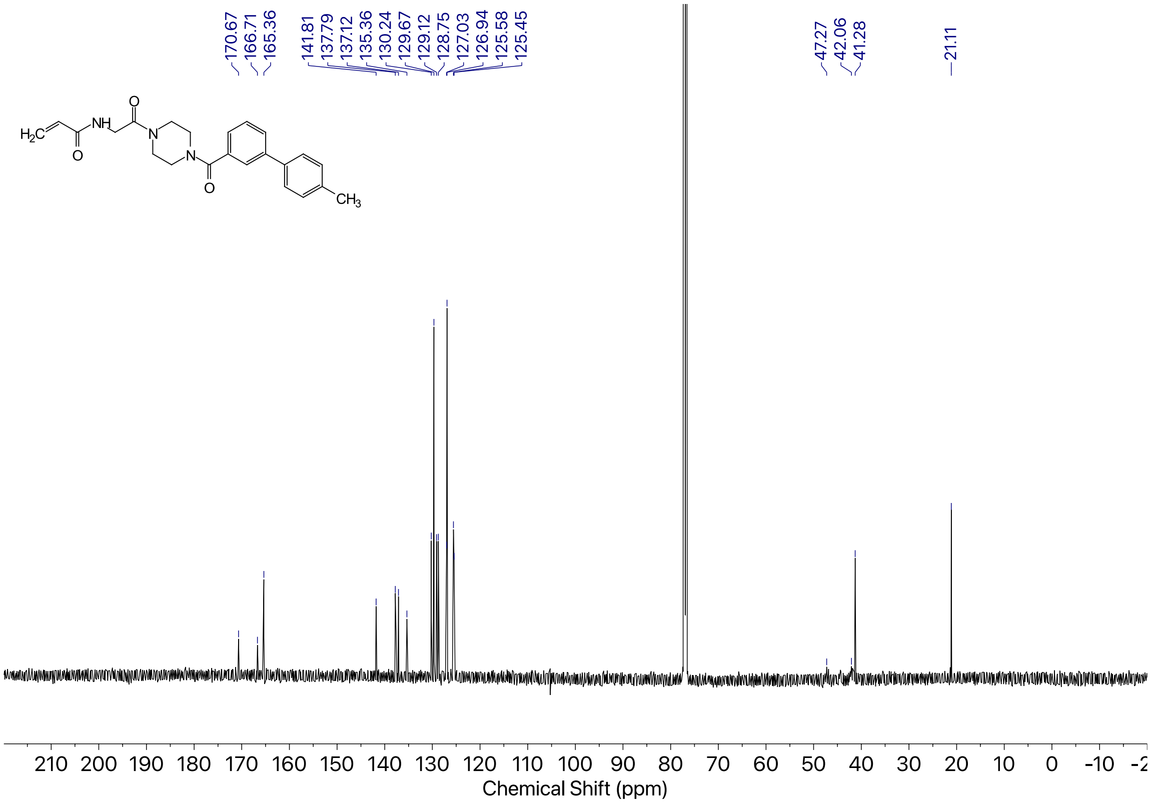

Compound 21g

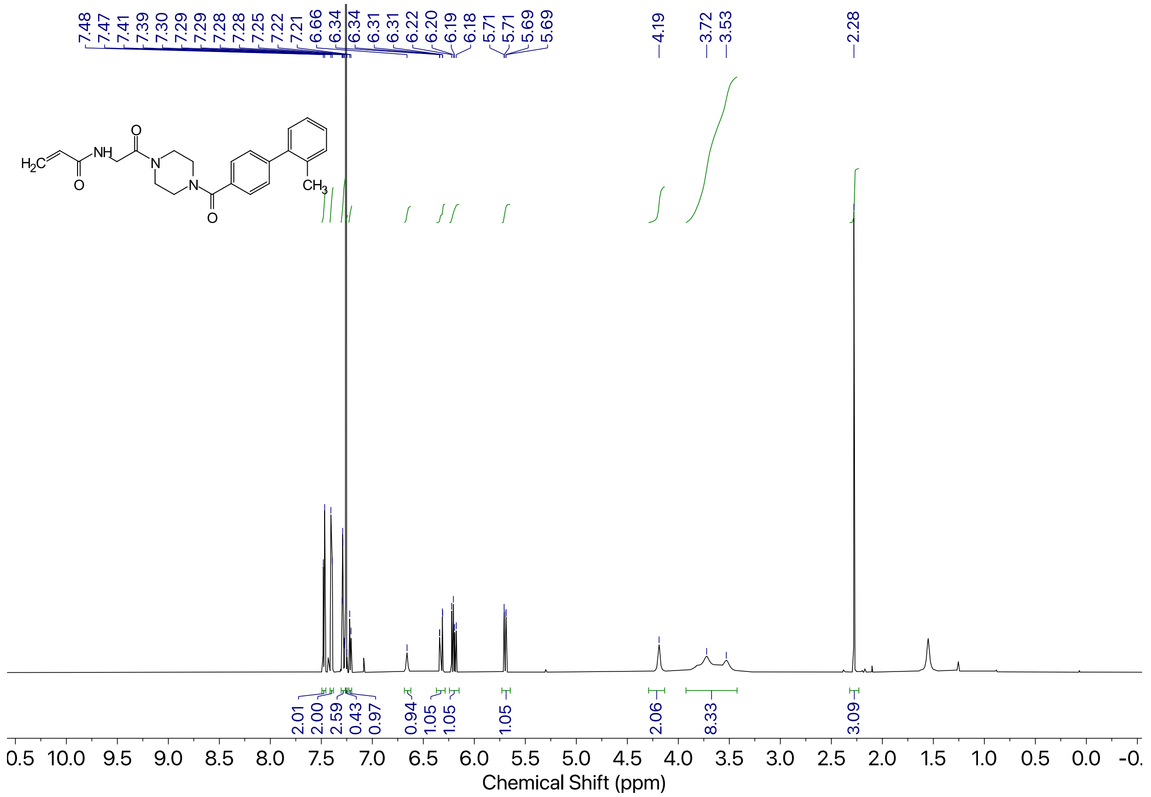

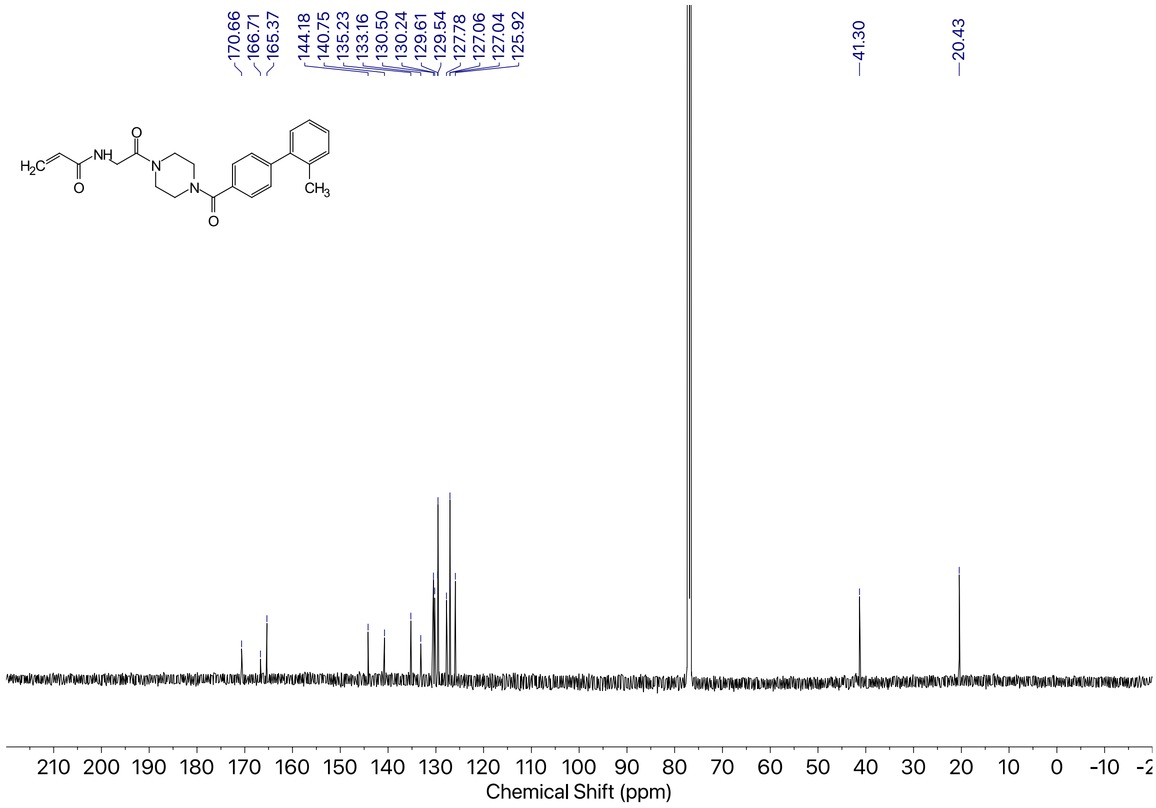

Compound 21h

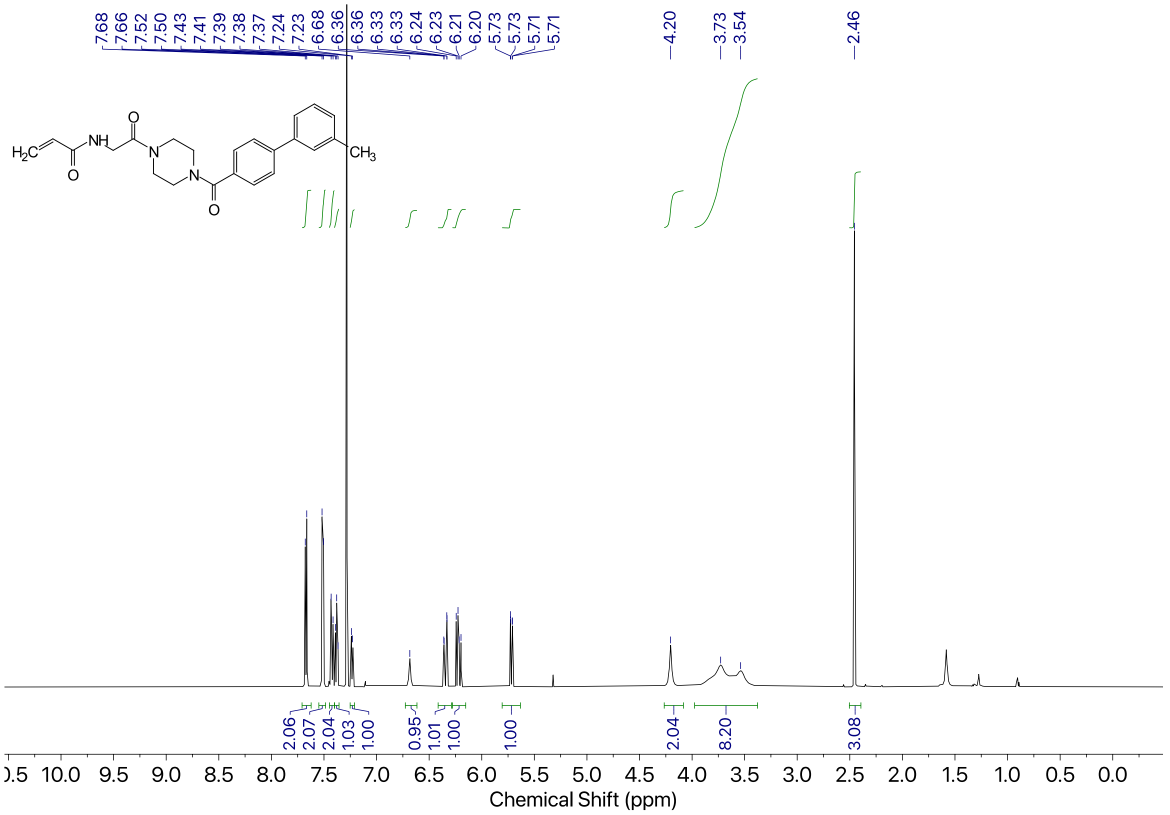

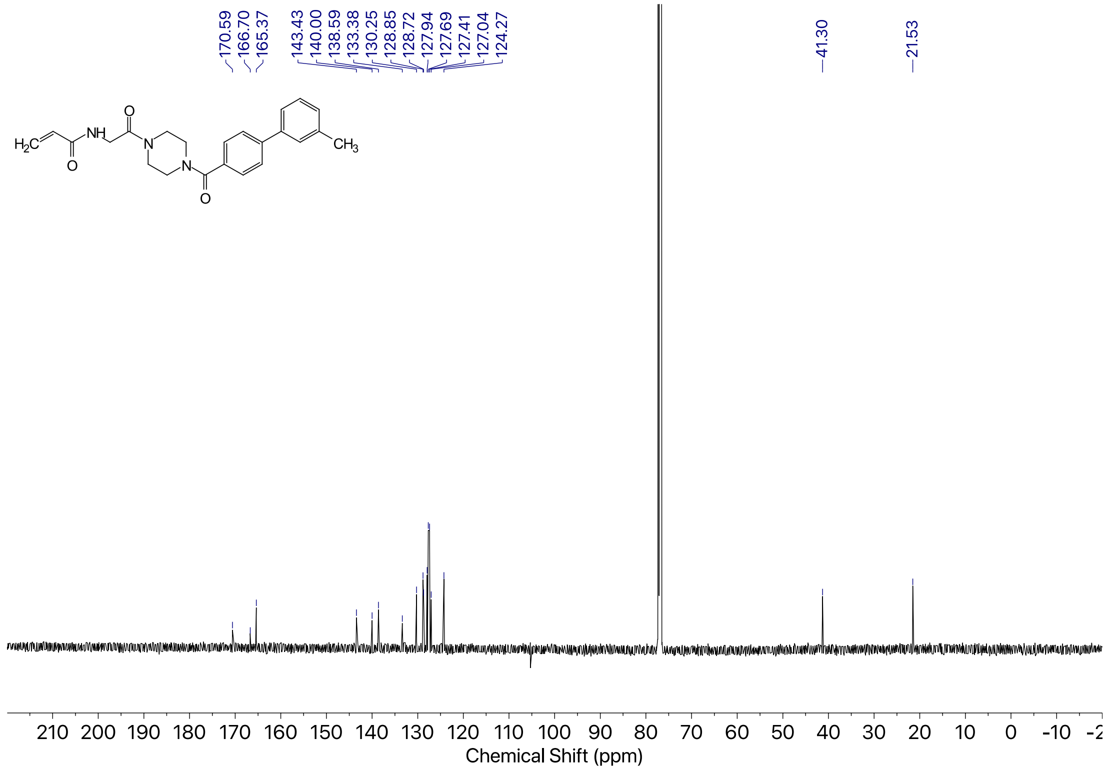

Compound 21i

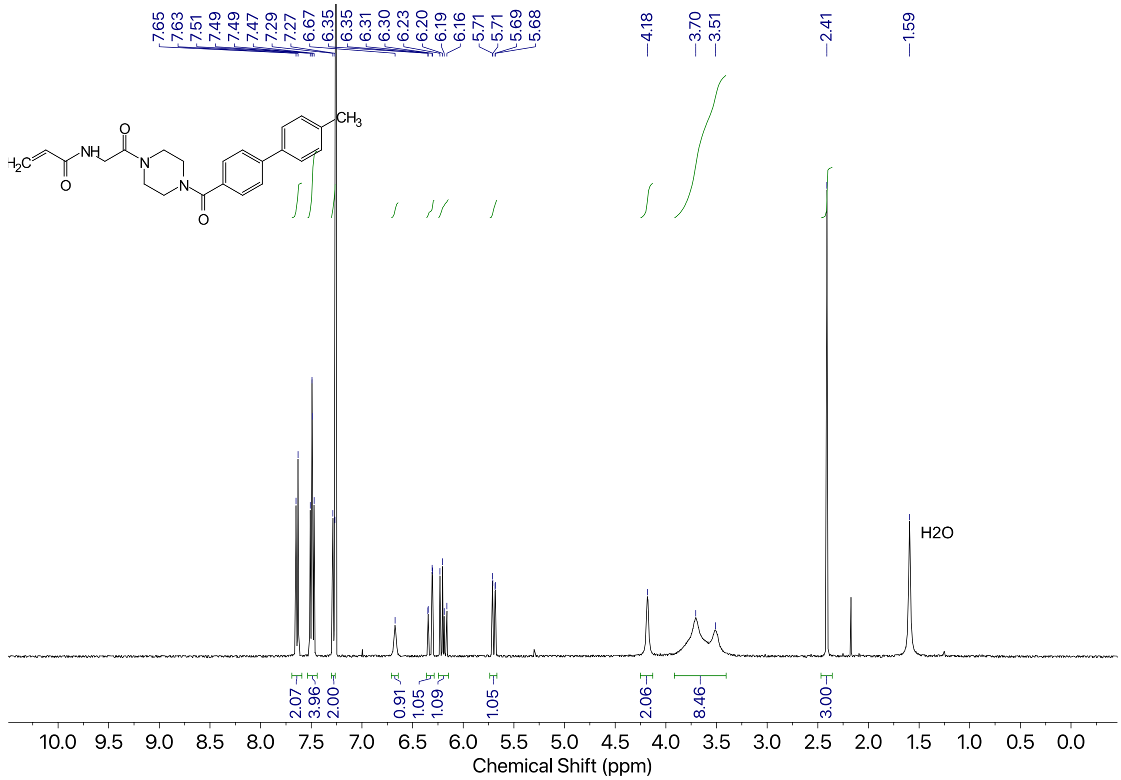

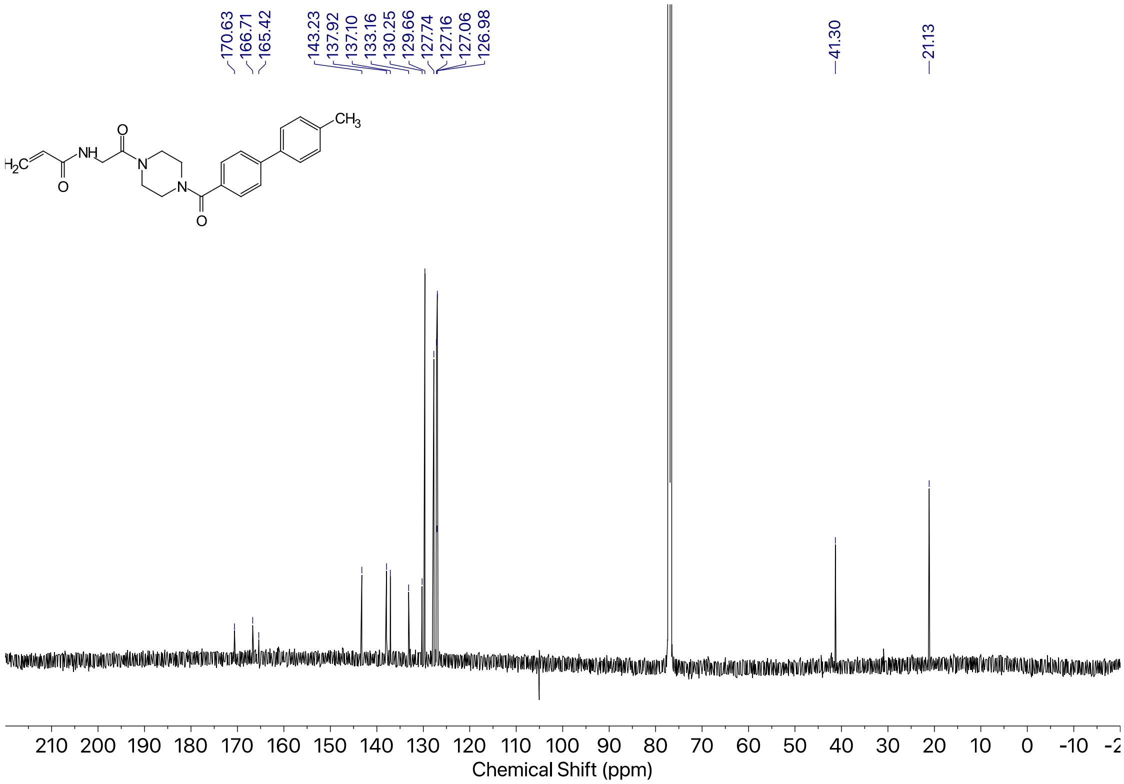

Compound 22a

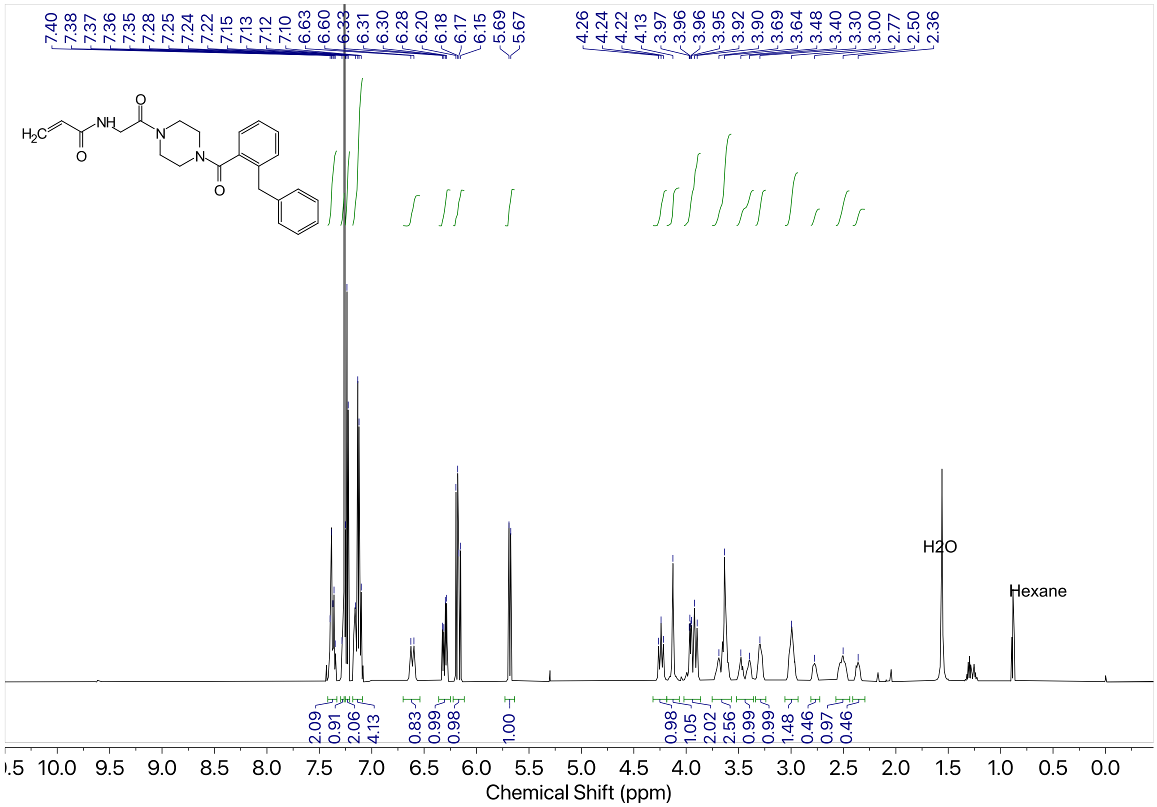

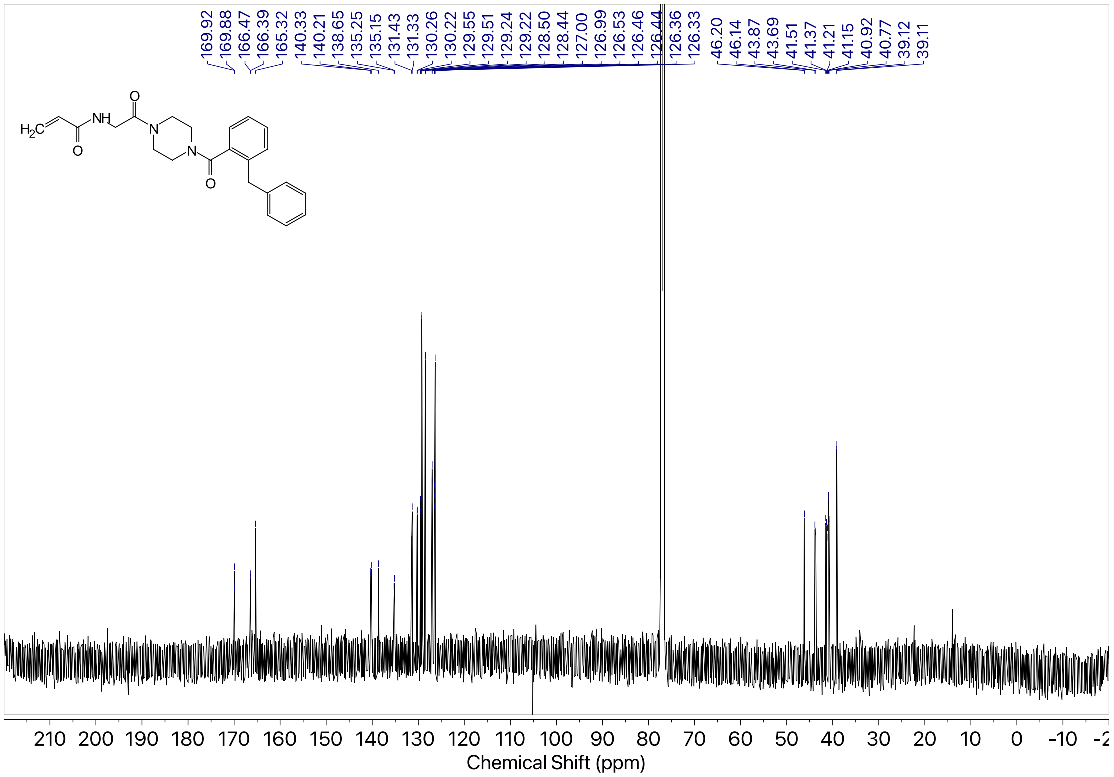

Compound 22b

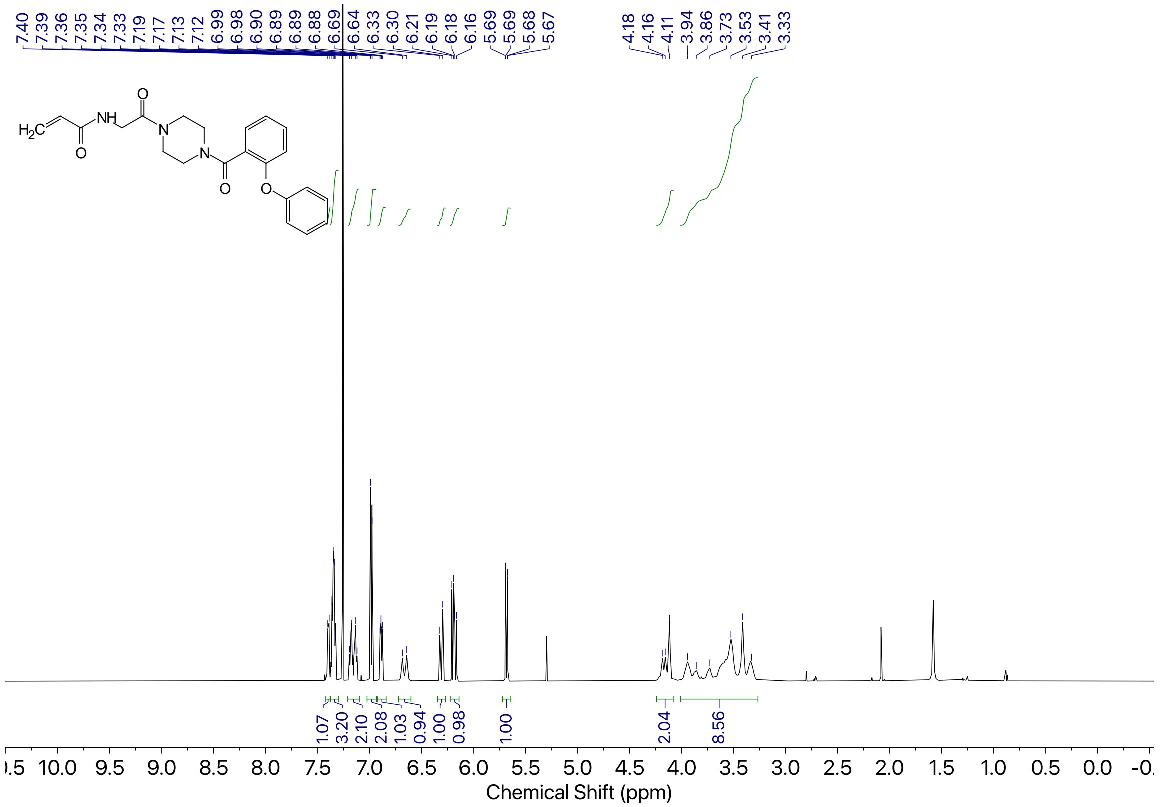

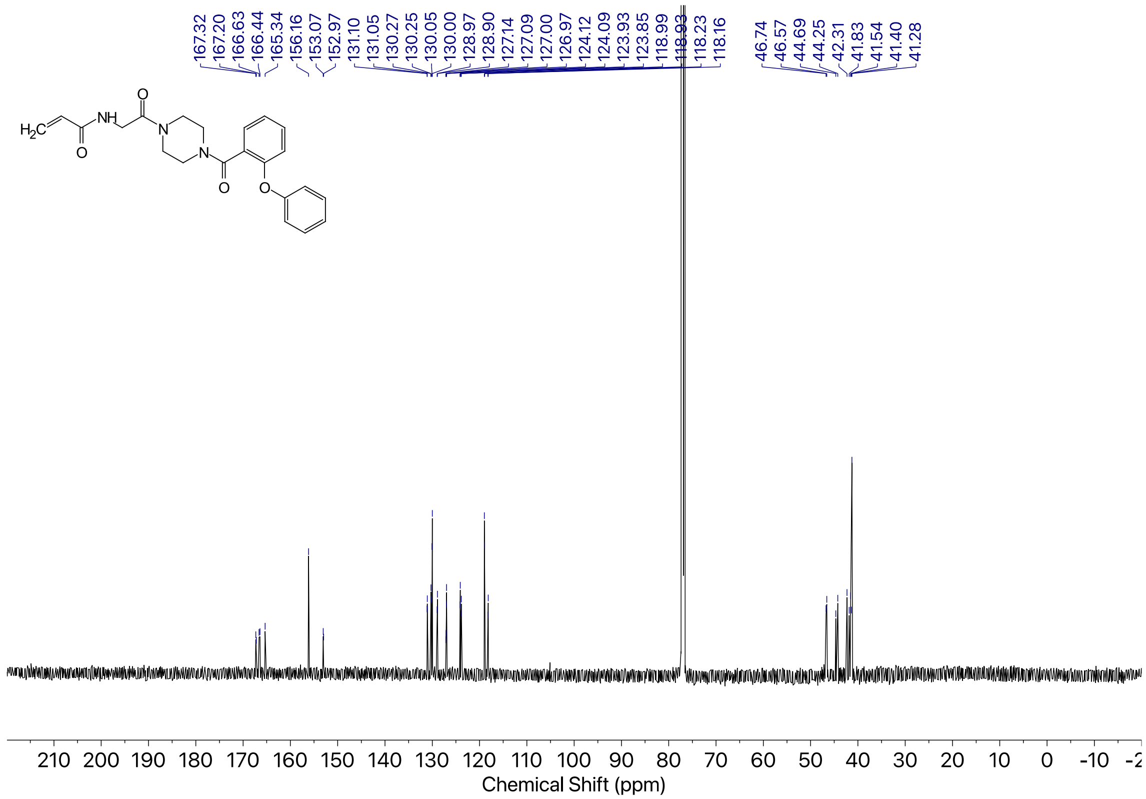

Compound 22c

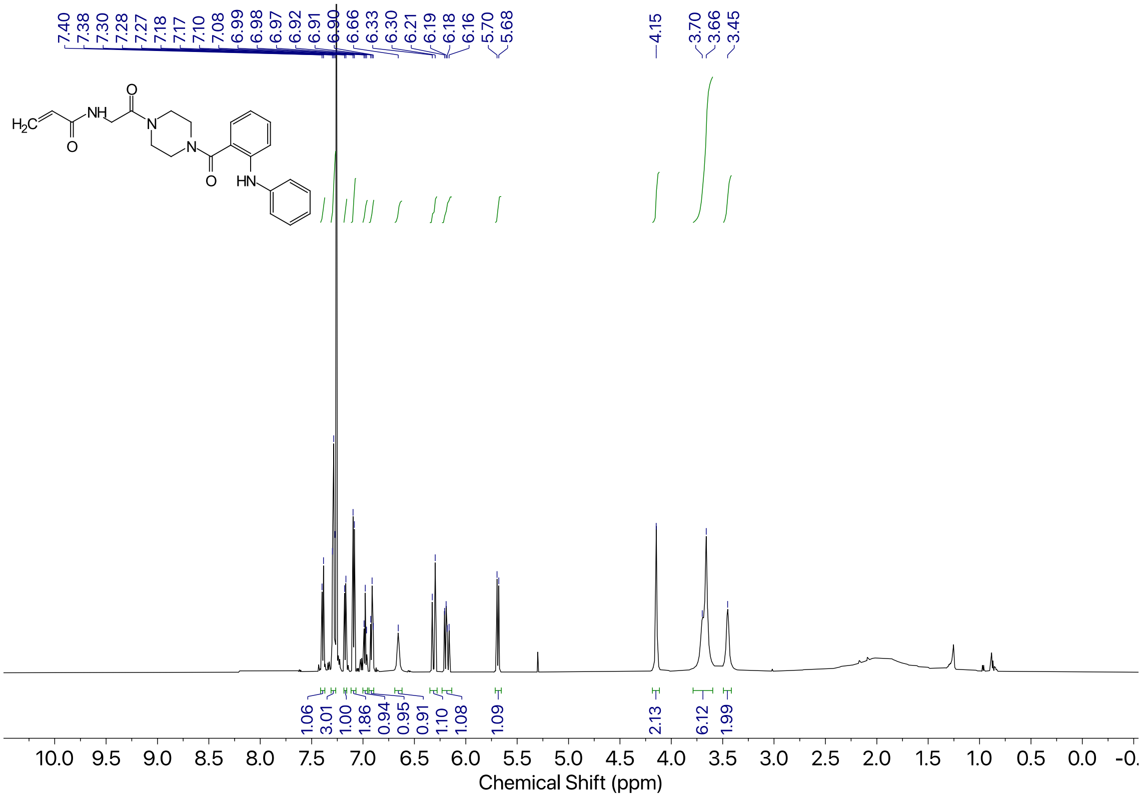

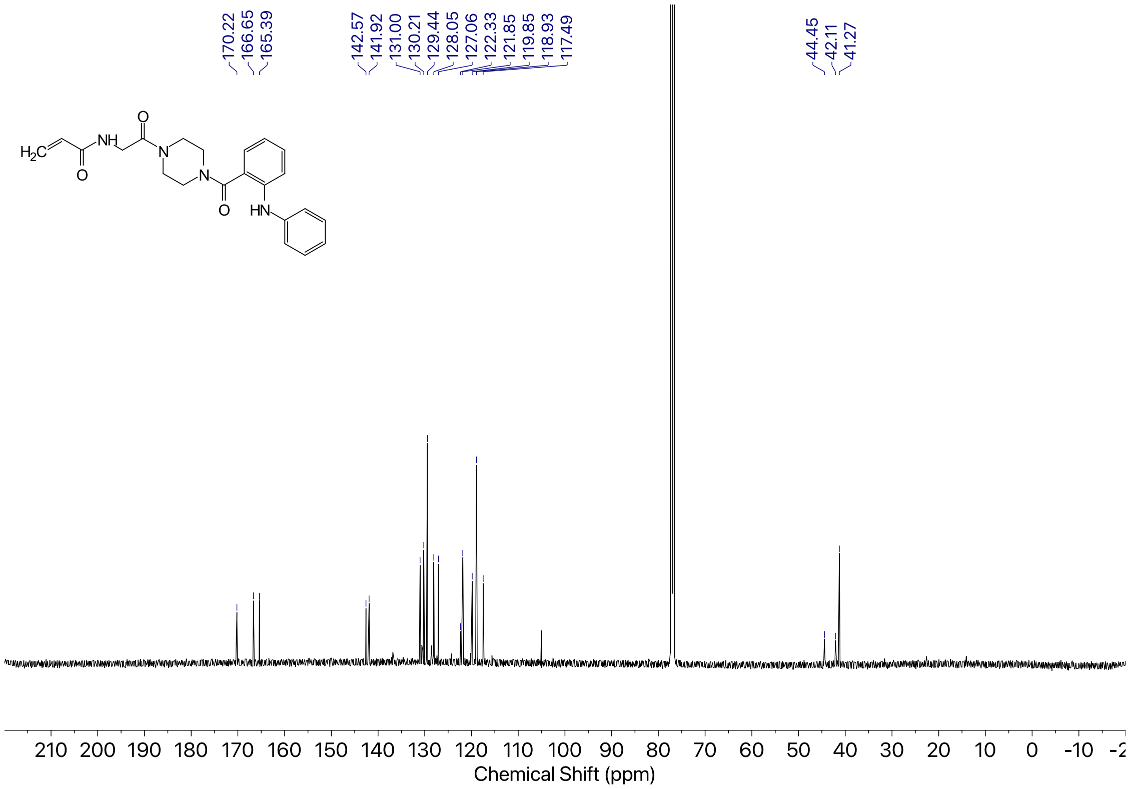

Compound 22d

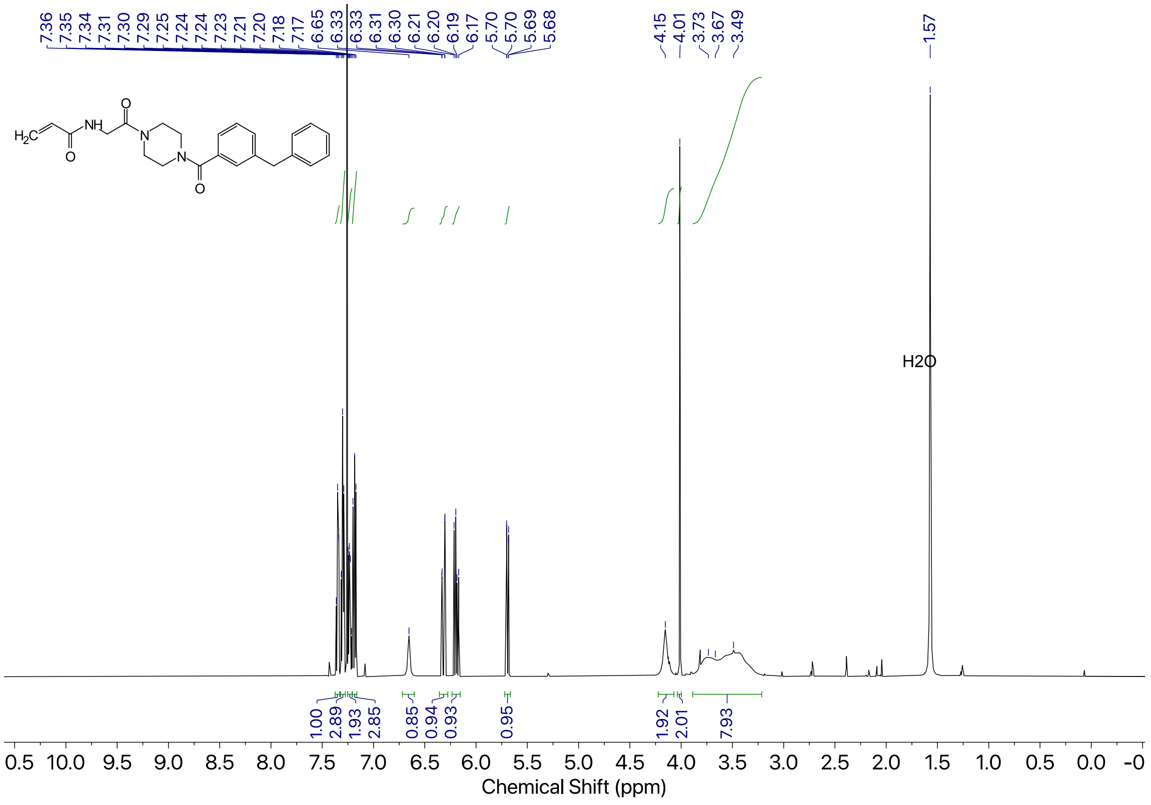

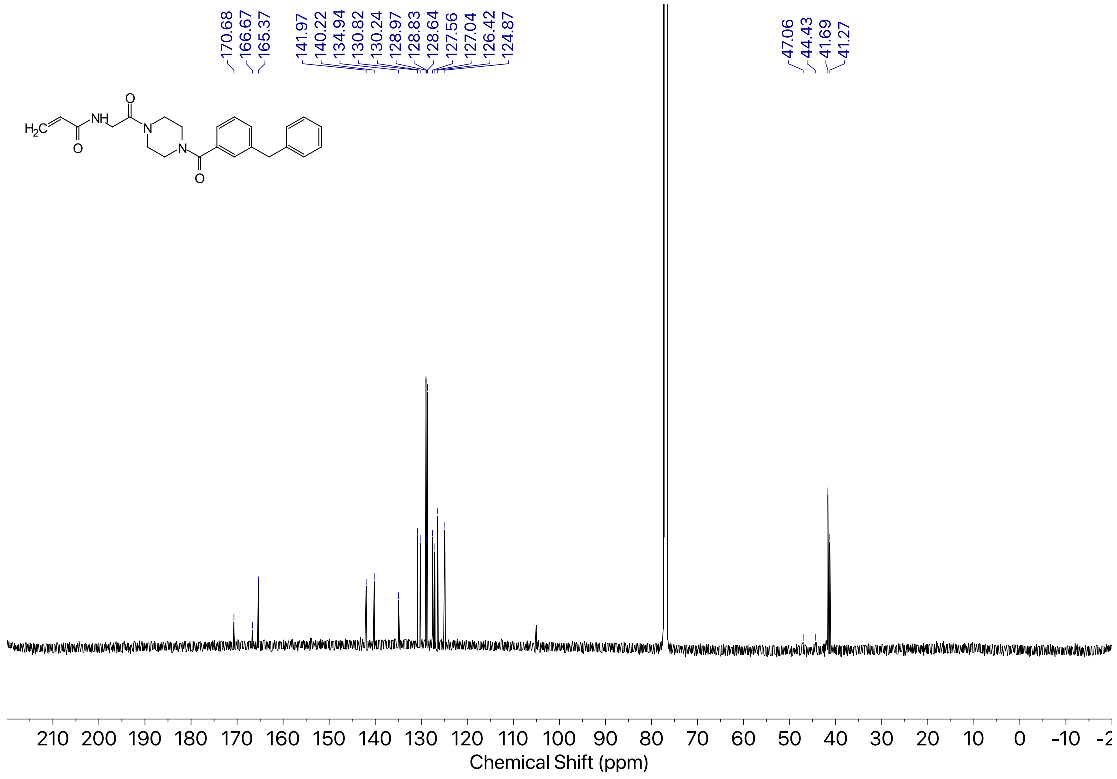

Compound 22e

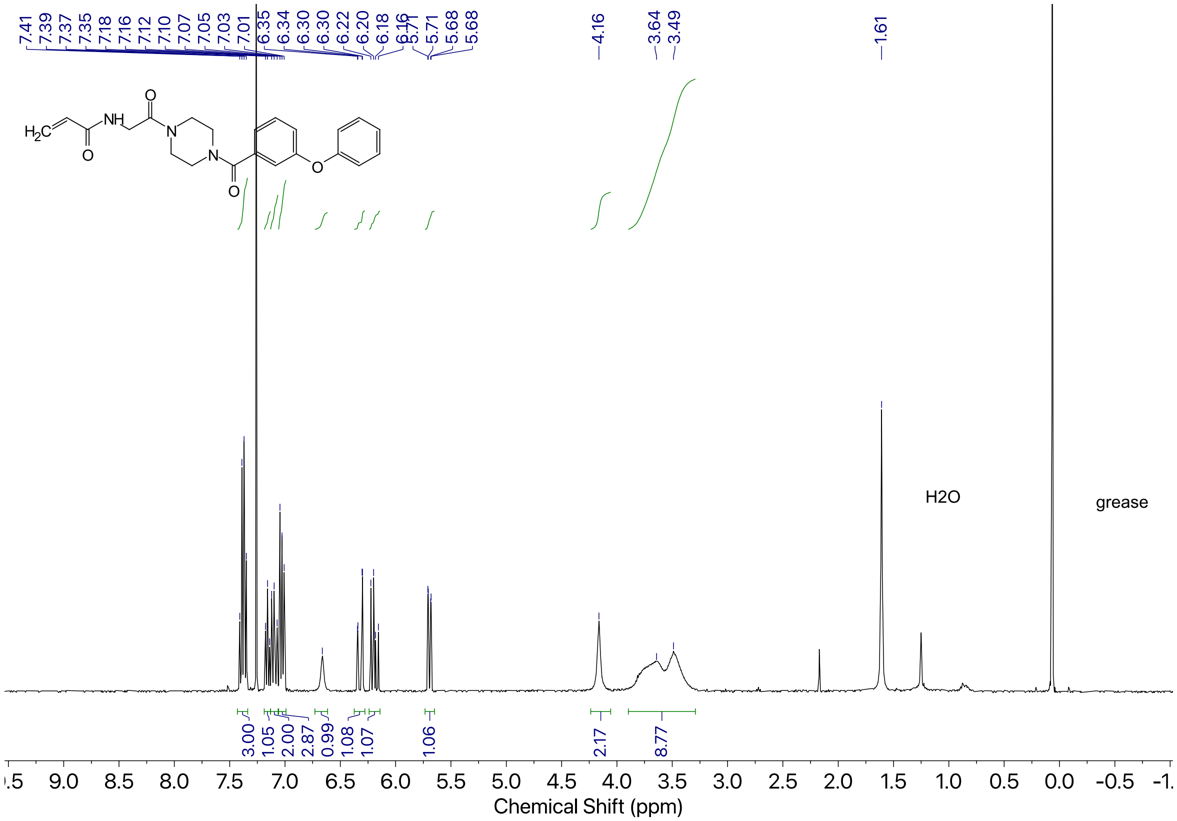

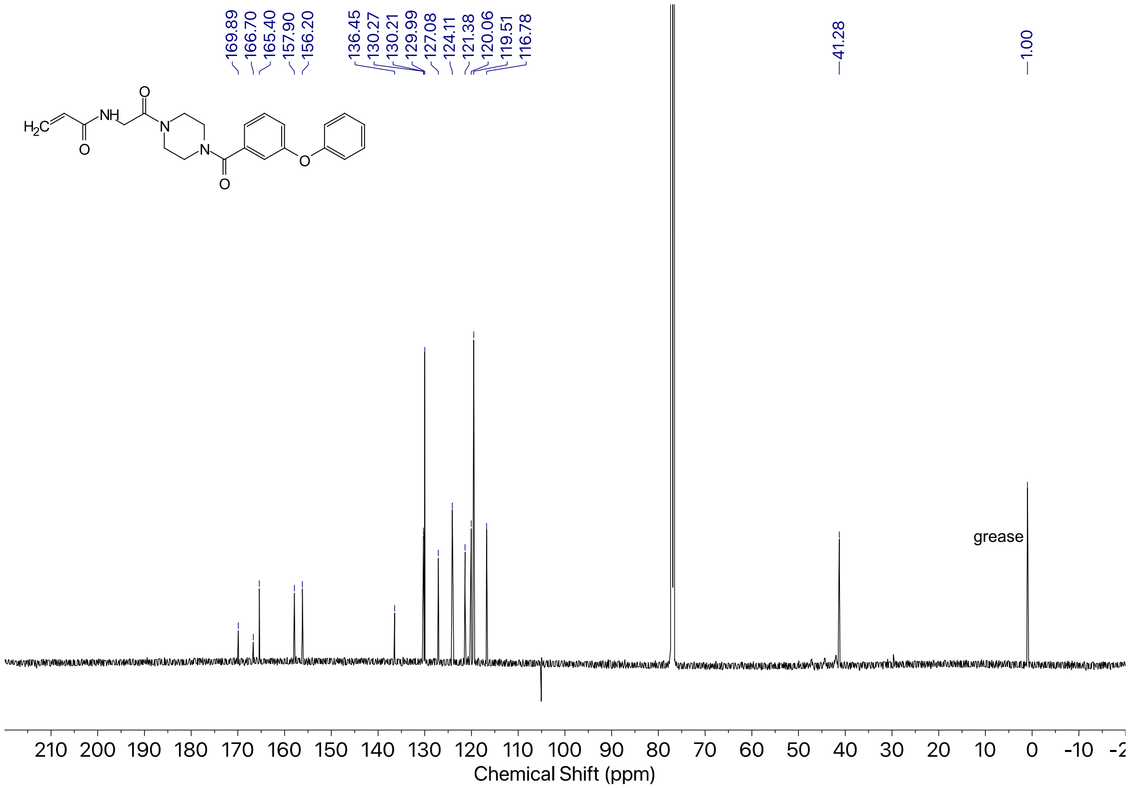

Compound 22f

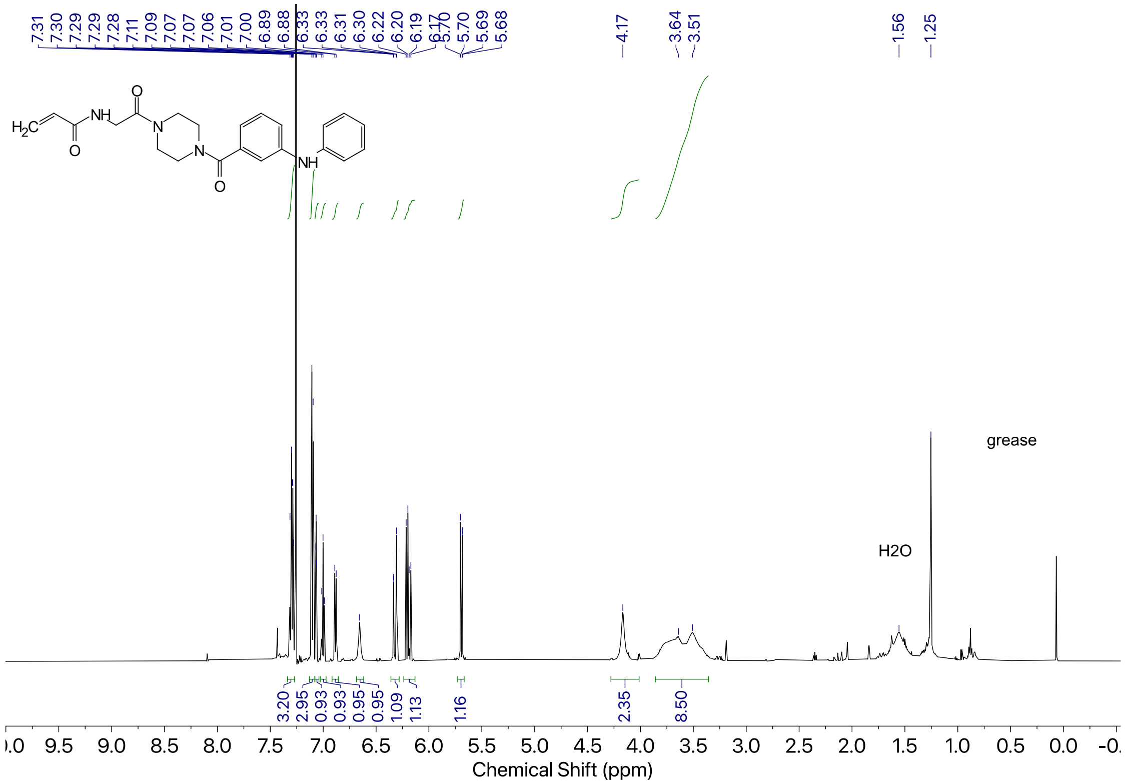

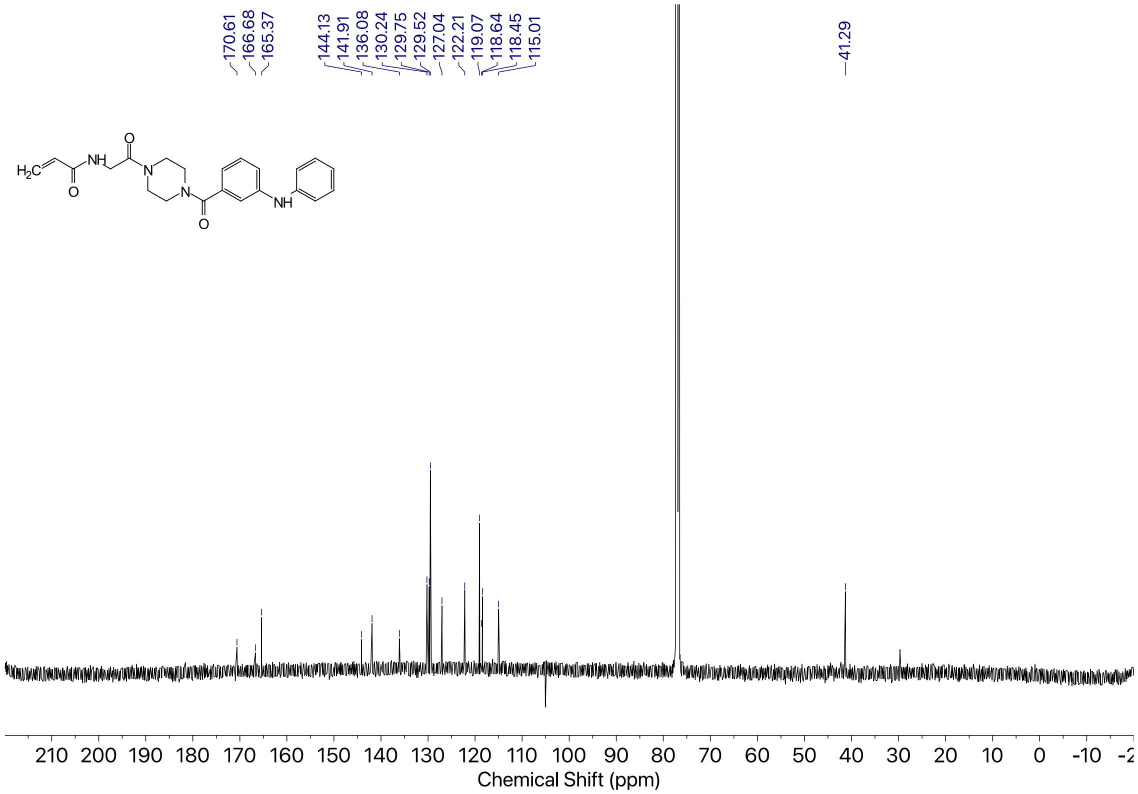

Compound 22g

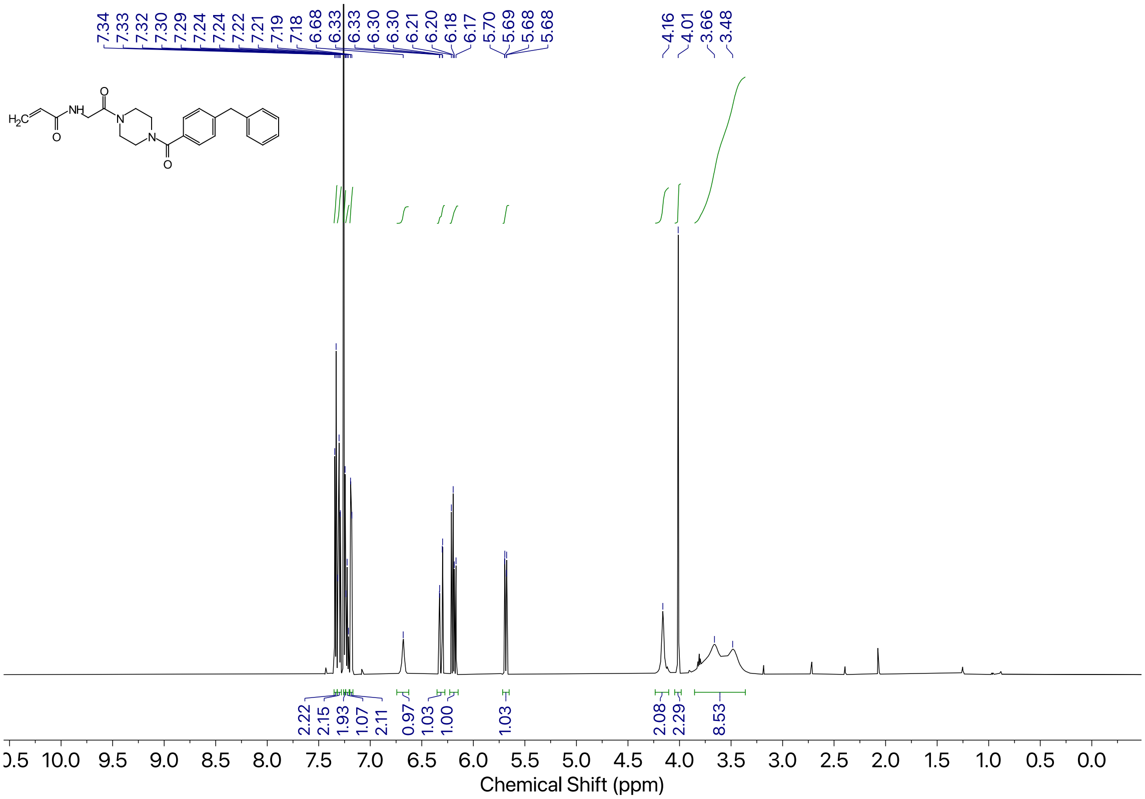

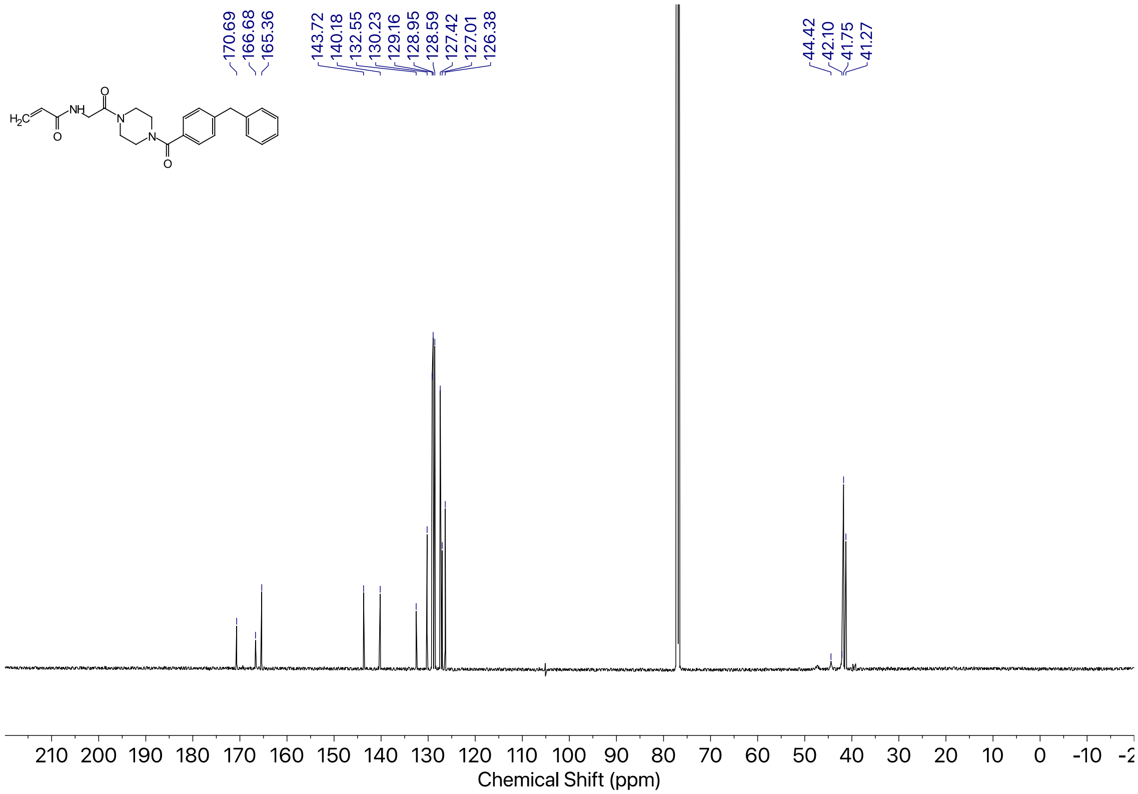

Compound 22h

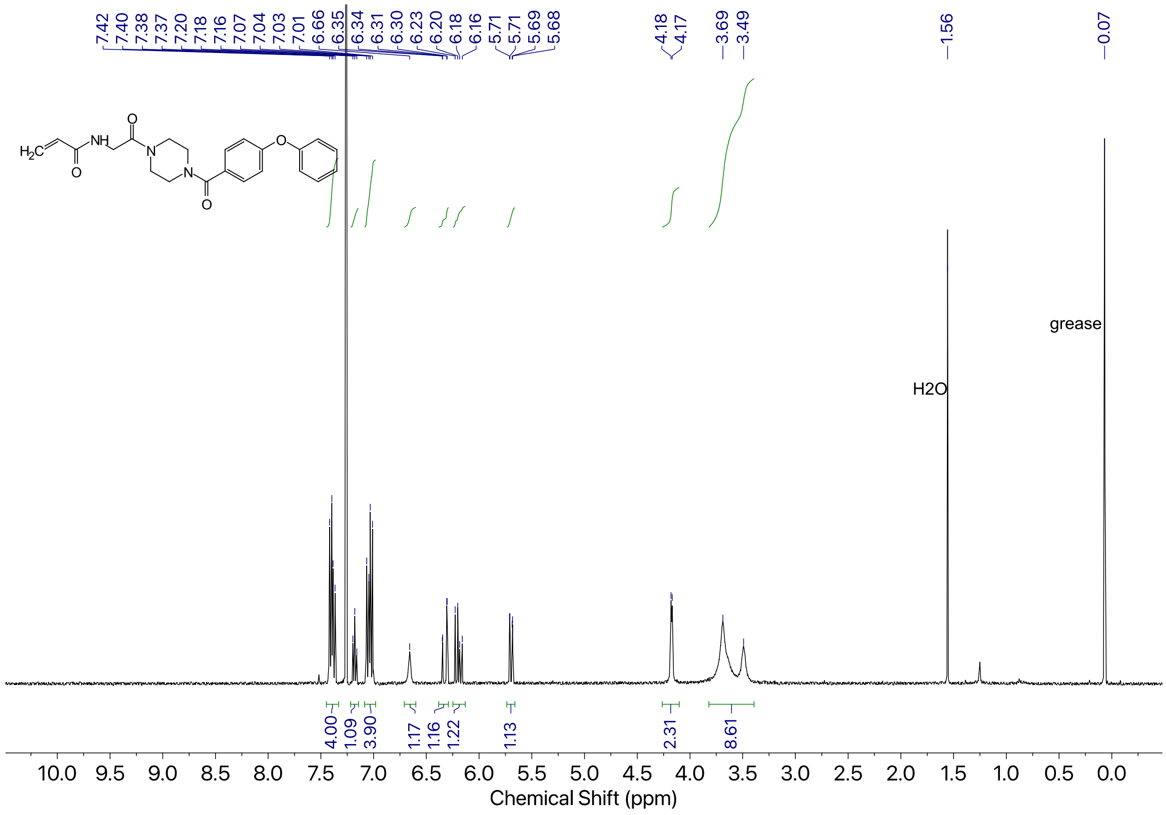

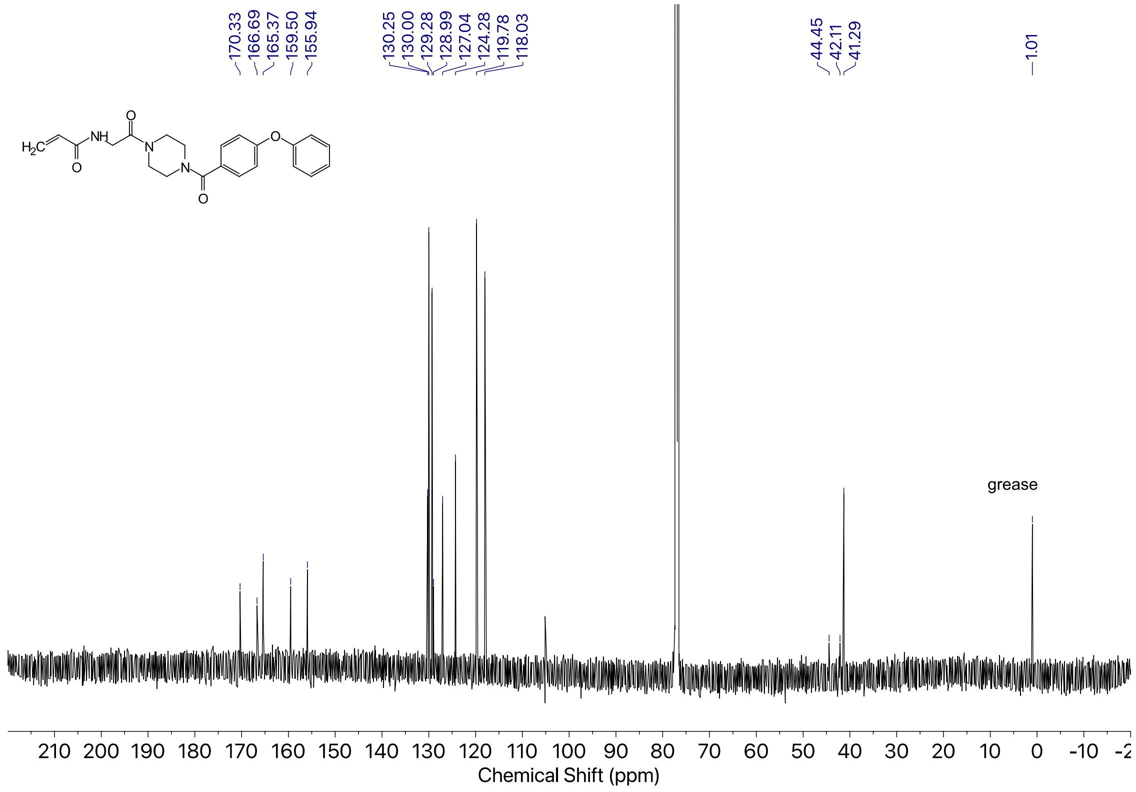

Compound 22i

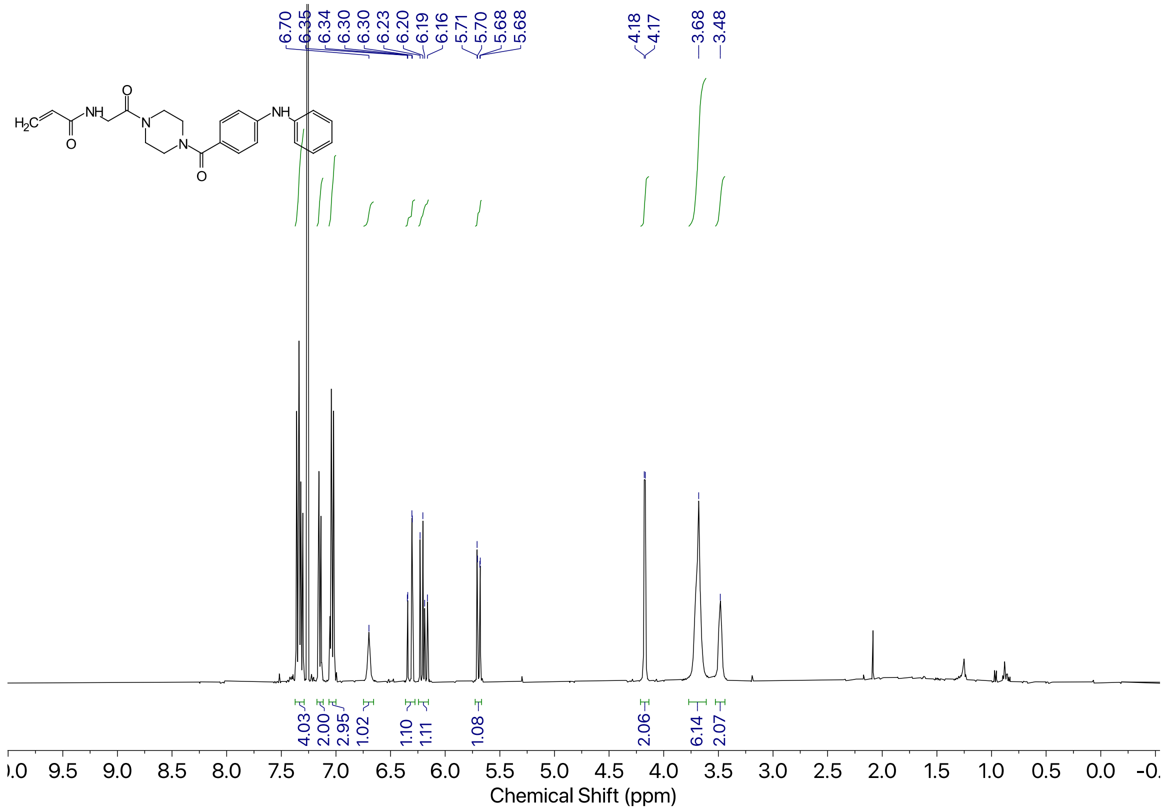

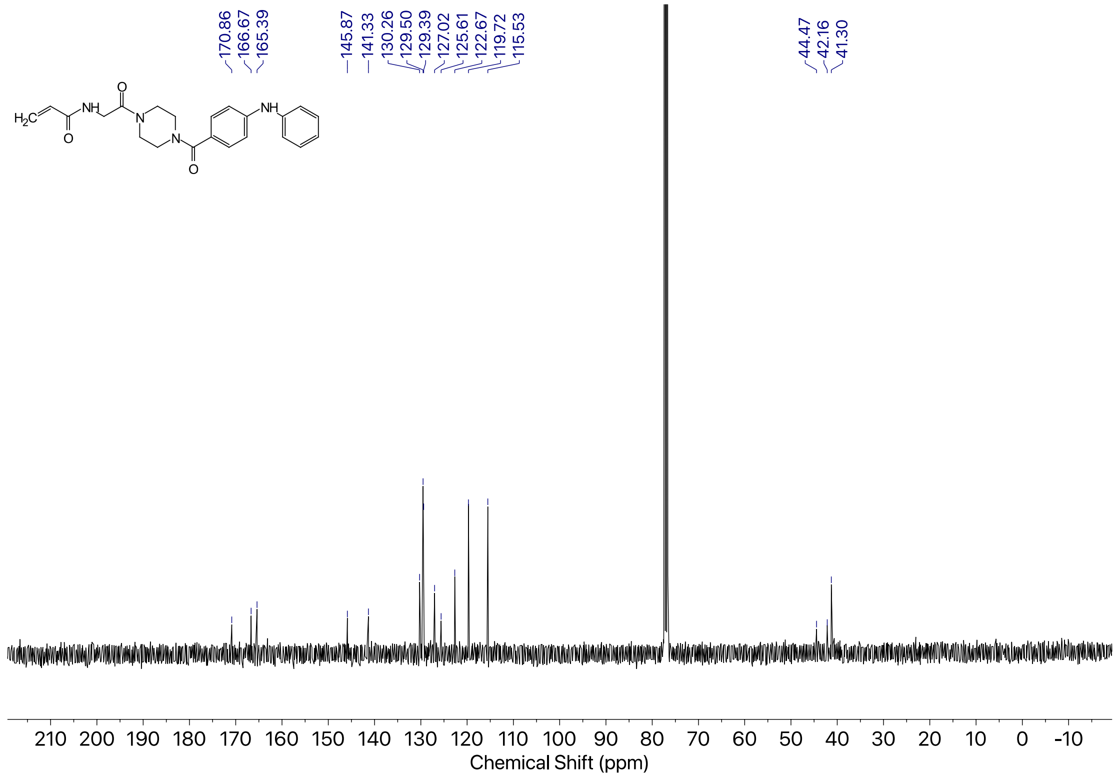

Compound 23a

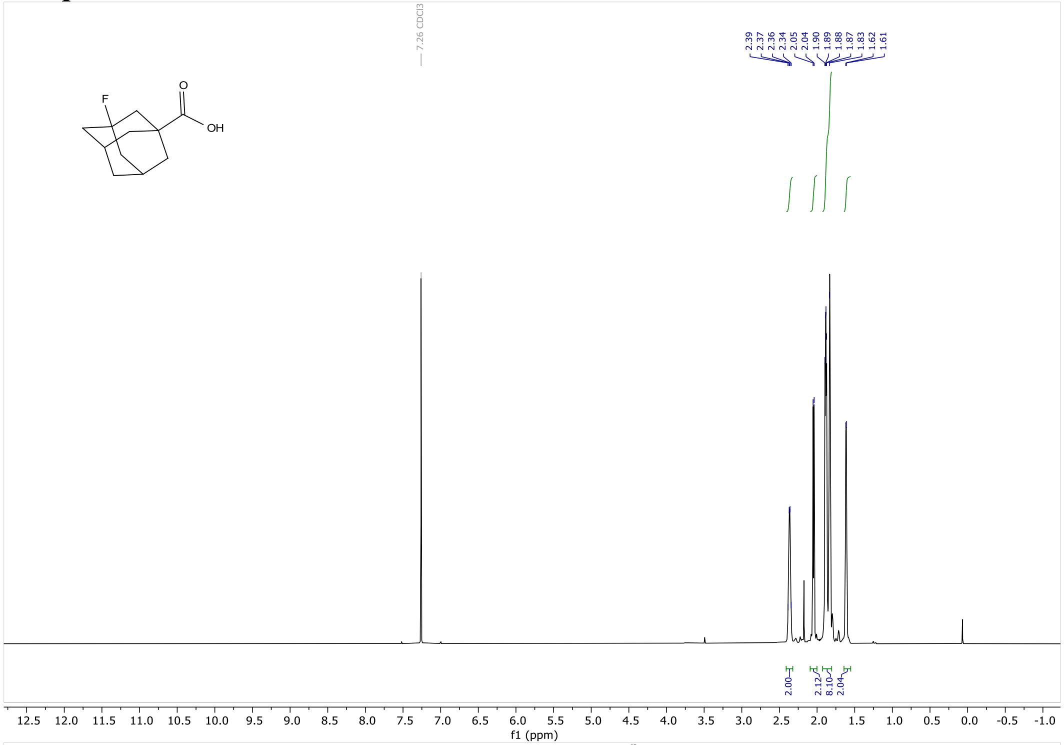

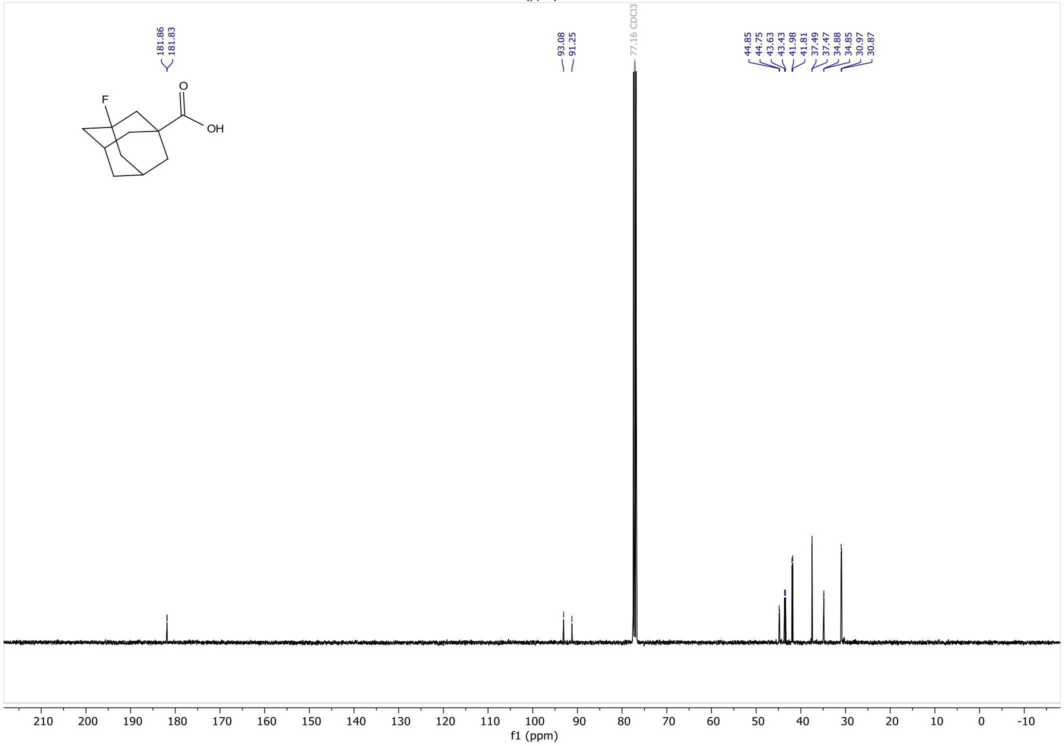

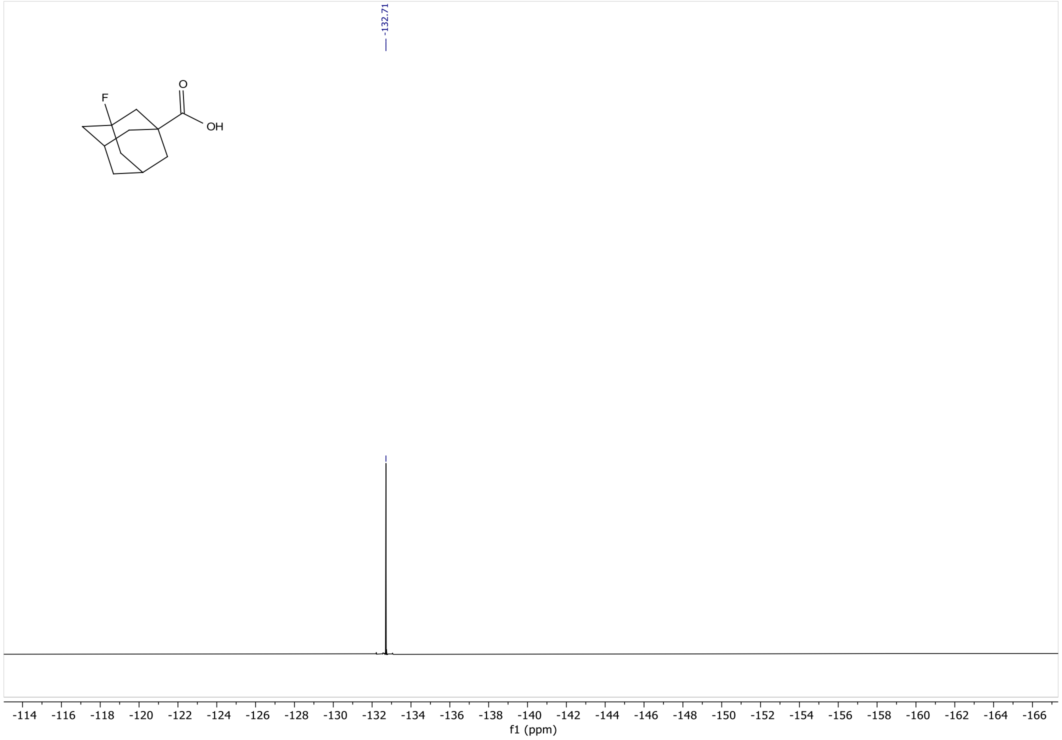

-132.71
F
O
OH
-114 -116 -118 -120 -122 -124 -126 -128 -130 -132 -134 -136 -138 -140 -142 -144 -146 -148 -150 -152 -154 -156 -158 -160 -162 -164 -166
f1 (ppm)

Compound 23b

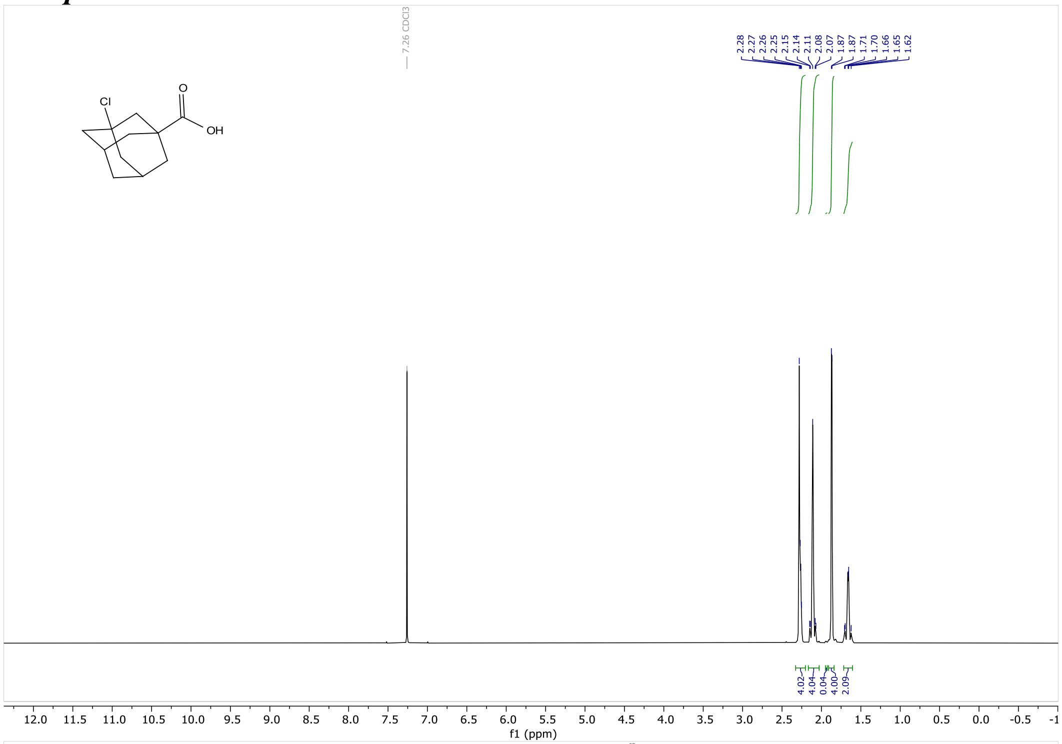

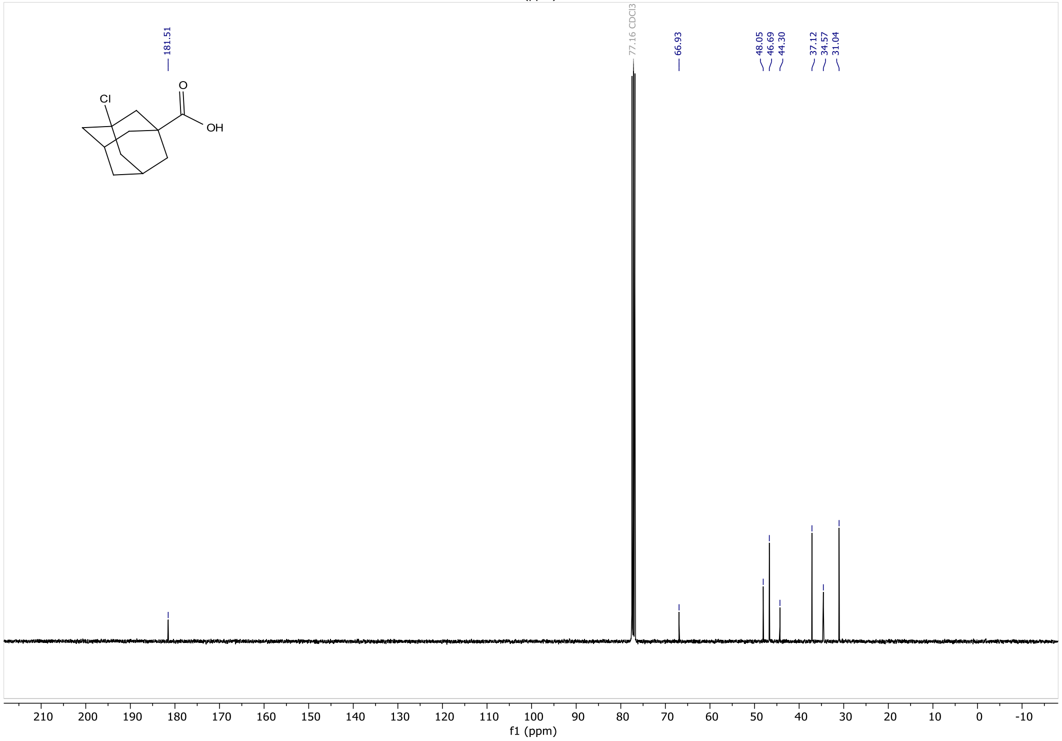

Compound 23c

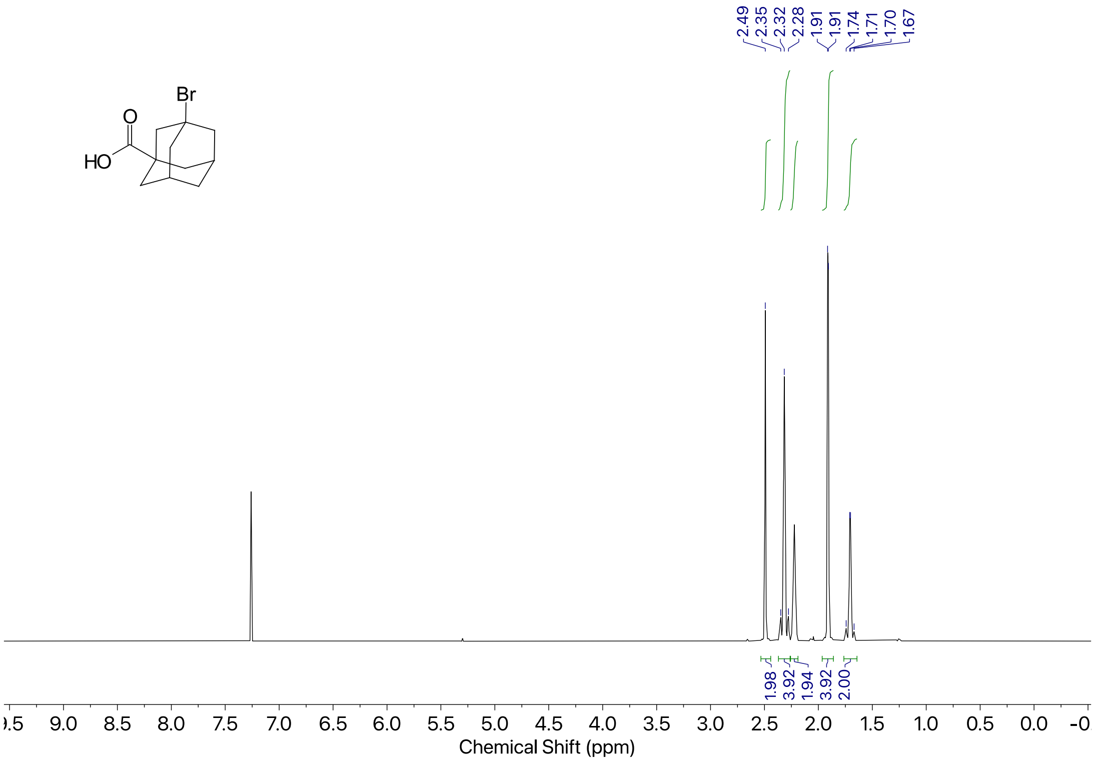

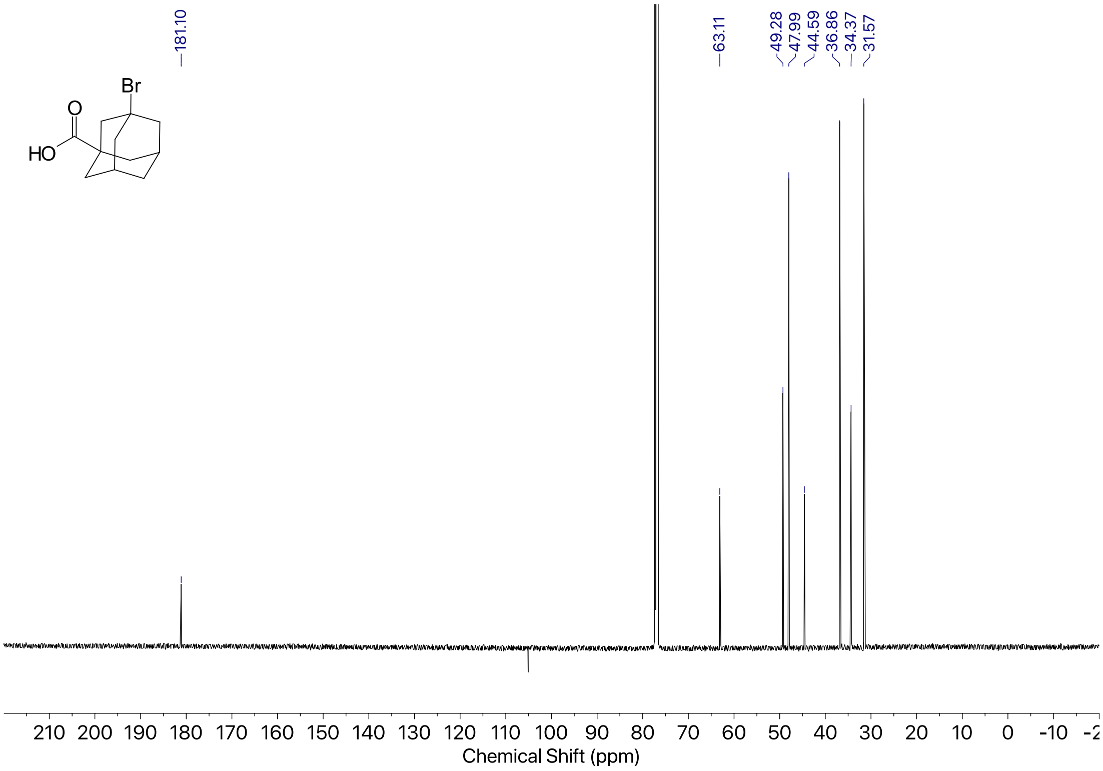

Compound 23d

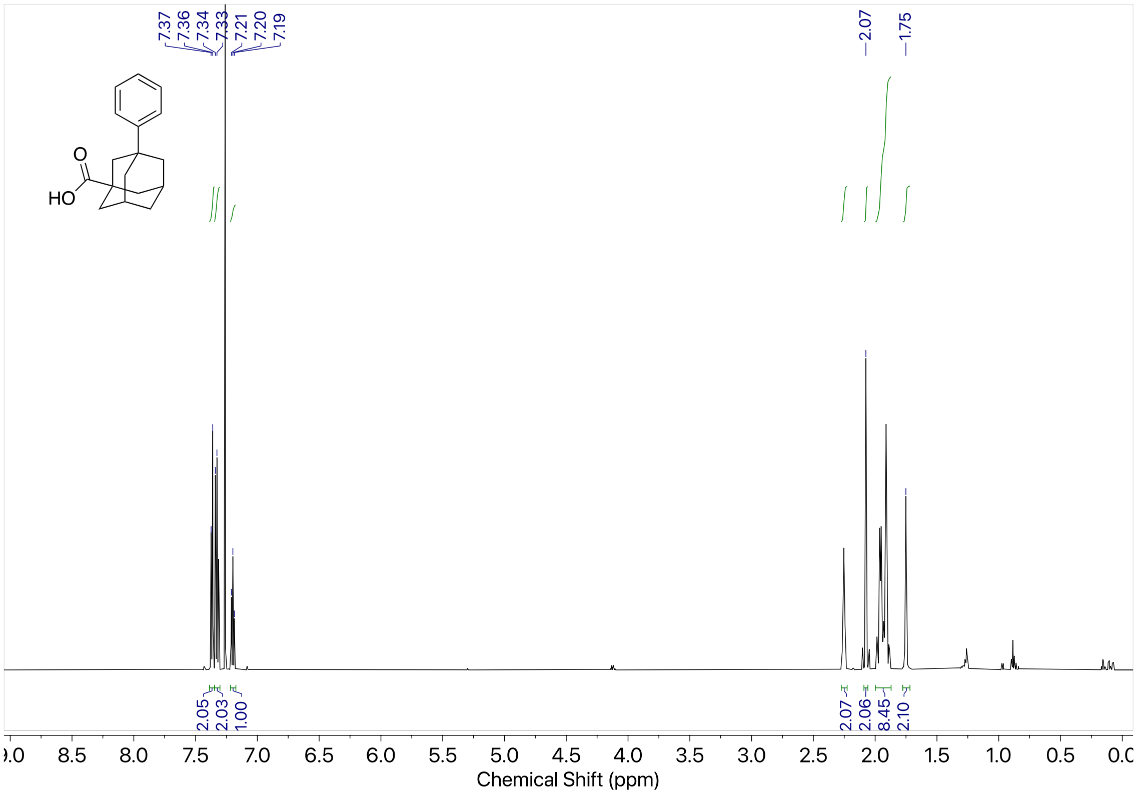

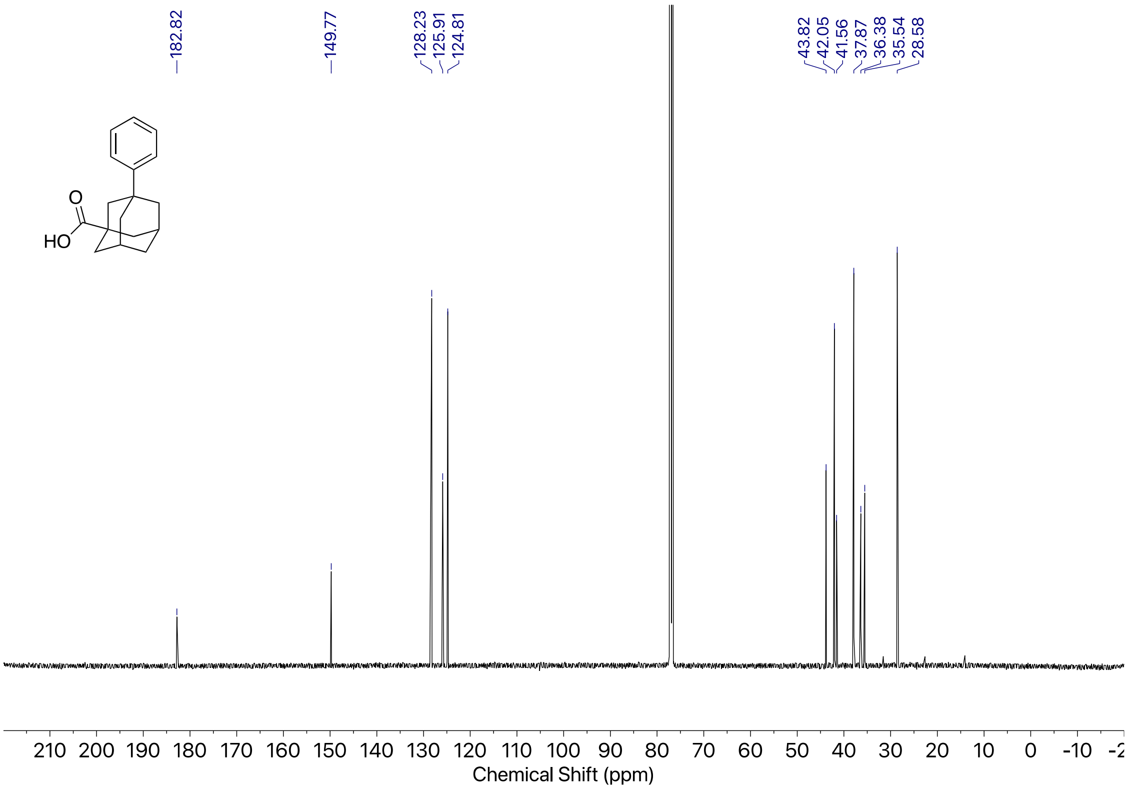

Compound 24a

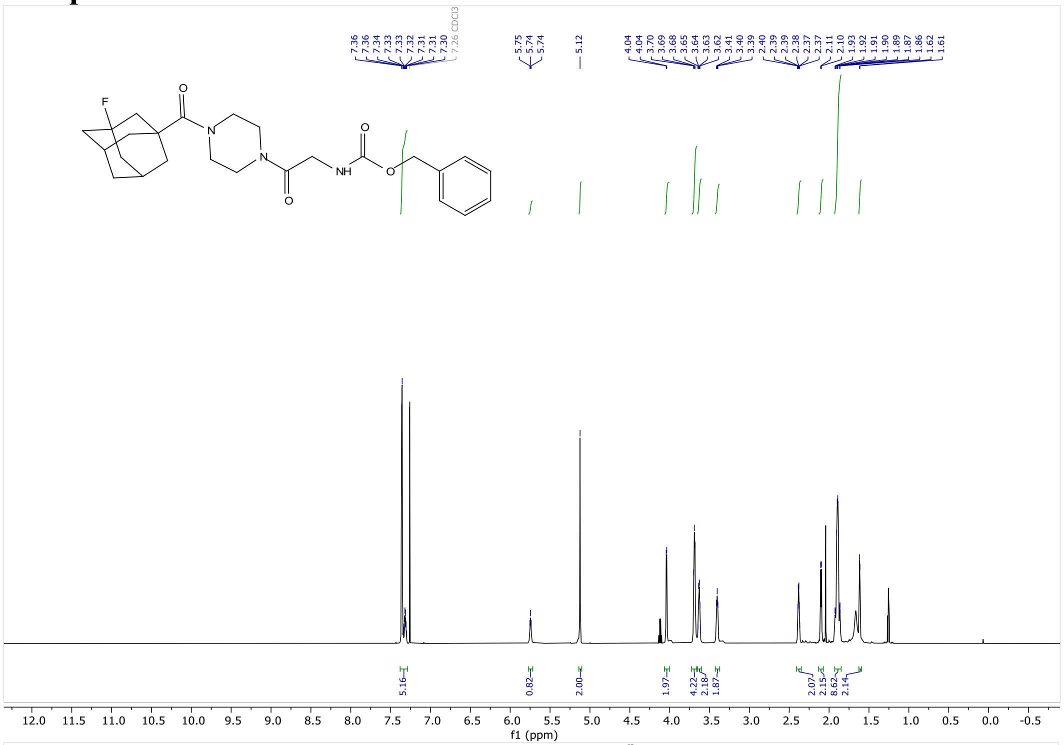

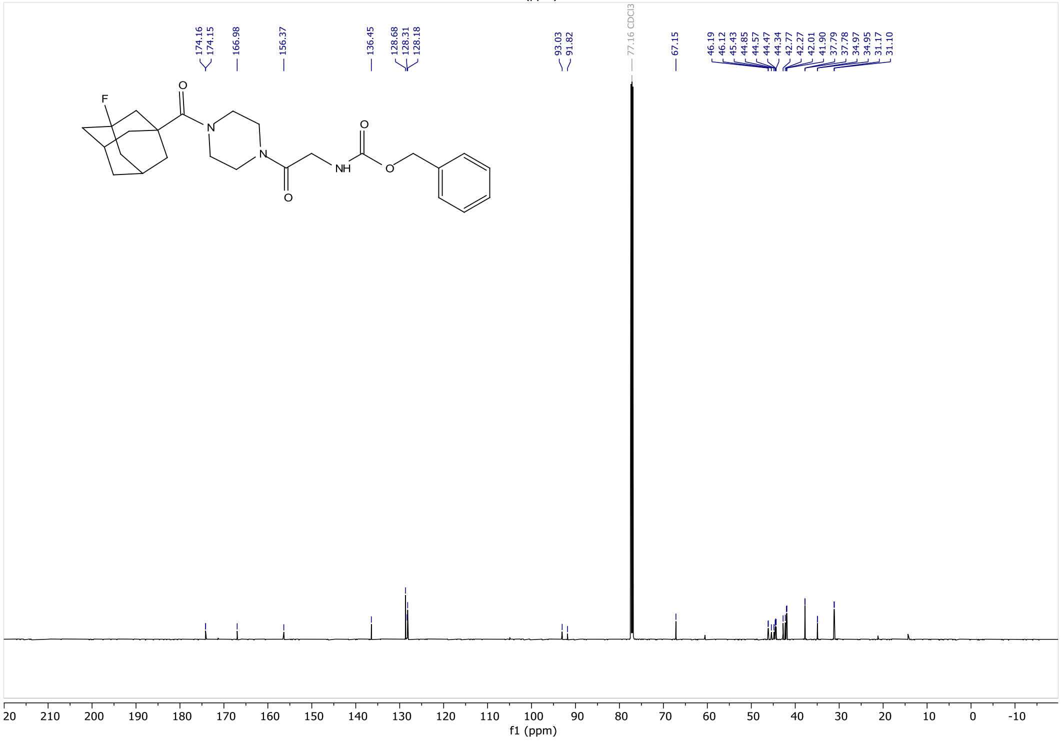

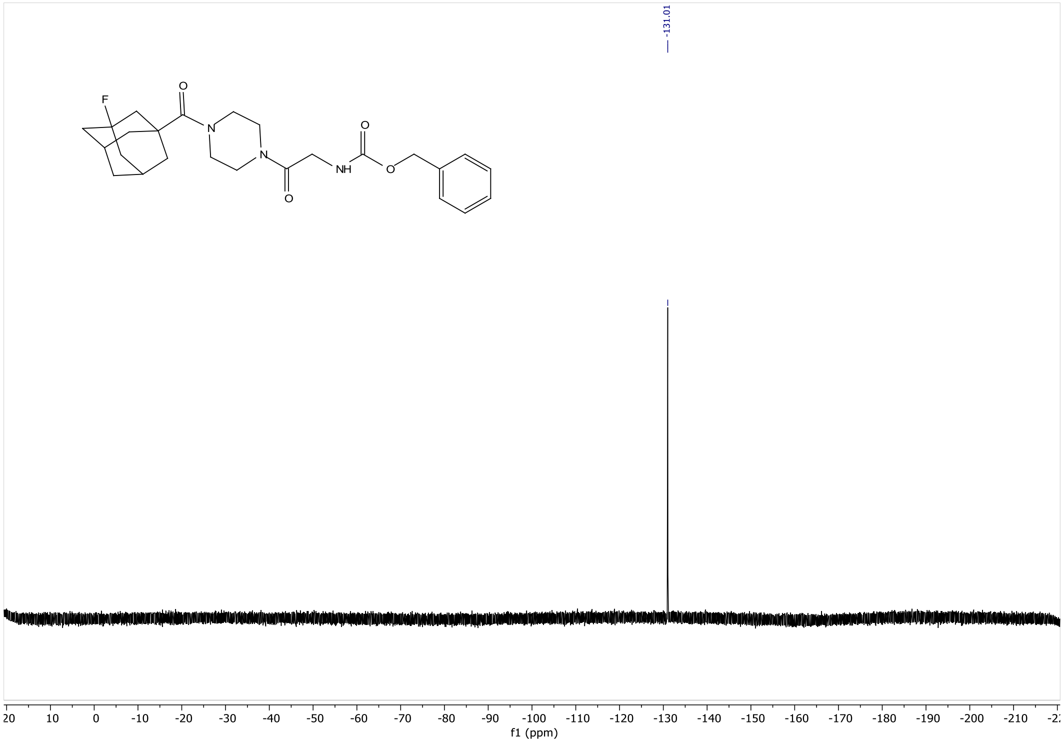
-131.01
F
O
N
N
O
NH
O
O
20
10
0
-10
-20
-30
-40
-50
-60
-70
-80
-90
-100
-110
-120
-130
-140
-150
-160
-170
-180
-190
-200
-210
f1 (ppm)

Compound 24b

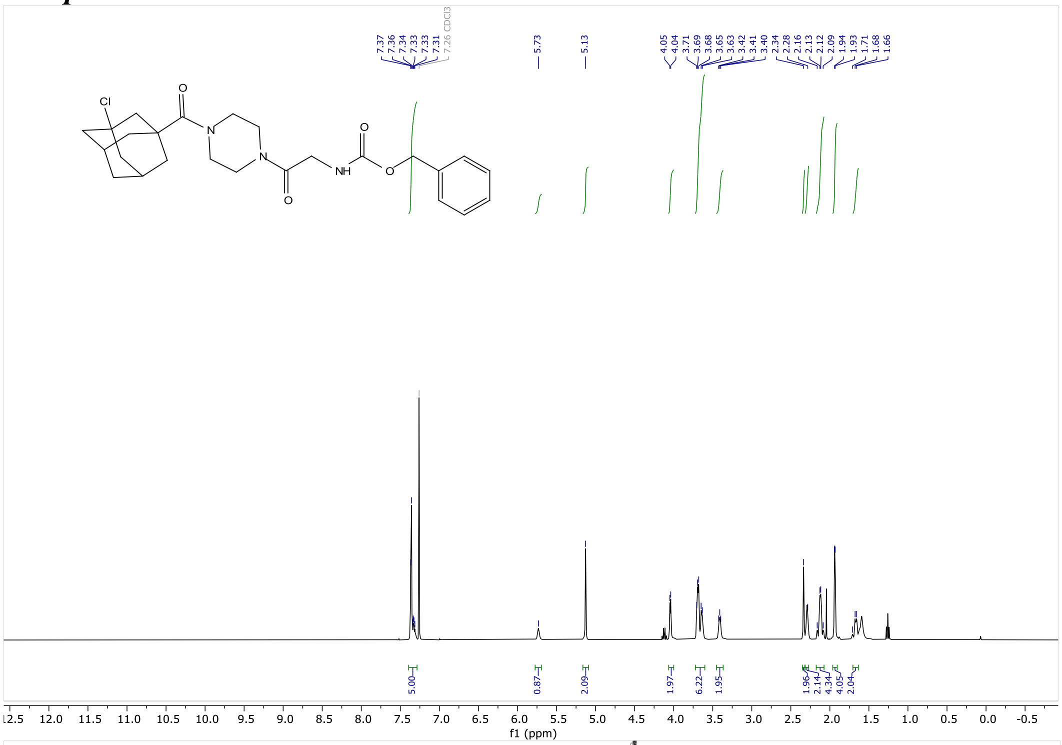

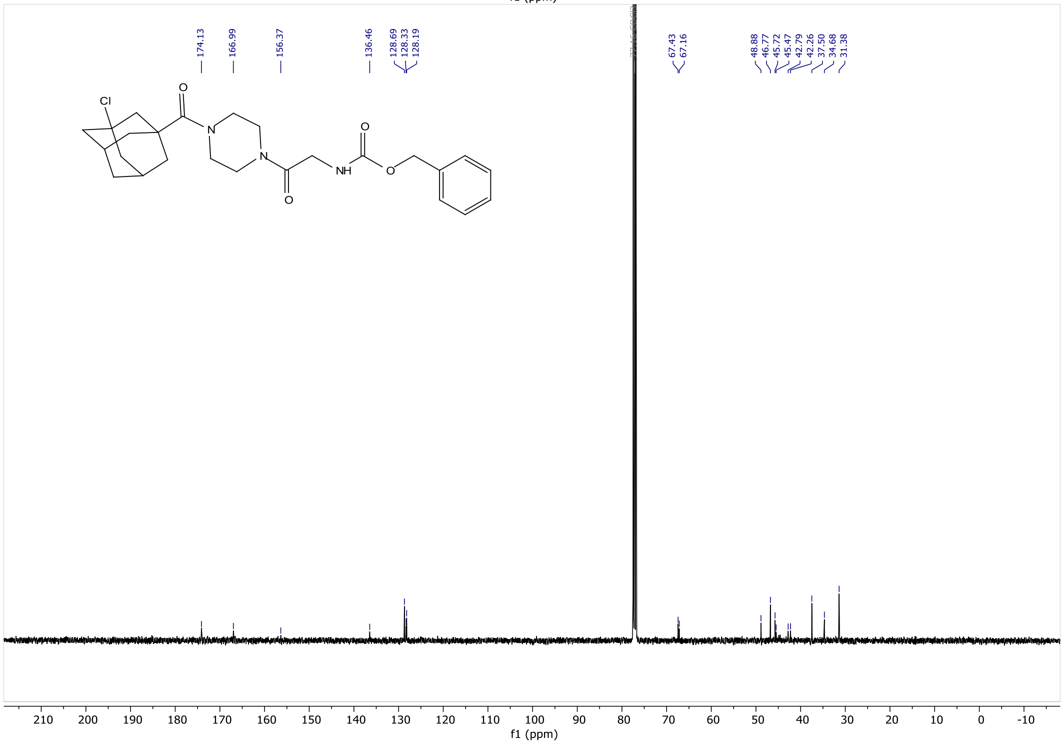

Compound 24c

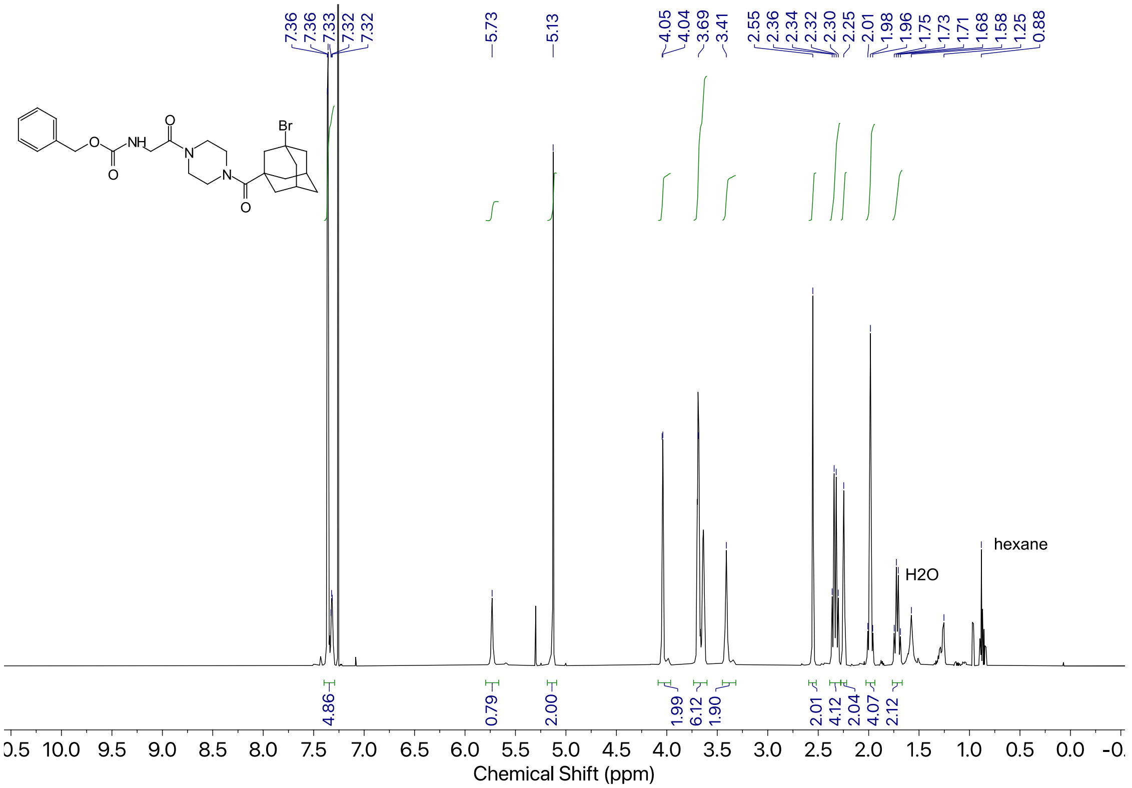

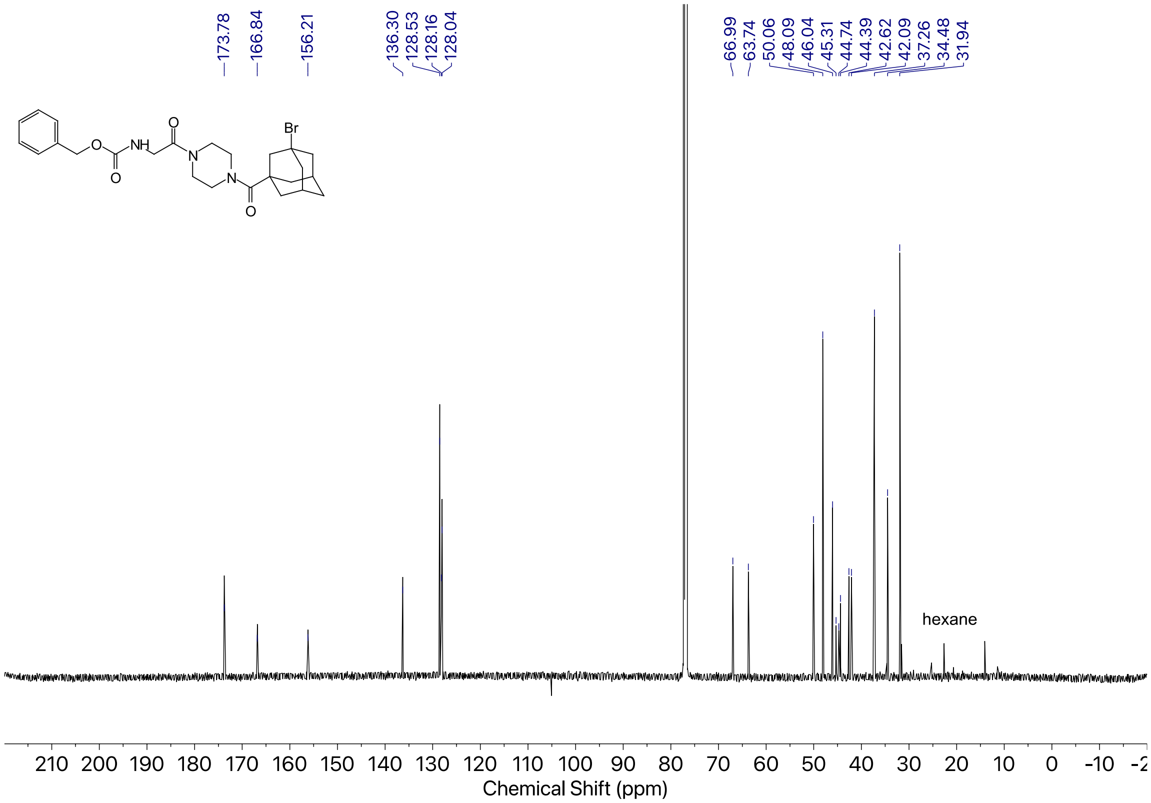

Compound 24d

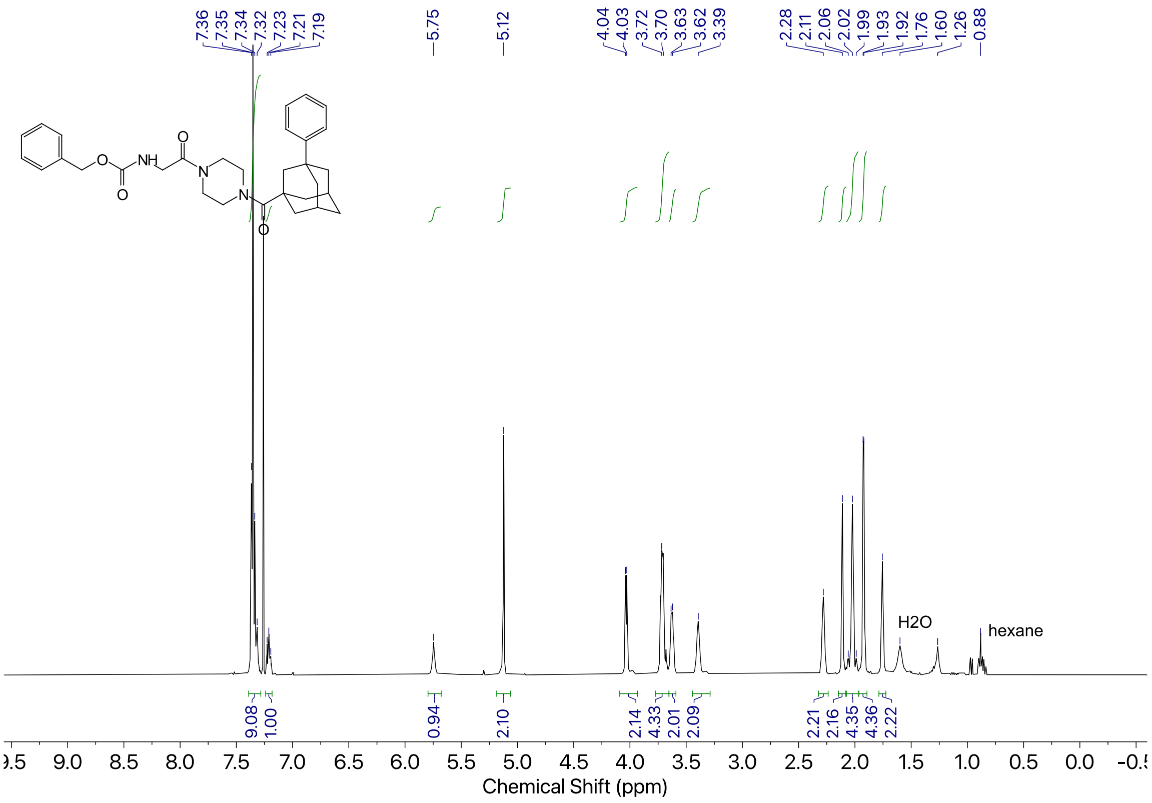

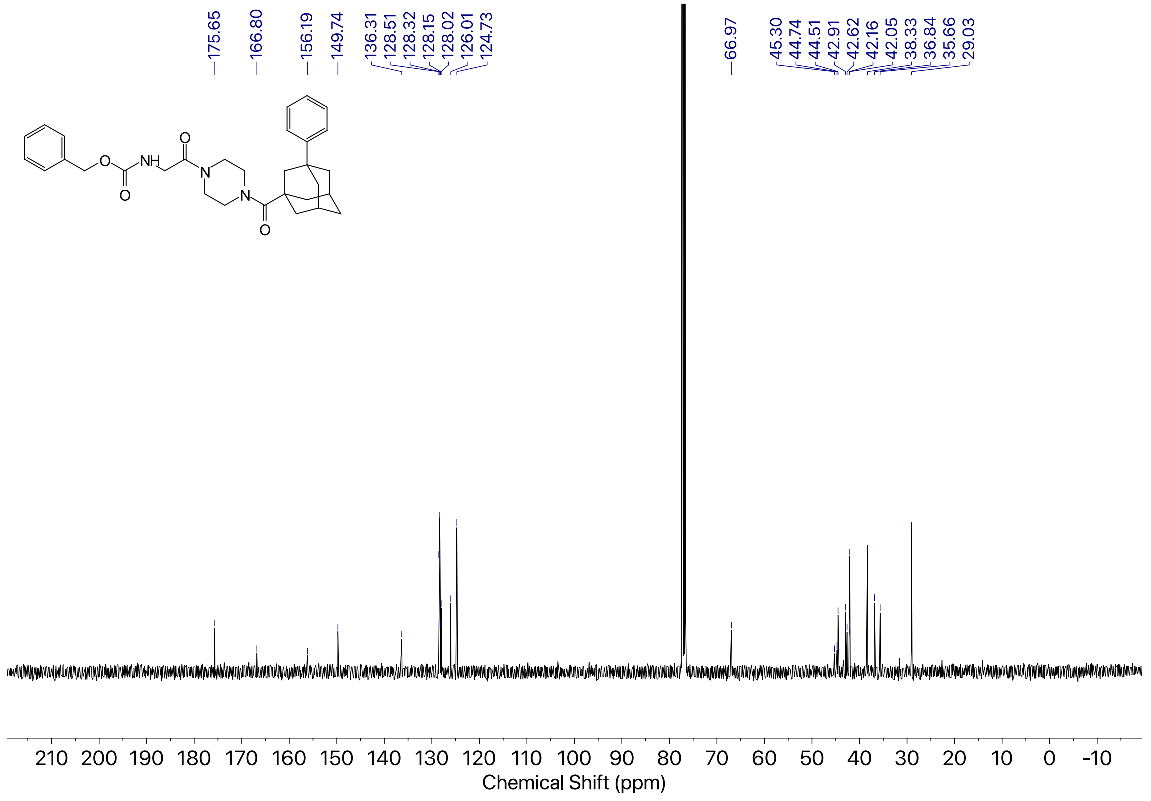

EB-2-16

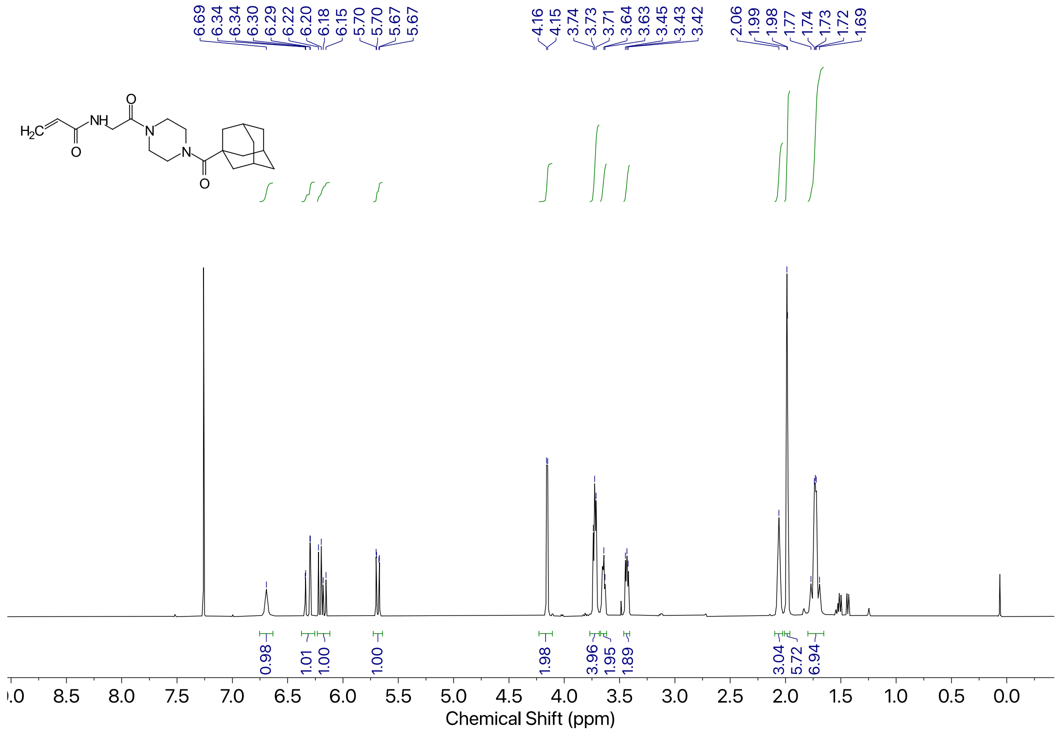
6.69
6.34
6.34
6.30
6.29
6.22
6.20
6.18
6.15
5.70
5.70
5.67
5.67
4.16
4.15
3.74
3.73
3.71
3.64
3.63
3.45
3.43
3.42
2.06
1.99
1.98
1.77
1.74
1.73
1.72
1.69
0.98
1.01
1.00
1.00
1.98
3.96
1.95
1.89
3.04
5.72
6.94
Chemical Shift (ppm)

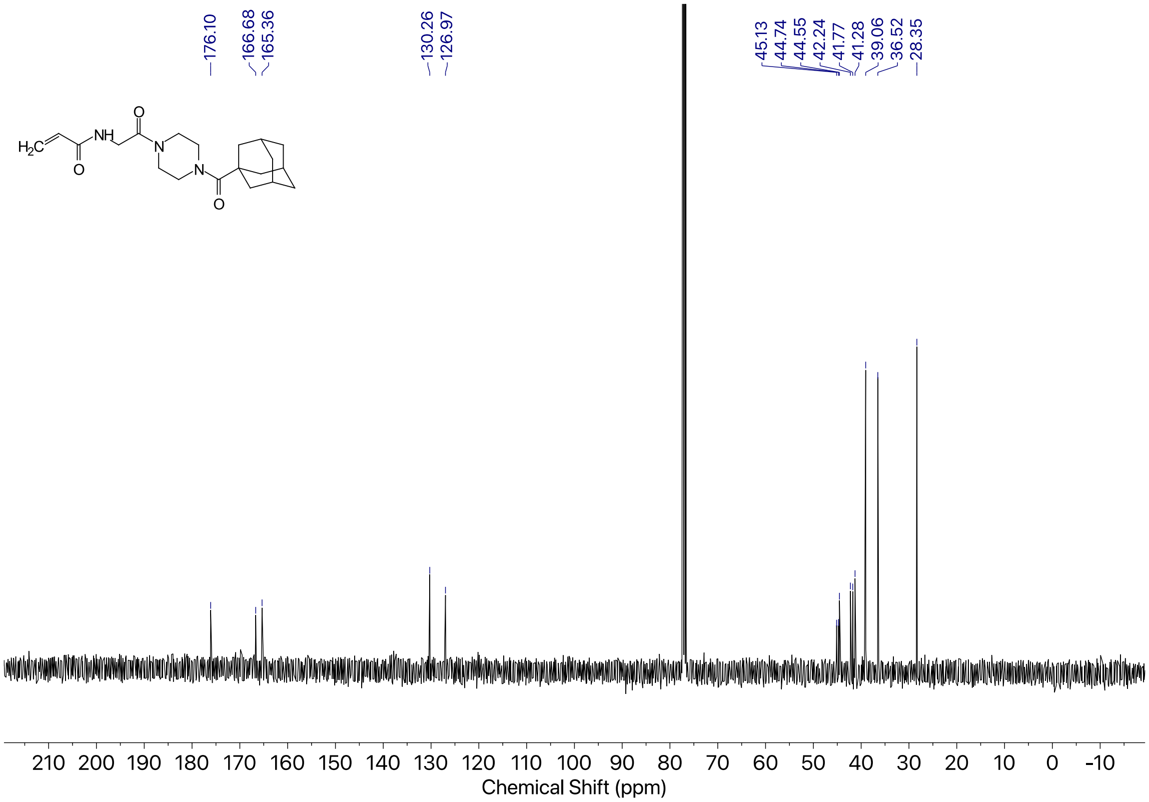
176.10
166.68
165.36
130.26
126.97
45.13
44.74
44.55
42.24
41.77
41.28
39.06
36.52
28.35
210 200 190 180 170 160 150 140 130 120 110 100 90 80 70 60 50 40 30 20 10 0 -10
Chemical Shift (ppm)

Compound 25a

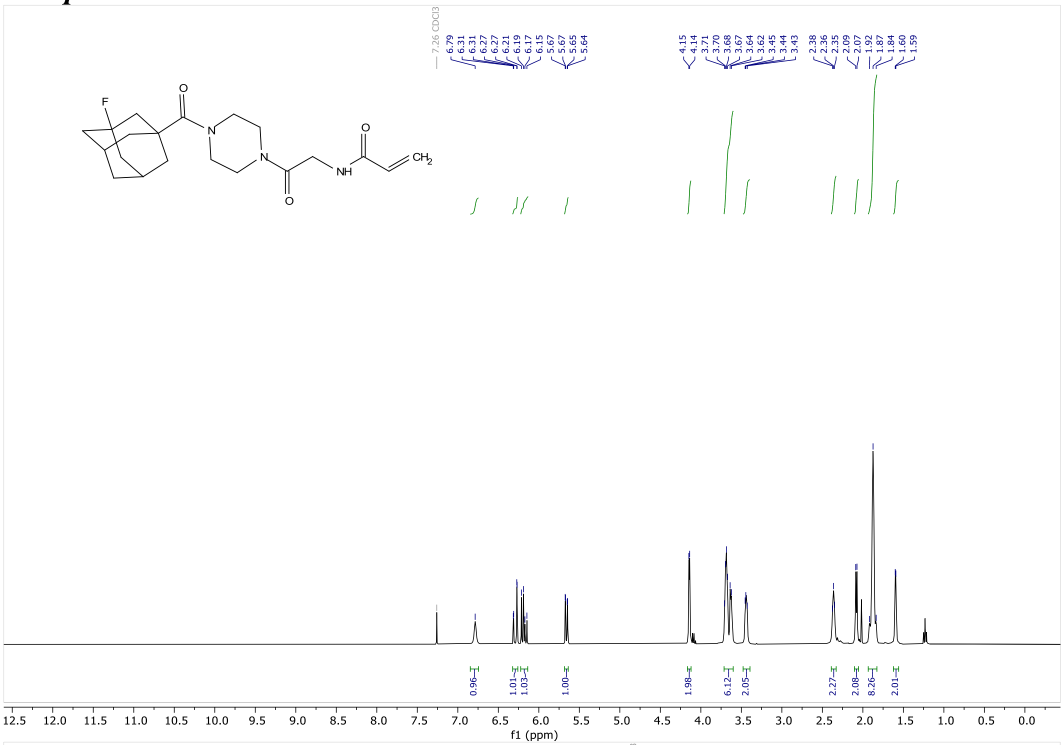

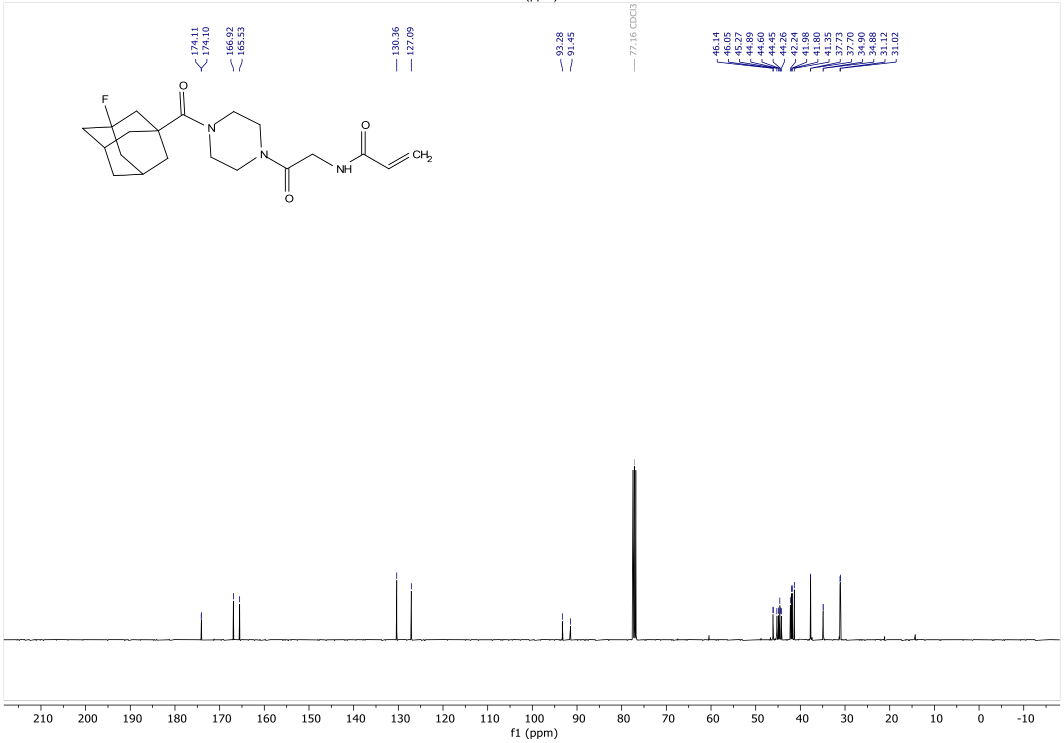

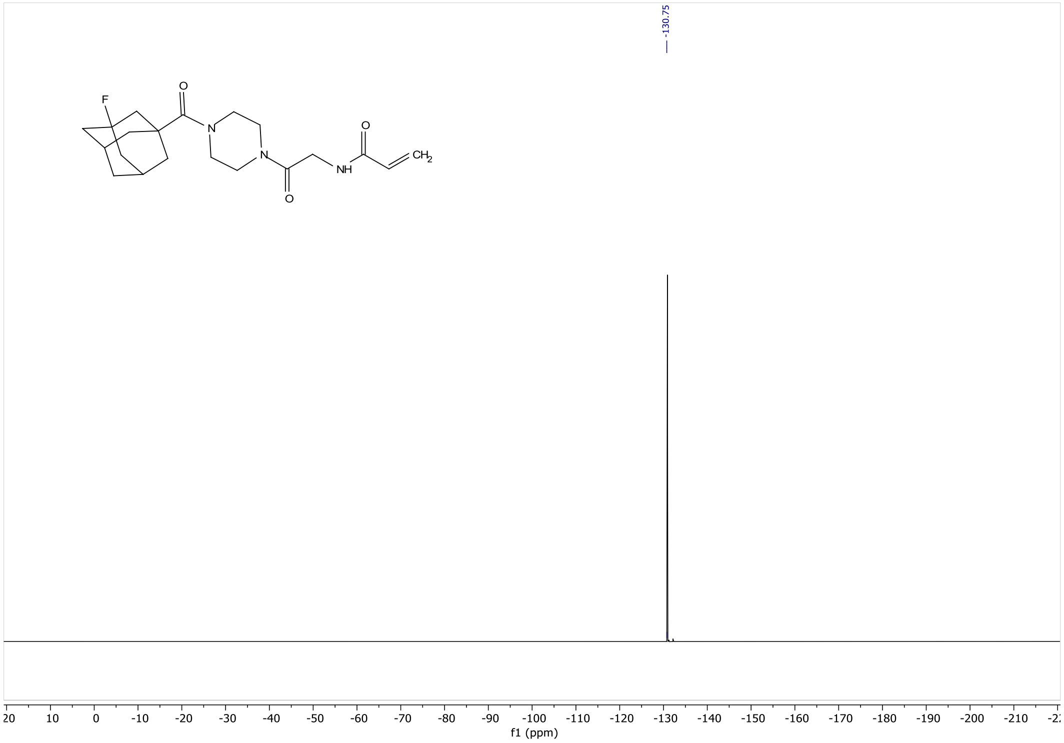

-130.75
F
O
N
N
O
NH
O
CH2
20
10
0
-10
-20
-30
-40
-50
-60
-70
-80
-90
-100
-110
-120
-130
-140
-150
-160
-170
-180
-190
-200
-210
f1 (ppm)

Compound 25b

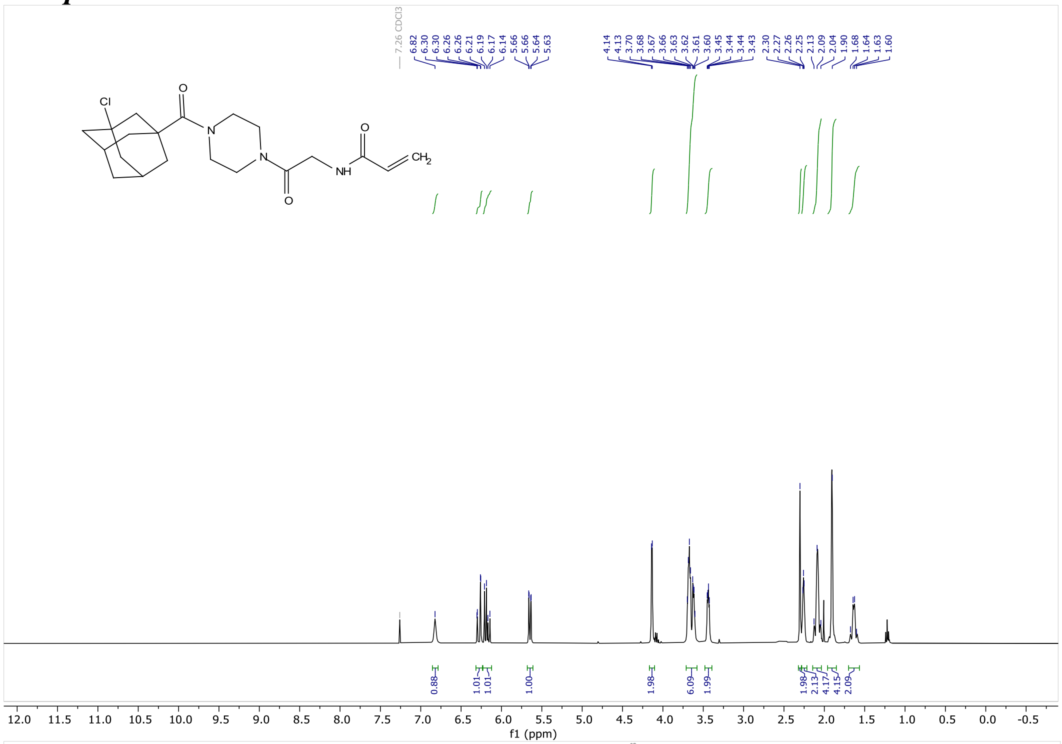

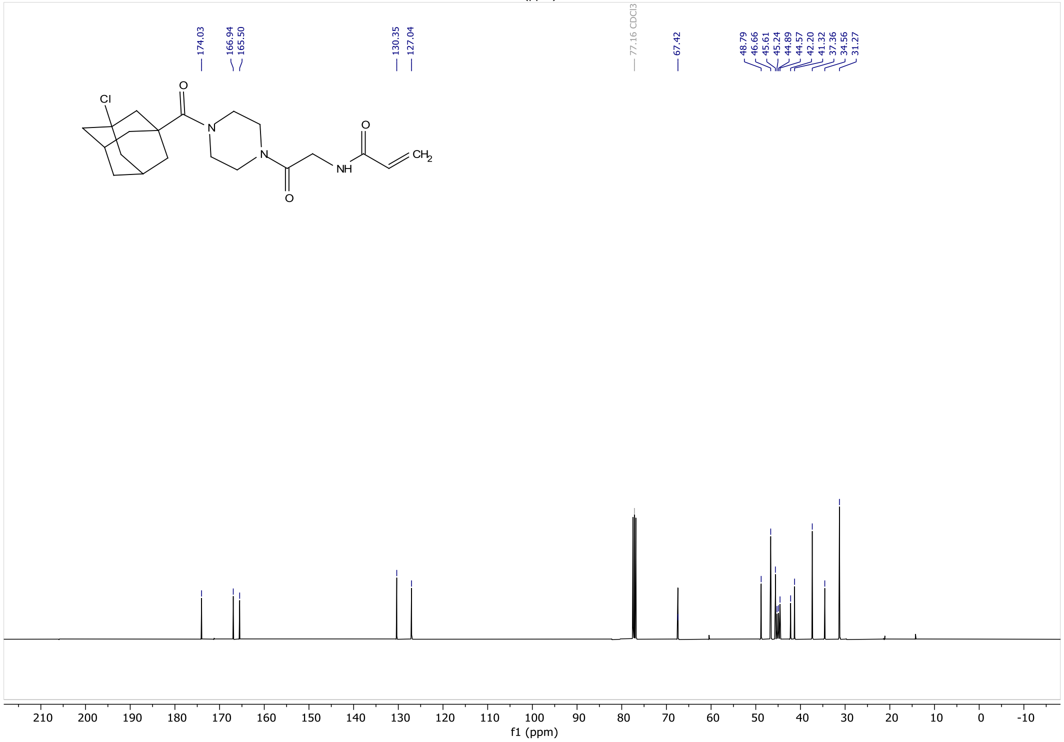

Compound 25c

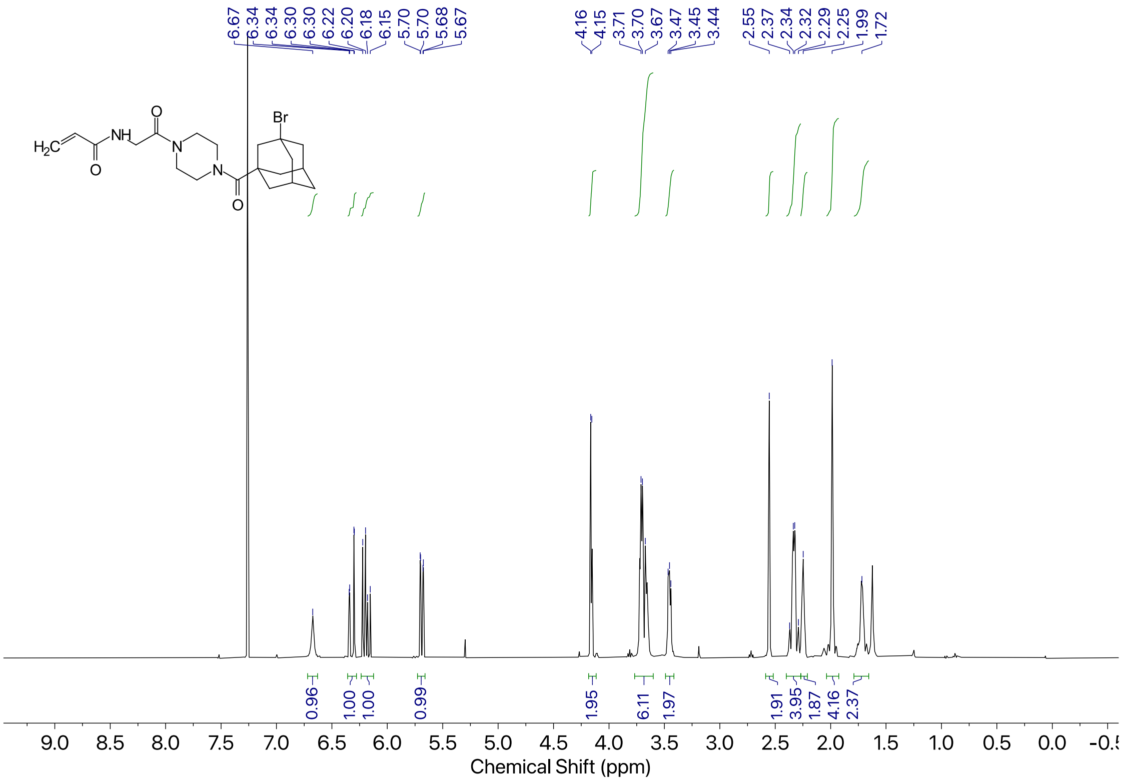

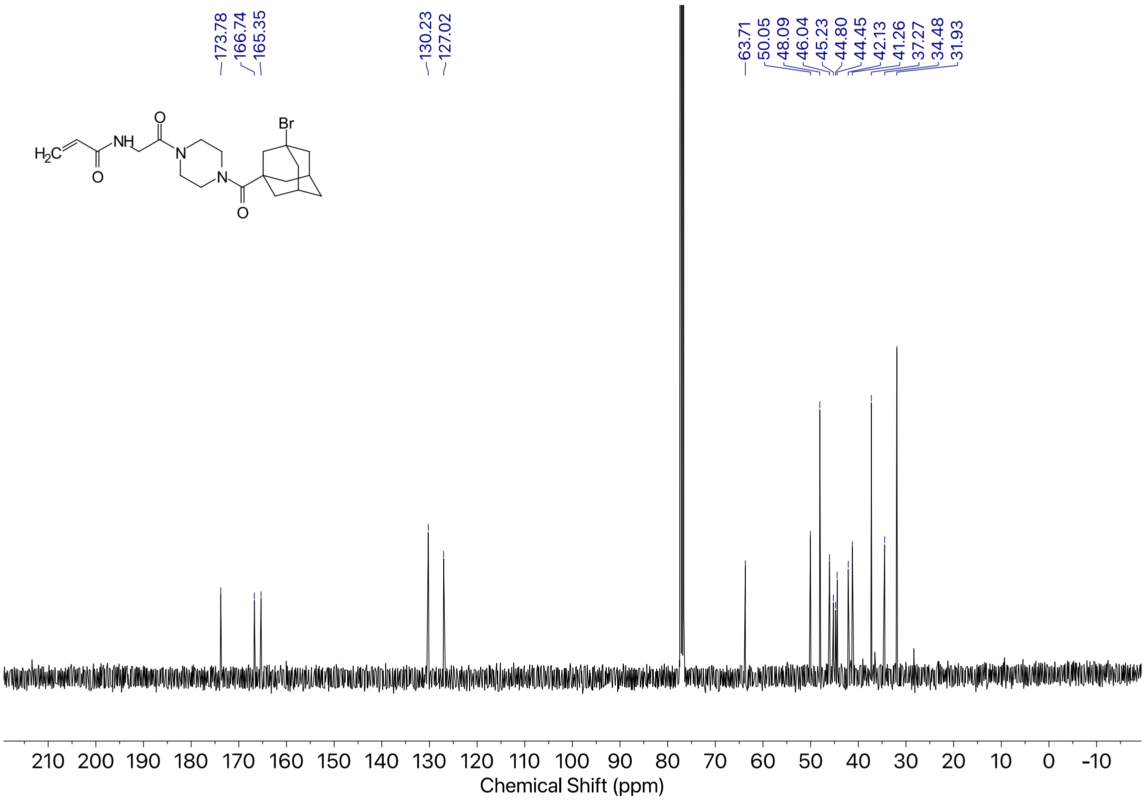

Compound 25d

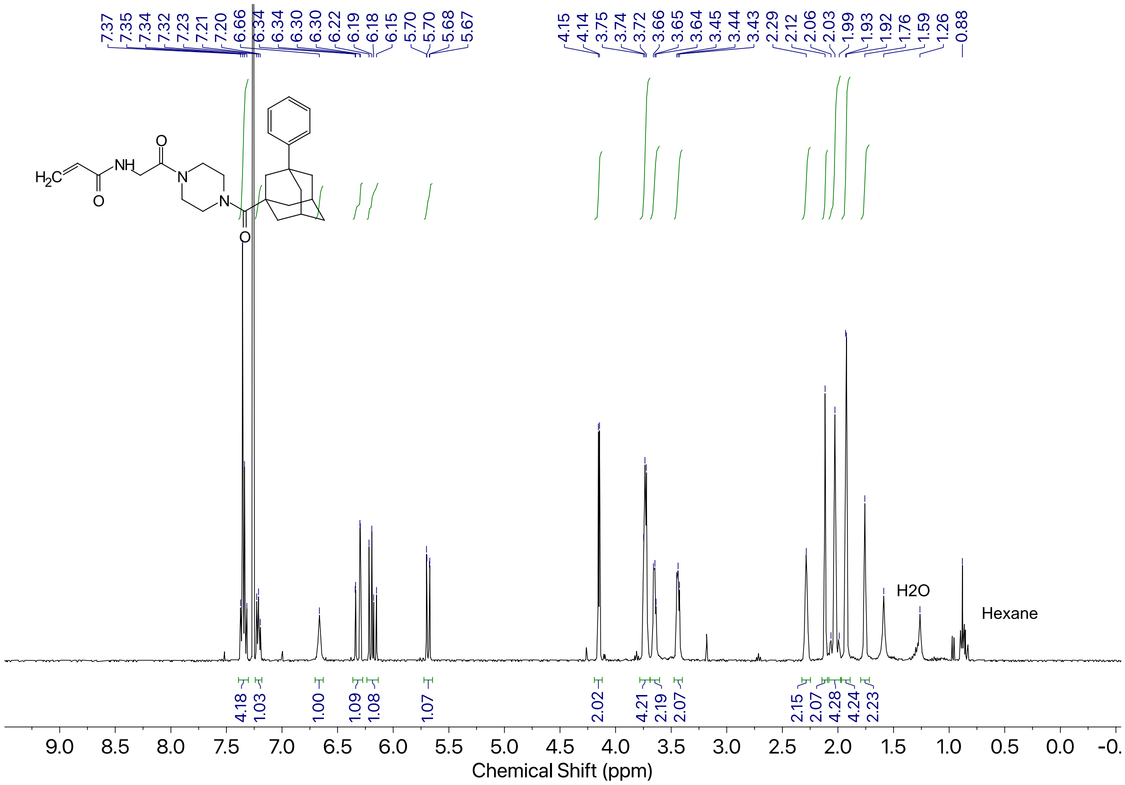

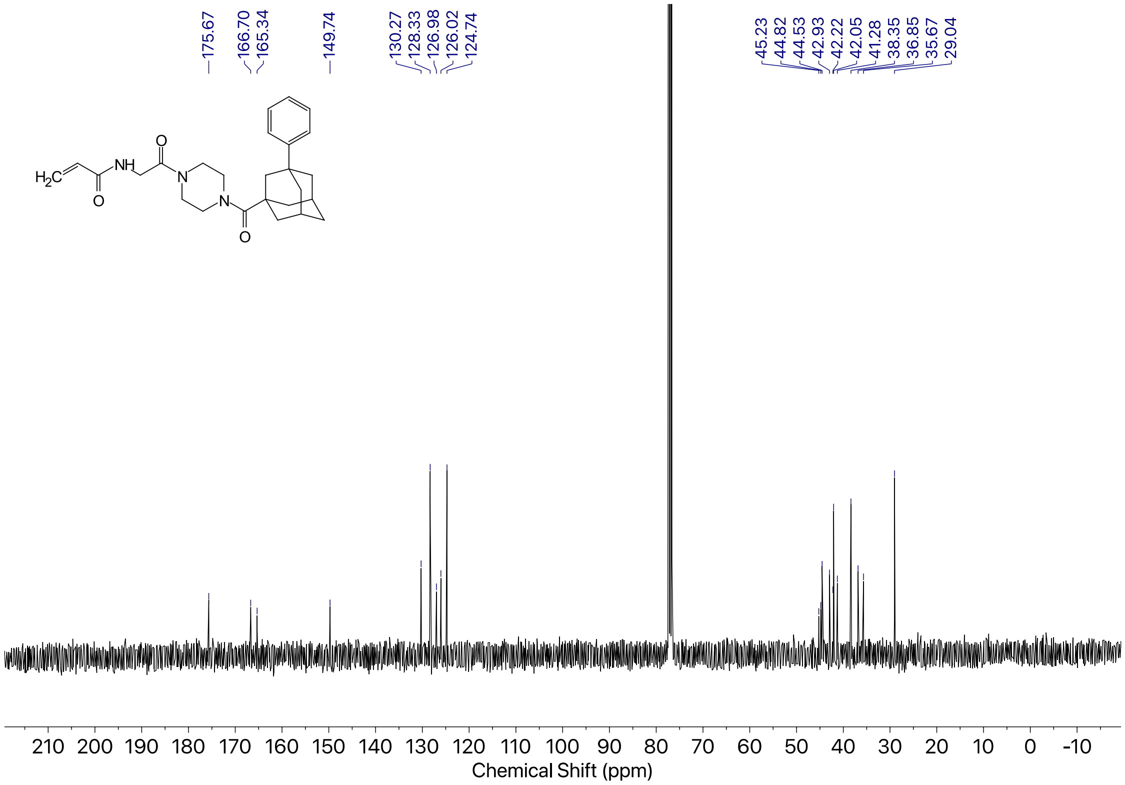

Compound 26

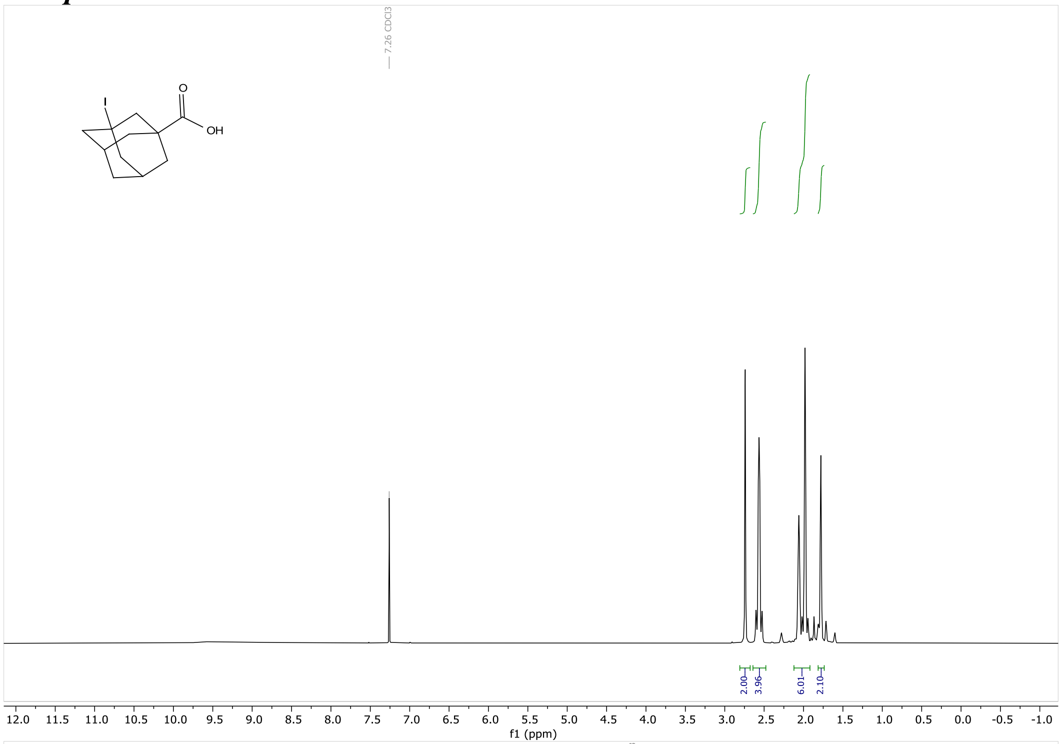

7.26 CDCl3
O
OH
I
2.00
3.96
6.01
2.10
12.0 11.5 11.0 10.5 10.0 9.5 9.0 8.5 8.0 7.5 7.0 6.5 6.0 5.5 5.0 4.5 4.0 3.5 3.0 2.5 2.0 1.5 1.0 0.5 0.0 -0.5 -1.0
f1 (ppm)

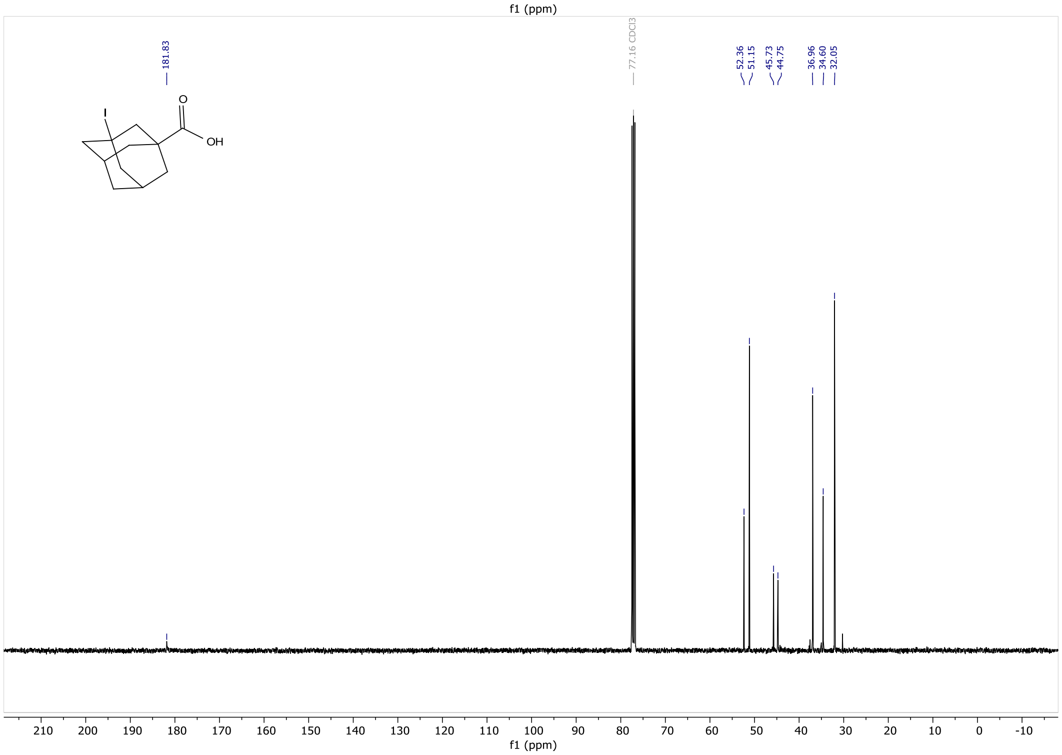

181.83
77.16 CDCl3
52.36
51.15
45.73
44.75
36.96
34.60
32.05
O
OH
I
210 200 190 180 170 160 150 140 130 120 110 100 90 80 70 60 50 40 30 20 10 0 -10
f1 (ppm)

Compound 27

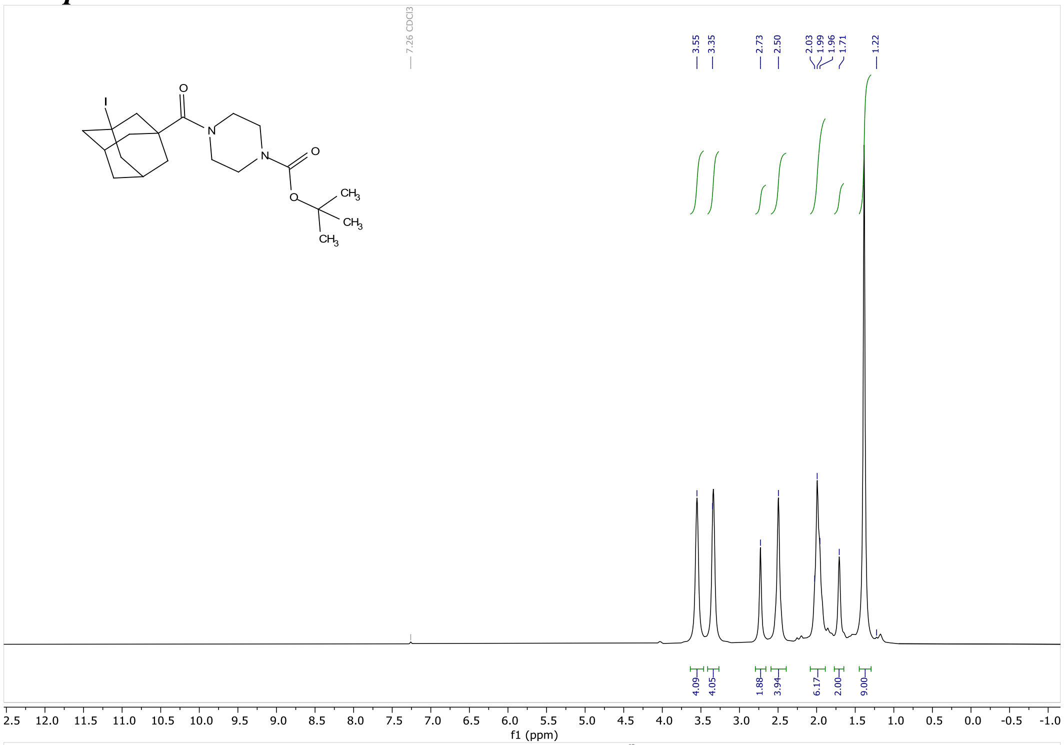
7.26 CDCl3
3.55
3.35
2.73
2.50
2.03
1.99
1.96
1.71
1.22
CH3
CH3
CH3
4.09
4.05
1.88
3.94
6.17
2.00
9.00
f1 (ppm)

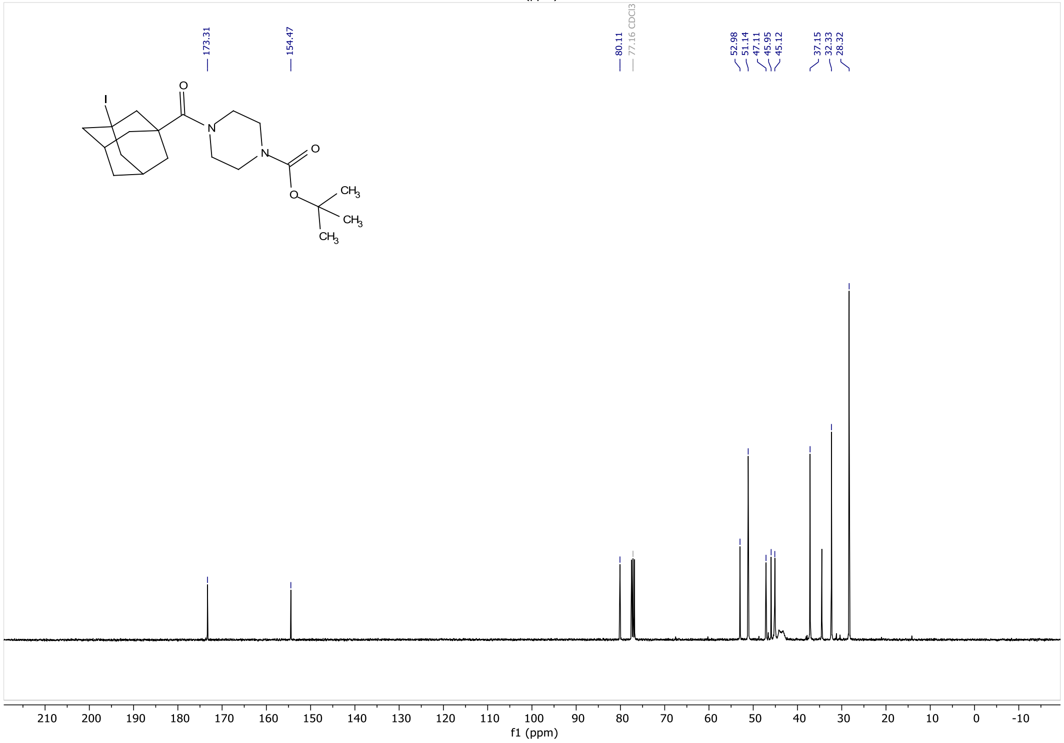
173.31
154.47
80.11
77.16 CDCl3
52.98
51.14
47.11
45.95
45.12
37.15
32.33
28.32
CH3
CH3
CH3
f1 (ppm)

Compound 28

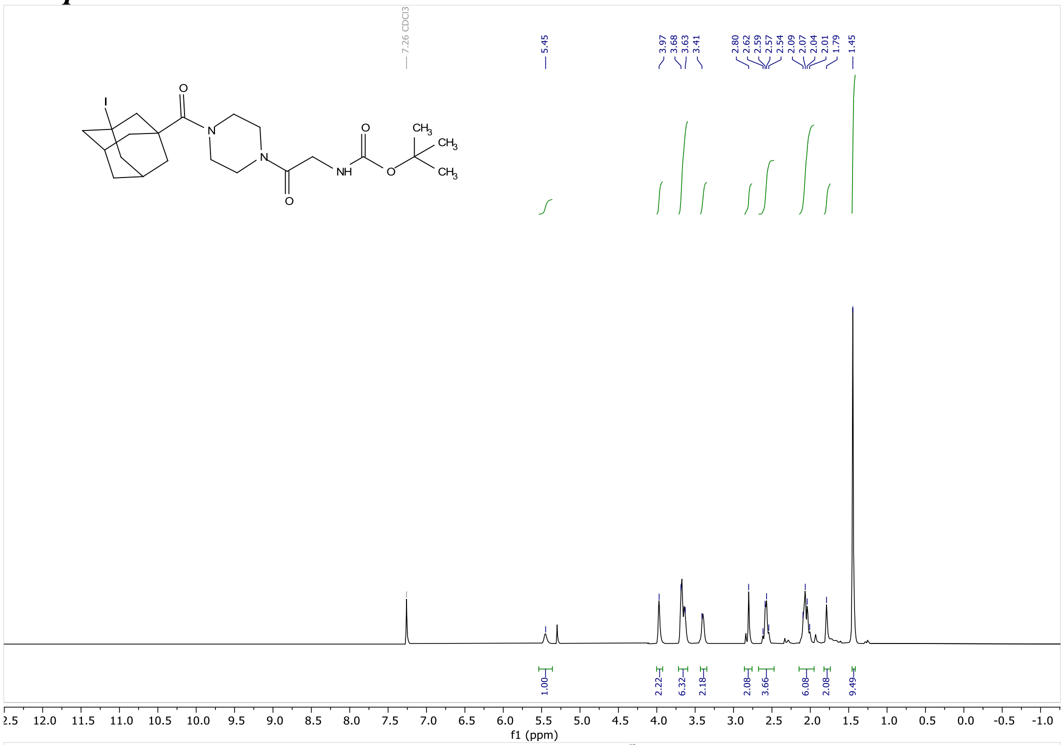

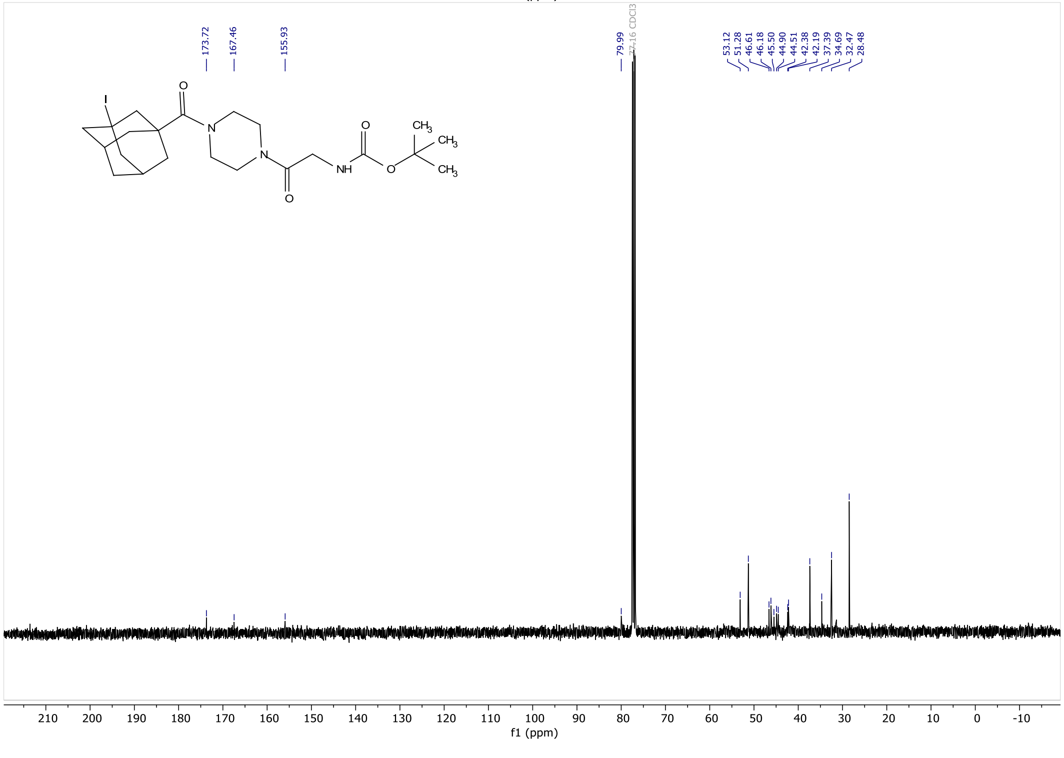

Compound 29

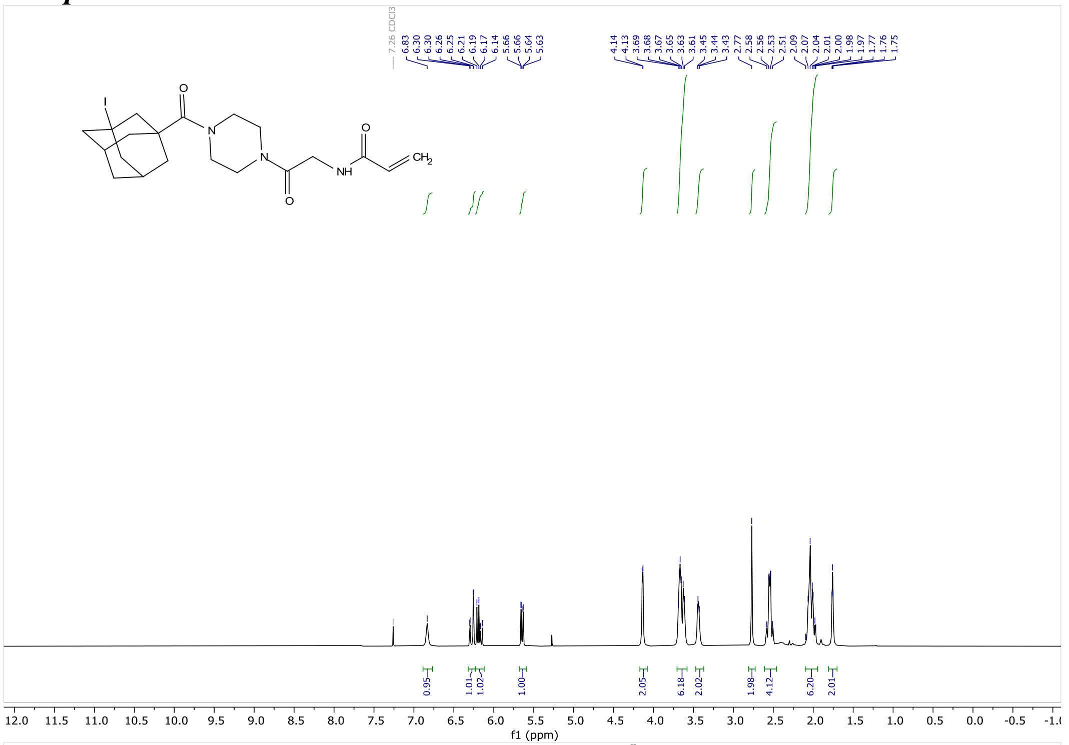

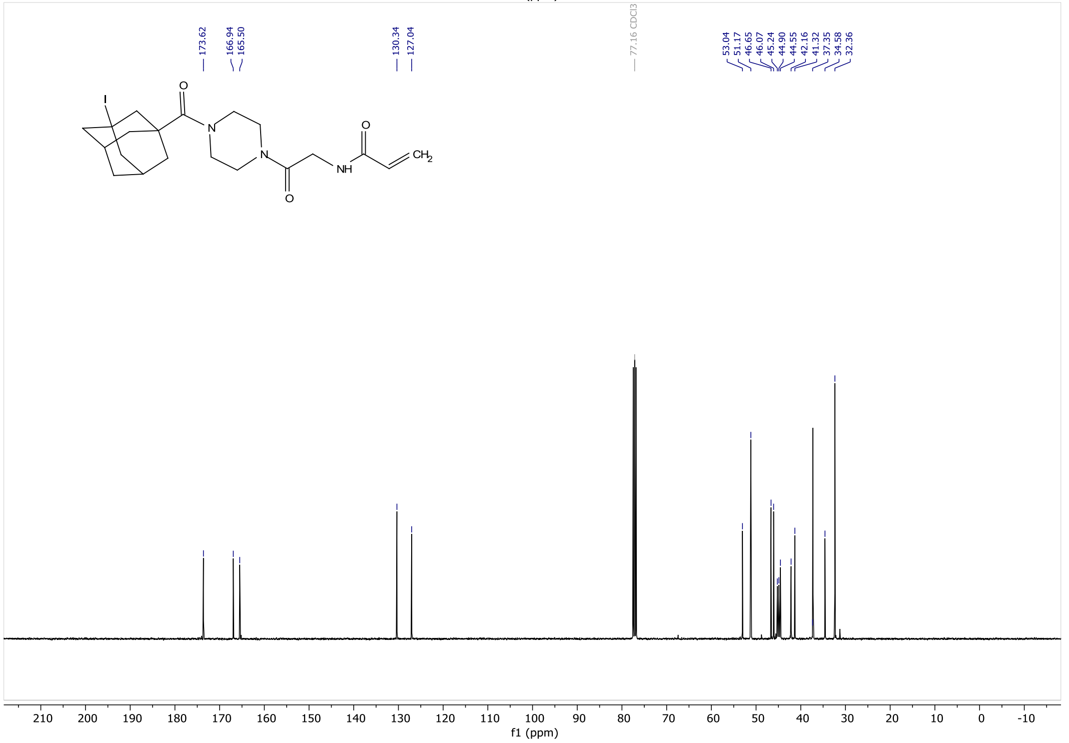

Compound 31

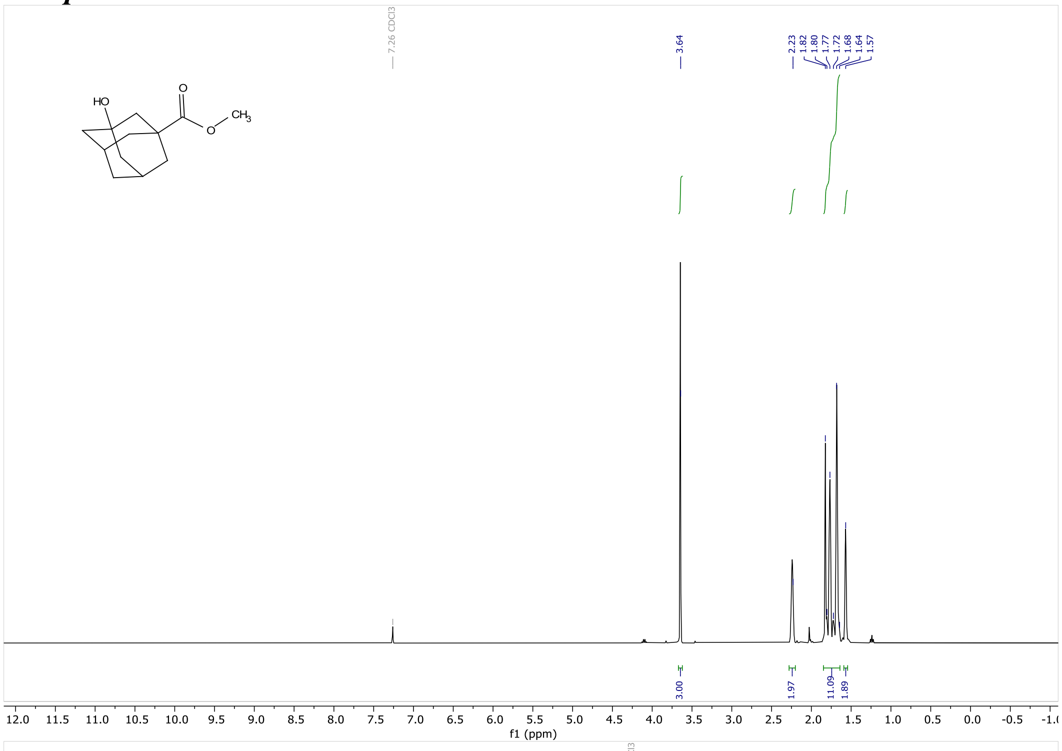

7.26 CDCl3
3.64
2.23
1.82
1.80
1.77
1.72
1.68
1.64
1.57
HO
O
O
CH3
3.00
1.97
11.09
1.89
12.0 11.5 11.0 10.5 10.0 9.5 9.0 8.5 8.0 7.5 7.0 6.5 6.0 5.5 5.0 4.5 4.0 3.5 3.0 2.5 2.0 1.5 1.0 0.5 0.0 -0.5
f1 (ppm)

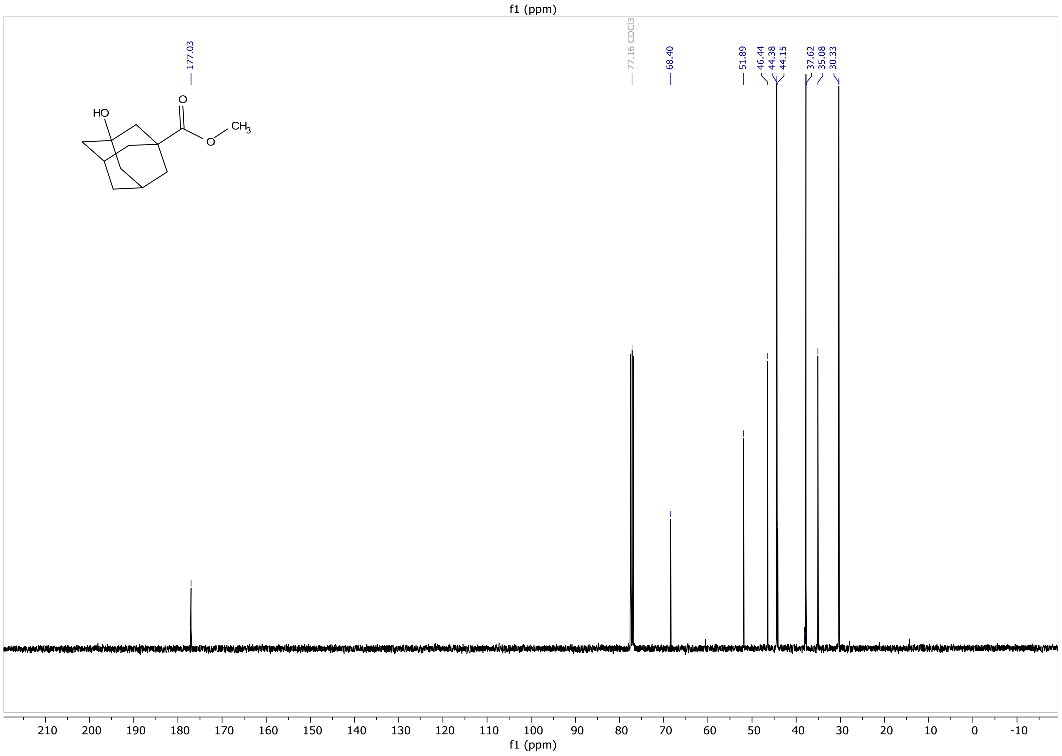

177.03
77.16 CDCl3
68.40
51.89
46.44
44.38
44.15
37.62
35.08
30.33
HO
O
O
CH3
210 200 190 180 170 160 150 140 130 120 110 100 90 80 70 60 50 40 30 20 10 0 -10
f1 (ppm)

Compound 34

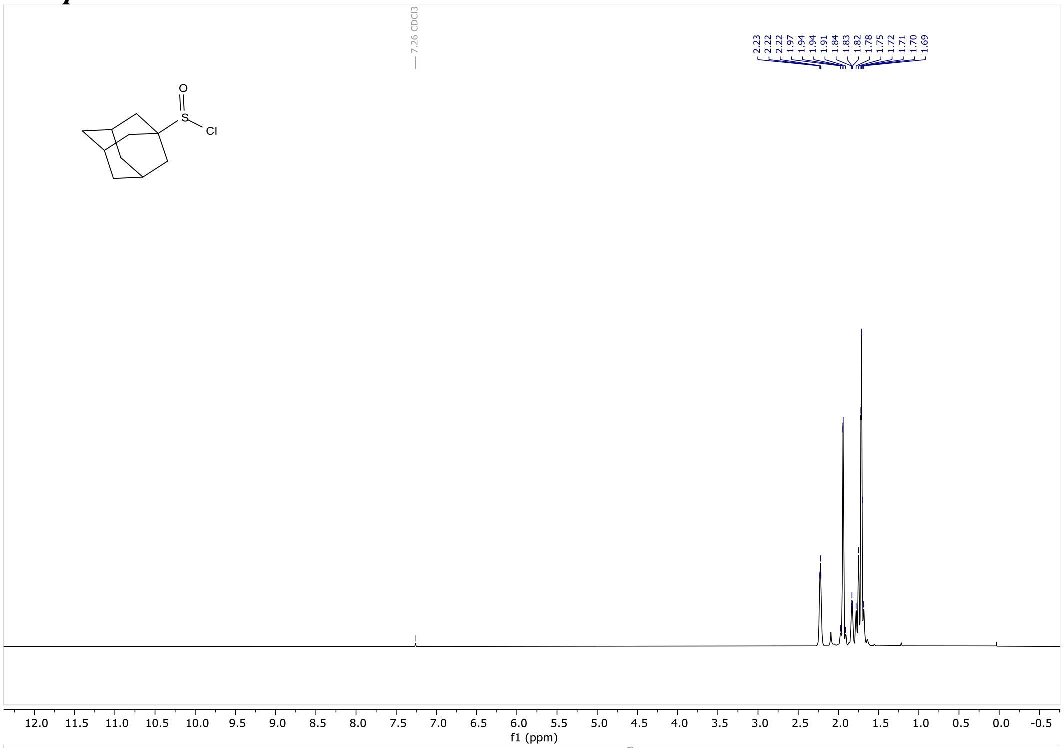
7.26 CDCl3
2.23
2.22
2.22
1.97
1.94
1.94
1.91
1.84
1.83
1.82
1.78
1.75
1.72
1.71
1.70
1.69
O
S
Cl
12.0
11.5
11.0
10.5
10.0
9.5
9.0
8.5
8.0
7.5
7.0
6.5
6.0
5.5
5.0
4.5
4.0
3.5
3.0
2.5
2.0
1.5
1.0
0.5
0.0
-0.5
f1 (ppm)

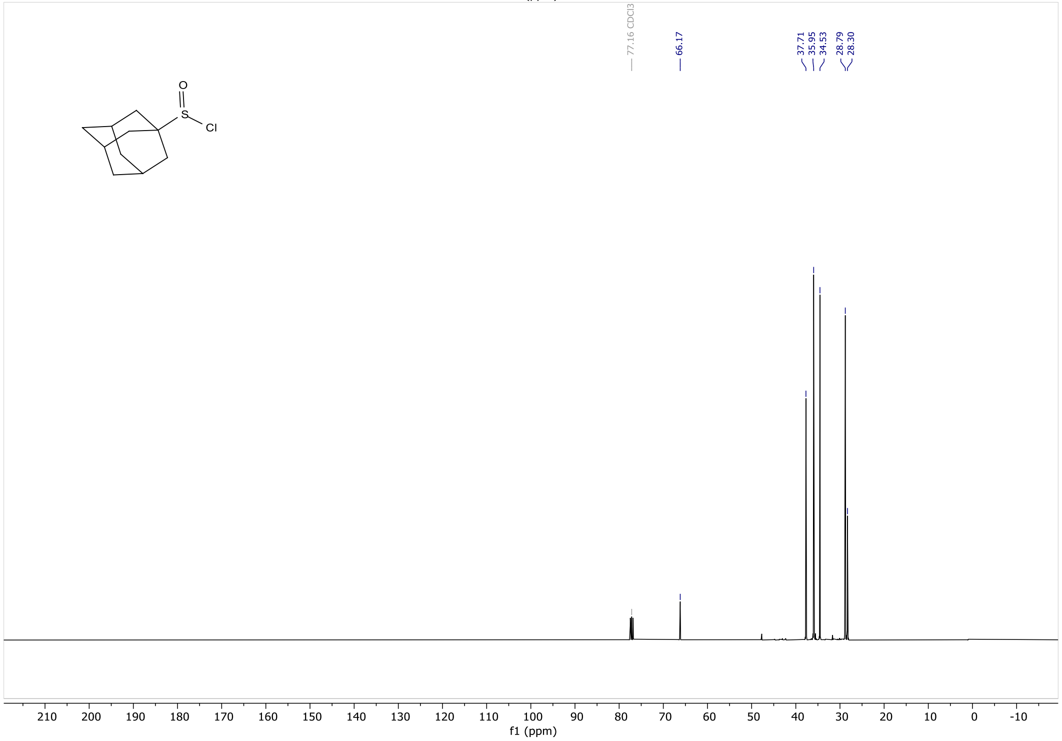
77.16 CDCl3
66.17
37.71
35.95
34.53
28.79
28.30
O
S
Cl
210
200
190
180
170
160
150
140
130
120
110
100
90
80
70
60
50
40
30
20
10
0
-10
f1 (ppm)

Compound 35

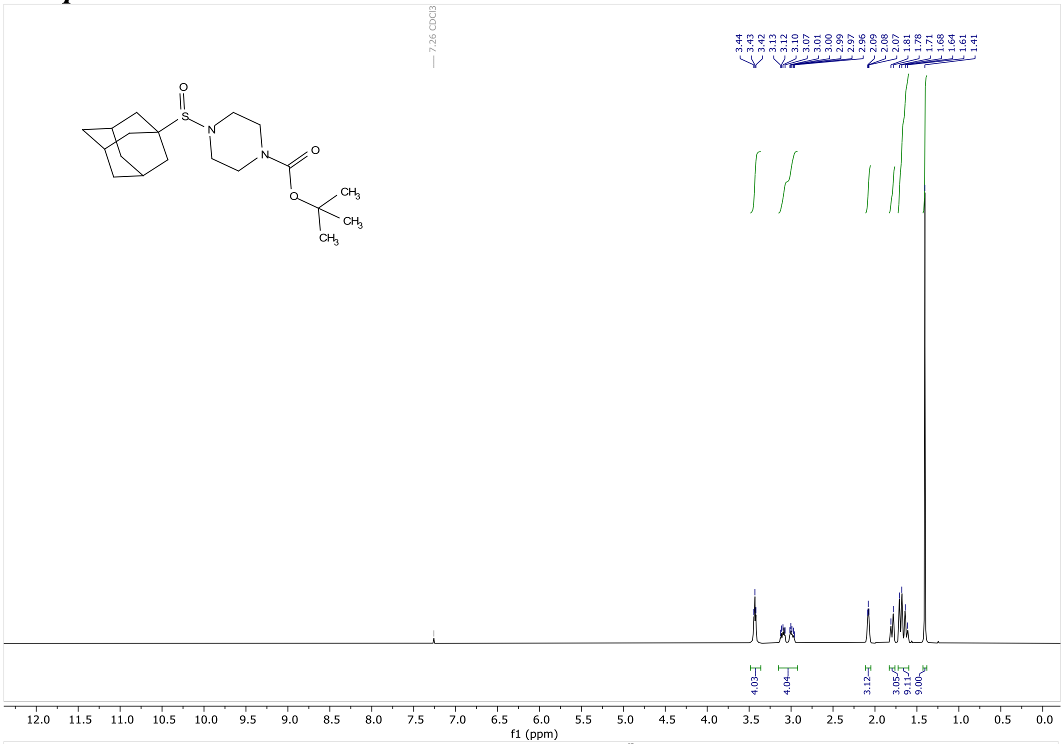
7.26 CDCl3
3.44
3.43
3.42
3.13
3.12
3.10
3.07
3.01
3.00
2.99
2.97
2.96
2.09
2.08
2.07
1.81
1.78
1.71
1.68
1.64
1.61
1.41
4.03
4.04
3.12
3.05
9.11
9.00
O
S
N
N
O
O
CH3
CH3
CH3
f1 (ppm)

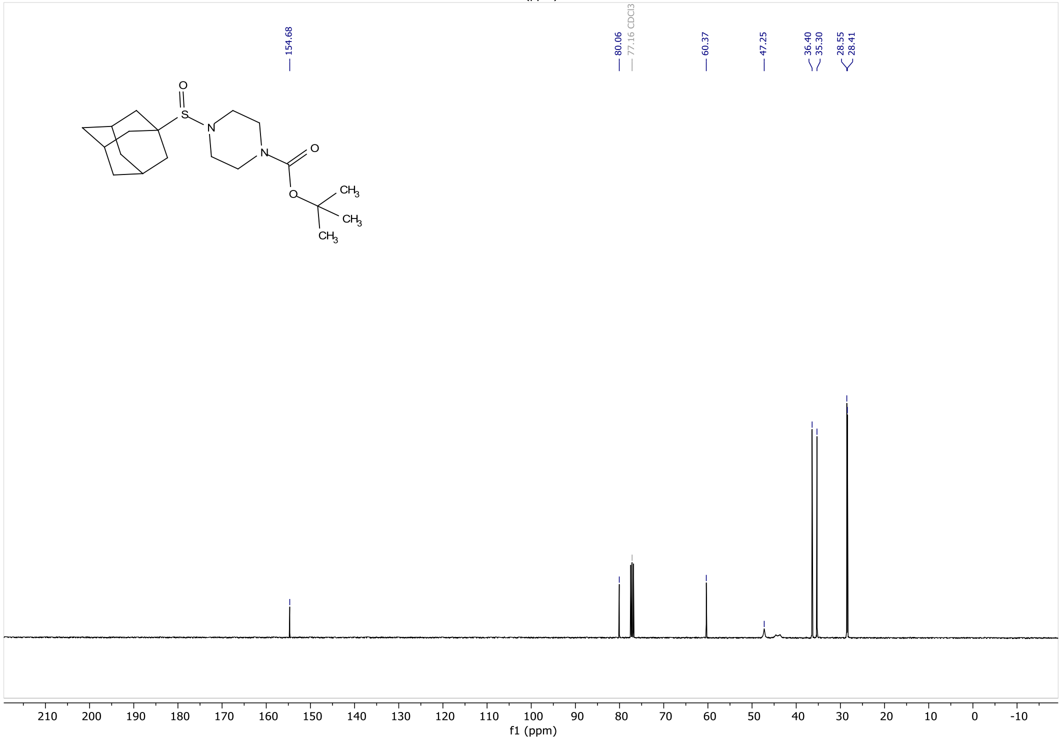
154.68
80.06
77.16 CDCl3
60.37
47.25
36.40
35.30
28.55
28.41
O
S
N
N
O
O
CH3
CH3
CH3
f1 (ppm)

Compound 36

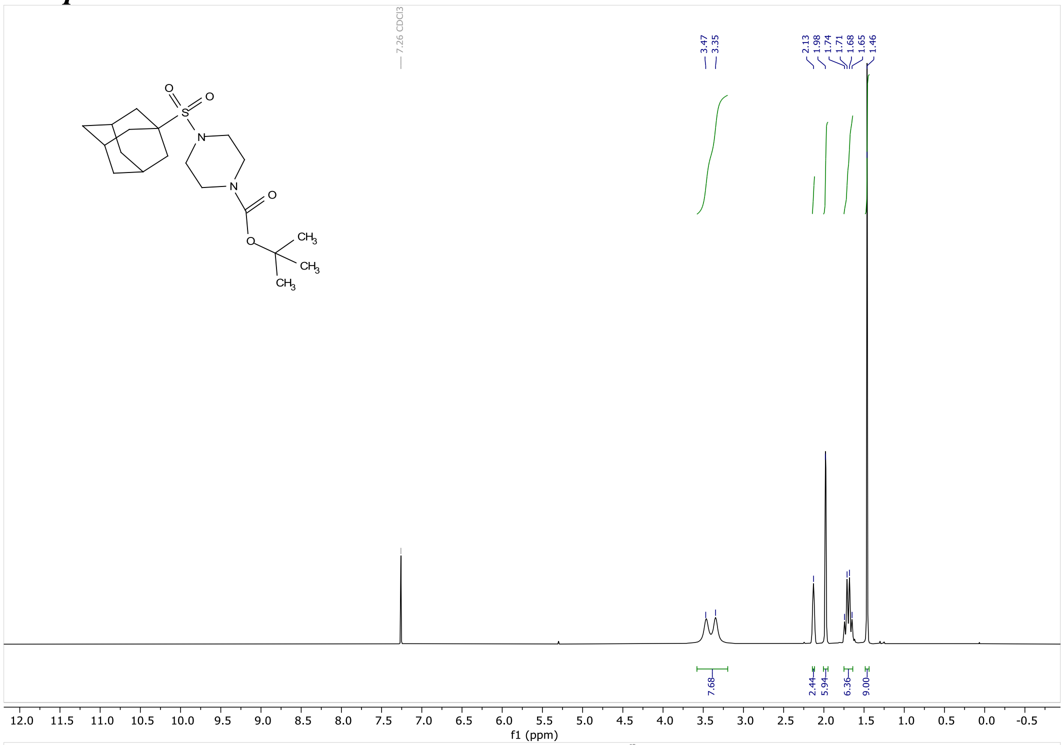
7.26 CDCl3
3.47
3.35
2.13
1.98
1.74
1.71
1.68
1.65
1.46
7.68
2.44
5.94
6.36
9.00
f1 (ppm)

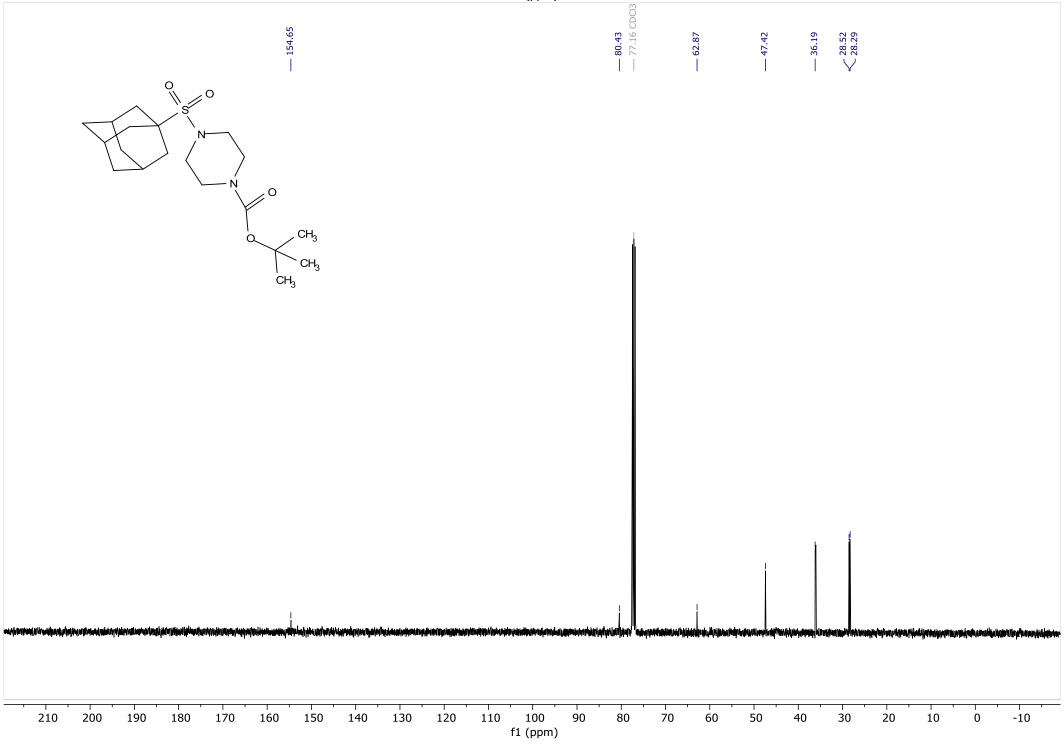
154.65
80.43
77.16 CDCl3
62.87
47.42
36.19
28.52
28.29
f1 (ppm)

Compound 37

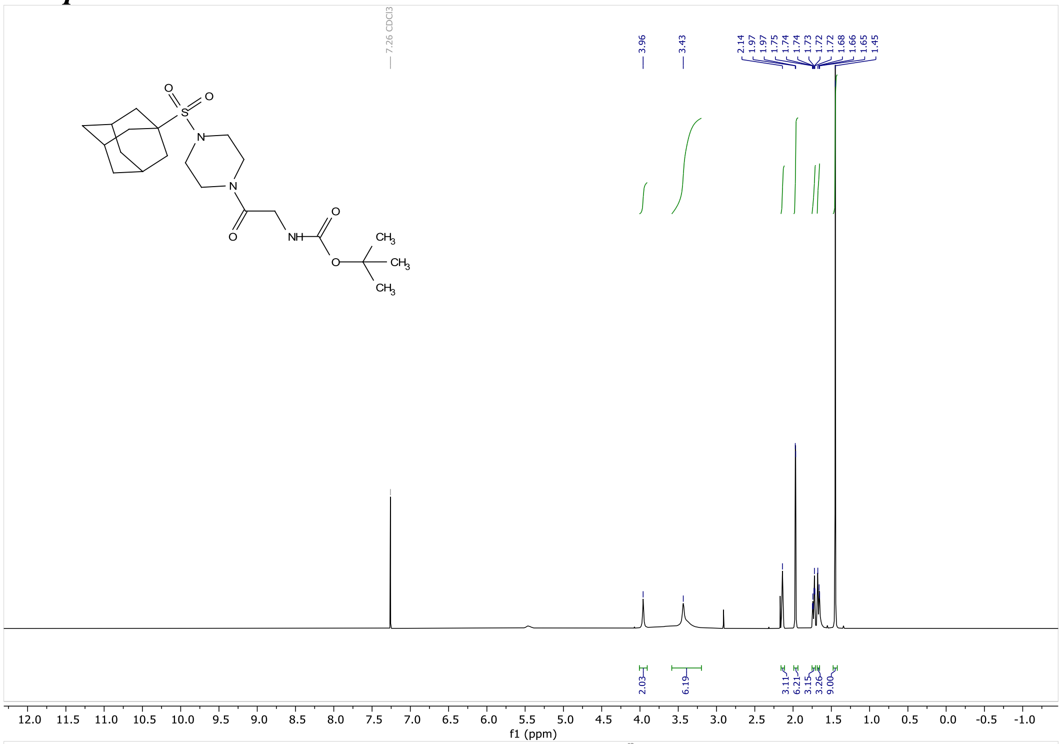
7.26 CDCl3
3.96
3.43
2.14
1.97
1.97
1.75
1.74
1.74
1.73
1.72
1.72
1.68
1.66
1.65
1.45
2.03
6.19
3.11
6.21
3.15
3.26
9.00
f1 (ppm)

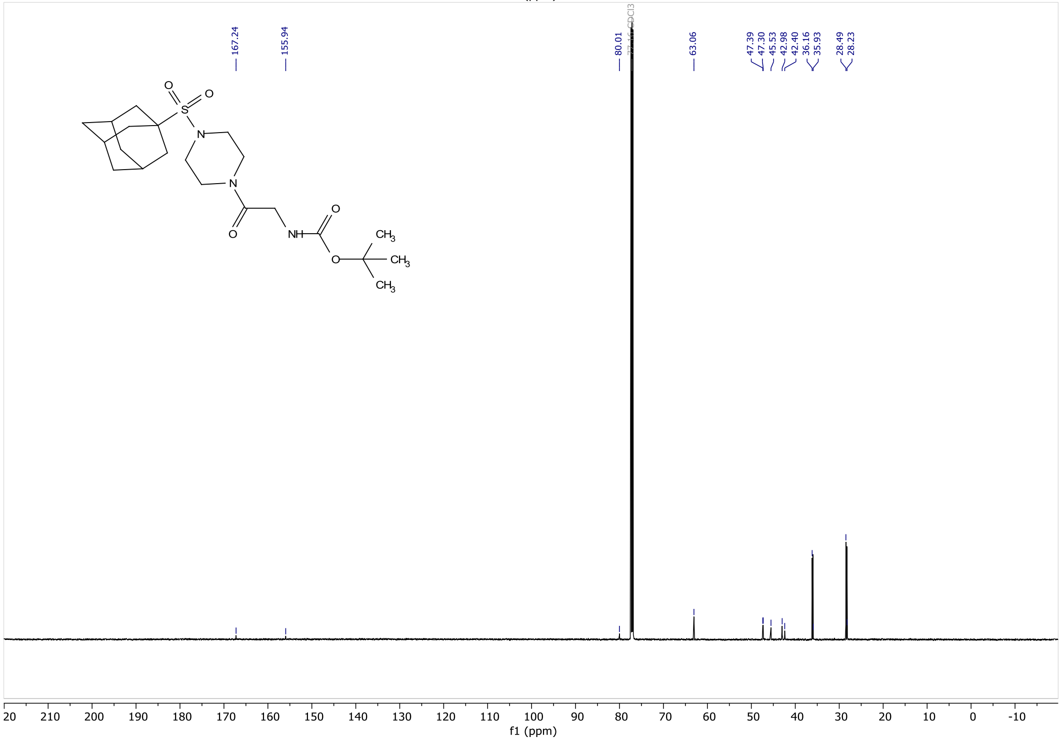
167.24
155.94
80.01
63.06
47.39
47.30
45.53
42.98
42.40
36.16
35.93
28.49
28.23
f1 (ppm)

Compound 38

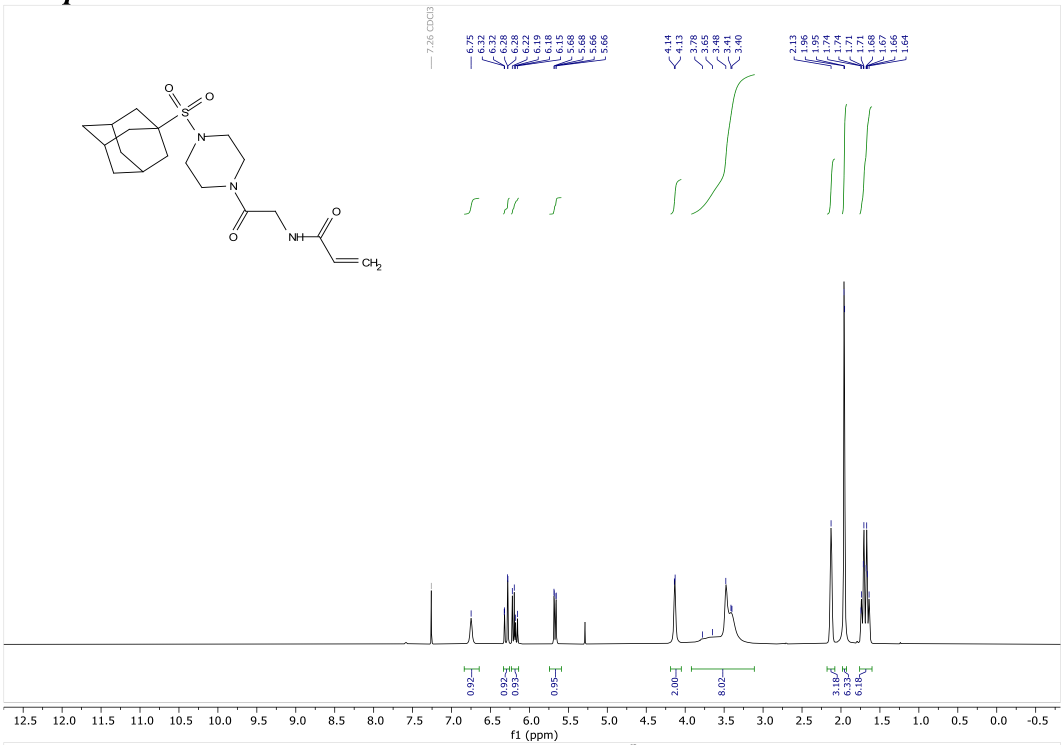

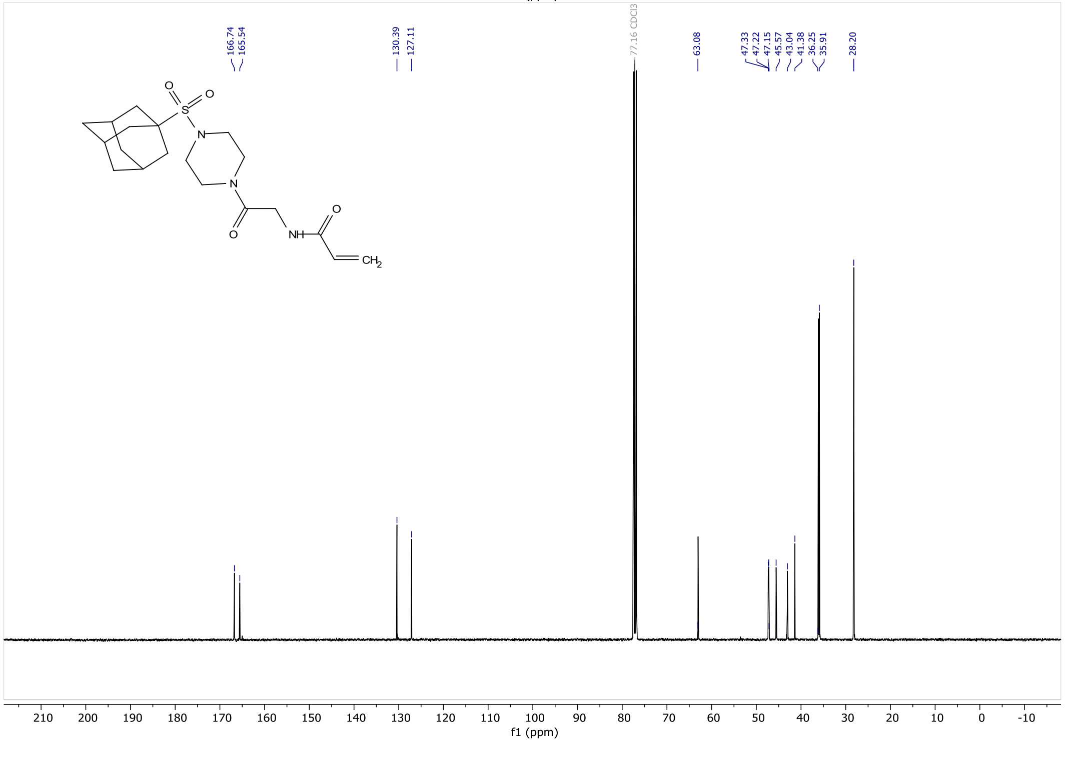

Supplement: MD-017-D5MD00815H-s001 [file MD-017-D5MD00815H-s001.pdf]
